# Radiocarbon, Bayesian chronological modeling and early European metal circulation in the sixteenth-century AD Mohawk River Valley, USA

**DOI:** 10.1371/journal.pone.0226334

**Published:** 2019-12-16

**Authors:** Sturt W. Manning, John P. Hart

**Affiliations:** 1 Cornell Tree Ring Laboratory, Department of Classics, Cornell University, Ithaca, NY, United States of America; 2 Research and Collections Division, New York State Museum, Albany, NY, United States of America; University of Liverpool, UNITED KINGDOM

## Abstract

European metal artifacts in assemblages from sites predating the physical presence of Europeans in Northern Iroquoia in present-day New York, USA and southern Ontario, Canada have been used as chronological markers for the mid-sixteenth century AD. In the Mohawk River Valley of New York, European metal artifacts at sites pre-dating the physical presence of Europeans have been used by archaeologists as a *terminus post quem* (TPQ) of 1525 to 1550 in regional chronologies. This has been done under the assumption that these metals did not begin to circulate until after sustained European presence on the northern Atlantic coast beginning in 1517. Here we use Bayesian chronological modeling of a large set of radiocarbon dates to refine our understanding of early European metal circulation in the Mohawk River Valley. Our results indicate that European iron and cuprous metals arrived earlier than previously thought, by the beginning of the sixteenth century, and cannot be used as TPQs. Together with recent Bayesian chronological analyses of radiocarbon dates from several sites in southern Ontario, these results add to our evolving understanding of intra-regional variation in Northern Iroquoia of sixteenth-century AD circulation and adoption of European goods.

## Introduction

Over millennia Native Americans in the Northeast participated in geographically extensive trade and exchange networks moving raw materials such as marine shell, copper, and tool stone hundreds of kilometers from their sources [1–3]. Participants in these networks included ancestors of Iroquoian-speaking peoples in present-day New York, Ontario, and Quebec (Northern Iroquoia) (e.g., [4]). European presence on the northern Atlantic coast beginning in the late fifteenth/initial sixteenth century introduced new materials into these networks [5] (all dates in this paper are AD). The occurrence of objects fashioned from European metals including iron, copper alloy, and brass on interior archaeological sites pre-dating the physical presence of Europeans has been viewed as a chronological marker for the mid-sixteenth century including in regional chronologies of Iroquoian sites (e.g., [5–7]).

Thule people in southern Labrador were interacting with European explorers and fishermen and adopting European metals and beads by the late fifteenth century [8]. Beothuk individuals encountered by Europeans in 1501 in Newfoundland at the east side of the Gulf of St. Lawrence possessed European metals including a pair of silver earrings and piece of a gilt sword [9]. Both of these suggest the possibility of European metal circulation into the interior at the beginning of the sixteenth century, or earlier [10, 11]. The finding of an iron object at the ~1500–1550 Mantle (or Jean-Baptiste Lainé) site in southern Ontario was the best-documented early evidence of European metals in Northern Iroquoia [12]. However, recent re-dating of the site with a large number of radiocarbon dates (n = 41) and Bayesian modeling indicates an occupation of ~1596–1618 [13], thus raising questions about the timeframe for early exchange of European metals in the region and, along with new Bayesian dating of other southern Ontario sites, the appropriateness of presence or absence of metal objects as chronological markers in northern Iroquoia [13, 14].

One Iroquoian region where early European metals have been employed as key chronological markers is the Mohawk River Valley in present-day New York State. Presence of European metals has been used there as a *terminus post quem* (TPQ) of ca.1520–1525 for site occupations (e.g., [15–17]) to 1550 [18] under the assumption that circulation of European metals did not initiate until the sustained presence of Europeans in the Gulf of St. Lawrence beginning in 1517 [19]. For example, in a pioneering chronology-building project, Snow [17, 20] obtained 38 AMS radiocarbon dates on maize kernels, which he combined with pottery type frequencies and the presence and kinds of European artifacts, to build a chronology for fifteenth- through seventeenth-century ancestral Iroquoian sites in the Mohawk Valley. The presence of European metal on pre-contact sites was used by Snow as a TPQ of 1525 for the occupations of those sites.

There have been several substantial developments in radiocarbon dating since Snow’s project. These include refinements in sample pretreatment and AMS dating resulting in the ability to date much smaller samples but with considerably greater precision (substantially smaller radiocarbon age error terms), on-going calibration curve refinements through several iterations of the mid-latitude northern hemisphere radiocarbon calibration curve, and the advent of Bayesian analysis of large radiocarbon age datasets which takes into consideration prior information about archaeological sites and regional sequences enabling the building of robust site and regional chronology models at much increased resolution [21–27]. As recently demonstrated, the application of Bayesian modeling with large suites of AMS radiocarbon dates can substantially alter traditional archaeological chronologies in Northern Iroquoia [13, 14].

Here, we use 102 radiocarbon dates, including 60 AMS radiocarbon dates reported for the first time, with Bayesian modeling to refine the chronology of the key fifteenth- and sixteenth-century Mohawk Valley site chronology (see Methods). This is the first use of radiocarbon dates to build a chronology for fifteenth- and sixteenth-century sites in the Mohawk Valley independent of considerations of changes in artifact frequencies and presence–absences and quantities of European artifacts. While others have suggested chronologies for selected series of sites in the Mohawk River basin (e.g. [15, 28–30]), we use Snow’s [17] results as a baseline for comparison with our new modeling results because his was the most comprehensive prior effort based at least in part on radiocarbon dating. The primary goals for our analyses are arriving at an independent, robust and refined understanding of the Mohawk sequence and the early circulation of European metals in the valley. Our focus is on four key sites attributed to the mid- to late-sixteenth century (1525–1580) by Snow [17] that have European iron and/or cuprous artifacts that may be of European origin. We subject the latter to pXRF analysis to determine the presence of trace elements that indicate European smelting. We consider how the resultant revised timescale coupled with the presence of European metal artifacts—and with comparison to work elsewhere in the Northeast—informs new perspectives on early European metal circulation among Iroquoian societies through this period.

## Results

**Fig 1:**
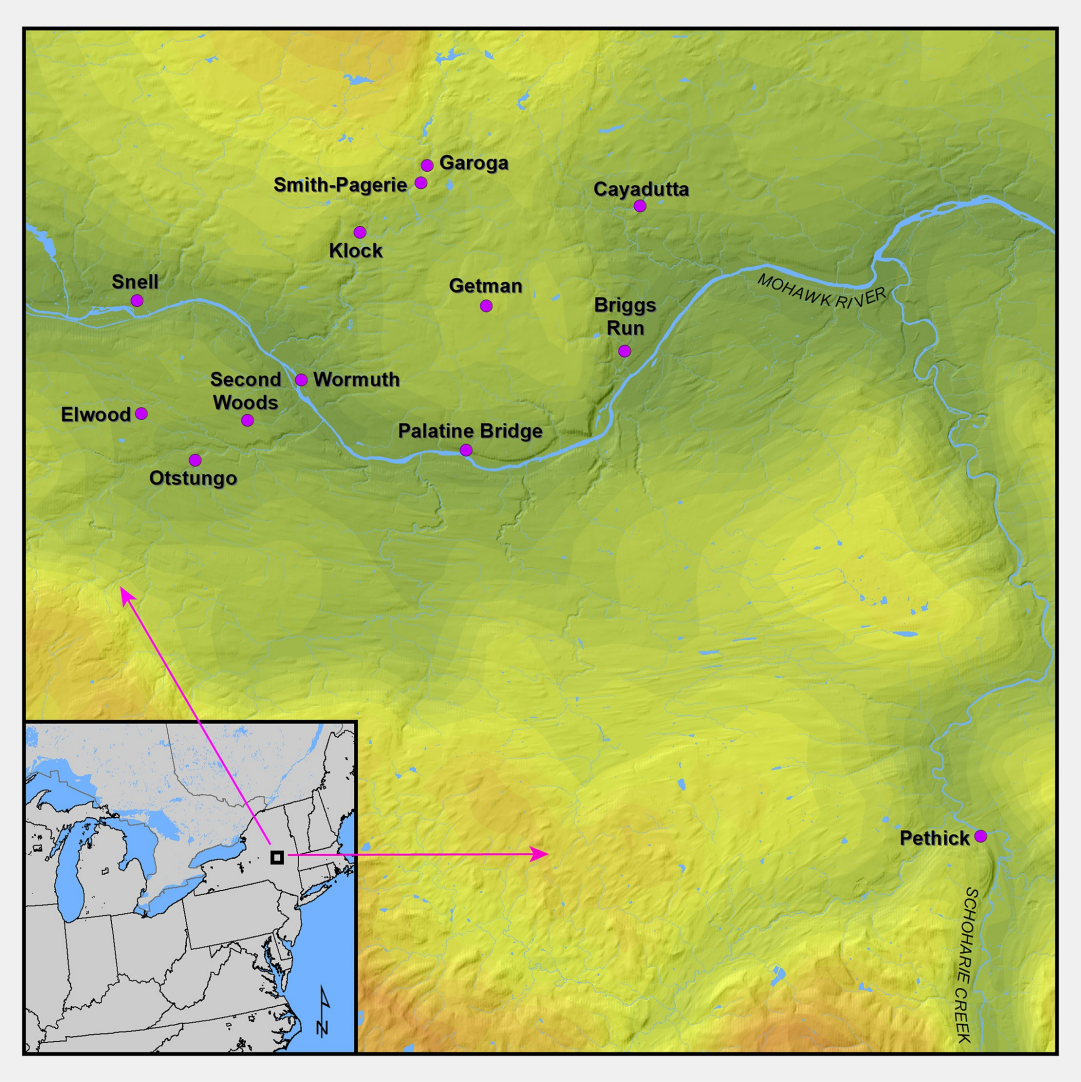
A map showing the Mohawk Valley region in northeast North America and all the sites analyzed in this study. This map was produced in ArcGIS v 10.6 at the New York State Museum in Albany by compiling GIS shapefiles obtained from publicly available sources including Statistics Canada, the United States Census, and the United States Geological Survey.

Our primary focus for this analysis is on four key sites in the Mohawk River Valley sequence chosen for analysis because they have evidence for the early circulation of European metal artifacts and have been traditionally dated to the mid-to-late sixteenth century: Cayadutta, Garoga, Klock, and Smith-Pagerie (Fig 1). European iron artifacts occurred at Cayuga, Klock, and Smith-Pagerie, while potentially smelted cuprous artifacts occurred at each of the four sites. These are all large village sites that were constructed on defensible peninsula-like upland ridges, protected by steep slopes on three sites and a palisade on the fourth. However, to place those sites in chronological sequence, we have also included in our dating program seven sites believed to be earlier and two sites placed later. The former, Snell, Pethick, Second Woods, Elwood, Otstungo and Wormuth are generally dated to the thirteenth through early sixteenth centuries, and the latter, Briggs Run and Palatine Bridge, to the early seventeenth century. We first discuss evidence for European metals at the sites and then the results of the radiocarbon dating and Bayesian analyses.

### Early European metals

European metal artifacts have been reported from five of the sites and one component of a multicomponent site included in our analysis that were dated to the sixteenth-century by Snow [17]. These are Cayadutta, Garoga, Klock, Otstungo, and Smith-Pagerie, and the protohistoric-component from Wormuth. The artifacts from Otstungo and Wormuth were not considered by Snow but are reported by Lenig [30]. Analysis of cuprous artifacts was performed with a Bruker Tracer III-V pXRF. Along with iron artifacts, previous pXRF work [31] has established that cuprous artifacts with two or more trace element peaks derive from European smelted metal (versus indigenous copper). Spectra and Region of Interest (ROI) data are presented in S1 File; a summary of findings are listed in Tables 1 and 2. Review of the findings suggests successful discrimination of the two types of copper (sometimes at the same site) and positive evidence for the presence of European-source metals in several cases.

**Table 1:** Summary of pXRF results for trace elements on six artifacts. For details, see S1 File.

Site	NYSM #	Artifact	As	Pb	Sn	Zn	Interpretation
Cayudutta	n/a	Amulet	+	+	–	–	European copper alloy
Garoga	A-A2009.07BB.99.15	Tube	+	+	+	+	European copper alloy
Garoga	A-42864A	Scrap	+	+	–	–	European copper alloy
Getman #1	A-A2002.47DU.99.10	Bead	–	–	–	–	Native copper
Smith-Pagerie	A-A2002.32AC.7.38	Bead	–	–	–	–	Native copper
Smith-Pagerie	A-44736.1	Bead	+	+	–	–	European copper alloy
Wormuth	A-2002.10CC.07.11	Scrap	+	+	–	+	European copper alloy

**Table 2:** European metals at radiocarbon-dated pre-seventeenth-century Mohawk Valley sites.

Site	Copper Alloy	Identification	Iron	Identification
Snell	–	n/a	–	
Pethick	–	n/a	–	
Second Woods	–	n/a	–	
Getman #1	–	This study	–	
Elwood	–	n/a	–	
Smith-Pagerie	+	This study	+	This study
Otstungo	–	[17, 30]	–	[17, 30]
Klock	+?	[15, 30]	+	[30]
Cayadutta	+	This study	+	[17]
Garoga	+	This study	–	
Wormuth	+	This study	+	This study

A piece of native copper (Cu) from Houghton County, Michigan (NYSM Mineralogy #7005) evinced a trace of iron (Fe), but otherwise no other trace element spectrum peaks. Any presence of lead (Pb), arsenic (As), or zinc (Zn) in this native copper [32] are, as expected [31], below the detection limits of the pXRF instrument. The spectra for rolled copper beads recovered from the Morse (NYSM A20529, 273932A) site in Jefferson County, New York [33] and a crescent-shaped tanged knife blade from the Getman #1 site (NYSM A2 A2002.47DU.99.10) have similar spectra with only an iron peak in addition to copper. The Morse site dates to the late fifteenth-early sixteenth century [34], while Getman #1 dates to the second half of the fifteenth century (see below). Two triangular points and two tinklers (cone-shaped ornaments) from post-contact Briggs Run (NYSM A2005.13.AG.99.10A–D), on the other hand have arsenic and lead peaks along with zinc indicating European copper alloys. Copper points and tinklers are not known in the region until after European contact (see [17, 30]). Briggs Run dates to the first half of the seventeenth century (see below). Two post-contact triangular points from the Mohawk Valley in the Briggs Collection (NYSM 2204, 2205) also evince arsenic and lead peaks but without the presence of zinc. These results indicate that the instrument is capable of differentiating between native copper and European copper and copper alloys.

A trianguloid piece of iron was recovered from the Smith-Pagerie site during New York State Museum (NYSM) excavations in 1970 (NYSM A44765-4) in a secure context at a depth of 40.6 cm below ground surface beneath a piece of fire cracked rock in a charcoal concentration associated with Feature 38, a hearth within Longhouse 1. A rolled copper bead (NYSM A44736.1) was found in 1970 in a secure context at a depth of 17.8 cm in Feature 23, a pit feature within Longhouse 1. PXRF analysis indicates the presence of arsenic (As) and lead (Pb), which indicates European copper alloy. PXRF analysis of a second piece of copper recovered by avocational archaeologist John Swart ([30]; NYSM A-A2002.32AC.7.38) indicates it is of native copper.

According to the excavators no evident early European metal artifacts were recovered from the Klock site during the NYSM excavations in 1969–1970, except a “small scrap of brass” from the plow zone, which is not in the NYSM’s collections ([15], p.44). However, a small iron piece resembling a chain link, was recovered from Feature 147, a hearth, during the NYSM excavations. No notes on the feature’s excavations are present in the NYSM archaeology archives, so the circumstances of the piece’s recovery cannot be assessed. Funk and Kuhn [15] do not mention this piece specifically but assert that all iron artifacts recovered during the NYSM excavations are of nineteenth-century origin. Snow ([17], p.167) mentions a piece of copper and a length of chain reputedly found in a post mold during the NYSM excavations. These are not in the NYSM collections so cannot be confirmed and are not mentioned in [15]. It is possible that the iron piece is the chain segment to which Snow refers. Lenig [29] indicates that a second piece of iron was recovered from a secure context at a depth of 61 cm in a pit feature at Klock by Donald Lenig. He describes it as a badly rusted early axe bit or possibly a section of iron kettle band modified for use as a celt. Its recovery at depth in a large pit feature attests to its association with the Iroquoian occupation of the site.

Snow [17] lists five artifacts in various collections from Garoga of potential European copper alloy. Two of these are in the NYSM collections and were examined with pXRF. A metal bead/tube, originally collected from the site in 1886 by Adelbert G. Richmond (NYSM A-A2009.07BB.99.15), had peaks of zinc, lead, tin, and arsenic indicating a European origin. The second artifact is a small piece of copper (NYSM A-42856.003) recovered from a secure context at the bottom of a post mold during NYSM excavations ([15], p.127). PXRF analysis of this object shows peaks of arsenic and lead, again indicating it is smelted European copper alloy.

Snow [17] reports the recovery of a piece of iron during his excavations at the Cayadutta site, but it is not in the collections transferred to NYSM (accession A-A2005.13CE). Field notes in the NYSM archaeology archives indicate that the artifact was recovered from a seemingly secure context in the midden at a depth of ~30 cm in a unit that had no other non-Native American artifacts. An iron knife blade in the Klinkhart collection from the site is also mentioned ([17], p.189). Lenig [30] notes that a copper-alloy bead was collected from the site in 1892 by Dewitt Devendorf. The whereabouts of this artifact are unknown. An illustration of the artifact in the Rufus Grider scrapbooks appears very similar to the brass tube/bead in the Garoga collection [30]. PXRF analysis of a copper amulet recovered during avocational excavations at Cayadutta in the late 1950s or early 1960s has arsenic and lead peaks indicating European copper alloy.

Lenig [30] reports that an early iron axe was recovered from the flats below the Otstungo site. This axe, now in the NYSM collections weighs 1160g, consistent with a late sixteenth-century origin [34]. Early reports of copper/brass recovered from the site [30] cannot be confirmed. Lenig [30] lists a series of metal objects excavated from the Wormuth site by Jan Swart, including an iron axe now in the NYSM collections (NYSM# A2002.10CC.06.07) that weighs 662g, consistent with an early seventeenth century origin [34]. PXRF analyses of four pieces of copper scrap recovered by Jan Swart (NYSM # A2002.10CC.4.7a) [30] indicate the presence of arsenic, lead, and zinc consistent with European copper alloy. This material may relate to the Peter Wormuth Inn at the site, which dates to ~1750–1830 [30] or the sixteenth-century occupation (see below).

### Radiocarbon dates

The radiocarbon database available for this Mohawk Valley re-assessment of 13 sites, including those dates obtained for the present study, is presented in S2 File with details on sample context, material, non-modeled individual calibrated age ranges [22] and reference source (where the dates are taken from the literature). Samples were selected for the present study from collections curated at the NYSM that originated in secure feature or midden contexts as confirmed by field notes and laboratory records in the NYSM archaeology archive. All UCIAMS dates are published here for the first time, as are one ISGS and one OS date obtained for Palatine Bridge, which were obtained prior to the current project, but not previously published. Samples assayed for this study included 27 pieces of ungulate bone (*Odocoileus virginianus* or cf. *Odocoileus virginianus*) and 34 maize (*Zea mays* ssp. *mays*) kernels and one maize cob. Collagen yield, isotope, and C/N ratio data for bone samples are provided in S2 File. All >30kDa collagen yields were ≥1%, and C/N ratios for these samples fall within the established acceptable range for radiocarbon dating [35, 36].

We employ all radiocarbon data we are aware of from the 13 sites. The radiocarbon dates run many decades ago with large measurement errors become largely irrelevant in the Bayesian analyses (see below), but since their contextual associations appear valid, we include them, rather than adopt an arbitrary chronological cleansing (compare comments in [27], p.193). The Michigan (M) dates are taken from the original publications (S2 File)—we note that there are a few discrepancies compared with mentions in [17]. We employ the three Dicarb (DIC) dates for Wormuth as reported in [30]. We observe that Dicarb Corporation Radioisotope Laboratory dates have been shown to be suspect—typically too recent [37]. However, the DIC dates for Wormuth appear reasonable even if noisy and imprecise and are included in the Bayesian analyses.

### Bayesian chronological modeling

All Bayesian chronological models were run in OxCal 4.3 [23, 38] using what is at the time of writing the current mid-latitude Northern Hemisphere radiocarbon calibration curve, IntCal13 [22]. All modeling steps are presented in the Methods section, and the OxCal run files are presented in S3 File. OxCal command terms like Phase, Boundary, Order, etc. are capitalized in our text. The main aim of the analyses is to resolve plausible site dates and site durations for the set of sites despite the reversal/plateau in the radiocarbon calibration curve ~1500–1600 which has the tendency to spread out dating probability and create ambiguity in the absence of constraints [13, 14]. This problem was highlighted for our project in the initial review of the available dates and our preliminary Model 1 (S4 File). This considered the data from each site as separate Phases in OxCal within an over-arching Phase, thus allowing each site Phase to date independently of the others, and with no constraints on site Phase duration. Model 1 demonstrated the problem of ambiguity and smearing of dating probability creating apparent unrealistically over-long site duration estimates for several of the site Phases in the absence of additional constraints (see Methods): Table 3. All indications from ethnography, archaeology and radiocarbon suggest that sites in this region typically had relatively short total occupation durations [5, 11, 13–15, 17, 34, 39, 40], of a couple to a few decades (e.g. 0–40 years at most) and likely no more than 50–75 years and probably less.

**Table 3:** Site Phase durations from OxCal Interval queries for six example runs of Model 1 with no Interval constraint applied to the site Phases and employing all the data from the 11 sites.

	Interval Query results from six runs of Model 1–68.2% hpd and 95.4% hpd ranges in calendar years											
	*Am46*.*4*	*Ao46*.*9*	*Am47*	*Ao48*.*8*	*Am48*.*5*	*Ao41*.*2*	*Am50*.*2*	*Ao50*.*9*	*Am53*.*1*	*Ao40*.*2*	*Am55*.*5*	*Ao53*.*1*
**Site Phase**	*68*.*2%*	*95*.*4%*	*68*.*2%*	*95*.*4%*	*68*.*2%*	*95*.*4%*	*68*.*2%*	*95*.*4%*	*68*.*2%*	*95*.*4%*	*68*.*2%*	*95*.*4%*
**Snell**	0–16	0–42	0–17	0–45	0–17	0–46	0–15	0–36	0–14	0–37	**0–14**	**0–38**
**Pethick**	0–60	0–236	0–44	0–212	0–147	0–312	0–113	0–282	1–118	0–268	**0–109**	**0–265**
**Second Woods**	0–58	0–215	0–62	0–226	0–90	0–279	0–90	0–280	0–59	0–220	**0–88**	**0–276**
**Elwood**	1 to 82	0–193	1 to 81	0–192	1 to 82	0–192	0–80	0–191	0–81	0–192	**0–80**	**0–191**
**Getman**	56–118	2–202	60–130	42–216	57–119	4–203	56–121	1–206	57–119	5–205	**56–119**	**20–204**
**Smith-Pagerie**	99–204	69–259	0–205	0–246	1–208	1–262	100–205	1–255	97–205	2–258	**97–203**	**75–256**
**Otstungo**	77–175	25–204	0–174	0–200	0–162	0–193	74–175	19–202	0–159	0–194	**74–175**	**20–203**
**Klock**	0–66	0–185	0–70	0–184	0–67	0–186	0–68	0–183	0–71	0–188	**0–68**	**0–187**
**Cayadutta**	0–253	0–337	102–258	4–340	107–260	3–341	1–257	0–330	112–257	2–336	**112–258**	**0–335**
**Garoga**	1–317	0–417	0–298	0–400	164–323	1–410	0–260	0–429	177–306	121–485	**178–306**	**110–485**
**Wormuth**	1–146	0–272	0–141	0–260	0–144	0–275	0–141	0–264	1–146	0–277	**0–148**	**0–281**

Intervals are in (calibrated) calendar years. Results for six pairings of 68.2% highest posterior density (hpd) and 95.4% hpd ranges. Data from OxCal [23, 38] with IntCal13 [22] with curve resolution set at 1 year. The bold font (far right) run results are from the model returning the best OxCal indices A_model_ (Am) and A_overall_ (Ao) values.

**Fig 2:**
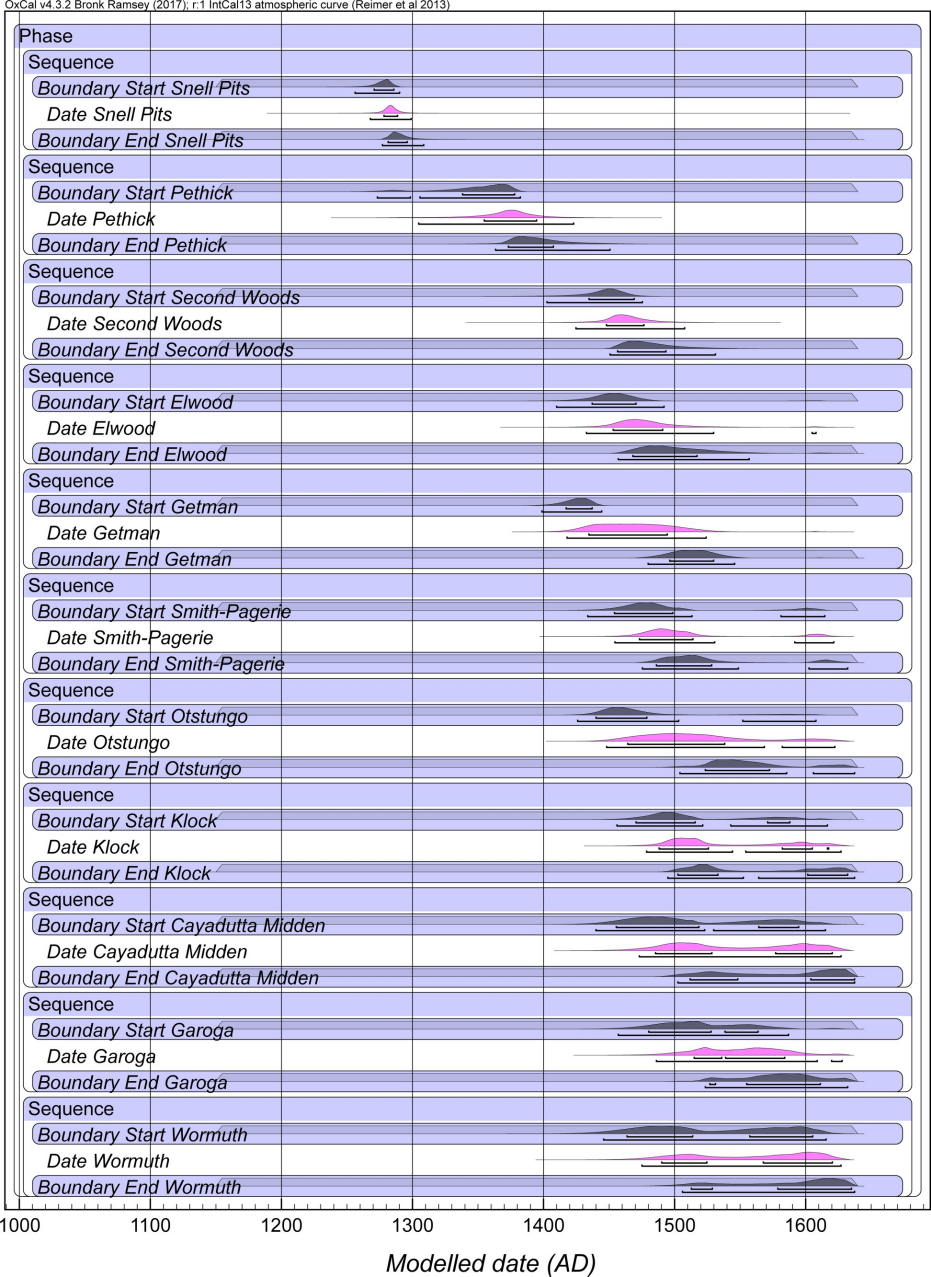
Model 2 selected elements with site Phase Intervals constrained (uniform probability) to 0–120 years. Data from OxCal [23, 38] and IntCal13 [22] with curve resolution set at 1 year. For full Model 2, see S5 File. Start and End Boundaries in Black, Date estimates for each site Phase in magenta. Lines under the histograms indicate the 68.2% and 95.4% hpd ranges.

We therefore considered three versions of a Model 2 with 0–80 years, 0–100 years and 0–120 years uniform probability Interval constraints for the site Phases for 11 of the Mohawk Valley sites (excluding Briggs Run and Palatine Bridge—see Methods) in order to investigate and determine the likely Order of these sites (using the OxCal Order query), see Fig 2, S5 File, and S6 File (and see Methods). The OxCal Order function quantifies the probability of the temporal order among the dated elements in the model. The models ran well with good typical OxCal A_model_ and A_overall_ values greater than the approximate acceptable value of 60 (see Fig 2, S5 File and S6 File). S6 File shows the probabilities comparing the start Boundary, the end Boundary and the OxCal Date estimate for each site Phase for the runs of Model 2. The OxCal Date estimate function determines a hypothetical event which describes the full temporal extent of the host Phase. If the start and end Boundaries were known exactly, then this Date estimate would cover the length of the Phase, but, in practice, the start and end Boundaries are known only within uncertainties, hence the Date estimate is a combination of the Phase period and these start/end uncertainties. Nonetheless, while not a strict proxy for the extent (length) of the Phase, overall, the Date estimate provides a good summary of the Phase. The Order of the site Phase OxCal Date estimates across the different versions of Model 2 is shown in Table 4. There are some small variations, but the results are very consistent with the order of (oldest to most recent):

1. Snell, 2. Pethick, 3. Second Woods, 4. Getman, 5. Elwood, 6. Smith-Pagerie, 7. Otstungo, 8. Klock, 9. Cayadutta, 10. Garoga, 11. Wormuth.

The order between Cayadutta and Garoga is not very clear, as noted in the Methods section below. Cayadutta seems slightly older overall, but we also consider a model where no order is assumed between Cayadutta and Garoga (Model 3A). While there seems a relatively clear order of the Date estimates for the sites (Table 4), a feature of the Order analysis, with the exception of Snell and then Pethick, is that it suggests that each of the other sites exhibit some degree of overlap with the following/preceding site(s). Some sites may also have longer durations than others (partly creating such overlaps in addition). Thus, there is an order or chronological trend in the site dates, but with overlaps in most cases. This is not surprising. The expectation is 11 sites (after Pethick) fitting into a period of around 150–175 or so years from the mid-fifteenth century to early seventeenth century, thus overlaps between sites, or parallel sites (especially between sites from the different eastern versus western parts of the Mohawk Valley), are entirely to be anticipated. Although two community site sequences are conjectured [17], there is no independent basis to the dates or order of the sites in our set and overlaps or parallel occupations are in fact also assumed [17].

**Table 4:** Order analyses for the OxCal Date estimates for each site Phase extracted from S5 File from the Model 2 versions with 0–120 years, 0–100 years and 0–80 years uniform probability Interval query constraints on the duration of each individual site Phase.

**Order, Model 2, 0–120 years Interval Constraint**											
**Probability *t***_**1**_ **< *t***_**2**_											
***t***_**1**_	*** ***										
	**Date Snell Pits**	**Date Pethick**	**Date Second Woods**	**Date Getman**	**Date Elwood**	**Date Smith-Pagerie**	**Date Otstungo**	**Date Klock**	**Date Cayadutta Midden**	**Date Garoga**	**Date Wormuth**
**Date Snell**	0.00	***0*.*99***	***1*.*00***	***1*.*00***	***1*.*00***	***1*.*00***	***1*.*00***	***1*.*00***	***1*.*00***	***1*.*00***	***1*.*00***
**Date Pethick**	0.01	0.00	***1*.*00***	***1*.*00***	***1*.*00***	***1*.*00***	***1*.*00***	***1*.*00***	***1*.*00***	***1*.*00***	***1*.*00***
**Date Second Woods**	0.00	0.00	0.00	***0*.*55***	***0*.*69***	***0*.*91***	***0*.*90***	***0*.*97***	***0*.*96***	***0*.*99***	***0*.*97***
**Date Getman**	0.00	0.00	0.45	0.00	***0*.*59***	***0*.*80***	***0*.*83***	***0*.*93***	***0*.*92***	***0*.*97***	***0*.*94***
**Date Elwood**	0.00	0.00	0.31	0.41	0.00	***0*.*79***	***0*.*80***	***0*.*92***	***0*.*91***	***0*.*95***	***0*.*92***
**Date Smith-Pagerie**	0.00	0.00	0.09	0.20	0.21	0.00	***0*.*57***	***0*.*73***	***0*.*74***	***0*.*81***	***0*.*77***
**Date Otstungo**	0.00	0.00	0.10	0.17	0.20	0.43	0.00	***0*.*64***	***0*.*68***	***0*.*75***	***0*.*72***
**Date Klock**	0.00	0.00	0.03	0.07	0.08	0.27	0.36	0.00	***0*.*55***	***0*.*61***	***0*.*60***
**Date Cayadutta**	0.00	0.00	0.04	0.08	0.09	0.26	0.32	0.45	0.00	***0*.*52***	***0*.*55***
**Date Garoga**	0.00	0.00	0.01	0.03	0.05	0.19	0.25	0.39	0.48	0.00	***0*.*55***
**Date Wormuth**	0.00	0.00	0.03	0.06	0.08	0.23	0.28	0.40	0.45	0.45	0.00
**Order, Model 2, 0–100 years Interval Constraint re-run excluding also UCIAMS192976**											
**Probability *t***_**1**_ **< *t***_**2**_											
***t***_**1**_	*** ***										
	**Date Snell Pits**	**Date Pethick**	**Date Second Woods**	**Date Getman**	**Date Elwood**	**Date Smith-Pagerie**	**Date Otstungo**	**Date Klock**	**Date Cayadutta Midden**	**Date Garoga**	**Date Wormuth**
**Date Snell**	0.00	***1*.*00***	***1*.*00***	***1*.*00***	***1*.*00***	***1*.*00***	***1*.*00***	***1*.*00***	***1*.*00***	***1*.*00***	***1*.*00***
**Date Pethick**	0.00	0.00	***1*.*00***	***1*.*00***	***1*.*00***	***1*.*00***	***1*.*00***	***1*.*00***	***1*.*00***	***1*.*00***	***1*.*00***
**Date Second Woods**	0.00	0.00	0.00	***0*.*63***	***0*.*68***	***0*.*91***	***0*.*91***	***0*.*98***	***0*.*97***	***0*.*99***	***0*.*97***
**Date Getman**	0.00	0.00	0.37	0.00	***0*.*52***	***0*.*79***	***0*.*83***	***0*.*93***	***0*.*93***	***0*.*98***	***0*.*94***
**Date Elwood**	0.00	0.00	0.32	0.48	0.00	***0*.*80***	***0*.*82***	***0*.*93***	***0*.*92***	***0*.*96***	***0*.*93***
**Date Smith-Pagerie**	0.00	0.00	0.09	0.21	0.20	0.00	***0*.*58***	***0*.*73***	***0*.*74***	***0*.*81***	***0*.*77***
**Date Otstungo**	0.00	0.00	0.09	0.17	0.18	0.42	0.00	***0*.*62***	***0*.*67***	***0*.*74***	***0*.*72***
**Date Klock**	0.00	0.00	0.02	0.07	0.07	0.27	0.38	0.00	***0*.*57***	***0*.*62***	***0*.*62***
**Date Cayadutta**	0.00	0.00	0.03	0.07	0.08	0.26	0.33	0.43	0.00	***0*.*51***	***0*.*55***
**Date Garoga**	0.00	0.00	0.01	0.02	0.04	0.19	0.26	0.38	0.49	0.00	***0*.*56***
**Date Wormuth**	0.00	0.00	0.03	0.06	0.07	0.23	0.28	0.38	0.45	0.44	0.00
**Order, Model 2, 0–80 years Interval Constraint re-run excluding also UCIAMS192976**											
**Probability *t***_**1**_ **< *t***_**2**_											
***t***_**1**_	*** ***										
	**Date Snell Pits**	**Date Pethick**	**Date Second Woods**	**Date Getman**	**Date Elwood**	**Date Smith-Pagerie**	**Date Otstungo**	**Date Klock**	**Date Cayadutta Midden**	**Date Garoga**	**Date Wormuth**
**Date Snell**	0.00	***1*.*00***	***1*.*00***	***1*.*00***	***1*.*00***	***1*.*00***	***1*.*00***	***1*.*00***	***1*.*00***	***1*.*00***	***1*.*00***
**Date Pethick**	0.00	0.00	***1*.*00***	***1*.*00***	***1*.*00***	***1*.*00***	***1*.*00***	***1*.*00***	***1*.*00***	***1*.*00***	***1*.*00***
**Date Second Woods**	0.00	0.00	0.00	***0*.*63***	***0*.*69***	***0*.*92***	***0*.*94***	***0*.*98***	***0*.*98***	***1*.*00***	***0*.*98***
**Date Getman**	0.00	0.00	0.37	0.00	***0*.*54***	***0*.*83***	***0*.*89***	***0*.*96***	***0*.*95***	***0*.*99***	***0*.*96***
**Date Elwood**	0.00	0.00	0.31	0.46	0.00	***0*.*82***	***0*.*87***	***0*.*94***	***0*.*94***	***0*.*97***	***0*.*95***
**Date Smith-Pagerie**	0.00	0.00	0.08	0.17	0.18	0.00	***0*.*63***	***0*.*74***	***0*.*75***	***0*.*82***	***0*.*79***
**Date Otstungo**	0.00	0.00	0.06	0.11	0.13	0.37	0.00	***0*.*57***	***0*.*63***	***0*.*67***	***0*.*68***
**Date Klock**	0.00	0.00	0.02	0.04	0.06	0.26	0.43	0.00	***0*.*57***	***0*.*62***	***0*.*63***
**Date Cayadutta**	0.00	0.00	0.02	0.05	0.06	0.25	0.38	0.43	0.00	0.50	***0*.*56***
**Date Garoga**	0.00	0.00	0.00	0.01	0.03	0.18	0.33	0.38	***0*.*50***	0.00	***0*.*60***
**Date Wormuth**	0.00	0.00	0.02	0.04	0.05	0.21	0.32	0.37	0.44	0.40	0.00

The probabilities shown are for t_1_ as less than, that is older than, t_2_. Data from OxCal [23, 38] and IntCal13 [22] with curve resolution set at 1 year. See comments in S1 Appendix. Probabilities >0.5 are in bold italic font.

We separately, and then subsequently, considered the Order of the two late sites, Briggs Run and Palatine Bridge versus each other, and against the other sites (see Methods). This found that they both clearly date after all the other sites in our analysis and so offer a constraint for the recent end of our sequence. Given one set of assumptions, Briggs Run dates slightly earlier than Palatine Bridge, and given another set of assumptions, the reverse occurs. We thus considered the Briggs Run then Palatine Bridge order in Model 3 and a coeval order scenario in Model 3A (S7 File, S8 File). We also considered Models 4 and 4A without these two late sites to compare the effect of their inclusion/non-inclusion (S9 File).

**Fig 3:**
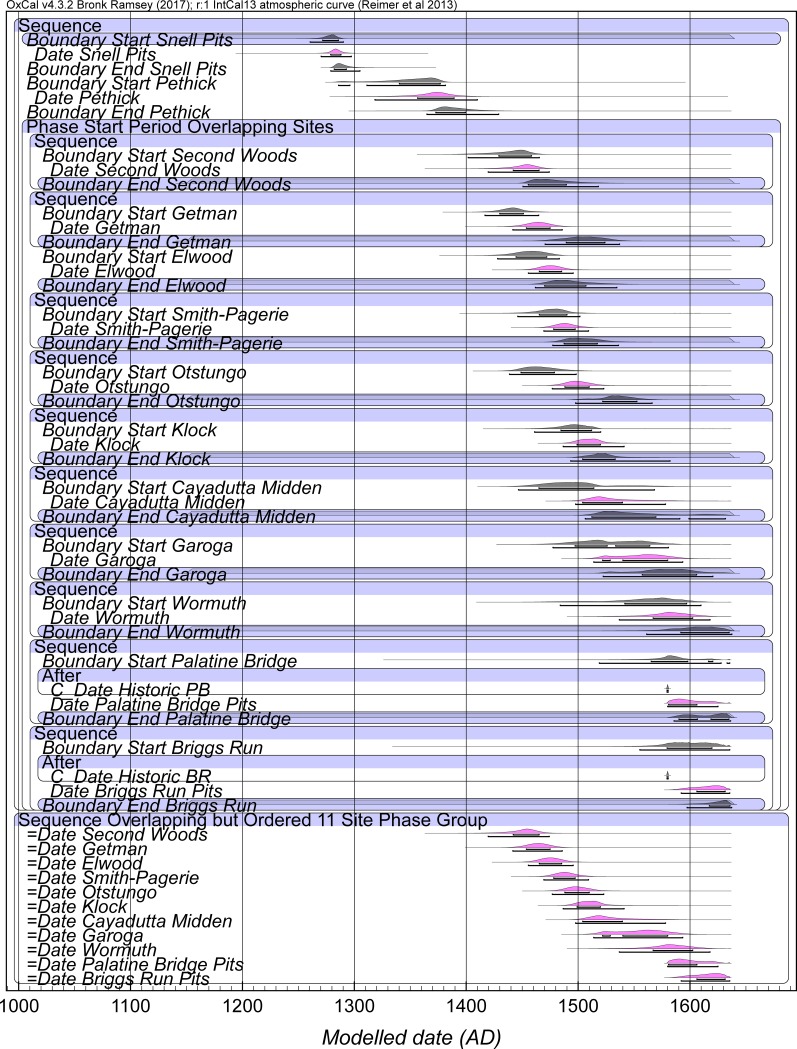
Model 3 selected elements with site Phase Intervals constrained (uniform probability) to 0–100 years. Data from OxCal [23, 38] and IntCal13 [22] with curve resolution set at 1 year. Example shown with OxCal A_model_ = 82.1 and A_overall_ = 69.1. For full Model 3, see S7 File. Start and End Boundaries in Black, Date estimates for each site Phase in magenta. Lines under the histograms indicate the 68.2% and 95.4% hpd ranges. The separate but cross-referenced Sequence of the Date estimates is shown at the bottom of the main model (see Methods). Historic BR and Historic PB refer to the TPQ of 1580 applied to Briggs Run and Palatine Bridge (see Methods).

**Fig 4:**
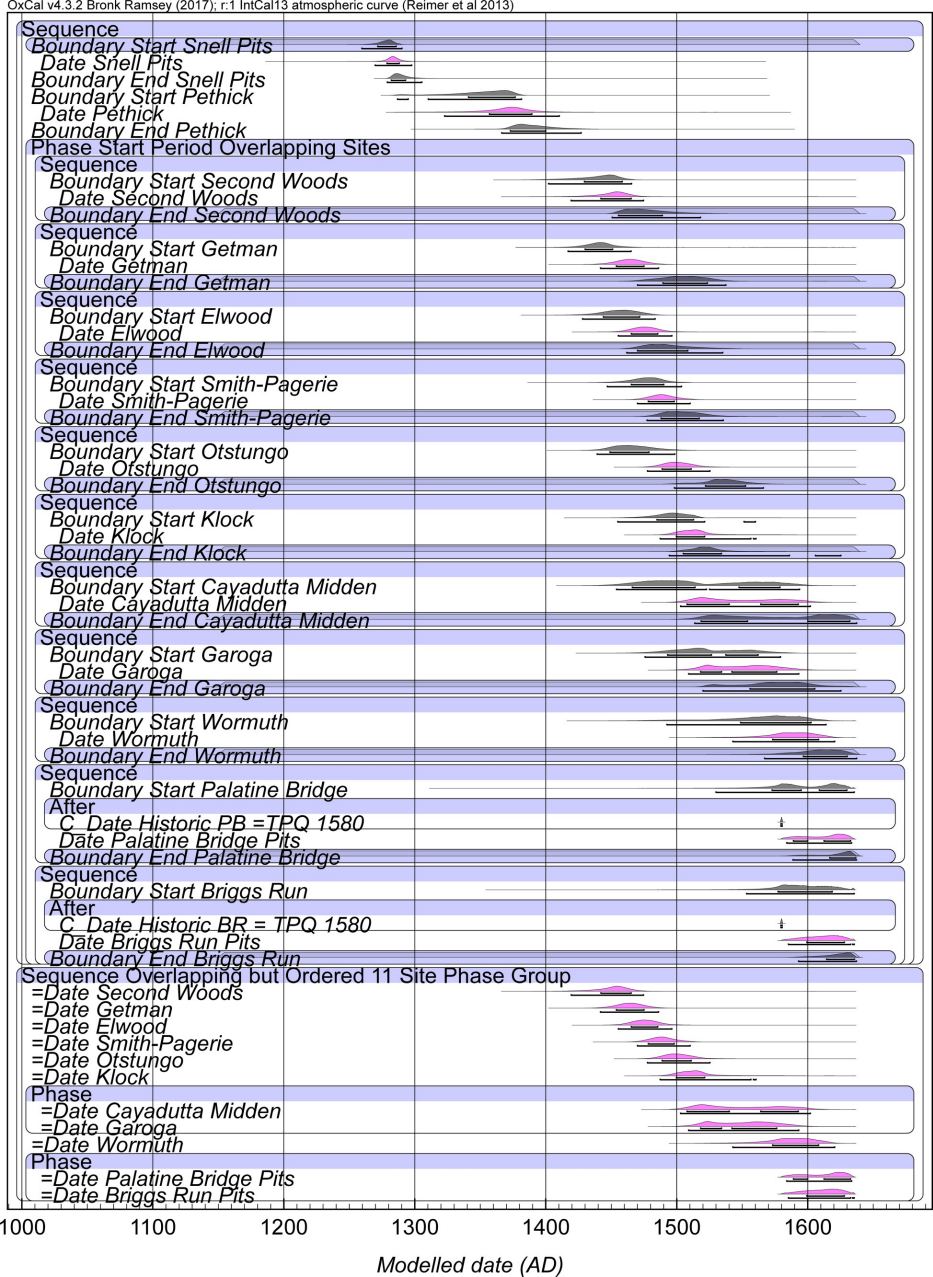
Model 3A selected elements with site Phase Intervals constrained (uniform probability) to 0–100 years. Data from OxCal [23, 38] and IntCal13 [22] with curve resolution set at 1 year. Example shown with OxCal A_model_ = 80.9 and A_overall_ = 70. For full Model 3, see S7 File. Start and End Boundaries in Black, Date estimates for each site Phase in magenta. Lines under the histograms indicate the 68.2% and 95.4% hpd ranges. The separate but cross-referenced Sequence of the Date estimates is shown at the bottom of the main model (see Methods)—note (i) Cayadutta + Garoga and (ii) Palatine Bridge + Briggs Run are considered as potentially coeval (and floating against each other). As in Fig 3, Historic BR and Historic PB refer to the TPQ of 1580 applied to Briggs Run and Palatine Bridge (as noted in this figure).

**Fig 5:**
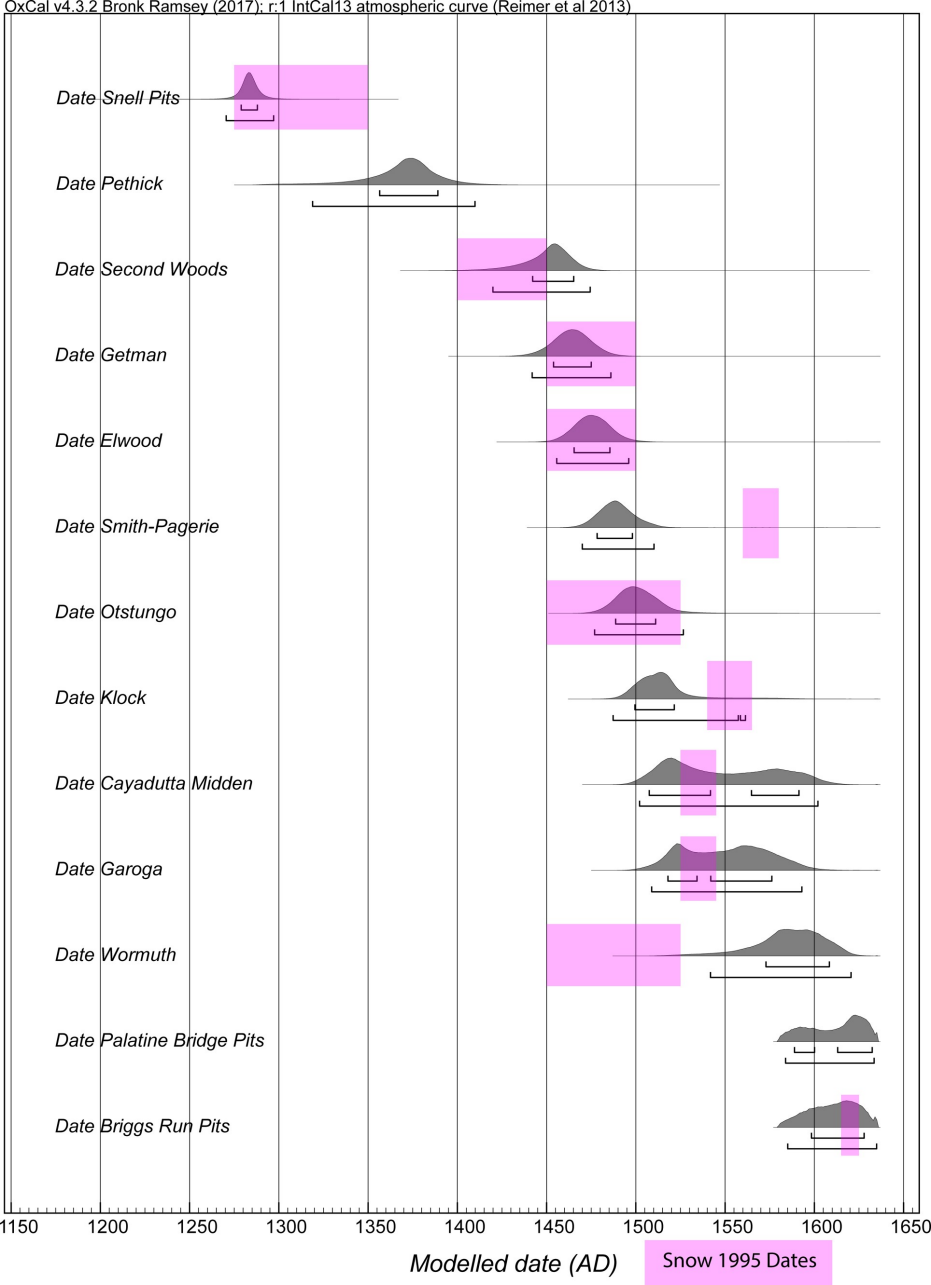
A comparison of the modelled date ranges for selected elements of a run of Model 3A versus the calendar dates estimated in Snow [17]. This example of Model 3A ran with A_model_ = 76.4 and A_overall_ = 68.6 (compare with and very similar to but very slightly different to Fig 4, Table 5 as an illustration of small variations between satisfactory runs). See also Table 5.

Models 3 and 3A provide our best calendar date estimates for the set of sites, see Figs 3, 4, and 5 and Table 5, S7 File, S8 File. We note that both Model 3 and 3A tend not to get good Convergence (C) values for elements of Briggs Run and often Palatine Bridge (i.e. they are less than the satisfactory level of ~95). It is clearly a challenge to squeeze the respective date information into a short period of time. We compare our findings with the dates proposed for a number of the sites in the study of Snow [17] in Table 5 and Fig 5. The sixteenth century region in particular is conspicuous for differences, highlighting the role assumptions about the distribution and presence of trade goods have played in building the existing timescale in this proto-historic period. This situation emphasizes the need now to investigate timescales independent of such assumptions and unjustified step-wise logic transfers. Compared to expectations, Smith-Pagerie moves substantially earlier, Klock is somewhat earlier, Garoga likely is a little later, and Wormuth substantially later on the basis of the available radiocarbon dates and analysis in Models 3 and 3A (and even more so if we do not add the late-end constraints of Briggs Run and Palatine Bridge: S9 File). The main difference between Models 3 and 3A concerns the placements of Cayadutta and Garoga (see Table 5). Model 3 prescribed an order (Cayadutta older than Garoga), whereas Model 3A allowed the two sites to float relative to each other. In Model 3 Cayadutta is 1504–1539 (68.2% hpd) and Garoga is primarily 1540–1580 (60.0% range of the 68.2% hpd). In Model 3A the majority of the probability favors the same scenario and thus order, Cayadutta 1507–1540 with 41.4% of the 68.2% hpd and Garoga 1542–1576 with 45.8% of the 68.2% hpd, but there is also a 26.8% more recent sub-range 1564–1592 for Cayadutta and a 22.4% older sub-range 1517–1534 for Garoga. This situation illustrates possible, but less likely, alternative site histories. The respective 95.4% hpd ranges for Cayadutta and Garoga from Models 3 and 3A cover more or less the entire sixteenth century. The sixteenth century should thus be a focus for additional future chronological investigation to establish greater temporal resolution.

**Table 5:** Selected results from runs of Model 3 and Model 3A (see S7 File, S8 File) and compared to the date estimates of Snow [17].

Site (Date Estimate)	Model 3 Modelled 68.2%	Model 3 Modelled 95.4%	Model 3A Modelled 68.2%	Model 3A Modelled 95.4%	Snow [17] Date	Snow [17] reference
***Snell***	1278–1288	1270–1297	1278–1288	1269–1297	1275–1350	p. 55
***Pethick***	1356–1389	1318–1409	1357–1389	1322–1410	N/A	N/A
***Second Woods***	1442–1465	1419–1474	1442–1465	1419–1474	1400–1450	p. 97
***Getman***	1453–1475	1441–1485	1453–1475	1441–1486	1450–1500	p. 89
***Elwood***	1465–1485	1455–1495	1465–1485	1455–1496	1450–1500	p. 89
***Smith-Pagerie***	1478–1497	1469–1509	1478–1498	1470–1510	1560–1580	p. 29
***Otstungo***	1488–1509	1477–1522	1488–1511	1477–1525	1450–1525	p. 89
***Klock***	1499–1519	1446–1568	1499–1521	1487–1556 (95.1%) 1558–1560 (0.3%)	1540–1565	p. 29
***Cayadutta***	1504–1539	1497–1578	1507–1540 (40.9%) 1564–1592 (27.3%)	1502–1602	1525–1545	p.29
***Garoga***	1521–1528 (8.2%) 1540–1580 (60.0%)	1514–1593	1518–1534 (22.1%) 1542–1576 (46.1%)	1509–1593	1525–1545	p. 29
***Wormuth***	1567–1602	1536–1617	1573–1608	1542–1620	1450–1525	p. 89
***Briggs Run***	1606–1631	1592–1635	1599–1628	1585–1632 (94.3%) 1634–1625 (1.1%)	1614–1626	p. 29
***Palatine Bridge***	1580–1606	1580–1625	1589–1600 (17.8%) 1612–1632 50.4%)	1584–1633	N/A	N/A

Where Snow states a revised age or similar we cite this (e.g. Snow [17], p.29 cites revised ages for Wormuth, Garoga, Klock, Smith-Pagerie, Cayadutta, Otstungo and Elwood, Snow [17], p.55 cites a revised age for Snell, and Snow [17], p.89 cites revised ages for Wormuth, Otstungo, Elwood and Getman). See also Fig 5. Note there are small differences in results between different model runs, typically of just a few (e.g. 0–2) years.

The results of the analyses in Models 2 and 3 offer some surprises. In particular, the date of Wormuth is not as would have been predicted. Snow ([17], p.142) placed this site 1450–1525 and earlier than Cayadutta, Garoga, Klock and Smith-Pagerie. We instead find a likely date range of 1567–1602 (68.2%, hpd, Model 3) or 1573–1608 (68.2%, hpd, Model 3A) or, without the constraints of Briggs Run and Palatine Bridge (Models 4 and 4A), even later: 1586–1621 (68.2% hpd, Model 4) or 1591–1622 (68.2% hpd, Model 4A) (S9 File). But it is relevant to observe that the existing assessment had issues. Snow ([17], p.139) does mention what he considers a late third minor occupation around the end of the sixteenth century, which includes a reported rolled copper spiral and a Cromwell Incised sherd ([17], pp.141–142) citing Lenig (pers. comm.). Lenig [30], citing notes by H.A. Glosser, mentions surface finds including “a copper? Serpent shaped coil broach, a few copper or brass kettle scraps” and observes that Snow mentioned his “contention that there is a protohistoric component … although he believes the evidence for this later occupation is scanty and somewhat equivocal”. However, Lenig [30] then goes on to argue that there were several late, i.e. protohistoric, finds including: “several incised ceramic rim sherds with luted human face effigies, a full-figure human effigy sherd and early iron axe from the Swart collection, the ‘carved bone comb’, brass scraps and copper spiral described by Glosser”. The weight of the axe, 662g, is consistent with an early seventeenth-century occupation ([34], p.443). Lenig’s assessment, while agreeing there was also likely an earlier occupation in the area, places a major component of the Wormuth site and likely “the vast majority–if not all–of the burials” in the later protohistoric period [30]. Thus, our Order analysis finding that Wormuth appears to date late in the site set (before only Briggs Run and Palatine Bridge in Model 2) and into the protohistoric period is in fact not really unexpected.

Perhaps the most unanticipated result of the present study is the placement of the Smith-Pagerie site in the sequence. Smith-Pagerie is one of three large village sites along with Garoga and Klock located on Caroga Creek that were extensively excavated by NYSM crews in the 1960s and in 1970 [15, 41]. The three sites have been considered to be the sequence of a single community over several generations during the sixteenth century. While the sixteenth century occupation date estimates vary, the generally accepted sequence of the three sites is Garoga, Klock, and Smith [15, 17, 29]. D. Lenig [18] reverses the order of Garoga and Klock in the sequence, and W. Lenig [30] reverses the order of Klock and Smith. Although Snow [17] based his occupational estimates in part on calibrated radiocarbon dates, the generally agreed upon sequence is based primarily on interpretations of seriations of pottery types [29] or attributes [15]. The relative order of these sites in the present analysis is Smith, Klock, Garoga (Table 4, S6 File). Not only is Smith-Pagerie placed earliest of the three, but the 68.2% hpd range in Models 3 and 3A is 1478–1497/98, while the 95.4% hpd range is 1469–1509/10. The relative order matrix indicates that there is an 81–82% probability that Garoga’s occupation (site Phase Date estimate) and a 73–74.0% probability that Klock’s occupation (site Phase Date estimate) occurred later in time than Smith-Pagerie’s occupation period (site Phase Date estimate) (Table 4).

The recovery of a piece of iron and rolled copper bead of European copper from the Smith-Pagerie site [15, 30], as confirmed by pXRF analysis, could argue for its chronological placement after 1525. This placement is also suggested by the presence of human face effigies below the castellations on two rim collars, which Funk and Kuhn [15] argue are not present on fifteenth-century sites. Funk and Kuhn ([15], p.81) dismissed the (then) two radiocarbon dates [17] as “aberrant” because: “there is currently no evidence that the site is multicomponent, and all aspects of the artifact assemblage are characteristic of the mid-sixteenth century.” Following this line of argument for the current results would require one to conclude that each of the (now) 9 radiocarbon dates used in the models is aberrant. This is unlikely given that these dates were obtained from different laboratories on differing materials that were recovered from secure contexts in six different pit features directly associated with longhouses. Rather, it is more likely that the traditional estimated occupational history of the site is incorrect, as are, perhaps, chronological assumptions concerning the circulation of European metals and face effigies on pottery. Rather than a TPQ for assessing the age of the site, it is apparent that the European metal artifacts circulated to the site during its occupation, which began in the fifteenth century. Of note is that Smith-Pagerie is the largest of the three Caroga Creek villages. The hypothesis that its size reflects community population growth through the sequence of the three sites [15, 17] can also likely be rejected.

Given our available evidence, the dating of the end of the Sequence is less than entirely satisfactory, while nonetheless clearly about correct. We outline the inherent ambiguity involved in radiocarbon dating an early seventeenth century site in the Methods section below. A TPQ that removes much of the otherwise possible date range from the late fifteenth through sixteenth century is necessary in order to achieve a refined date (such as dates on tree-rings from wood-charcoal that are older than the short-lived material which can help resolve such an ambiguity, as in the case of the Warminster site [13]). We also have just five dates, three not recently processed, from Briggs Run and Palatine Bridge altogether, and we have had to rely on an approximation versus a real TPQ. The most likely (68.2% hpd) dates of 1606–1631 (Model 3) and 1599–1628 (Model 3A) for Briggs Run and 1580–1606 (Model 3) and 1588–1600 (19.5%) and 1613–1632 (48.7%) (Model 3A) for Palatine Bridge nonetheless cover the anticipated date range (Table 5). These two sites, which the Order analysis suggest are both later than Wormuth, also help limit how late the Wormuth site is placed, since it would otherwise include an earlier seventeenth century age range if there is no constraint in play (compare the Wormuth date placements in Models 3 and 3A in Table 5 with those from Models 4 and 4A in S9 File).

We highlight and consider a final issue: the robustness of our model findings. The dates for the Snell and Pethick sites should be stable within very small margins regardless of model. These Phases do not overlap with the Phase of another site and are well defined on the calibration curve. However, the date ranges for the later sites, from Second Woods onwards, are more complicated as they potentially overlap and enter the plateau/reversal region of the calibration curve. As in most complicated model runs in OxCal, there is an expected small amount of variation in results obtained from different model runs. Moreover, models do not always run successfully. If we consider Model 3, then of 10 runs two had a majority of Convergence (C) values less than the acceptable value of 95 (many well below). The reason is a sustained attempt by the model in these two cases to find some possible region for all the site Phases from Pethick onwards in the early seventeenth century. Thus, for example, considering the 95.4% hpd ranges, Pethick 1314–1413 (86.3%), 1591–1606 (9.1%), Second Woods 1417–1475 (85.7%), 1600–1609 (9.7%), Getman 1441–1486 (85.6%), 1603–1610 (9.8%), etc. While this is semi-possible mathematically, it is completely impossible in real-world terms, and the consistently poor C values in the OxCal analysis indicate the problem. This seventeenth-century range is excluded in the successful runs (8 of 10 in the above set of runs) with the vast majority of C values ≥95. In the 8 successful runs there were just a handful of C values <95 and these were always for elements of the Palatine Bridge and Briggs Run sites. This indicates, as noted above, that these two sites were the least satisfactorily dated part of the model with a few loosely defined dates being crammed into a very short temporal interval. For the 8 successful runs the average OxCal A_model_ value is 77±2.3 and the average A_overall_ value is 69±0.5. Table 6 compares the 8 successful runs of Model 3. We see that the start and end ranges of the 68.2% and 95.4% hpd ranges are well defined with only 0–3 years variation across the runs with the sole exception of one sub-range for Palatine Bridge which only occurs in 2 of the 8 models. We observe a similar situation for Model 3A. In 12 runs 3 instances did not complete with satisfactory A_model_ and A_overall_ values and in these 3 cases the vast majority of C values were also <95. In these instances, the model (as above) tried to include an earlier seventeenth-century dating range for most sites. In the 9 successful runs the average A_model_ value was 78.1±1.7 and the average A_overall_ value 70.1±0.2. Even in the successful models, some C values for Briggs Run and Palatine Bridge regularly failed to reach 95 (see comments above). Table 7 compares the 9 successful runs of Model 3A. We see that the start and end ranges of the 68.2% and 95.4% hpd ranges are well defined with only 0–3 years variation across the runs. Overall, for the model runs with good A_model_ and A_overall_ values and with the vast majority of C values ≥95, we find that we have robust findings (despite the small amount of noise in the system).

**Table 6:** Start and End values for the 68.2% hpd and 95.4% hpd ranges for Second Woods to Briggs Run from 8 runs of Model 3 with A_model_ and A_overall_ values above 60 and the vast majority of C values ≥95.

Date Estimate	68.2% Start	STDEV	68.2% End	STDEV	95.4% Start	STDEV	95.4% End	STDEV
**Second Woods**	1441.9	0.4	1465.0	0.0	1419.1	0.4	1474.0	0.0
**Getman**	1453.0	0.0	1474.3	0.5	1441.0	0.0	1485.0	0.0
**Elwood**	1465.0	0.0	1485.0	0.0	1455.0	0.0	1495.1	0.4
**Smith-Pagerie**	1478.0	0.0	1497.0	0.0	1469.0	0.0	1508.9	0.4
**Otstungo**	1488.0	0.0	1509.1	0.4	1477.0	0.0	1522.0	0.5
**Klock**	1499.0	0.0	1519.1	0.4	1486.0	0.5	1541.4	2.6
**Cayadutta**	1503.8	0.5	1538.9	0.8	1497.1	0.4	1577.9	1.1
**Garoga**	1521.4	0.5	1528.4	0.5	1513.6	0.5	1593.5	0.8
** **	1539.4	0.5	1579.9	1.0				
**Wormuth**	1566.8	0.5	1602.5	1.1	1536.4	0.7	1618.3	1.6
**Palatine Bridge**	1580.1	0.4	1605.9	0.6	1580.0	0.0	1624.4	1.1
** **	1626.0	N/A	1628.0	N/A	1629.0	5.7	1631.5	3.5
** **	1630.0	N/A	1633.0	N/A	1630.0	N/A	1634.0	N/A
**Briggs Run**	1606.8	1.4	1631.5	1.1	1591.8	0.7	1634.5	0.8
** **					1634.0	N/A	1635.0	N/A

The standard deviations on these values are also shown. Where there are sub-ranges within the 68.2% or 95.4% ranges these are detailed on the following line.

**Table 7:** Start and End values for the 68.2% hpd and 95.4% hpd ranges for Second Woods to Briggs Run from 9 runs of Model 3A with A_model_ and A_overall_ values above 60 and the vast majority of C values ≥95.

Date Estimate	68.2% Start	STDEV	68.2% End	STDEV	95.4% Start	STDEV	95.4% End	STDEV
**Second Woods**	1442.0	0.0	1465.0	0.0	1419.0	0.5	1474.1	0.3
**Getman**	1453.4	0.5	1475.0	0.0	1441.3	0.5	1486.0	0.5
**Elwood**	1465.0	0.0	1485.0	0.0	1455.0	0.0	1496.1	0.3
**Smith-Pagerie**	1478.0	0.0	1498.0	0.0	1469.8	0.4	1510.1	0.3
**Otstungo**	1488.0	0.0	1510.6	0.5	1476.7	0.5	1525.6	1.0
**Klock**	1499.1	0.3	1521.0	0.0	1487.0	0.0	1555.2	2.9
** **					1557.0	1.4	1560.0	2.2
**Cayadutta**	1507.0	0.0	1540.7	0.5	1501.9	0.3	1601.6	0.7
** **	1563.4	0.5	1590.9	0.8				
**Garoga**	1517.8	0.4	1534.0	0.5	1508.3	0.7	1592.6	0.7
** **	1541.8	0.4	1575.6	0.5				
**Wormuth**	1572.4	0.5	1607.9	0.3	1541.3	0.7	1620.2	0.7
**Palatine Bridge**	1588.1	0.6	1599.6	0.7	1583.6	0.7	1633.4	0.7
** **	1613.0	0.5	1632.4	1.0				
**Briggs Run**	1598.2	0.4	1627.3	0.5	1585.4	0.5	1633.2	1.1
** **					1634.0	0.0	1635.0	0.0

The standard deviations on these values are also shown. Where there are sub-ranges within the 68.2% or 95.4% ranges these are detailed on the following line.

## Discussion

In his pioneering chronology building work for the Mohawk River Valley, Snow [17] made the first attempt in northeastern North America to use a large series of AMS radiocarbon dates to refine a chronological sequence of sites. In this effort, Snow used the AMS radiocarbon dates on maize kernels in combination with assumptions and interpretations based on Native American artifact type frequencies and the absence or presence of artifacts made from European materials. This was a reasonable approach for the time, when AMS radiocarbon dating was relatively new, dated samples per site were limited, and there was a strong tradition in the region of relative dating anchored by only one or a few radiocarbon dates (e.g., [29, 41, 42]). The results of research presented here take a further step away from building regional chronologies for archaeological sites based solely on, or combined with, ideas about changes in artifact assemblages. Like other recent Bayesian analyses in Northern Iroquoia [13, 14], our results challenge traditional interpretations of regional chronologies tied to site artifact assemblages.

The Bayesian modeling results in several changes to the occupational date ranges established by Snow [17] as shown in Fig 5 and Table 5 for Models 3 and 3A. Snell clearly dates to the second half of the thirteenth century—it does not extend into the fourteenth century as suggested by Snow [17]. At 68.2% hpd, the site’s OxCal Date estimate range is limited to the beginning of the fourth quarter of the thirteenth century, while the 95.4% hpd range extends through the last three decades of the century. Rather than being sequentially occupied at 1400–1450 and 1450–1500, respectively, the Second Woods and Getman and Elwood occupations overlap significantly with one another in the second half of the fifteenth century. While Snow had the start of their occupations separated by a century, Smith-Pagerie and Otstungo were evidently occupied simultaneously during the second half of the fifteenth century (68.2% hpd) and extending into the first quarter of the sixteenth century (95.4% hpd). The occupations of Klock and Cayadutta overlapped during the first quarter of the sixteenth century (68.2% hpd) or throughout much of the century (95.4% hpd), while Snow indicated distinct occupational ranges, with Cayadutta preceding Klock. As already discussed, Wormuth’s dated component initiated substantially later than Snow’s estimate.

Thus, while there are adjustments to all site occupation date ranges, the largest revisions occur to those sites generally thought to date to the mid- to late-sixteenth century. Smith-Pagerie and Klock date earlier than estimated by Snow [17] and others (e.g., [15, 29]), while Cayadutta’s date range initiates two decades earlier than estimated by Snow [17]. The significance of these changes involves the presence of small amounts of European metals, including iron and smelted cuprous artifacts as identified through pXRF analysis, at all the sites (S1 File, Tables 1 and 2). These artifacts led Snow to place the sites after 1525 based on the belief that the presence of European metals serves as a TPQ for site occupations in the Mohawk River Valley. Our results, rather, indicate that European metal at Smith-Pagerie, Klock, and Cayadutta is likely to have arrived earlier, during those site’s occupations, which initiated earlier than 1525. For Smith-Pagerie, with iron and smelted copper artifacts, the modeled occupation occurred in the last quarter of the fifteenth century (68.2% hpd) and extended into the first decade of the sixteenth century (95.4% hpd). Excavations at Klock produced two iron artifacts in secure contexts. Klock’s occupation originated at ~1500 and extended to the second decade of the sixteenth century (68.2% hpd), or the last half of the fifteenth and extending through the first half of the sixteenth centuries (95.4% hpd). Cayadutta, from which iron and smelted copper artifacts were recovered, originated ~1500 and extended to likely ~1540 (perhaps later to 1592: Model 3A less likely scenario) (68.2% hpd) or 1578/1602 (95.4% hpd). The European metals at these sites arrived during rather than at the outset of their occupations. Similarly, at Garoga, the smelted cuprous artifacts arrived during its occupation, which initiated in the early to mid-sixteenth century. No European artifacts were recovered from Otstungo, which has date ranges similar to/between Smith-Pagerie and Klock, during Snow’s excavations [17], although there are indications that metals have been found on and near the site [30]. For example, Lenig [30] notes the recovery of an early iron axe on the flats below Otstungo. However, this find seemingly does not relate to the site’s occupation because iron axes did not begin circulating until later in the sixteenth century (e.g., [43]). What is clear from these results is that the presence of European metal artifacts cannot be used to establish the start of a site’s occupation. Rather, European goods were obtained during the course of late fifteenth to early sixteenth-century occupations at sites in the Mohawk River Valley—very early in the histories of European metal circulation at inland sites. The European iron and copper artifacts at Smith-Pagerie are the earliest well-dated examples of European metal circulation in Northern Iroquoia. The presence of a European axe weighing 662g at Wormuth is consistent with the modeled late sixteenth to early seventeenth occupation of the site, but also indicates the adoption of European artifacts during the course of occupation, rather than as denoting a TAQ. Other than the Atlantic coast it is not possible based on current evidence to determine specific points of origin for the European metal artifacts recovered from these sites. That the Cayadutta, Garoga, Klock, and Smith Pagerie site communities were participating in networks that moved goods from the Atlantic coast into the interior is further attested by the presence of marine shell artifacts at each of the sites [15, 17, 30].

Our Mohawk Valley pXRF analyses of cuprous artifacts and Bayesian chronological modeling results open a window onto an interesting and wider perspective of how the presence and distribution of European trade goods have formed the basis to assembling chronologies until now, and how independent dating, via radiocarbon, reveals a number of serious problems with such assumptions (and consequent interpretative frameworks). This is especially true when assumptions and chronologies have been based around the presence/absence of rare items from available assemblages (which are rarely complete, nor representative of both settlement and mortuary contexts). In particular, we can compare and contrast our findings versus previous assumptions in the Mohawk Valley with similar recent reassessments based on radiocarbon and Bayesian modeling in southern Ontario to start to see the rich range of different situations and histories.

For example, whereas the current results have demonstrated that sites with European metal were occupied earlier than previous estimates, two sites lacking evidence for the adoption of European metals in the Rouge River-West Duffins drainage in southern Ontario, Draper and Spang, which were therefore thought to date to ca. 1450–1500, in fact date later. Metal was not present, at least in the excavated assemblages at Draper, which dates to the first half and Spang, which dates to the second half of the sixteenth century [13]. Another case in the Trent Valley in southern Ontario offers a different situation. Benson, with an assemblage of European metals similar to Smith-Pagerie, Cayadutta, Klock, and Garoga, was originally thought to date to the second half of the sixteenth century. However, recent radiocarbon investigation places it within the early to mid-sixteenth century (68.2% hpd), with the metal having likely circulated to the site during its occupation rather than at its beginning [14]. The Mantle (or Jean-Baptiste Lainé) site, in the Rouge River-West Duffins drainage, has an assemblage of European artifacts similar to Smith-Pagerie, Cayadutta, Klock, and Garoga. Originally Mantle was thought to date to ca. 1500–1530 [12, 44]. Radiocarbon analysis places it to the late sixteenth to early seventeenth century [13]. The absence of European material thought typical of the mid-later sixteenth to start of the seventeenth centuries within the assemblage from the site was not chronologically relevant. It instead likely reflects differential trading patterns and practices, social and political limitations and choices, geography, and possibly differences between settlement and mortuary assemblages. Contrasting with this last case is the Ball site, near Lake Simcoe in southern Ontario, which Bayesian analysis indicates dates to the second half of the sixteenth century, but which unlike Garoga, produced glass beads [14]. Here, and at the nearby Warminster site, there was likely direct contact with French traders and unusually large assemblages of European goods were available and recovered archaeologically [13, 14]. The radiocarbon dates for the sites match with the artifact-based dates of these large trade goods assemblages (again suggesting current import, versus heirlooms or other scenarios). However, access, availability, and interest for other groups in the Iroquoian world was not necessarily similar. Once dealing with scarce items and presence/absence, it becomes clear that trade goods can form a very insecure basis for chronology in the Northeast.

## Conclusion

The results of our pXRF analysis of cuprous artifacts and Bayesian analysis of a large suite of radiocarbon dates add to a growing appreciation of the inter-regional variations in the circulation and adoption patterns of European goods in northeastern North America in the sixteenth to earlier seventeenth centuries [45]. As noted by Birch and Williamson ([44], p.151), it is necessary to consider “each community to be unique in its interactions.” Whether this variation is the result of differing trade networks, competition for the control of European items, and/or preferences of individual members of communities to possess such items [13, 14], cannot be resolved based on the current sample of sites that have had assessments of occupational dates through Bayesian analysis of radiocarbon dates independent of artifactual assemblage content. However, results thus far, indicate that the early circulation of European materials through Northern Iroquoia was much more complex than has been previously understood and is a topic in urgent need of further investigation across the wider region.

## Methods

### PXRF analysis

All pXRF analysis of copper-based artifacts was performed at NYSM with a bench mounted Bruker Tracer III-V model instrument using the Cu filter with the following settings: high voltage = 151, filament current = 183, high voltage ADC = 40, filament current ADC = 3, pulse length = 200, and pulse period = 254. Following [31] the artifact surfaces were not cleaned prior to assay. Acquisition time for each artifact was 60s. Spectra were examined for peaks in addition to Cu. Artifacts with two or more trace element peaks (As, Pb, Sn, Zn) were considered of European origin [31]. Iron peaks occur on all analyzed artifacts including a piece of copper from Houghton County, Michigan and are therefore not considered as evidence of European smelting. The spectra and ROI data are provided in S1 File and summary information in Tables 1 and 2.

### Radiocarbon dating

The 58 UCIAMS samples first reported here were assayed by the W. M. Keck Carbon Cycle Accelerator Mass Spectrometry Laboratory (KCCAMS) at the University of California-Irvine [46]. At KCCAMS, bone samples were decalcified in 0.5N HCl, gelatinized at 60°C and pH 2, and ultrafiltered to select a high molecular weight fraction (>30kDa) [35]. δ^15^N was measured to a precision of <0.2‰ and δ^13^C<0.1‰ on ultrafiltered collagen aliquots with a Fisons NA1500NC elemental analyzer/Finnigan Delta Plus isotope ratio mass spectrometer at KCCAMS. Maize samples were subjected to the standard acid-base-acid (1N HCl and 1N NaOH, 75°C) pretreatment. Maize sample δ^13^C values were measured at KCCAMS to a precision of <0.1‰ relative to standards traceable to PDB with a Thermo Finnigan Delta Plus stable isotope ratio mass spectrometer (IRMS) with Gas Bench input. Details on KCCAMS dating protocols are available on the facility’s website (https://www.ess.uci.edu/group/ams/facility/ams). We report one ISGS date (Radiocarbon Dating Laboratory, Illinois State Geological Survey) and one OS date (National Ocean Sciences AMS Facility, Woods Hole Oceanographic Institution) for Palatine Bridge, which were obtained prior to the current project, but not previously published. The other radiocarbon dates employed in this study were taken from the literature. For information on all the radiocarbon dates, see S2 File.

### Bayesian modeling

Analysis of the date set was undertaken employing Bayesian chronological modelling using the OxCal software [23, 38] and the IntCal13 mid-latitude Northern Hemisphere radiocarbon calibration curve [22], current at the time of writing this paper, and with a view to transparent and robust analytical models [25, 27, 47]. We describe our modeling approach below and provide full details on all dates and models employed (see below). Our focus was on short-lived sample material, like annual-growth plant matter, e.g. maize, or on animal bones from herbivores. These samples should yield radiocarbon ages more or less contemporary with the time of their exploitation by humans, and, we can reasonably expect, of their archaeological find contexts [48]. Quality control on the radiocarbon dates for these samples was assessed using the General Outlier model in OxCal [38]. We anticipate some ‘noise’ in view of the imperfect and varying nature of the data (contexts, materials, different laboratories and technologies). Hence, we overlook minor possible outliers <10% probability (our arbitrary cut-off) but discuss those of ≥10% probability below. Some dates in the literature are also available on wood charcoal. Tree species identifications were not provided, but we have to assume that a varying in-built age (old wood) effect may apply [38, 49], thus a varying TPQ from substantial to ~0. We have employed the Charcoal Outlier model in OxCal [38] to try to allow for this in-built age element. Dates on such charcoal samples can in some cases offer important TPQ constraints to assist to resolve cases that would otherwise be ambiguous (e.g. [13]).

Our first aim was to consider chronological order within the 13 sites but as much as possible independent of subjective assumptions made previously based on the presence/absence or type of trade goods or assessments of ceramic types and so on. We made two exceptions: the Briggs Run and Palatine Bridge sites (see discussion below). The aim was thus to consider a dating model where each site was an independent Phase within an overall Phase encompassing all the sites (Note: capitalized terms like Phase, Sequence, Boundary, Order, etc. are OxCal terms [23]). Thus, the individual site Phases can overlap or separate in time as the data indicate–there is no prior assumption of any specific order. We aimed to assess the likely chronological order of the various site Phases with the Order function in OxCal. The dates for the sites variously come from site middens, pits and hearths at sites, and occasionally other contexts (like a grave fill) associated with a site. We treat individual pits as most likely single events within the lifetime of the site—thus the dates on samples from the general site midden, and other occupation/use contexts, like hearths, and the pits, can all be considered together as representative of time intervals within the lifetime of the overall site, i.e. a site Phase. We lack information on the relative placement of samples within the lifetime of each of the sites, thus we adopt the conservative assumption that the samples are random samples from a uniform distribution and so could come from any point within a site Phase with equal probability: a uniform prior assumption. Where we have multiple dates on short-lived plant material from a specific pit, we combine these together for a best estimate of the age of the pit (and to assess the hypothesis that they do in fact represent estimates of the same real radiocarbon age [50]). To achieve this, we use the R_Combine function in OxCal, including an additional 8 radiocarbon years to allow approximately for annual-scale variation in this period [51]. We employ the SSimple Outlier model for the individual data within the R_Combine and the General Outlier model for the R_Combine itself (following the coding examples in [52]). In the one case of a sample from an associated context (a grave fill at Wormuth) we include this within the site Phase (the archaeological assessment of Lenig [30], supports the graves likely belonging with this main protohistoric site occupation). We recognize that we assume approximate contemporaneity in this case, however, the assumption appears plausible, and as a sole instance and as a *Terminus Post Quem* (TPQ), since the sample was on wood-charcoal, it has very little impact on the modeling. For the Wormuth site three samples of maize lack any specific context information other than the general site, but we make the assumption that they date from the general site midden. The same applies to the samples from the Briggs Run and Palatine Bridge sites where the samples derive from avocational donations/collections (for Briggs Run, see [17], p.259) and no further specific context information is available.

Two sites are exceptions for our approach and modelling which need some discussion: Briggs Run and Palatine Bridge. We have only three dates (run several decades ago) from the former and two from the later. None are UCIAMS dates. We thus have especially poor/sparse radiocarbon data for these two sites, and no dates on charcoal samples which might have provided key TPQ information in this case (see below). The two sites both must date late in the sequence of sites given the associated material culture, around and likely after 1600. These two sites alone in our set yielded what are universally accepted as later material including in particular glass beads ([17], pp. 31, 43, 197, 239, 252–259, 279, [30]). Snow thus argues that the Briggs Run site must date from about/after the time of the establishment of Fort Nassau in 1614 when such European material became much more widely accessible in the area. Even if this assessment is considered too prescriptive and particular, the conclusion must be that the finds of substantial quantities of trade goods, including glass beads as well as metal items, at Briggs Run cannot be placed much earlier than the very late sixteenth century if not the start of the seventeenth century [34, 53]. Rumrill [53] places Briggs Run in his 1600–1615 “Early Historic Period” but notes the site may overlap with his next time period of 1615–1630.

**Fig 6:**
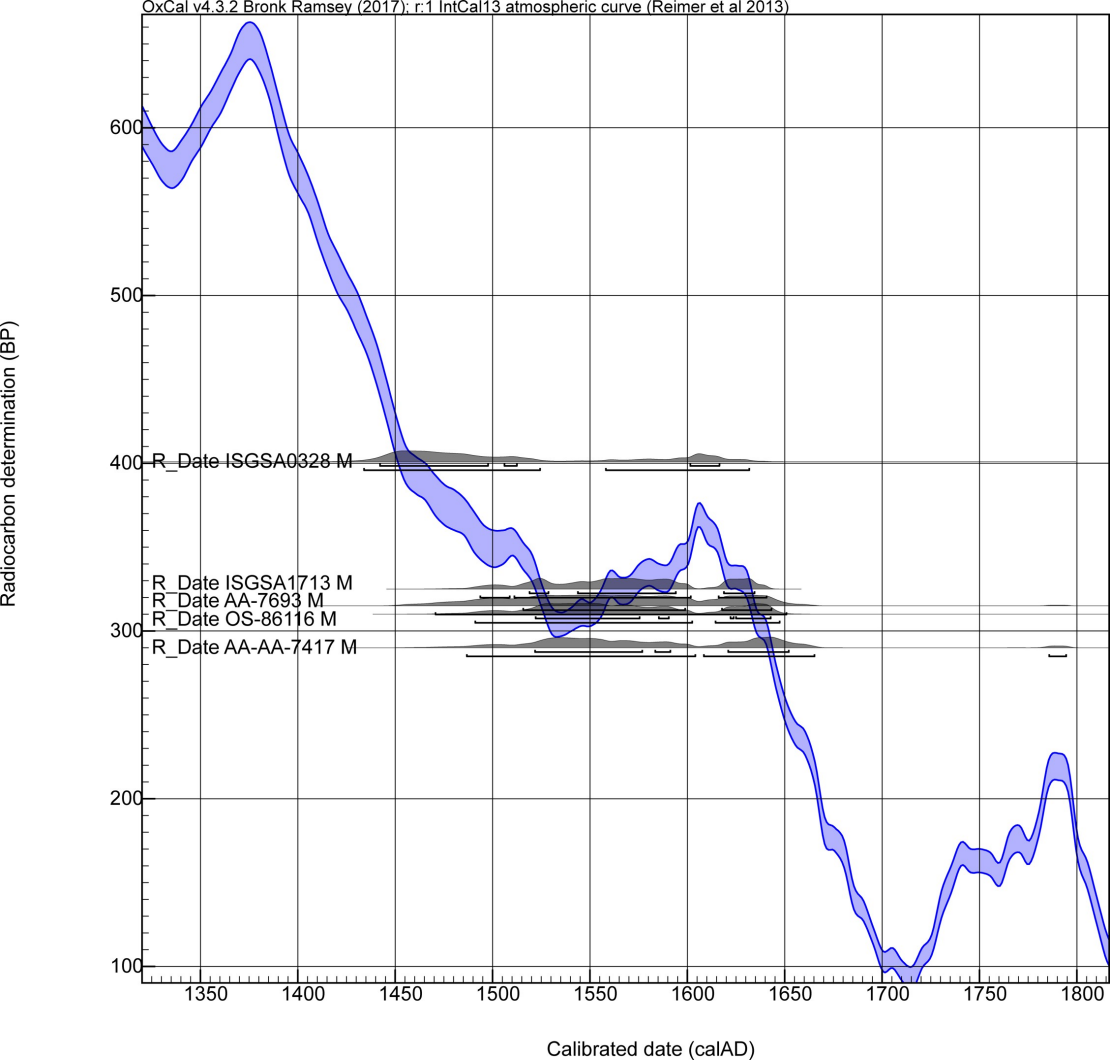
Non-modelled calibrated calendar age probabilities, and 68.2% and 95.4% probability ranges, for the five radiocarbon dates from the Briggs Run and Palatine Bridge sites shown against IntCal13 [22] using OxCal [23].

**Fig 7:**
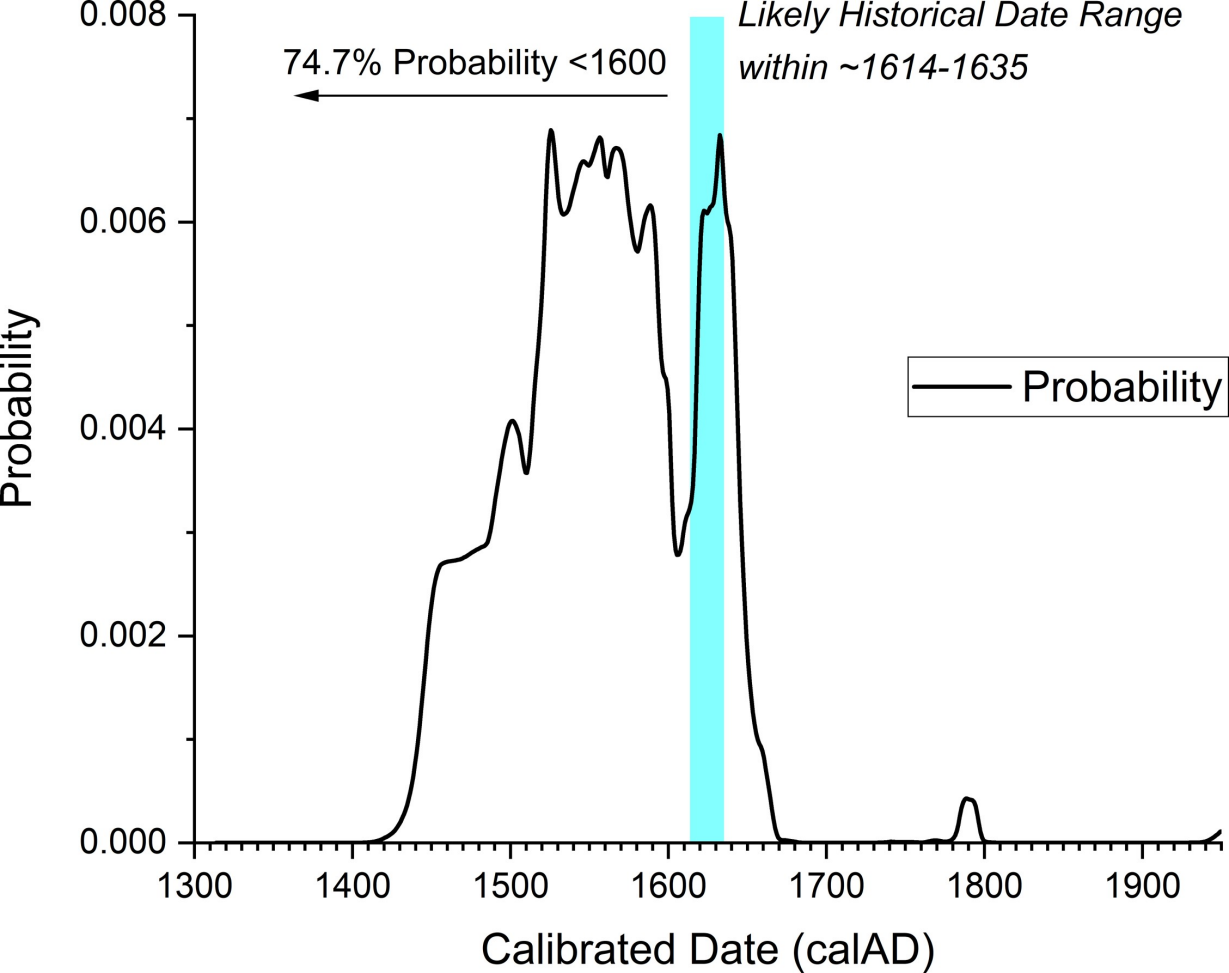
The combined normalized calendar dating probability from the five radiocarbon dates in Fig 6. 74.7% of the probability lies before 1600. Whereas it is argued on historical-archaeological grounds that the likely date of at least the Briggs Run site includes the period from 1614 to somewhere between 1626 to before 1635 ([17], pp.252–259). This later date range is also entirely compatible with the calibrated calendar probabilities (cyan bar in the figure).

**Fig 8:**
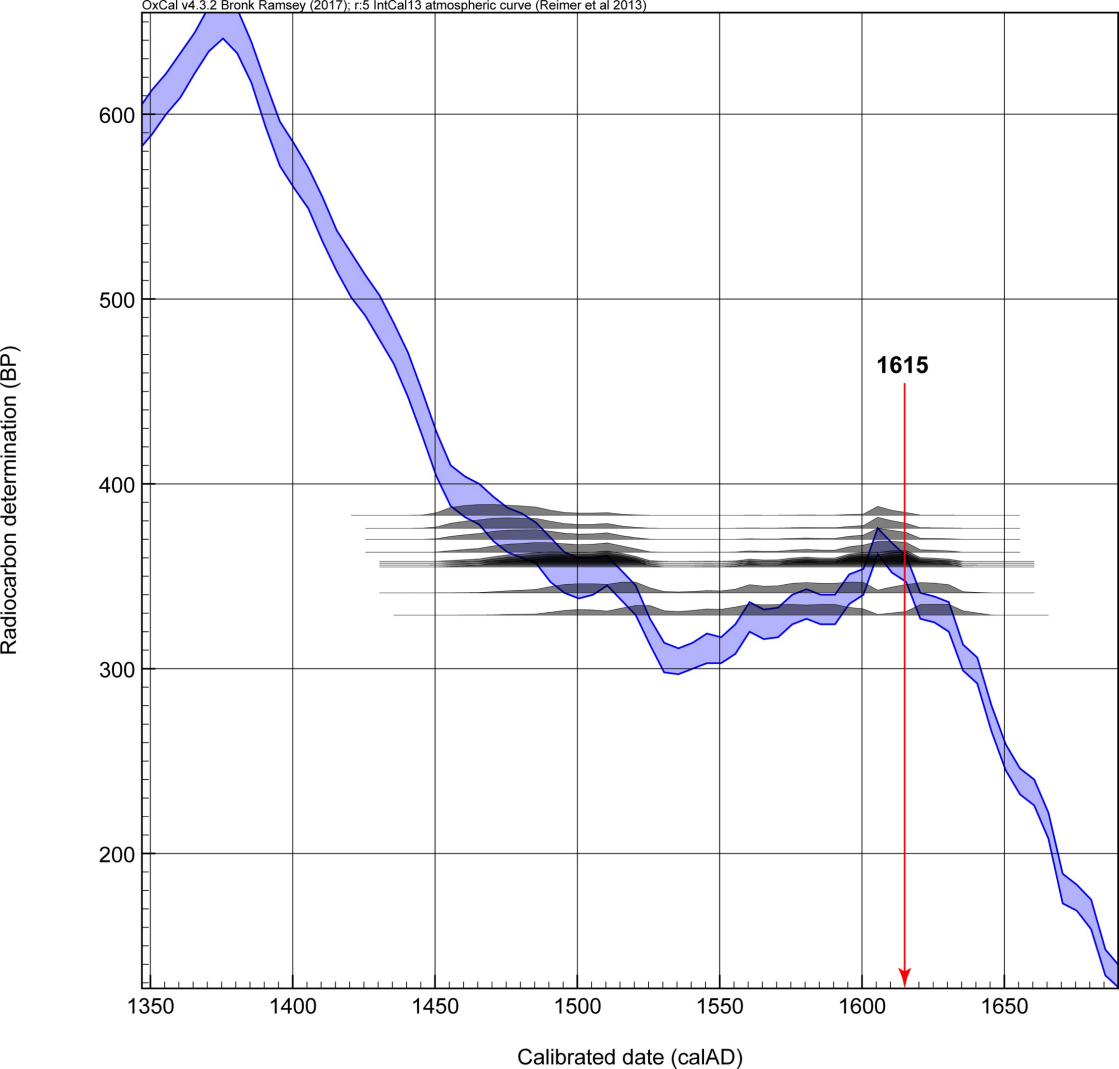
Ten simulated radiocarbon dates for 1615 with ±15 radiocarbon years measurement uncertainties (Using the R_Simulate function in OxCal [23]) with the IntCal13 radiocarbon calibration curve (curve resolution 5 years) [22]. The effect of the reversal in the calibration curve is clear with much of the calibrated probability lying up to a century or more too early.

The problem in radiocarbon terms if a site dates in the early seventeenth century, if there is no constraint on the early side from stratigraphy or from charcoal samples providing TPQ data, is that any radiocarbon dates, even if accurate, will seem to offer more of their probability in the period before 1600. They thus undermine an Order analysis. We illustrate the situation in Fig 6. The five radiocarbon dates from Briggs Run and Palatine Bridge are shown calibrated against the IntCal13 calibration curve (no modelling). The ‘M’ indicates they are all dates on samples of maize. All five dates indicate they *could* date in the expected period after ~1600 (even ISGSA0328 as a near miss for the major ‘wiggle’ in the calibration curve around 1605–1607). But a majority of the apparent probability lies before 1600, much well before 1600. Fig 7 shows the combined probability from the five dates in Fig 6. Again, we see that the likely historic date suggested for Briggs Run (and we assume approximately Palatine Bridge) falls in a likely probability range, but almost 74.7% of the total dating probability lies before 1600, and indeed fully 50% of the total probability lies before 1558 (likely ~50 or more years too early). Fig 8 emphasizes this point. We simulate 10 random radiocarbon ages for 1615 assuming a measurement precision of ±15 radiocarbon years. The resultant calibrated probability distributions all include the correct age and look very like some of the distributions in Fig 6, but most of the probability ends up earlier and much far too early.

We note that recent work has highlighted that the absence of various types of trade goods is not necessarily a reliable chronological indicator in the northeast [13, 14]. However, at the same time, positive evidence of finds of substantial quantities of a considerable range of trade goods including glass beads and metals nonetheless does offer a plausible TPQ. These items were simply not available in the wider region before the late sixteenth century at the very earliest (e.g. a date like after 1580 which is typically applied for the earliest glass beads in the Mohawk or Iroquois region [34, 53, 54]), and in all likelihood not until about the start of the seventeenth century [43]. In support, it can be observed in cases of sites with finds of large such trade goods assemblages, and good radiocarbon dating data sets, that the radiocarbon analysis offers calendar date ranges very compatible with existing assessments based around the trade goods (e.g. the Ball and Warminster sites [13, 14]). Thus, to return to Briggs Run and Palatine Bridge, the unqualified or unconstrained radiocarbon evidence from these sites indicating half or more of the possible probability range lies before e.g. ~1560 is clearly unhelpful and misleading. We therefore adopted the strategy that we did not include these two sites in our initial Order analysis. We only considered them later in our investigation after first and independently establishing the order of the sites before the early seventeenth century.

In order to facilitate the model to run, we restricted the possible overall age range for each of the 11 sites in our initial model. Ruling out the three clearly very much too old early Michigan dates on charcoal from Snell (TPQ dates), all the other radiocarbon dates offer individual calibrated ranges at 95.4% probability only beginning after 1150. Thus, we introduce the constraint that each site must start after 1150. At the recent end we can plausibly employ an historical *terminus ante quem* (TAQ). There was a Mahican attack on the Mohawk area in 1626, and a report by Van den Bogaert of 1635 (after an investigative trip in late 1634 and early 1635) that states that Mohawk sites north of the river had been abandoned by that date (whether directly after the 1626 attack but certainly by 1635) and were now only found south of the river ([17], pp.35, 37, 242, 257, 279). In addition, smallpox broke out in the Mohawk area from 1634 severely disrupting the local economy ([17], p.279, [55], pp.58–59). The net assessment is that all the later members of the set of sites we consider can reasonably be dated as *before* this historically documented 1626–1635 period of upheaval. Thus, we apply the constraint that each site ends by 1635. We therefore apply 1150 and 1635 constraints to the start and end Boundaries for each of the 11 sites.

A final consideration is the plausible length (duration) of any one of the individual Mohawk settlement sites. This becomes evident when one begins analysis. A first model (Model 1) with all the dates for the 11 site Phases was run with each as overlapping site Phases with a query for the Interval–the length of time between the start Boundary and the end Boundary for each of the site Phases. The model often did not produce stable results. OxCal A_model_ and A_overall_ values ranged quite widely (over six example runs A_model_ was 46.4, 47, 48.5, 50.2, 53.1 and 55.5; and A_overall_ was 40.2, 41.2, 46.9, 48.8, 50.9 and 53.1). Both measures should be above 60 to be deemed satisfactory, but for a large ‘all data’ model lower values are often encountered. The real problem was that in many runs one or more site Phases did not converge. OxCal Convergence (C) values should be ≥95. But in 5 of the 6 run one or two site Phases achieved values for the whole group of elements <10 (in the six cases: this included the Otstungo (twice), or Pethick + Garoga, or Pethick + Cayadutta, or Garoga). The changing non-converging site(s) indicates a wider problem, and not that one specific site is the difficulty. The real issue is the apparent site Phase durations from the Interval query. Across the six runs of Model 1 these varied widely, but consistently they were very long for several sites: Table 3. Even, and indeed especially, for the best run (A_model_ 55.5 and A_overall_ 53.1 and with Convergence values all ≥95 (S4 File)) this problem is very evident: Table 3. While a few Intervals were reasonable, a number of others manifestly were not, for example: Getman 56–119 years at 68.2% highest posterior density (hpd) and 20–204 years at 95.4% hpd, Smith-Pagerie 97–203 years at 68.2% hpd and 75–246 years at 95.4% hpd, Otstungo 74–175 years at 68.2% hpd and 20–203 years at 95.4% hpd, Cayadutta 112–258 years at 68.2% hpd and 0–335 years at 95.4% hpd and Garoga 178–306 years at 68.2% hpd and 110–485 years at 95.4% hpd. We do not, of course, know the occupation duration of any of these sites. However, the usual assumption, based on ethnography and archaeology, is that the period of occupation of the individual sites was, typically, not very long, measured in periods of decades and not much longer. The evidence especially for those ethnographically attested site occupations in the Northeast suggest typical periods of two to a few decades (e.g. [39]). Snow ([17], p.135) argues that some pre-seventeenth-century sites perhaps had longer durations than the shorter (only a few decades) durations attested for seventeenth-century sites, but, even so, no one (including Snow [17]) assumes durations lasting more than several decades to 50–75 years at the very most for a variety of reasons. Hence, the 100+ years durations suggested for some sites, due to probability spreading out across the plateau in the calibration curve, is excessive and misleading. To try to avoid this issue of possible overlong site Phases, we therefore introduced a constraint on an Interval query for each site Phase. The Interval query determines the period of time between the start and end Boundary for each site Phase and we set this as a uniform probability between 0 to 120 calendar years. This is a reasonable, generous, non-determinative compromise for sites which we assume likely had overall durations in the range (0–40 years), typically of no more than about one-third to at most about one-half of this constraining length of time–and yet rules out implausible site durations of well over 100 years. We consider the effects of such Interval constraints on some hypothetical known-age data in S1 Appendix and find that they typically slightly increase correct Order resolution, but we also highlight the need for appropriate caution.

A re-run of Model 1 with the Interval constraint of 0–120 years for each site Phase resulted in a more or less stable model. Across 6 model runs the range of OxCal A_model_ values ranged from only 36.8 to 39.5 and the A_overall_ values from 34.5 to 35.9. Six dates are typically identified as outliers at ≥10% probability: UCIAMS-218491 at 46% (we should also note that we do not know what this sample in fact comprised—it was assumed to be maize when it was submitted for dating but very clearly it is not with a δ^13^C value of -29.1—and so it is both suspect as substantially too recent—intrusive?—and as of unknown constitution), UCIAMS218493 at 39%, AA-7689 at 60%, AA-7695 at 52%, AA-7404 at 11% and AA-8370 at 99%. In all but one case these outlier dates were too old, and, apart from other possible sources of error, some (especially the larger outliers) may reflect older occupation/residual material at some of the sites (as suggested in several cases by Snow [17]). We used a calibration curve resolution of 1 year for the Order analysis. We then re-ran Model 1 excluding these six larger outliers as Model 2. Versions of Model 2 typically achieved satisfactory OxCal A_model_ and A_overall_ values >60. We considered different Interval constraints of 0–80 years, 0–100 years and 0–120 years to check for whether such variation had an impact on the Order analysis. Model 2 with an Interval constraint of 0–120 years for each site Phase is shown in summary in Fig 2 and the full results from the three model versions are listed in S5 File and S6 File and Table 4. In runs with the site Phase Interval constraint at 0–100 years and 0–80 years typically one additional date was found to be a larger outlier ≥10%. This is UCIAMS192976 from Getman (only a 6% outlier in the 0–120 constraint model, or 7% outlier in our original Model 1), which is flagged as too old versus the other dates in the site Phase with a shorter site Phase constraint. It is a 17% probability outlier in some runs of the 0–100 years model (other outliers are six at ~6% and two at ~8%) and a 13% probability outlier in some runs of the 0–80 years model (other outliers are five at ~6%, two at ~7% and two at ~8%). We re-ran these two versions of Model 2, excluding UCIAMS192976. The Order found for the sites did not vary. We employ these two re-run models in Table 4. Examination of the Order probabilities comparing the start Boundaries, Date Estimates and end Boundaries of each of the site Phases shows a generally clear approximate order among the Date estimates across the model versions (S6 File, Table 4), but variability among the end and start Boundaries indicates likely overlaps in most cases except for Snell and Pethick. The one very close call in terms of Order probabilities is Cayadutta v. Garoga. Model 2, 0–120 years Interval, places Cayadutta older with a probability of 0.52, the 0–100 years interval reduces this probability to 0.51 (P = 0.513), and it is more or less equal at 0.50 (P = 0.5024) with the 0–80 years Interval version. Visual inspection of the respective calibrated probability distributions shows several of those from Cayadutta with earlier (around 1500 and early sixteenth century) and later (either side 1600) possible ranges, whereas those from Garoga are more solidly sixteenth century (see Fig 2, S5 File). Thus, while the two sites have long been considered close, if not parallel, in time (e.g. [17], pp.144, 189), this circumstance suggests that Cayadutta is either more to the earlier side or more to the later side than Garoga, rather than strictly coeval. In line with the slight Order indications (Cayadutta slightly older), Garoga has more evidence of European trade goods and other finds suggestive of a somewhat later date than Cayadutta ([17], pp.151–160). Hence, we consider it most likely that Cayadutta dates before Garoga and use this Sequence in Model 3 (Fig 3). However, we also consider a Model 3A where the two sites are treated as parallel and floating versus each other, and not as ordered (Fig 4).

Once we establish a likely site Phase Order independently from the radiocarbon evidence through the Order analysis (see S6 File, Table 4), our second aim was to consider a more refined dating of the set of site Phases after applying the available OxCal Order information as an additional set of constraints. As discussed below, in this Mohawk Valley case it appears inappropriate to assume there is a clear contiguous site Sequence where Site A is > Site B is > site C, etc. (such a model applies only for a few instances: e.g. Snell > Pethick). Further, the inherent temporal compression involved in making a contiguous Sequence assumption incorporating a large number of sites appears highly inappropriate in this case when there is no reason to assume that many of the sites are either strictly successive or of very brief duration. But at the same time, we also have a clear Order trend where the central OxCal Date estimates for each site Phase follow a sequence, but the starts and ends of each Phase especially may/should overlap. Hence, we have a temporally informed overlapping site model. To try to offer an approximately appropriate model for this situation, we place the distinct Snell and Pethick Phases in an initial Sequence, but then kept the remaining site Phases independent in an overall Phase (the overlapping Phase model). Within this overall Phase for the 9 sites we constrain the Date estimates for each site Phase to fit the Order data found for the Date estimates in Model 2 and we employ the 0–100 years Interval constraint. Hence there is a Sequence among the OxCal Dates estimates for each site of Second Woods > Getman > Elwood > Smith-Pagerie > Otstungo > Klock > Cayadutta > Garoga > Wormuth. We linked this Sequence to the Date estimate in each independent site Phase within its own Phase Boundaries via a cross-reference. The Boundaries and Dates for the various site Phases thus overlap substantially, but there is also a Sequence in the Date estimates as the data indicate. Otherwise, we kept the dataset and constraints of Model 2. In Model 3A the site Phases of (i) Cayadutta + Garoga and (ii) Palatine Bridge + Briggs Run are placed together in Phases, and so not ordered relative to each other in the cross-referenced Sequence.

**Fig 9:**
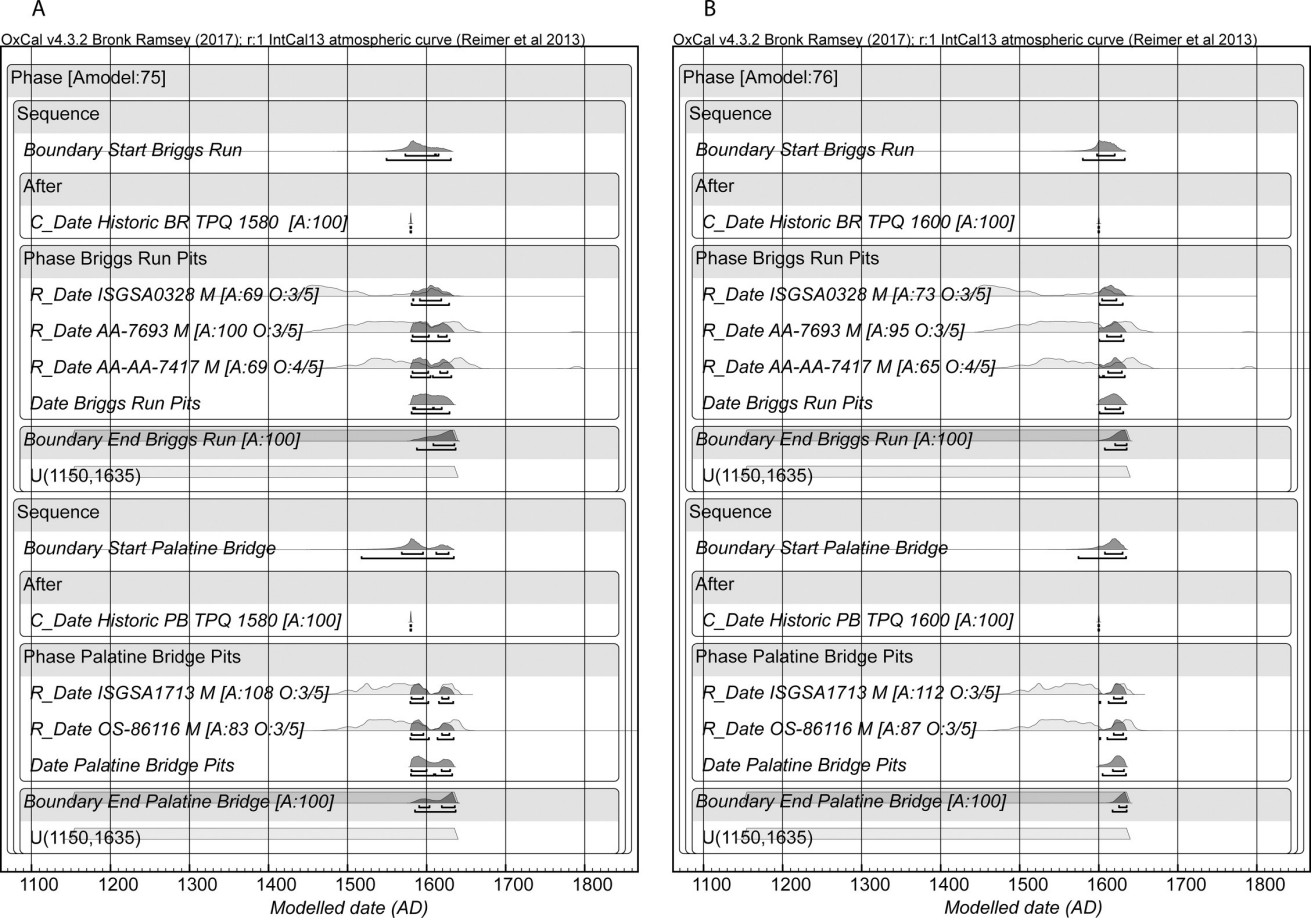
Comparisons of Briggs Run and Palatine Bridge applying (A) 1580 TPQ and (B) 1600 TPQ. Briggs Run appears slightly the more recent site in A (see Table 8) and slightly the older site in B. In each case the two sites clearly overlapped in calendar time.

In one additional step we included the two likely early seventeenth century sites of Briggs Run and Palatine Bridge. We re-ran the Model 2 Order analysis with Interval constraint 0–100 years but adding in the Briggs Run and Palatine Bridge site Phases with the conservative TPQ of 1580 applied to these two sites (see above). This finds that all the Date estimates for the nine site Phases are clearly older than Briggs Run and Palatine Bridge (lowest probability is Wormuth and this is still older with p = 0.80 and p = 0.78 respectively): Table 8. Thus, we placed Briggs Run and Palatine Bridge after Wormuth in a Sequence of the Date estimates for the site Phases. The Order between Briggs Run and Palatine Bridge is less clear. In an Order test, running the Briggs Run and Palatine Bridge site Phases in an over-arching Phase with an Order query, a TPQ of 1580 applied to both sites, and an end Boundary constraint of 1150–1635 for both sites, we find that Palatine Bridge is likely the slightly older site: see Fig 9. Calibration curve resolution set at 1 year. The comparisons of the start Boundaries places Palatine Bridge as older (p = 0.54), the comparisons of the Date estimates places Palatine Bridge as older (p = 0.52) and the comparisons of the end Boundaries places Palatine Bridge as older (p = 0.51). We use this order in Model 3. However, if we instead applied a TPQ of 1600, which might be more plausible given the historical information, then the situation reverses, and Briggs Run is the apparently older site with respective probabilities of p = 0.66, p = 0.67 and p = 0.65: see Fig 9. Our conclusion is that both site Phases may thus be largely contemporary, and so we consider in Model 3A the situation where the two sites are treated as parallel and not ordered. Models 3 and 3A are shown in Figs 3 and 4 and Table 5. We employ a calibration curve resolution of 1 year. The OxCal run files for Model 1, an example of Model 2, and for Models 3 and 3A are listed in S3 File. Finally, we consider Models 4 and 4A which are Model 3 and 3A but without the Palatine Bridge and Briggs Run sites (S9 File). In the OxCal run files, Figs and Tables, if not further described, M = Maize, B = Animal (herbivore, deer) Bone, R = pottery encrusted food residue, and C = Charcoal (see S2 File).

**Table 8:** A re-run of Model 2 with uniform probability Interval query constraint of 0–100 years applied to each site Phase and adding Palatine Bridge and Briggs Run with a 1580 TPQ applied to both these sites.

Probability *t*_1_ < *t*_2_													
*t*_1_	*t*_2_												
	Date Snell	Date Pethick	Date Second Woods	Date Getman	Date Elwood	Date Smith-Pagerie	Date Otstungo	Date Klock	Date Cayadutta	Date Garoga	Date Wormuth	Date Briggs Run	Date Palatine Bridge
**Date Snell**	0.00	***1*.*00***	***1*.*00***	***1*.*00***	***1*.*00***	***1*.*00***	***1*.*00***	***1*.*00***	***1*.*00***	***1*.*00***	***1*.*00***	***1*.*00***	***1*.*00***
**Date Pethick**	0.00	0.00	***1*.*00***	***1*.*00***	***1*.*00***	***1*.*00***	***1*.*00***	***1*.*00***	***1*.*00***	***1*.*00***	***1*.*00***	***1*.*00***	***1*.*00***
**Date Second Woods**	0.00	0.00	0.00	***0*.*63***	***0*.*68***	***0*.*91***	***0*.*91***	***0*.*98***	***0*.*97***	***0*.*99***	***0*.*97***	***1*.*00***	***1*.*00***
**Date Getman**	0.00	0.00	0.37	0.00	***0*.*53***	***0*.*79***	***0*.*83***	***0*.*93***	***0*.*93***	***0*.*98***	***0*.*94***	***1*.*00***	***1*.*00***
**Date Elwood**	0.00	0.00	0.32	0.47	0.00	***0*.*80***	***0*.*82***	***0*.*93***	***0*.*92***	***0*.*96***	***0*.*93***	***0*.*99***	***0*.*99***
**Date Smith-Pagerie**	0.00	0.00	0.09	0.21	0.20	0.00	***0*.*59***	***0*.*74***	***0*.*74***	***0*.*82***	***0*.*78***	***0*.*92***	***0*.*91***
**Date Otstungo**	0.00	0.00	0.09	0.17	0.18	0.41	0.00	***0*.*63***	***0*.*67***	***0*.*74***	***0*.*72***	***0*.*92***	***0*.*92***
**Date Klock**	0.00	0.00	0.02	0.07	0.07	0.26	0.37	0.00	***0*.*55***	***0*.*61***	***0*.*61***	***0*.*87***	***0*.*86***
**Date Cayadutta**	0.00	0.00	0.03	0.07	0.08	0.26	0.33	0.45	0.00	***0*.*52***	***0*.*55***	***0*.*83***	***0*.*82***
**Date Garoga**	0.00	0.00	0.01	0.02	0.04	0.18	0.26	0.39	0.48	0.00	***0*.*57***	***0*.*94***	***0*.*93***
**Date Wormuth**	0.00	0.00	0.03	0.06	0.07	0.22	0.28	0.39	0.45	0.43	0.00	***0*.*80***	***0*.*78***
**Date Briggs Run**	0.00	0.00	0.00	0.00	0.01	0.08	0.08	0.13	0.17	0.06	0.20	0.00	0.47
**Date Palatine Bridge**	0.00	0.00	0.00	0.00	0.01	0.09	0.08	0.14	0.18	0.07	0.22	***0*.*53***	0.00

All 11 other sites clearly date before Briggs Run and Palatine Bridge (78 to 100% probability). Palatine Bridge is slightly older than Briggs Run in this analysis (compare Fig 9). Probabilities >0.5 are in bold italic font.

## Supporting information

S1 File Reports of pXRF analyses of cuprous artifacts. Details on pXRF analyses of the cuprous objects from the Mohawk Valley area summarized in Tables 1 and 2.
(PDF)

S2 File Radiocarbon database for Mohawk Valley. The set of radiocarbon dates and their contexts available for this reassessment of 13 Mohawk Valley sites. The individual non-modelled calibrated calendar age ranges for each date are also given using OxCal [23] and the IntCal13 Northern Hemisphere radiocarbon calibration curve [22] (curve resolution set at 5 years).
(XLS)

S3 File OxCal Runfiles. The OxCal [23, 38] runfiles for a range of the models employed in the paper.
(PDF)

S4 File Table of results from Model 1 and Figures (part 1 and part 2) showing the results. Example of the run of Model 1 in Table 3 with the best A_model_ (55.5) and A_overall_ (53.1) values. Unmodelled results are the individual calibrated ranges for the samples (68.2% and 95.4% probability). Modelled results are after applying the model, results are 68.2% hpd and 95.4% hpd. A = OxCal Agreement index value (should be above 60 if data agree with model), C = OxCal Convergence value (should be ≥95).
(DOCX)

S5 File Table of results from Model 2 with 0–120 calendar years uniform probability constraint on an Interval query for each site Phase and Figures (part 1 and part 2) showing the results. Example of a run of Model 2 with good A_model_ (89.8) and A_overall_ (83.8) values. Unmodelled results are the individual calibrated ranges for the samples (68.2% and 95.4% probability). Modelled results are after applying the model, results are 68.2% hpd and 95.4% hpd. A = OxCal Agreement index value (should be above 60 if data agree with model), C = OxCal Convergence value (should be ≥95).
(DOCX)

S6 File Model 2 Order Results for the 0–120 years, 0–100 years and 0–80 years uniform probability Interval constraint models. (XLSX)

S7 File Table of results from Model 3 with 0–100 calendar years uniform probability constraint on an Interval query for each site Phase and Figures (part 1 and part 2) showing the results. Example of a run of Model 3 with good A_model_ (82.1) and A_overall_ (69.1) values. Unmodelled results are the individual calibrated ranges for the samples (68.2% and 95.4% probability). Modelled results are after applying the model, results are 68.2% hpd and 95.4% hpd. A = OxCal Agreement index value (should be above 60 if data agree with model), C = OxCal Convergence value (should be ≥95). Note some of the Palatine Bridge and nearly all the Briggs Run elements fall below this level (but not by a large amount)–see text.
(DOCX)

S8 File Table of results from Model 3A with 0–100 calendar years uniform probability constraint on an Interval query for each site Phase and Figures (part 1 and part 2) showing the results. Example of a run of Model 3A with good A_model_ (80.9) and A_overall_ (70) values. Unmodelled results are the individual calibrated ranges for the samples (68.2% and 95.4% probability). Modelled results are after applying the model, results are 68.2% hpd and 95.4% hpd. A = OxCal Agreement index value (should be above 60 if data agree with model), C = OxCal Convergence value (should be ≥95). Note one of the Palatine Bridge and nearly all the Briggs Run elements fall below this level (but not by a large amount)—see text.
(DOCX)

S9 File Tables of results from Models 4 and 4A with 0–100 calendar years uniform probability constraint on an Interval query for each site Phase. Example of a run of Model 4 with good A_model_ (87.6) and A_overall_ (77.6) values, and of Model 4A with good A_model_ (88.1) and A_overall_ (77.7) values. Unmodelled results are the individual calibrated ranges for the samples (68.2% and 95.4% probability). Modelled results are after applying the model, results are 68.2% hpd and 95.4% hpd. A = OxCal Agreement index value (should be above 60 if data agree with model), C = OxCal Convergence value (should be ≥95). The models are the same as Model 3 (S7 File) and 3A (S8 File) except for the removal of the Palatine Bridge and Briggs Run site data. Without the TAQ factor of the Palatine Bridge and Briggs Run sites the later sites, and especially Wormuth, are found to potentially have more recent date ranges. The Model 4 and 4A date ranges for some sites are compared to those from Models 3 and 3A at the end of this file.
(DOCX)

S1 Appendix Comparison test cases for the use of an Interval constraint on hypothetical known-age site Phase data. (PDF)
