# Supplementary material for: Radiocarbon, Bayesian chronological modeling and early European metal circulation in the sixteenth-century AD Mohawk River Valley, USA
Source: PLoS One. 2019 Dec 16;14(12):e0226334. doi: 10.1371/journal.pone.0226334 (PMC6913979; doi:10.1371/journal.pone.0226334)
Supplement: S1 File — Details on pXRF analyses of the cuprous objects from the Mohawk Valley area summarized in Tables 1 and 2. (PDF) [file pone.0226334.s001.pdf]

NYSM Mineralogy #7005. Native copper from Houghton County, Michigan

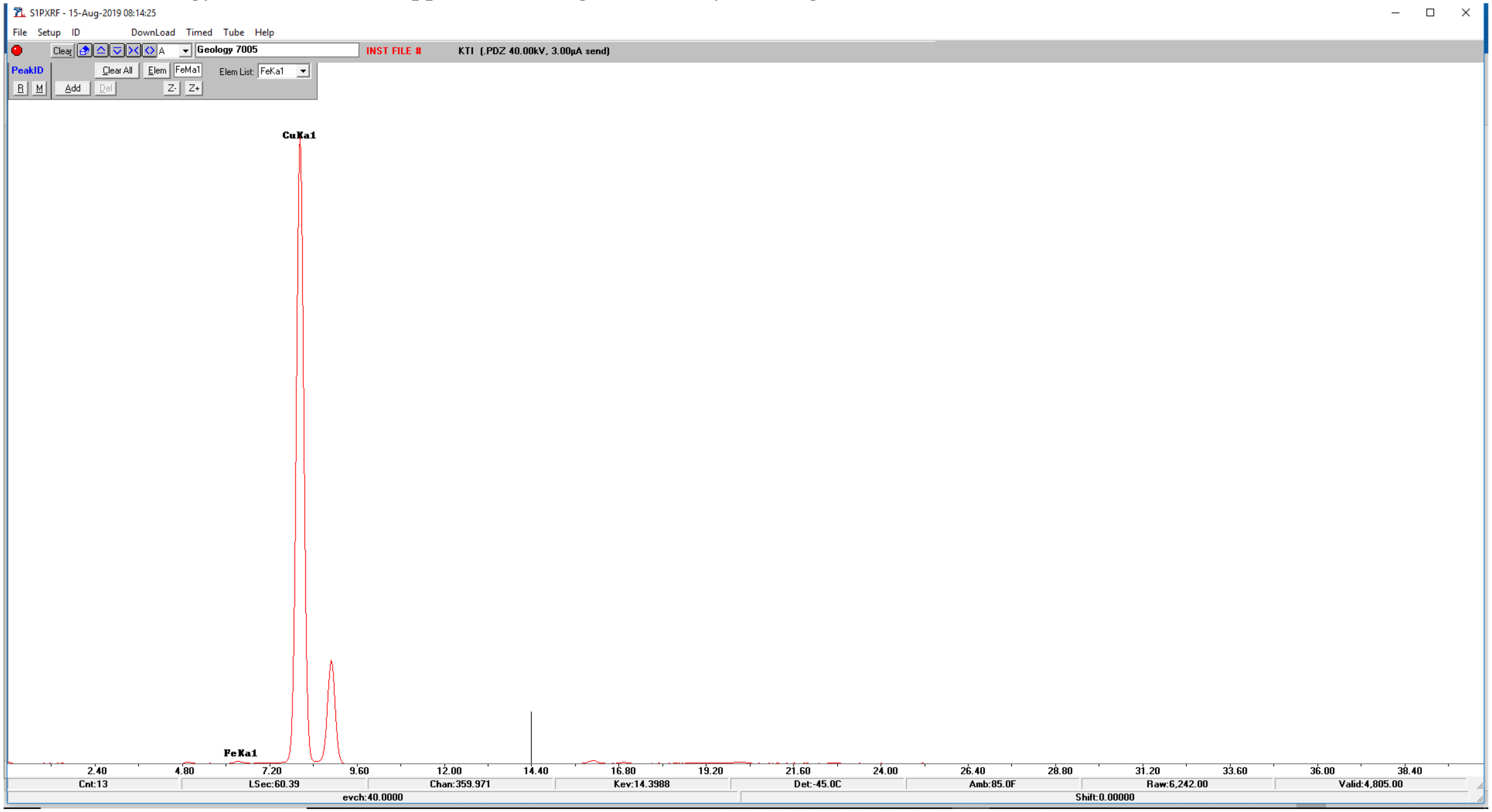


| Geology 7005 | Energy1 | Energy2 | Chan-Start | Chan-End | Chan-Counts | ROI |
|---|---|---|---|---|---|---|
| FeKa1 | 6.2347 | 6.5730 | 155.8667 | 164.3253 | 887.85 | 0.00408 |
| CuKa1 | 7.8658 | 8.2298 | 196.6451 | 205.7439 | 216701.80 | 0.99592 |

Morse A-20529 pre-contact rolled native copper bead, Jefferson County, New York

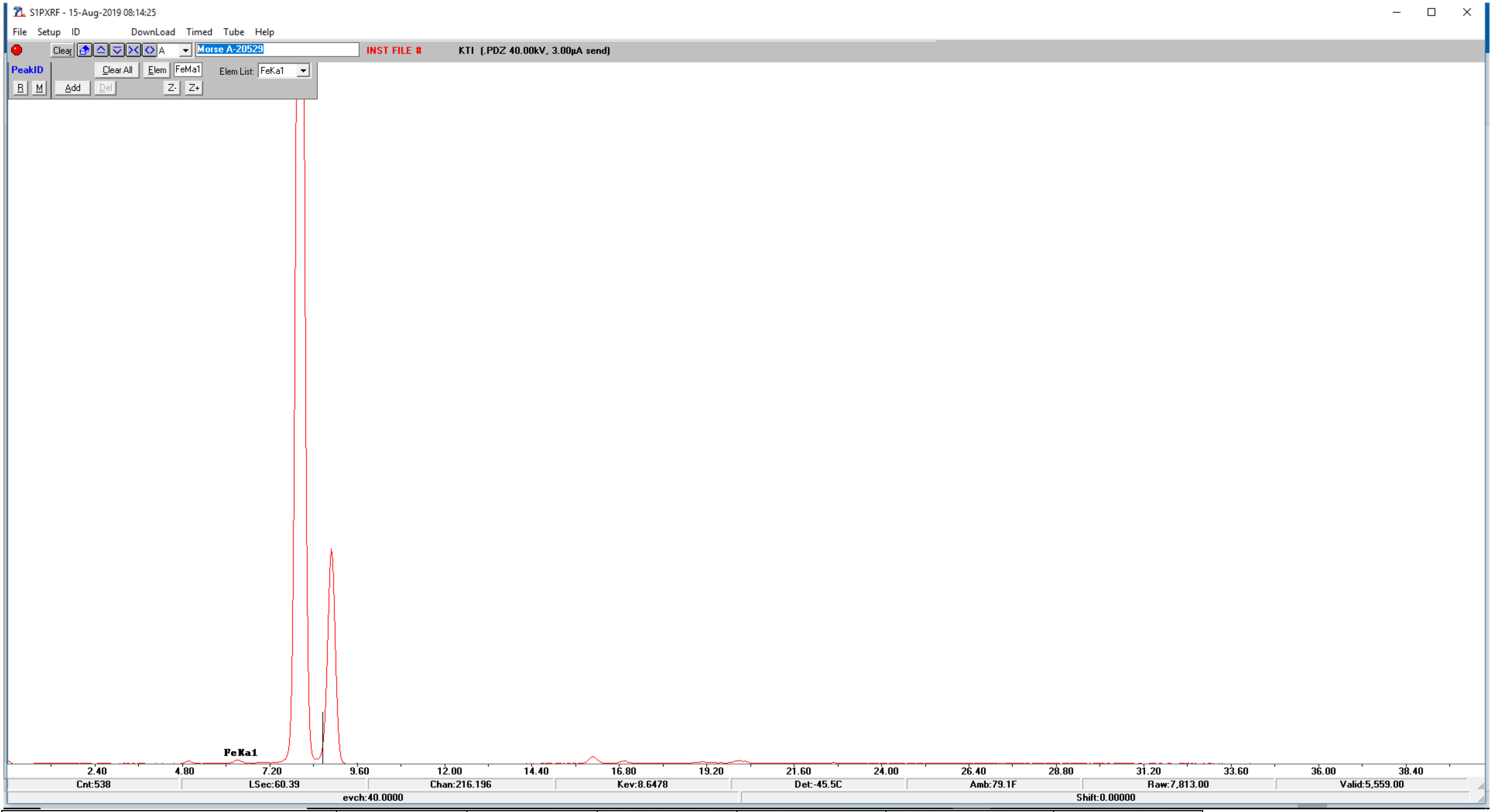


| Morse A-20529 | Energy1 | Energy2 | Chan-Start | Chan-End | Chan-Counts | ROI |
|---|---|---|---|---|---|---|
| FeKa1 | 6.2347 | 6.573 | 155.8667 | 164.3253 | 809.51 | 0.003224 |
| CuKa1 | 7.8658 | 8.2298 | 196.6451 | 205.7439 | 250266.3 | 0.996776 |

Morse A-27393A pre-contact rolled native copper bead, Jefferson County, New York

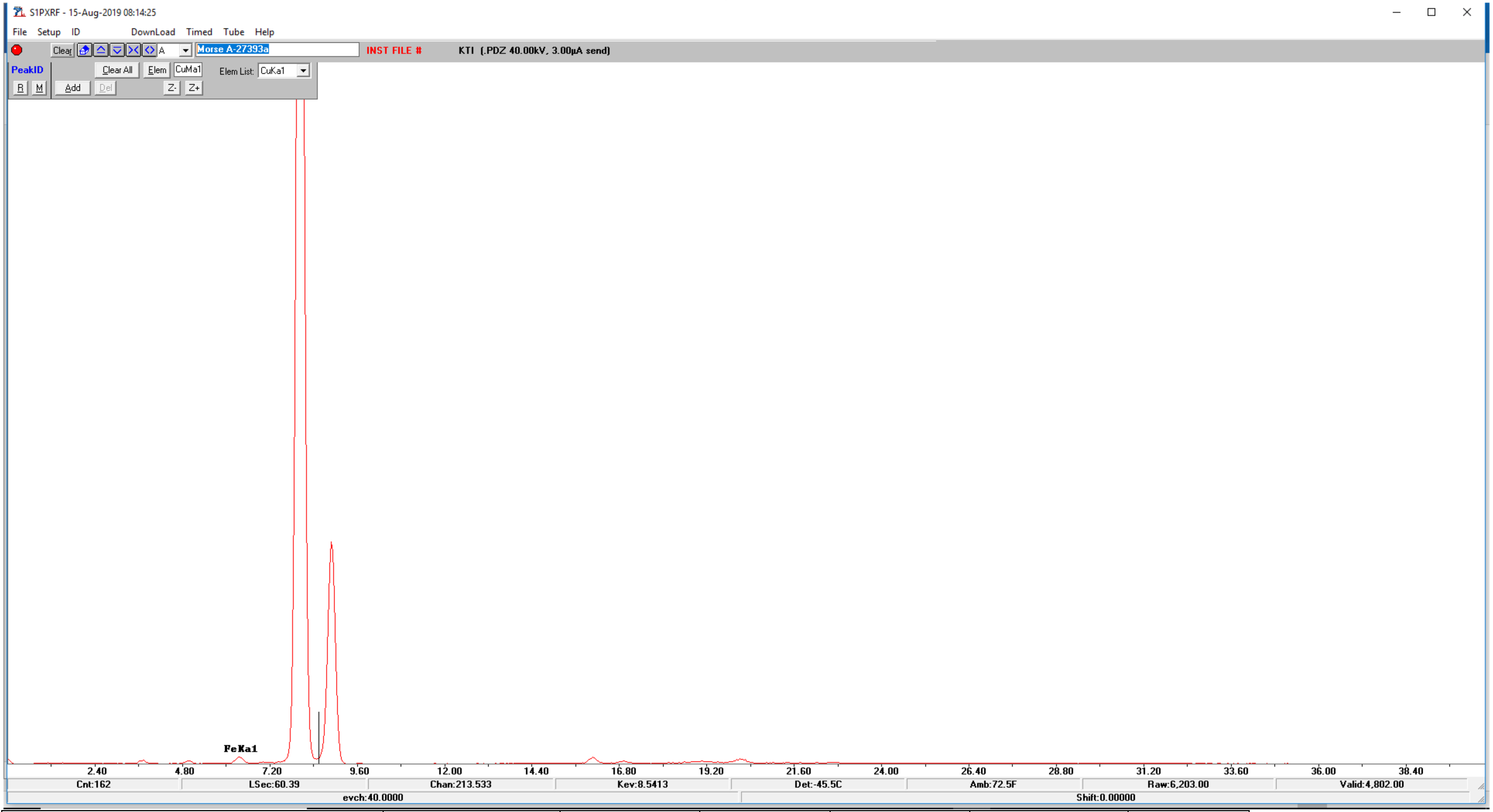


| Morse A27393A | Energy1 | Energy2 | Chan-Start | Chan-End | Chan-Counts | ROI |
|---|---|---|---|---|---|---|
| FeKa1 | 6.2347 | 6.573 | 155.8667 | 164.3253 | 1207.03 | 0.005765 |
| CuKa1 | 7.8658 | 8.2298 | 196.6451 | 205.7439 | 208171.4 | 0.994235 |

Morse A-27393D pre-contact rolled native copper bead, Jefferson County, New York

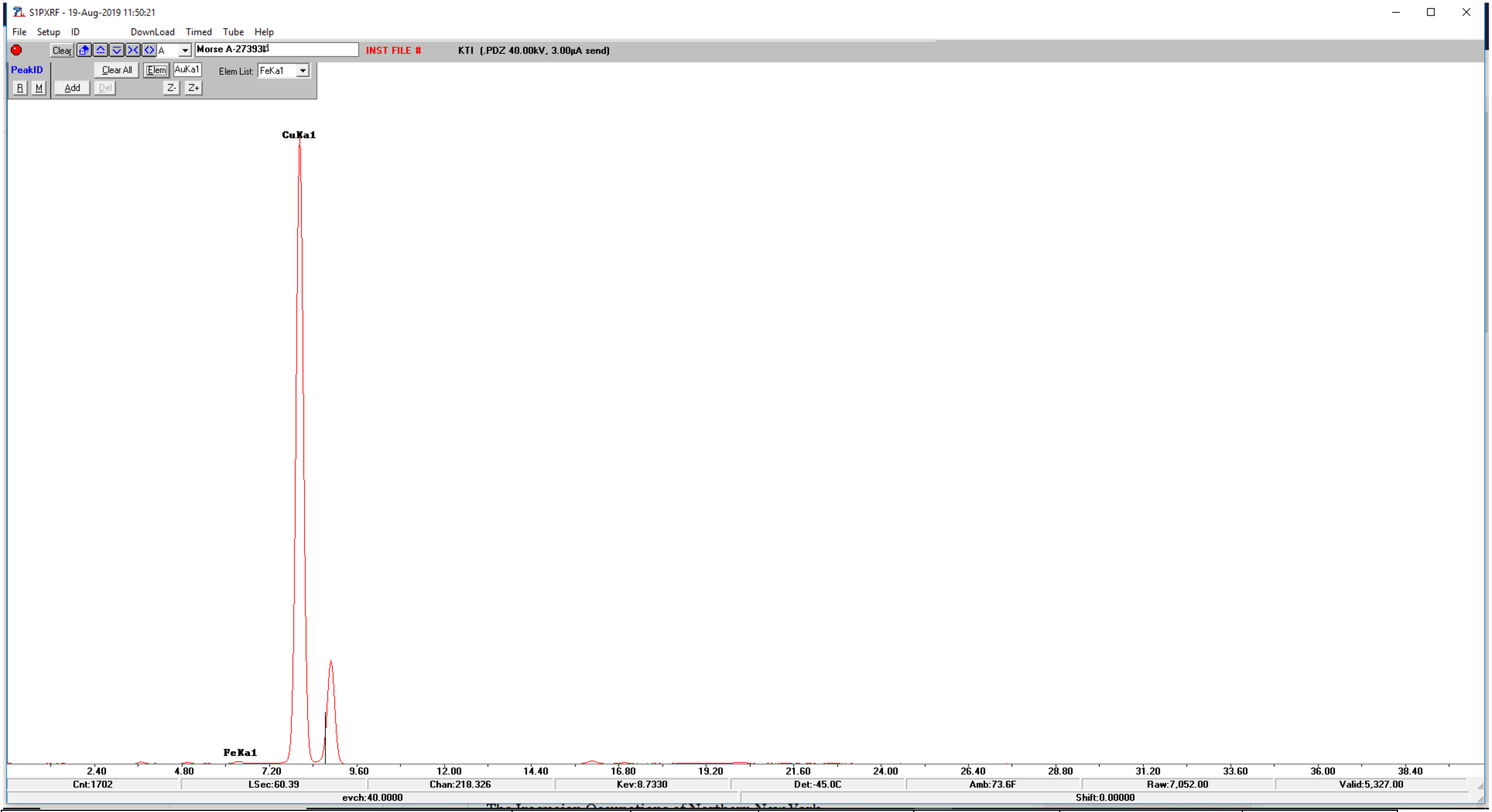


| Morse A-27393d | Energy1 | Energy2 | Chan-Start | Chan-End | Chan-Counts | ROI |
|---|---|---|---|---|---|---|
| FeKa1 | 6.2347 | 6.573 | 155.8667 | 164.3253 | 1015.58 | 0.0043560 |
| CuKa1 | 7.8658 | 8.2298 | 196.6451 | 205.7439 | 232126.76 | 0.9956439 |

Getman A2002.47DU.99.10 pre-contact native copper crescent-shaped tanged knife.

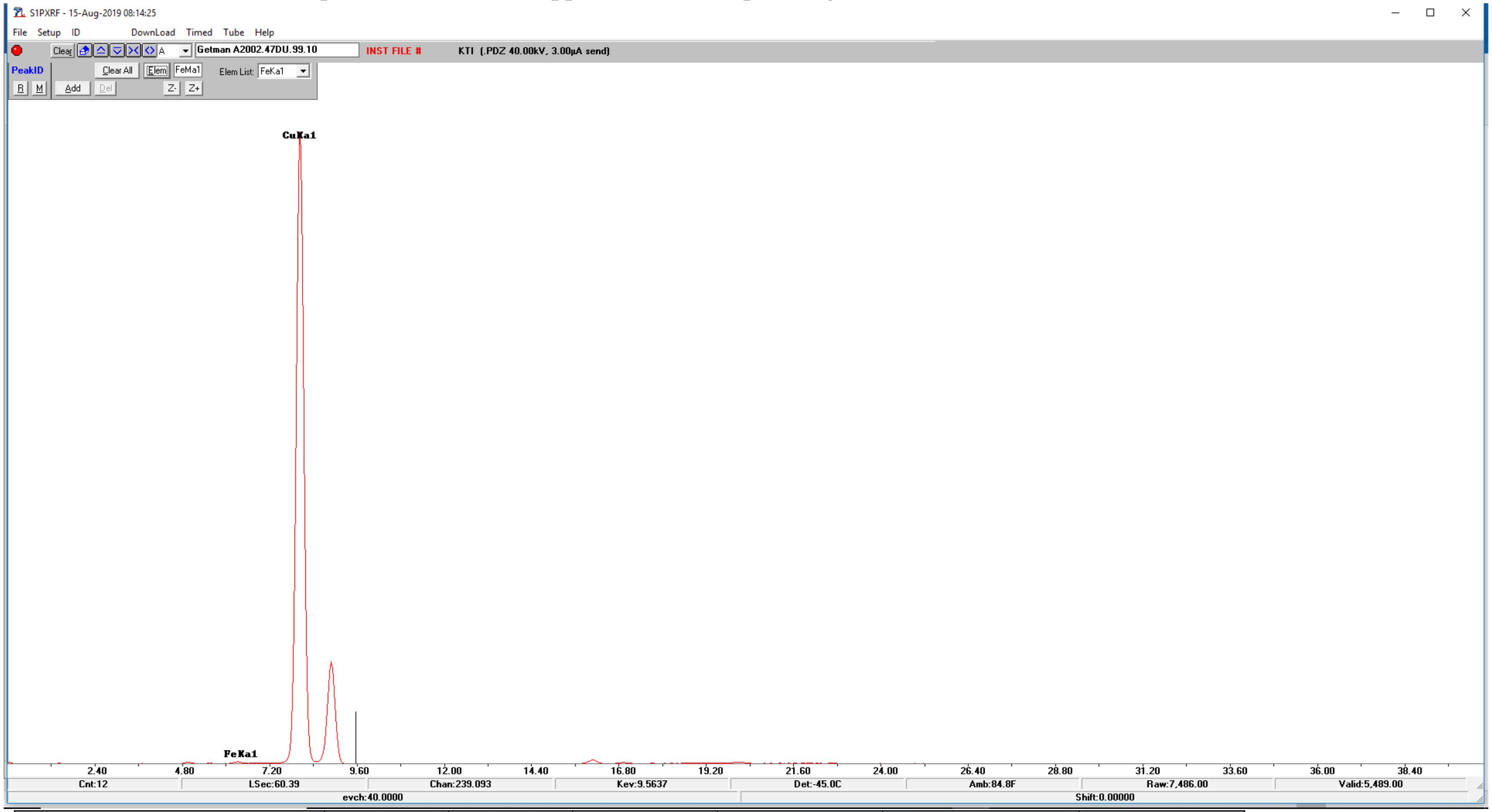


| Getman A2002.47DU.99.10 | Energy1 | Energy2 | Chan-Start | Chan-End | Chan-Counts | ROI |
|---|---|---|---|---|---|---|
| FeKa1 | 6.2347 | 6.573 | 155.8667 | 164.3253 | 838.98 | 0.003344 |
| CuKa1 | 7.8658 | 8.2298 | 196.6451 | 205.7439 | 250027.2 | 0.996656 |

Smith Pagerie A2002.32AC.7.38 native copper rolled bead

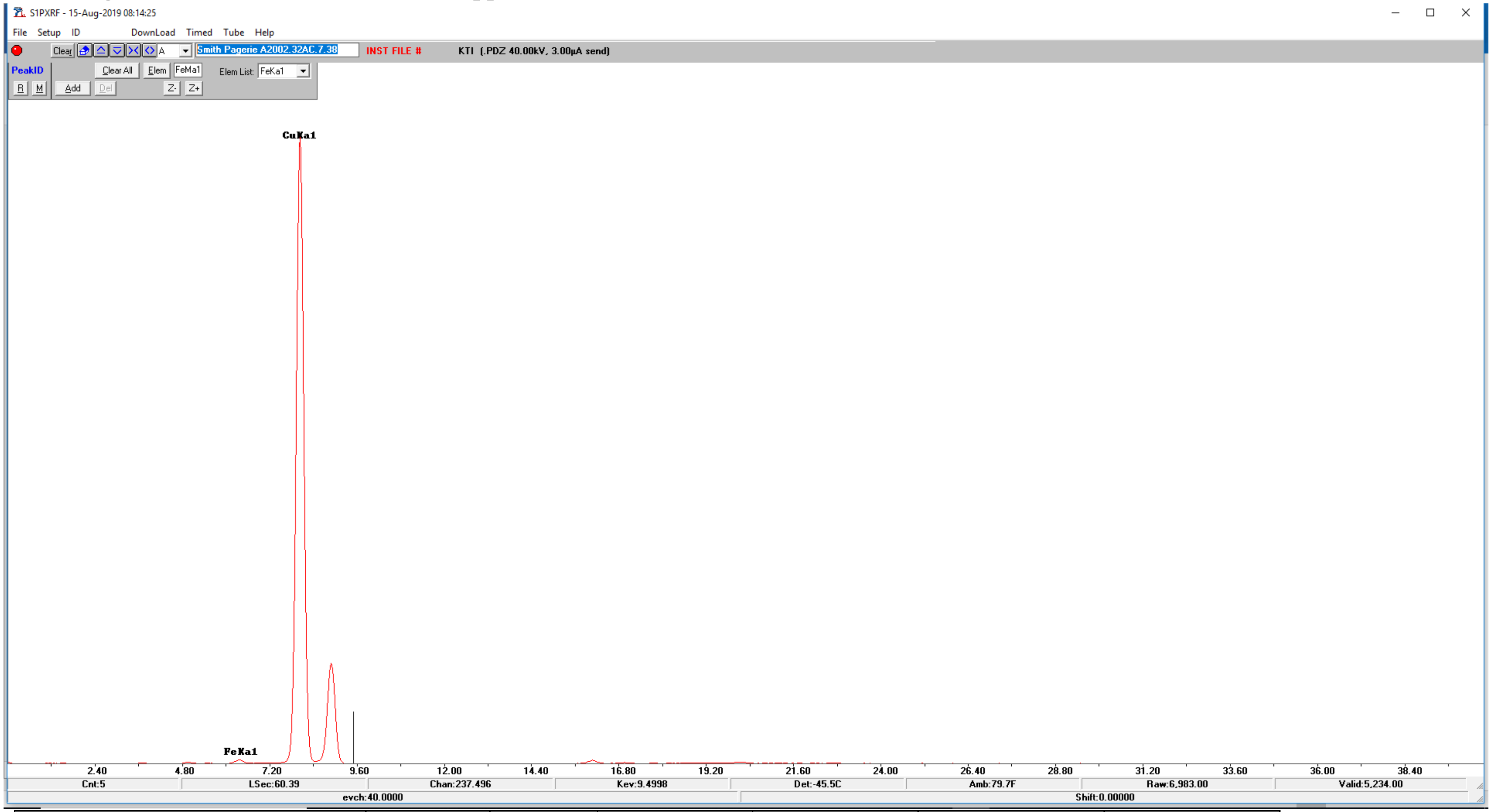


| Smith Pagerie A2002.32AC.7.38 | Energy1 | Energy2 | Chan-Start | Chan-End | Chan-Counts | ROI |
|---|---|---|---|---|---|---|
| FeKa1 | 6.2347 | 6.573 | 155.8667 | 164.3253 | 1527.8 | 0.006573 |
| CuKa1 | 7.8658 | 8.2298 | 196.6451 | 205.7439 | 230920.2 | 0.993427 |

Smith Pagerie A-44736 European copper rolled bead

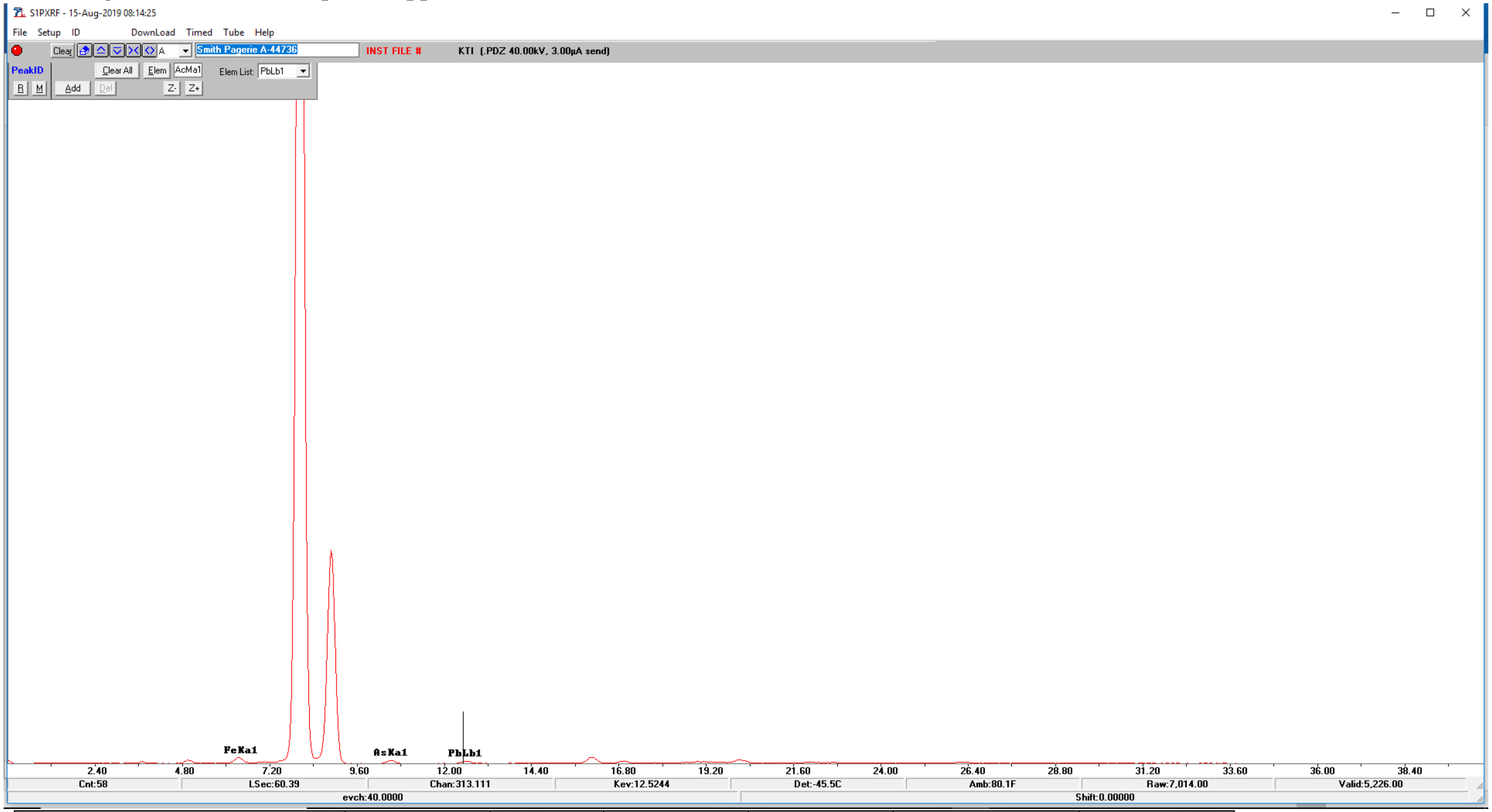


| Smith Pagerie A-44736 | Energy1 | Energy2 | Chan-Start | Chan-End | Chan-Counts | ROI |
|---|---|---|---|---|---|---|
| FeKa1 | 6.2347 | 6.573 | 155.8667 | 164.3253 | 1194.97 | 0.005165 |
| CuKa1 | 7.8658 | 8.2298 | 196.6451 | 205.7439 | 228992.3 | 0.989813 |
| AsKa1 | 10.3439 | 10.7436 | 258.5966 | 268.5894 | 614.48 | 0.002656 |
| PbLb1 | 12.4002 | 12.8272 | 310.0038 | 320.6812 | 547.38 | 0.002366 |

Garoga A-42864A European copper scrap

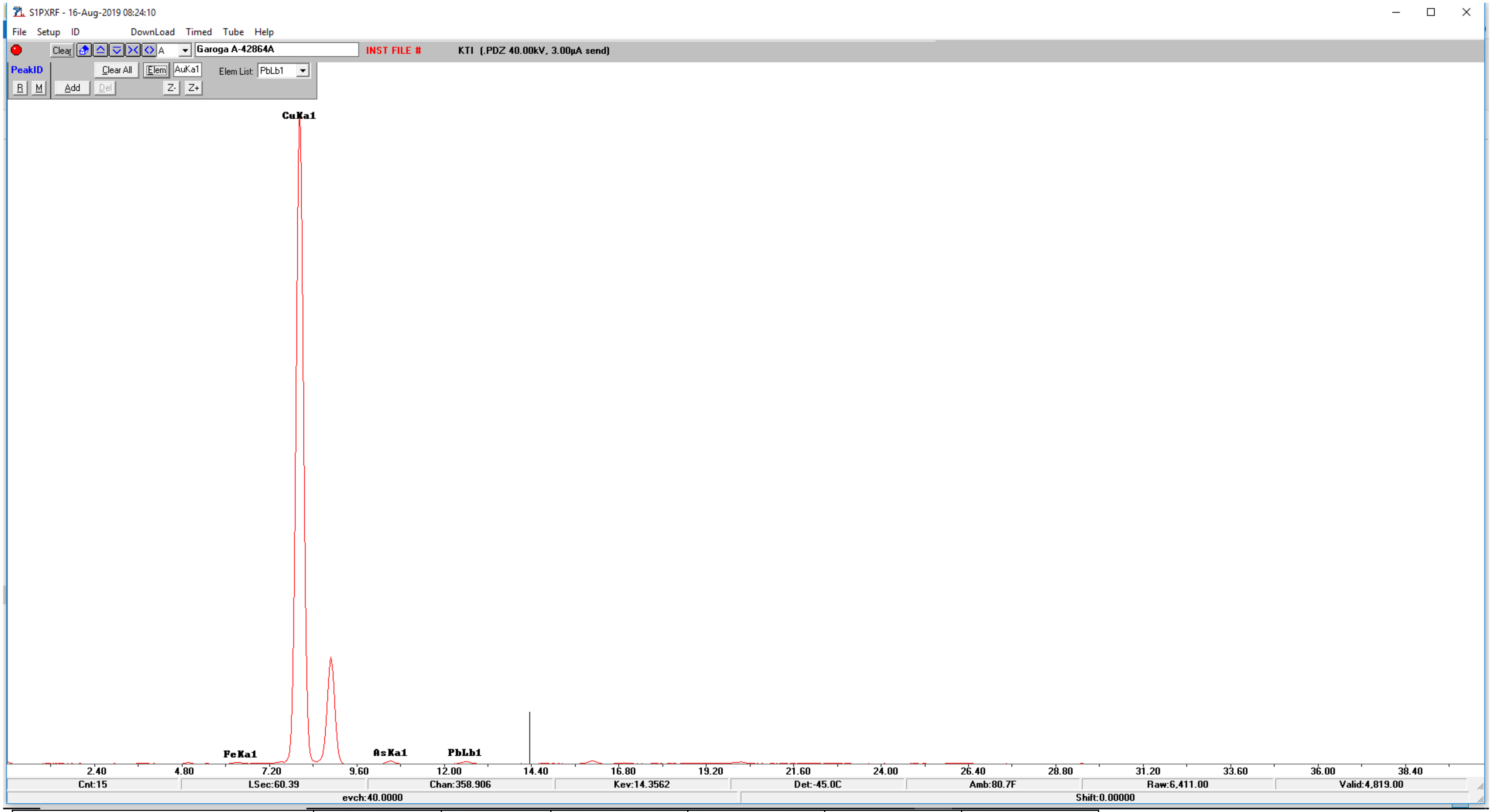


| Garoga A-42864A | Energy1 | Energy2 | Chan-Start | Chan-End | Chan-Counts | ROI |
|---|---|---|---|---|---|---|
| FeKa1 | 6.2347 | 6.573 | 155.8667 | 164.3253 | 687.52 | 0.003128 |
| CuKa1 | 7.8658 | 8.2298 | 196.6451 | 205.7439 | 216998.5 | 0.987285 |
| AsKa1 | 10.3439 | 10.7436 | 258.5966 | 268.5894 | 1095.6 | 0.004985 |
| PbLb1 | 12.4002 | 12.8272 | 310.0038 | 320.6812 | 1011.45 | 0.004602 |

Garoga A2009.07BB.99.15 European copper alloy tube/bead.

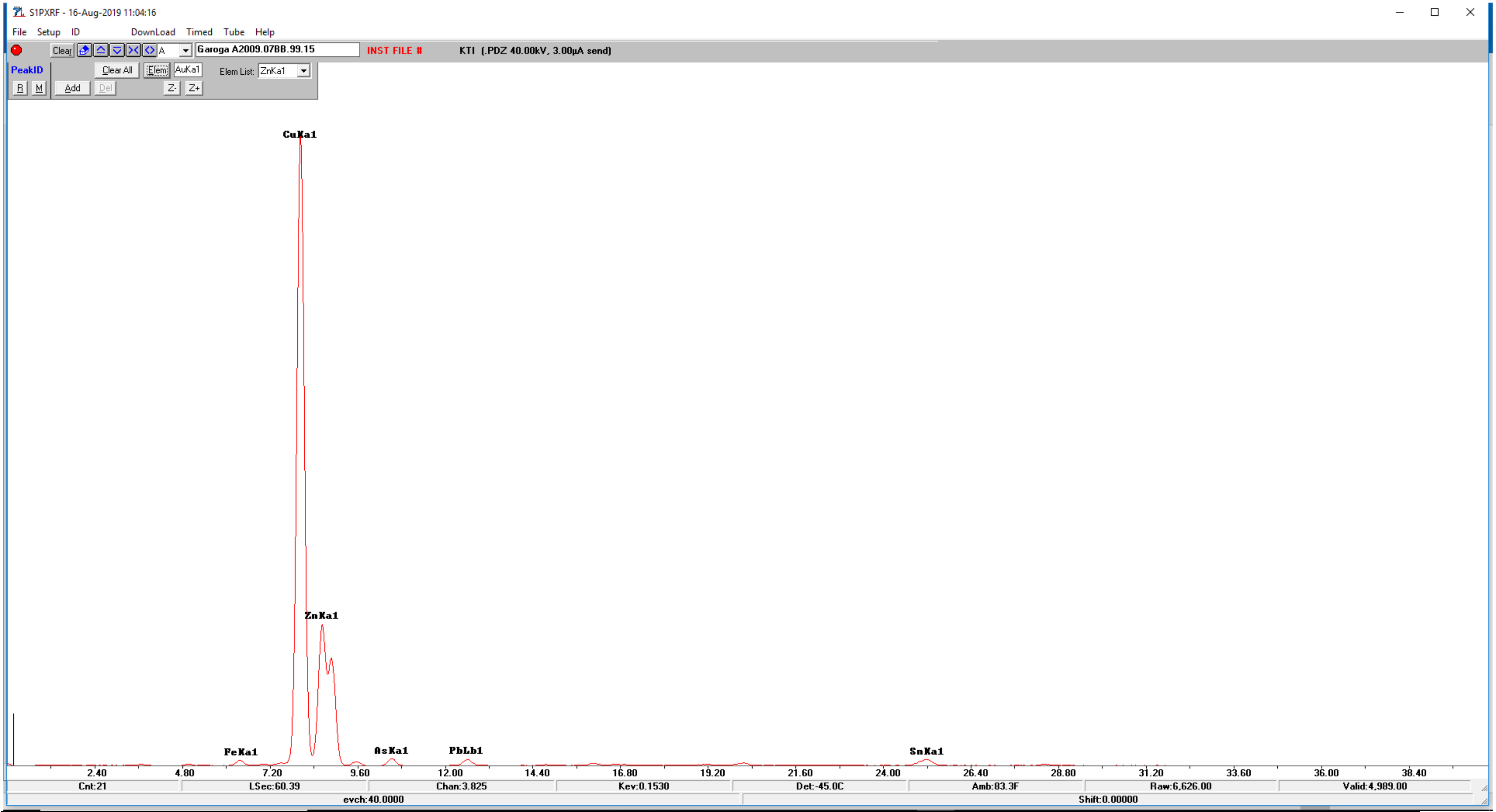


| Garoga A2009.07BB.99.15 | Energy1 | Energy2 | Chan-Start | Chan-End | Chan-Counts | ROI |
|---|---|---|---|---|---|---|
| FeKa1 | 6.2347 | 6.573 | 155.8667 | 164.3253 | 16134.61 | 0.006826 |
| CuKa1 | 7.8658 | 8.2298 | 196.6451 | 205.7439 | 1820347 | 0.770156 |
| ZnKa1 | 8.4525 | 8.8252 | 211.3124 | 220.6306 | 451098.7 | 0.190852 |
| AsKa1 | 10.3439 | 10.7436 | 258.5966 | 268.5894 | 23060.43 | 0.009756 |
| PbLb1 | 12.4002 | 12.8272 | 310.0038 | 320.6812 | 20935.04 | 0.008857 |
| SnKa1 | 24.9881 | 25.5545 | 624.7016 | 638.8634 | 32031.36 | 0.013552 |

Cayadutta European copper amulet

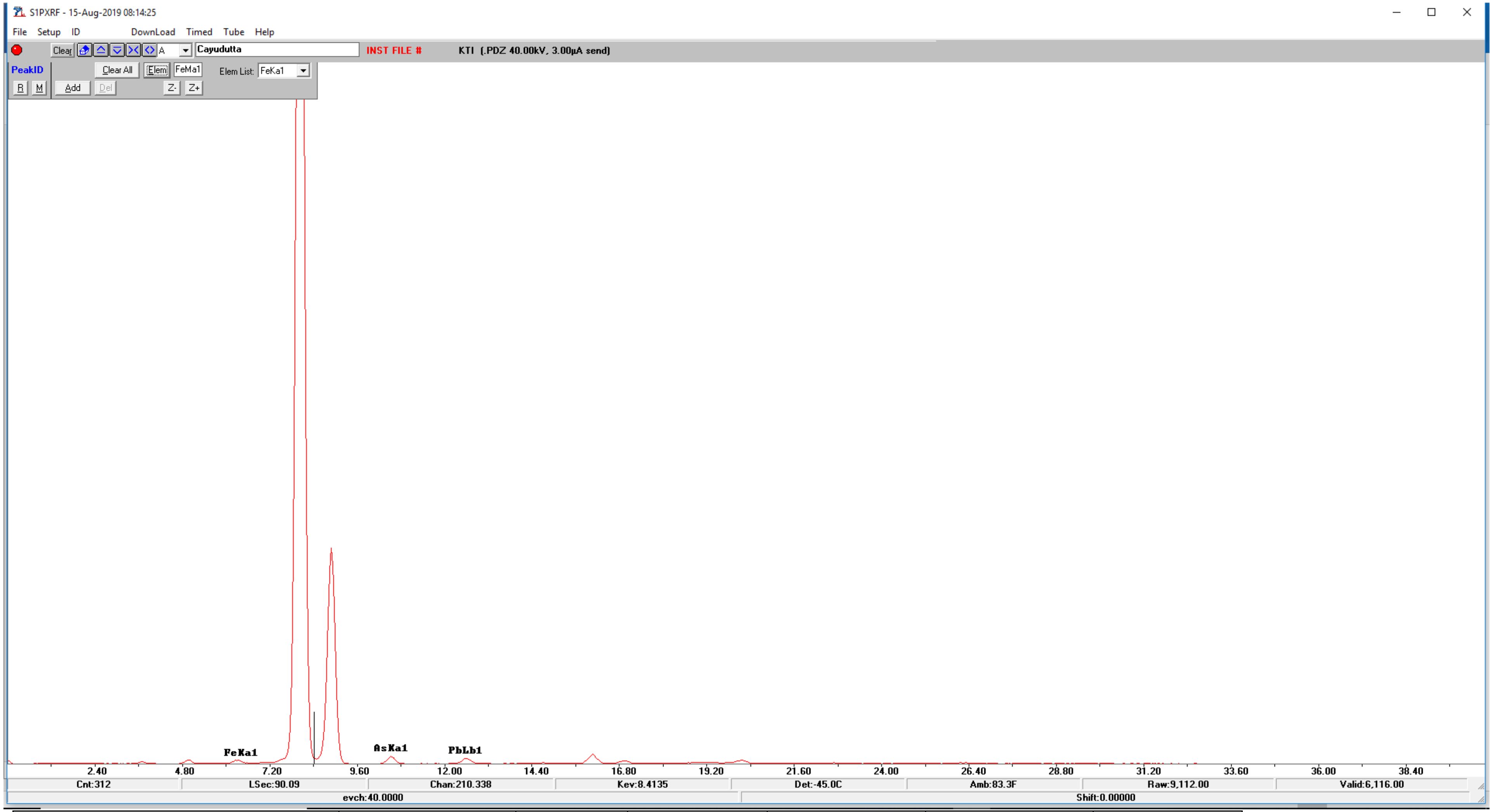


| Cayudutta | Energy1 | Energy2 | Chan-Start | Chan-End | Chan-Counts | ROI |
|---|---|---|---|---|---|---|
| FeKa1 | 6.2347 | 6.573 | 155.8667 | 164.3253 | 1306.16 | 0.003125 |
| CuKa1 | 7.8658 | 8.2298 | 196.6451 | 205.7439 | 412098.4 | 0.986099 |
| AsKa1 | 10.3439 | 10.7436 | 258.5966 | 268.5894 | 2441.42 | 0.005842 |
| PbLb1 | 12.4002 | 12.8272 | 310.0038 | 320.6812 | 2061.95 | 0.004934 |

Wormuth A2002.10CC.4.7a European copper alloy scrap

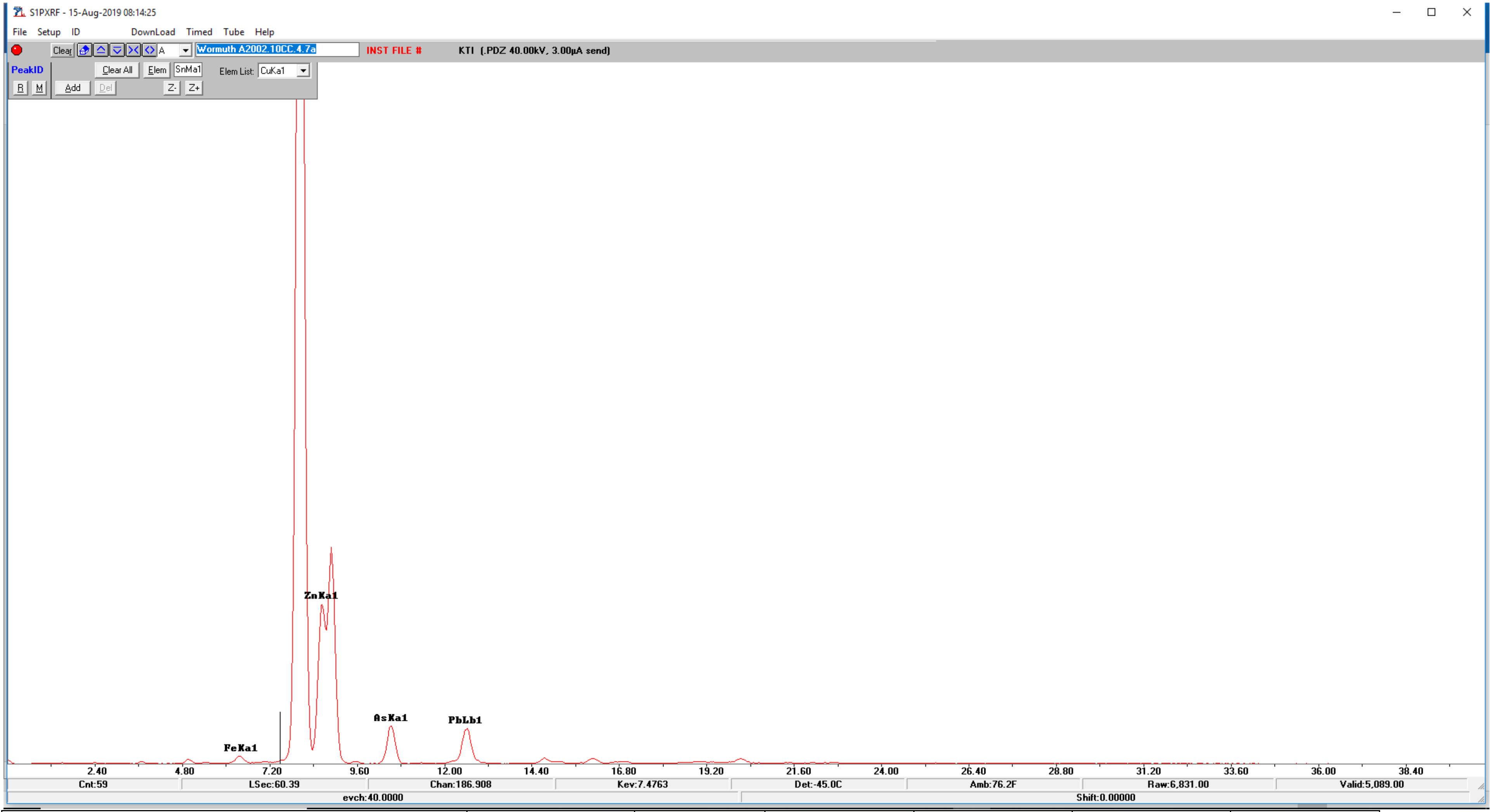


| Wormuth A2002.10CC.4.7a | Energy1 | Energy2 | Chan-Start | Chan-End | Chan-Counts | ROI |
|---|---|---|---|---|---|---|
| FeKa1 | 6.2347 | 6.573 | 155.8667 | 164.3253 | 1271.93 | 0.005389 |
| CuKa1 | 7.8658 | 8.2298 | 196.6451 | 205.7439 | 194970.5 | 0.826112 |
| ZnKa1 | 8.4525 | 8.8252 | 211.3124 | 220.6306 | 27158.47 | 0.115073 |
| AsKa1 | 10.3439 | 10.7436 | 258.5966 | 268.5894 | 6227.05 | 0.026385 |
| PbLb1 | 12.4002 | 12.8272 | 310.0038 | 320.6812 | 6381.93 | 0.027041 |

Wormuth A2002.10CC.4.7b European copper alloy scrap

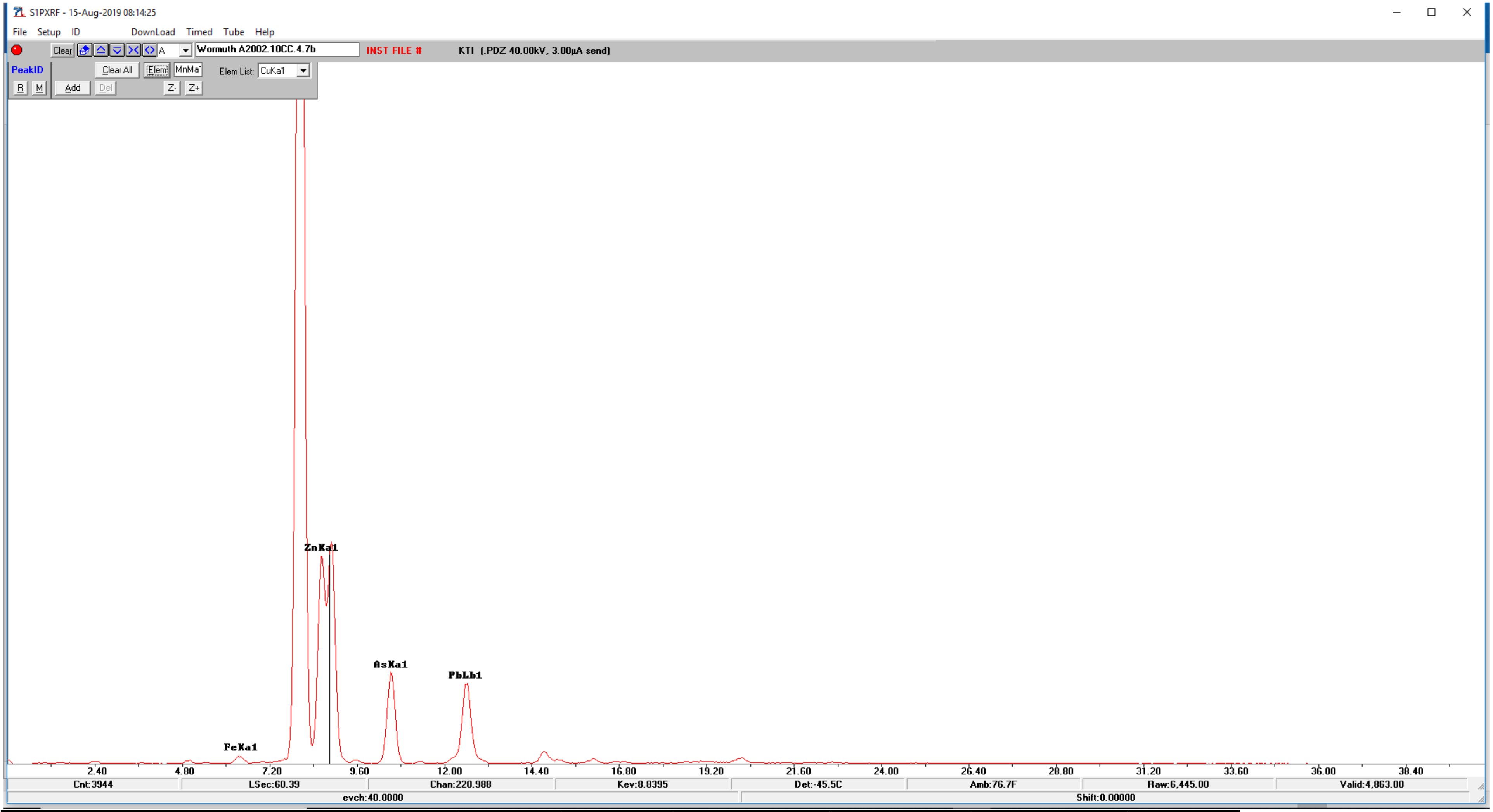


| Wormuth A2002.10CC.4.7b | Energy1 | Energy2 | Chan-Start | Chan-End | Chan-Counts | ROI |
|---|---|---|---|---|---|---|
| FeKa1 | 6.2347 | 6.573 | 155.8667 | 164.3253 | 1093.09 | 0.004854 |
| CuKa1 | 7.8658 | 8.2298 | 196.6451 | 205.7439 | 169093 | 0.750871 |
| ZnKa1 | 8.4525 | 8.8252 | 211.3124 | 220.6306 | 29885.44 | 0.132709 |
| AsKa1 | 10.3439 | 10.7436 | 258.5966 | 268.5894 | 12746.79 | 0.056603 |
| PbLb1 | 12.4002 | 12.8272 | 310.0038 | 320.6812 | 12377.38 | 0.054963 |

Wormuth A2002.10CC.4.7c European copper alloy scrap

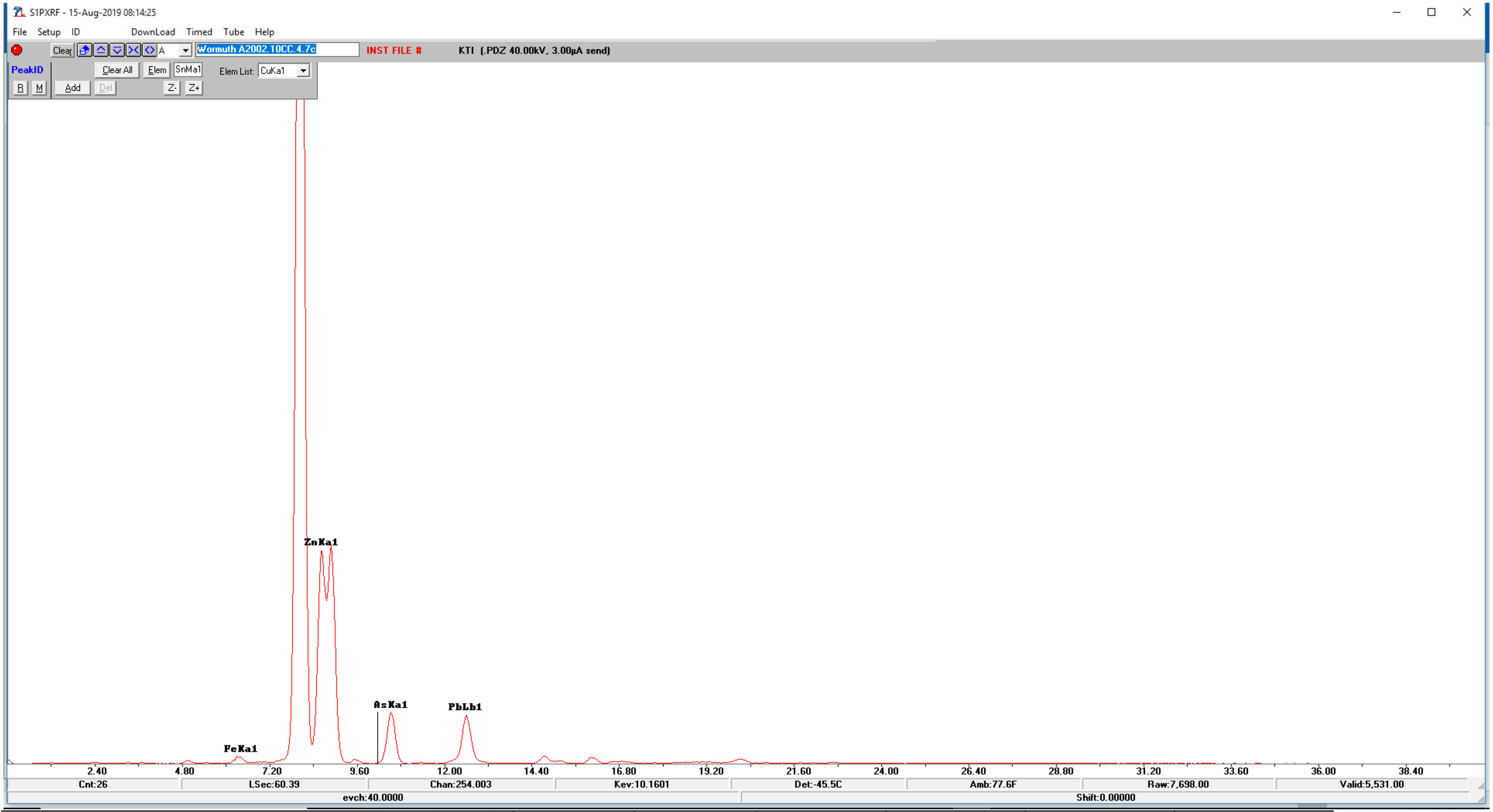


| Wormuth A2002.10CC.4.7c | Energy1 | Energy2 | Chan-Start | Chan-End | Chan-Counts | ROI |
|---|---|---|---|---|---|---|
| FeKa1 | 6.2347 | 6.573 | 155.8667 | 164.3253 | 1284.6 | 0.004909 |
| CuKa1 | 7.8658 | 8.2298 | 196.6451 | 205.7439 | 205810.5 | 0.786511 |
| ZnKa1 | 8.4525 | 8.8252 | 211.3124 | 220.6306 | 37065.57 | 0.141647 |
| AsKa1 | 10.3439 | 10.7436 | 258.5966 | 268.5894 | 8738.9 | 0.033396 |
| PbLb1 | 12.4002 | 12.8272 | 310.0038 | 320.6812 | 8775.85 | 0.033537 |

Wormuth A2002.10CC.4.7d European copper alloy scrap

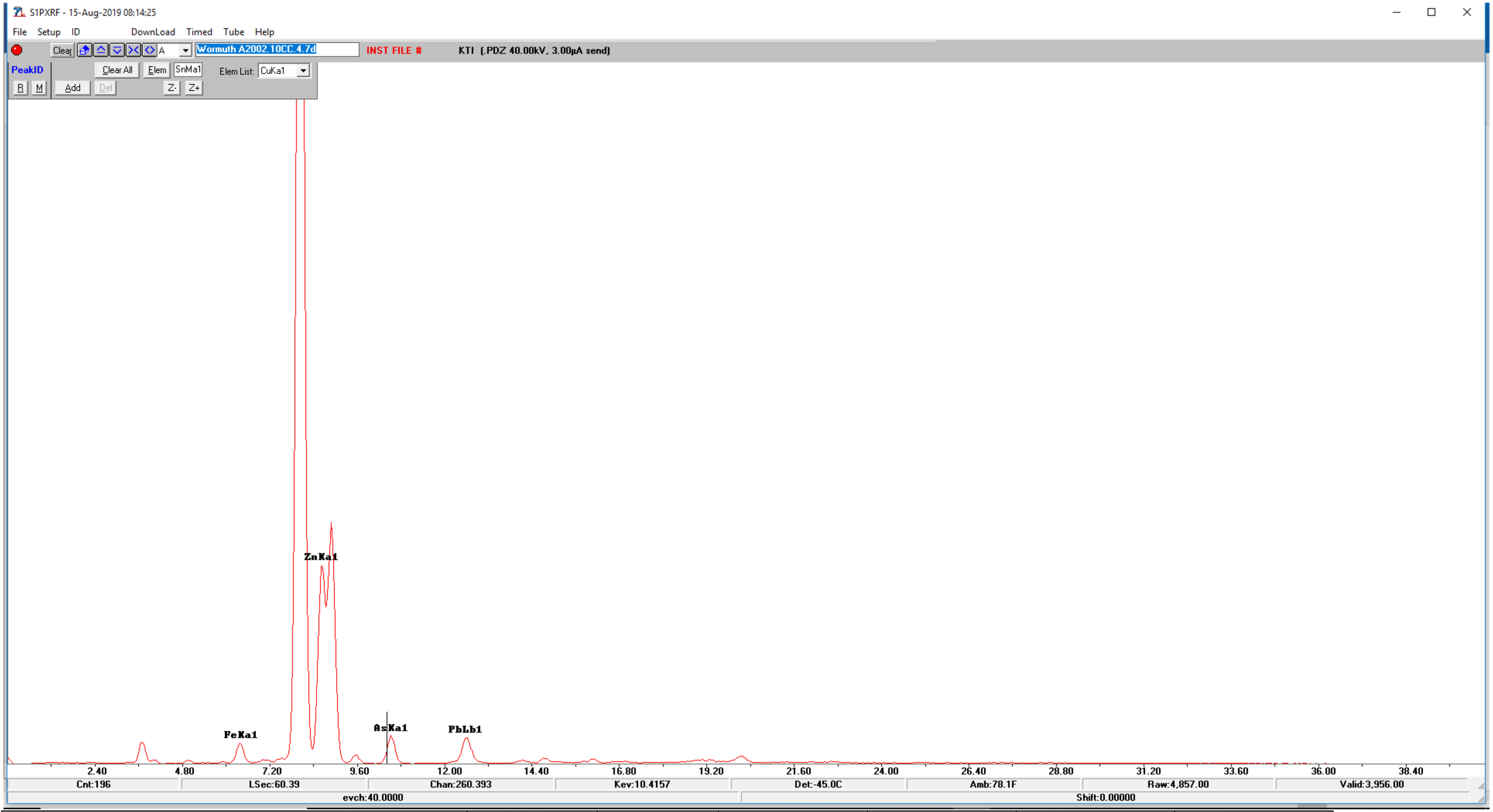


| Wormuth A2002.10CC.4.7d | Energy1 | Energy2 | Chan-Start | Chan-End | Chan-Counts | ROI |
|---|---|---|---|---|---|---|
| FeKa1 | 6.2347 | 6.573 | 155.8667 | 164.3253 | 2180.05 | 0.012841 |
| CuKa1 | 7.8658 | 8.2298 | 196.6451 | 205.7439 | 137543.1 | 0.810151 |
| ZnKa1 | 8.4525 | 8.8252 | 211.3124 | 220.6306 | 23545 | 0.138684 |
| AsKa1 | 10.3439 | 10.7436 | 258.5966 | 268.5894 | 3103.9 | 0.018282 |
| PbLb1 | 12.4002 | 12.8272 | 310.0038 | 320.6812 | 3402.56 | 0.020042 |

Briggs Run A2005.13AG.99.10a European copper alloy triangular point

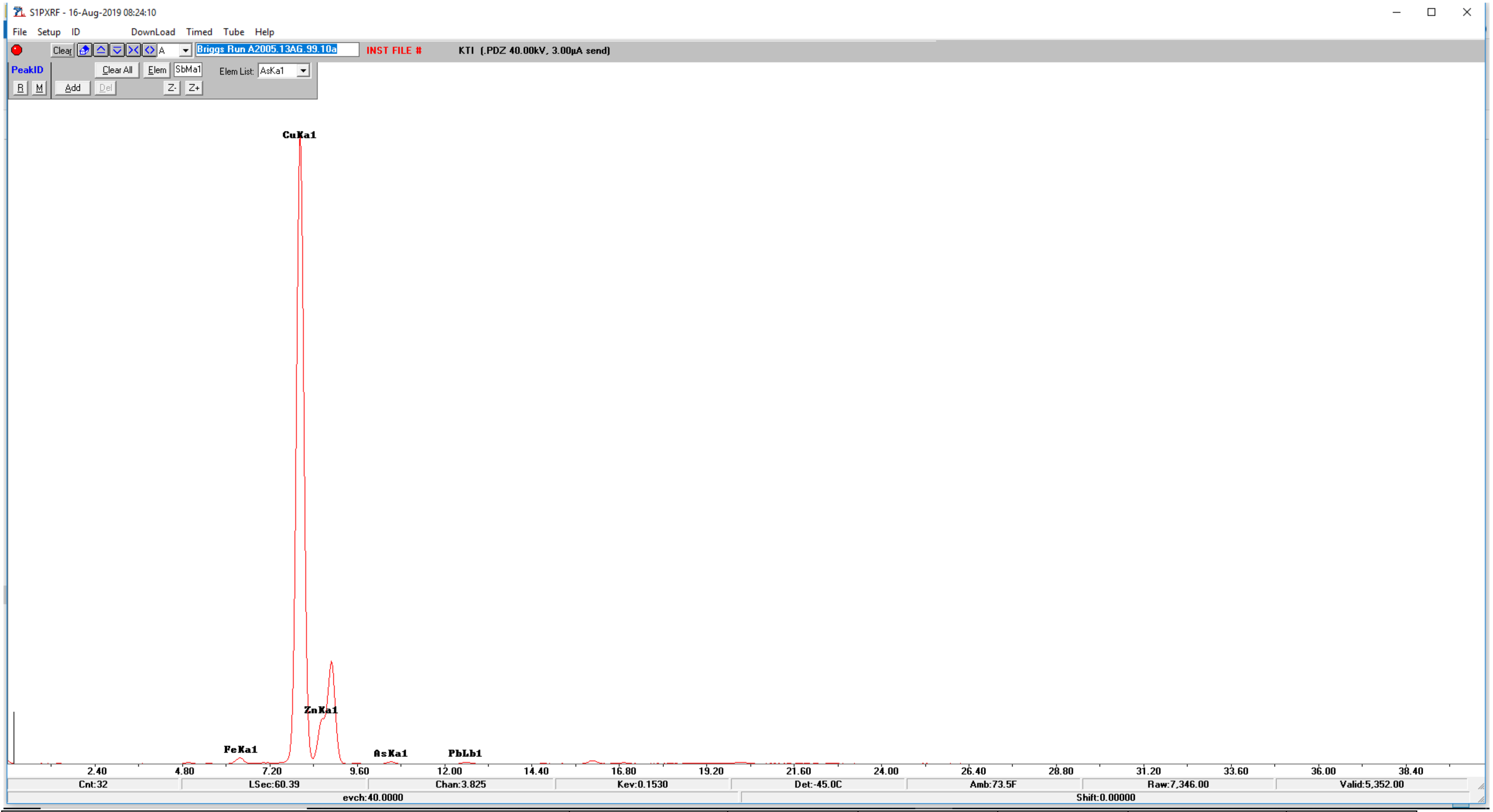


| Briggs Run A2005.13AG.99.10a | Energy1 | Energy2 | Chan-Start | Chan-End | Chan-Counts | ROI |
|---|---|---|---|---|---|---|
| FeKa1 | 6.2347 | 6.573 | 155.8667 | 164.3253 | 2350 | 0.009302 |
| CuKa1 | 7.8658 | 8.2298 | 196.6451 | 205.7439 | 228375 | 0.903992 |
| ZnKa1 | 8.4525 | 8.8252 | 211.3124 | 220.6306 | 20251.2 | 0.080162 |
| AsKa1 | 10.3439 | 10.7436 | 258.5966 | 268.5894 | 865.63 | 0.003426 |
| PbLb1 | 12.4002 | 12.8272 | 310.0038 | 320.6812 | 787.54 | 0.003117 |

Briggs Run A2005.13AG.99.10b European copper alloy triangular point

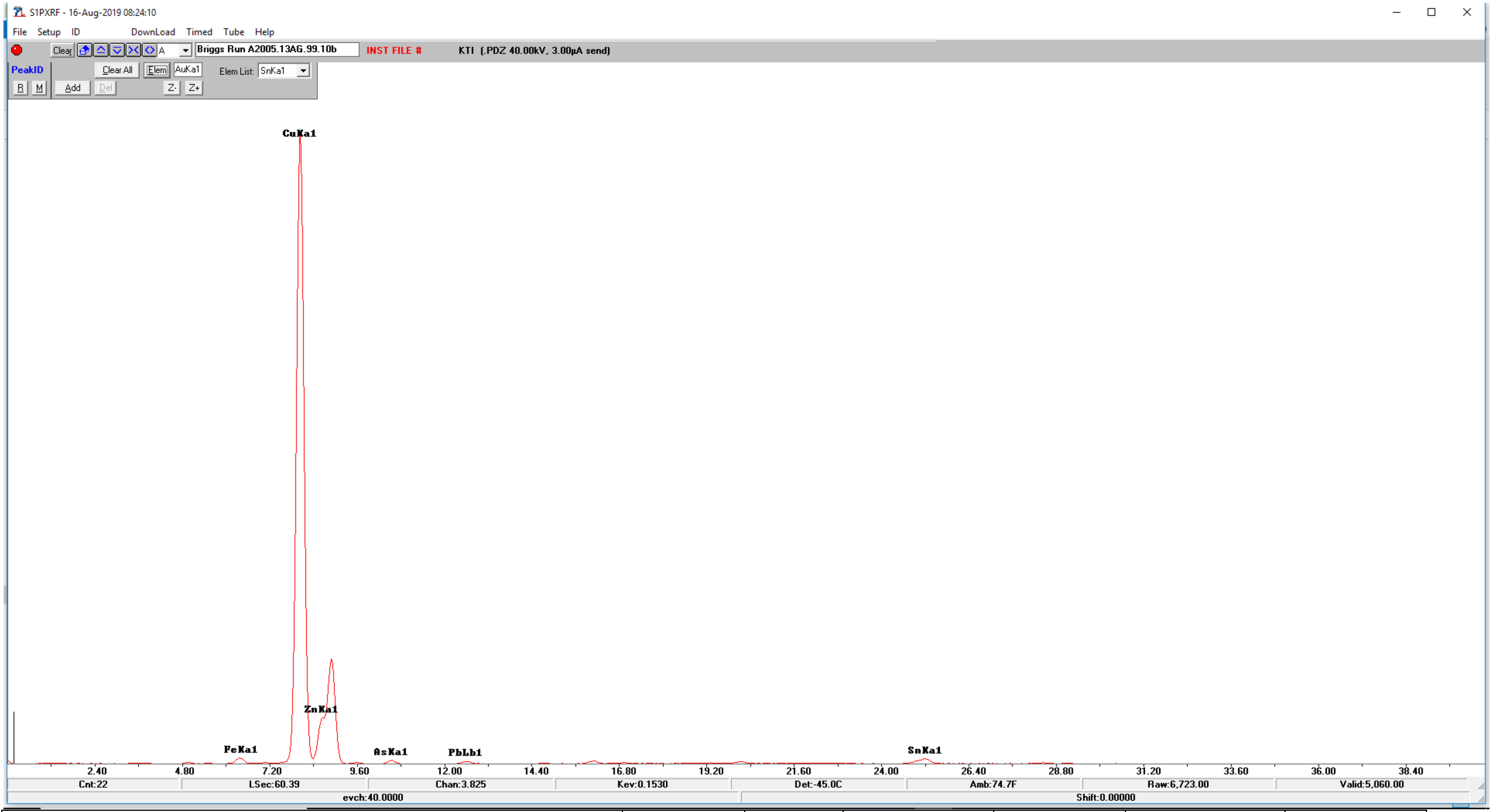


| Briggs Run A2005.13AG.99.10b | Energy1 | Energy2 | Chan-Start | Chan-End | Chan-Counts | ROI |
|---|---|---|---|---|---|---|
| FeKa1 | 6.2347 | 6.573 | 155.8667 | 164.3253 | 2069.47 | 0.00891 |
| CuKa1 | 7.8658 | 8.2298 | 196.6451 | 205.7439 | 206557.3 | 0.889363 |
| ZnKa1 | 8.4525 | 8.8252 | 211.3124 | 220.6306 | 18390.52 | 0.079183 |
| AsKa1 | 10.3439 | 10.7436 | 258.5966 | 268.5894 | 1237.36 | 0.005328 |
| PbLb1 | 12.4002 | 12.8272 | 310.0038 | 320.6812 | 1064.14 | 0.004582 |
| SnKa1 | 24.9881 | 25.5545 | 624.7016 | 638.8634 | 2934.27 | 0.012634 |

Briggs Run A2005.13AG.99.10c European copper alloy metal tinkler

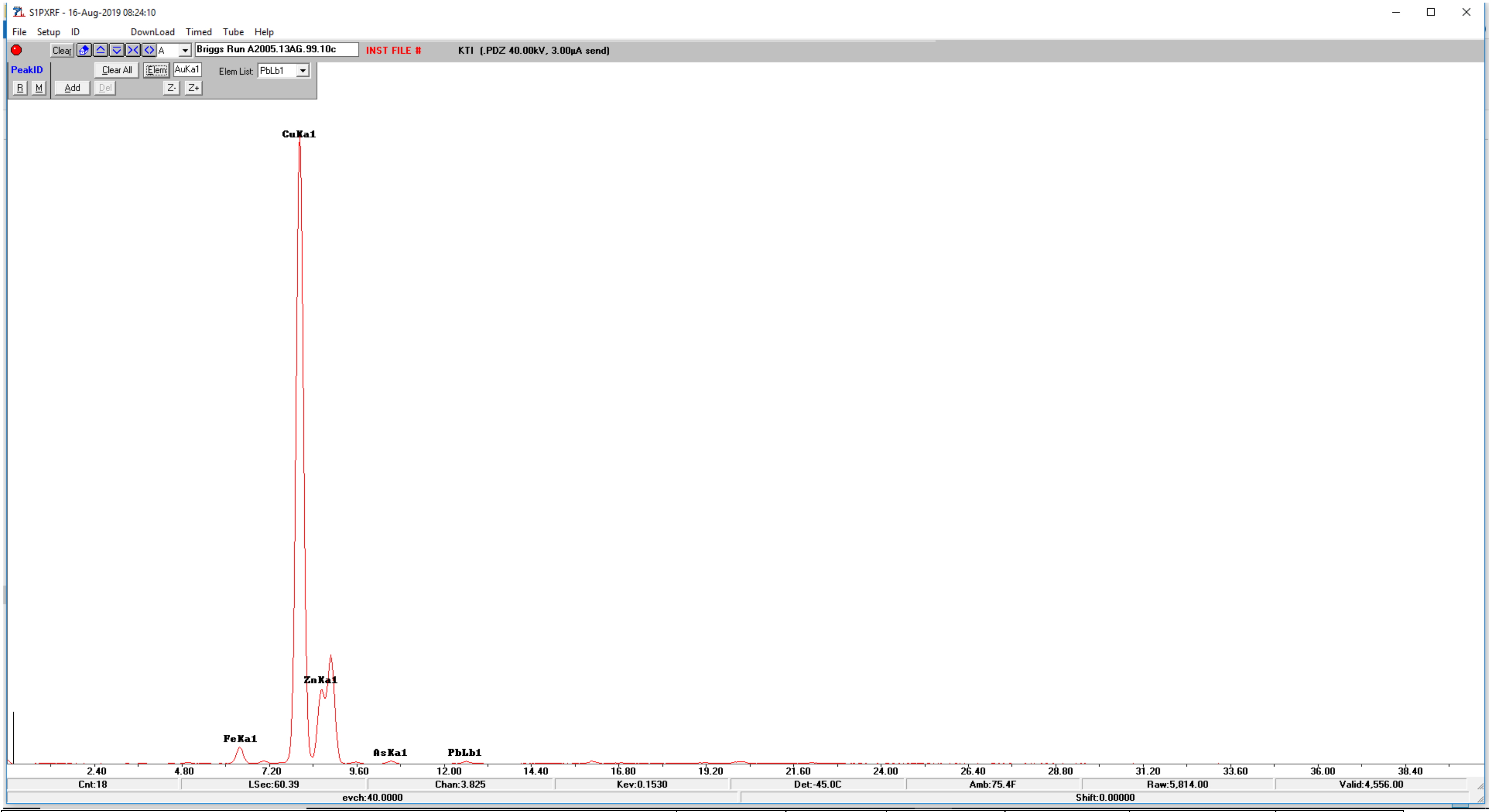


| Briggs Run A2005.13AG.99.10c | Energy1 | Energy2 | Chan-Start | Chan-End | Chan-Counts | ROI |
|---|---|---|---|---|---|---|
| FeKa1 | 6.2347 | 6.573 | 155.8667 | 164.3253 | 4636.55 | 0.021946 |
| CuKa1 | 7.8658 | 8.2298 | 196.6451 | 205.7439 | 180078.3 | 0.852371 |
| ZnKa1 | 8.4525 | 8.8252 | 211.3124 | 220.6306 | 24754.19 | 0.11717 |
| AsKa1 | 10.3439 | 10.7436 | 258.5966 | 268.5894 | 922.96 | 0.004369 |
| PbLb1 | 12.4002 | 12.8272 | 310.0038 | 320.6812 | 875.48 | 0.004144 |

Briggs Run A2005.13AG.99.10d European copper alloy metal tinkler

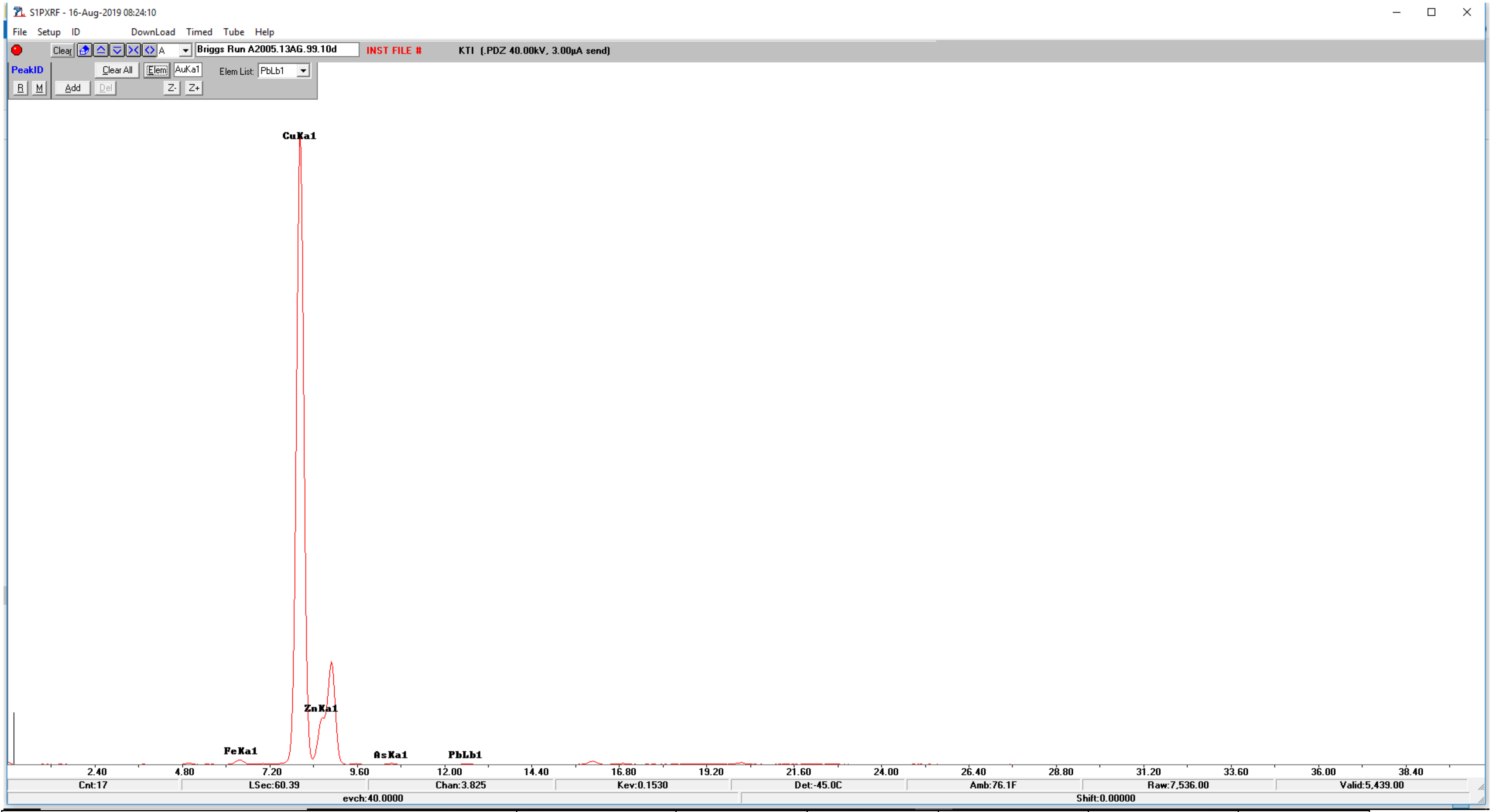


| Briggs Run A2005.13AG.99.10d | Energy1 | Energy2 | Chan-Start | Chan-End | Chan-Counts | ROI |
|---|---|---|---|---|---|---|
| FeKa1 | 6.2347 | 6.573 | 155.8667 | 164.3253 | 1958.98 | 0.007722 |
| CuKa1 | 7.8658 | 8.2298 | 196.6451 | 205.7439 | 229606.5 | 0.905048 |
| ZnKa1 | 8.4525 | 8.8252 | 211.3124 | 220.6306 | 21093 | 0.083143 |
| AsKa1 | 10.3439 | 10.7436 | 258.5966 | 268.5894 | 544.66 | 0.002147 |
| PbLb1 | 12.4002 | 12.8272 | 310.0038 | 320.6812 | 492.19 | 0.00194 |

Briggs collection A-2204 post-contact European copper triangular point, Mohawk Valley.

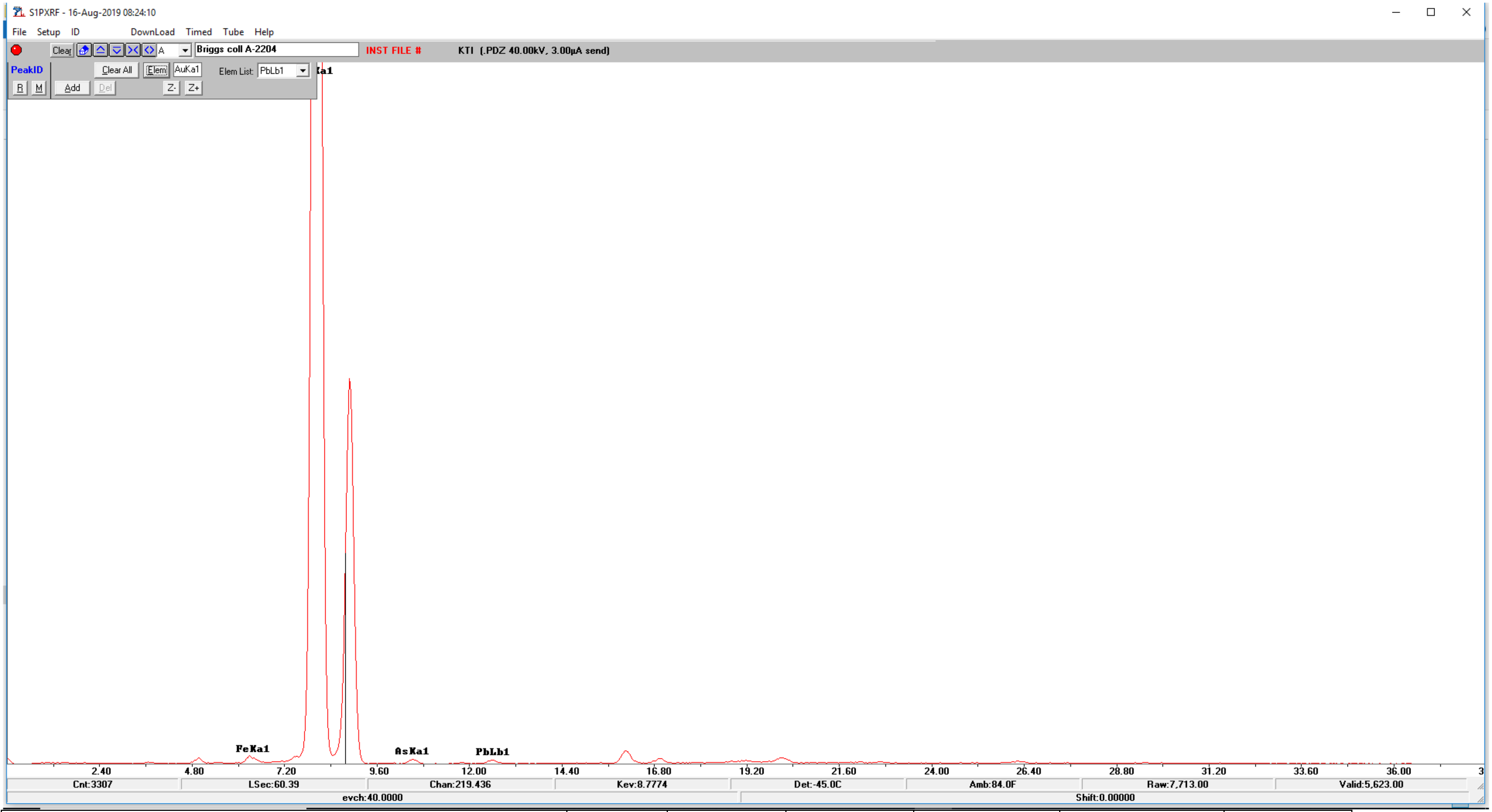


| Briggs coll A-2204 | Energy1 | Energy2 | Chan-Start | Chan-End | Chan-Counts | ROI |
|---|---|---|---|---|---|---|
| FeKa1 | 6.2347 | 6.573 | 155.8667 | 164.3253 | 924.85 | 0.003674 |
| CuKa1 | 7.8658 | 8.2298 | 196.6451 | 205.7439 | 249749.1 | 0.992186 |
| AsKa1 | 10.3439 | 10.7436 | 258.5966 | 268.5894 | 550.71 | 0.002188 |
| PbLb1 | 12.4002 | 12.8272 | 310.0038 | 320.6812 | 491.4 | 0.001952 |

Briggs collection A-2205 post-contact European copper triangular point, Mohawk Valley.

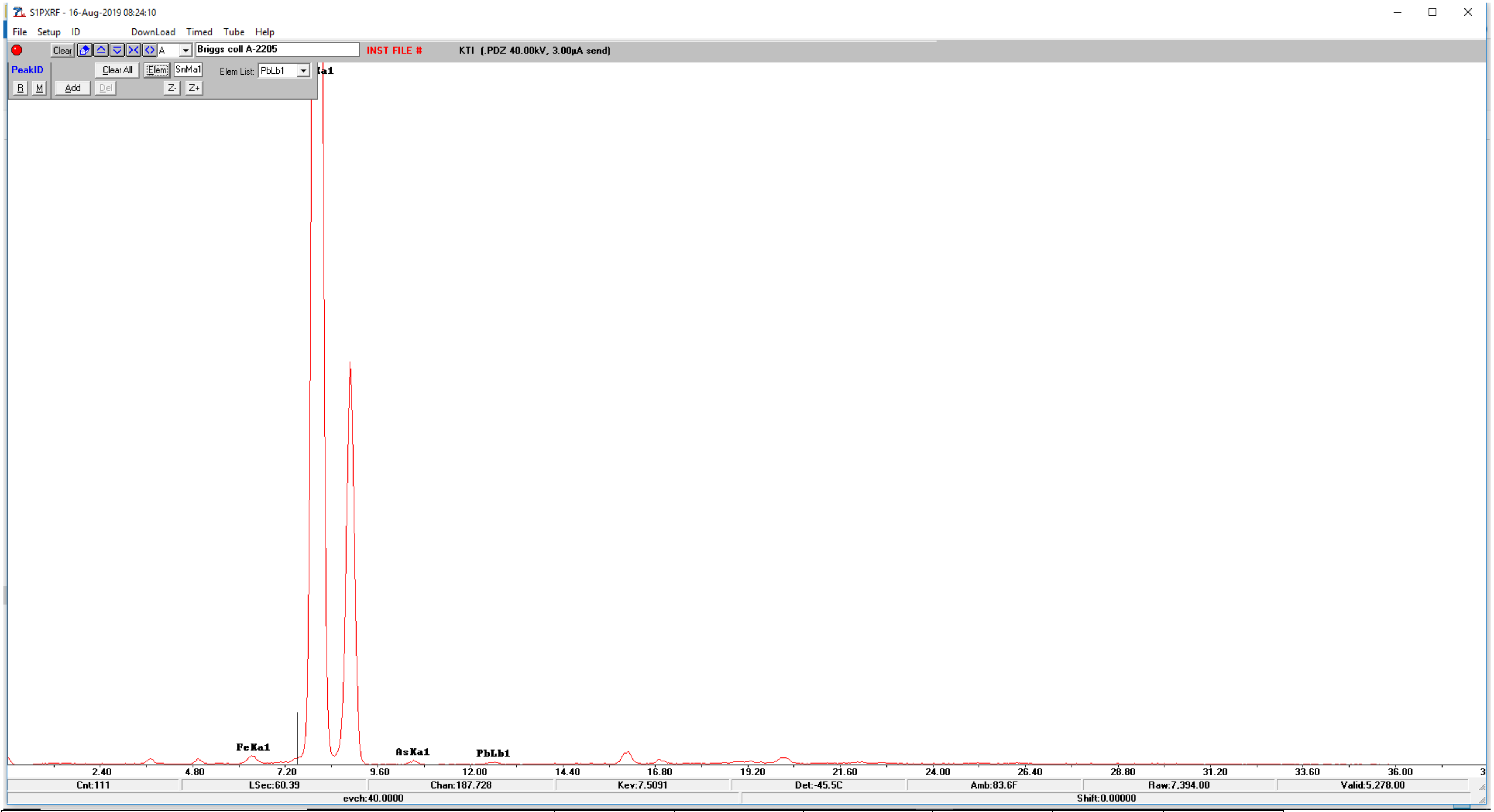


| Briggs coll A-2205 | Energy1 | Energy2 | Chan-Start | Chan-End | Chan-Counts | ROI |
|---|---|---|---|---|---|---|
| FeKa1 | 6.2347 | 6.573 | 155.8667 | 164.3253 | 1023.12 | 0.004245 |
| CuKa1 | 7.8658 | 8.2298 | 196.6451 | 205.7439 | 239171.3 | 0.992359 |
| AsKa1 | 10.3439 | 10.7436 | 258.5966 | 268.5894 | 437.15 | 0.001814 |
| PbLb1 | 12.4002 | 12.8272 | 310.0038 | 320.6812 | 381.39 | 0.001582 |
