# Supplementary material for: Radiocarbon, Bayesian chronological modeling and early European metal circulation in the sixteenth-century AD Mohawk River Valley, USA
Source: PLoS One. 2019 Dec 16;14(12):e0226334. doi: 10.1371/journal.pone.0226334 (PMC6913979; doi:10.1371/journal.pone.0226334)
Supplement: S3 File — The OxCal [23, 38] runfiles for a range of the models employed in the paper. (PDF) [file pone.0226334.s003.pdf]

S3 File. The main OxCal [23, 38] runfiles employed in the paper are listed, for Model 1, for Model 2 (0-120 years Interval constraint version), for the re-run of Model 2 including Palatine Bridge and Briggs Run, and for each of Models 3 and 3A (models 4 and 4A merely exclude the elements for Palatine Bridge and Briggs Run).

Model 1 OxCal runfile

```
Options()
 {
  Resolution=1;
 };
 Plot()
 {
  Outlier_Model("General",T(5),U(0,4),"t");
  Outlier_Model("Charcoal",Exp(1,-10,0),U(0,3),"t");
  Outlier_Model("SSimple",N(0,2),0,"s");
  Phase ()
  {
   Sequence()
   {
    Boundary("Start Snell Pits",Date(U(1150,1635)));
    Phase("Snell Pits")
    {
     R_Date("M-28 charred wood",1670,300)
     {
      Outlier("Charcoal",1);
     };
     R_Date("M-178 charred wood",1170,200)
     {
      Outlier("Charcoal",1);
     };
     R_Date("M-492 charred wood",794,200)
     {
      Outlier("Charcoal",1);
     };
     R_Date("UCIAMS190544 B",710,15)
     {
      Outlier("General",0.05);
     };
     R_Date("UCIAMS190542 B",710,20)
     {
      Outlier("General",0.05);
     };
     R_Date("UCIAMS190543 B",705,20)
     {
      Outlier("General",0.05);
     };
     R_Date("ISGS-A0327 M",691,39)
     {
      Outlier("General",0.05);
     };
     R_Date("UCIAMS192977 B",685,15)
     {
      Outlier("General",0.05);
```

```
   };
   Interval("Interval Snell");
   Date("Date Snell Pits");
  };
  Boundary("End Snell Pits",Date(U(1150,1635)));
 };
 Sequence()
 {
  Boundary("Start Pethick",Date(U(1150,1635)));
  Phase("Pethick hearths or small pits")
  {
   R_Date("Beta 199857 C",730,70)
   {
    Outlier("Charcoal",1);
   };
   R_Date("Beta 198540 C",670,90)
   {
    Outlier("Charcoal",1);
   };
   R_Date("Beta 211490 C",560,40)
   {
    Outlier("Charcoal",1);
   };
   R_Date("Beta 22779 C",560,40)
   {
    Outlier("Charcoal",1);
   };
   R_Date("UCIAMS218494 M",675,15)
   {
    Outlier("General",0.05);
   };
   R_Date("UCIAMS218495 M",670,15)
   {
    Outlier("General",0.05);
   };
   R_Date("UCIAMS218496 M",595,20)
   {
    Outlier("General",0.05);
   };
   Interval("Interval Pethick");
   Date("Date Pethick");
  };
  Boundary("End Pethick",Date(U(1150,1635)));
 };
 Sequence()
 {
  Boundary("Start Second Woods",Date(U(1150,1635)));
  Phase("Second Woods")
  {
   R_Combine("Feature 2 Shallow Pit Deposit - Assume One Event",8)
   {
    Outlier("General",0.05);
    R_Date("UCIAMS190546 B Feature 2",405,15)
    {
     Outlier("Simple",0.05);
    };
    R_Date("UCIAMS190547 B Feature 2",375,15)
```

```
   {
    Outlier("Simple",0.05);
   };
  };
  R_Date("UCIAMS190536 M",420,15)
  {
   Outlier("General",0.05);
  };
  R_Date("UCIAMS190535 M",380,20)
  {
   Outlier("General",0.05);
  };
  Interval("Interval Second Woods");
  Date("Date Second Woods");
 };
 Boundary("End Second Woods",Date(U(1150,1635)));
};
Sequence()
{
 Boundary("Start Elwood",Date(U(1150,1635)));
 Phase ("Elwood")
 {
  R_Date("UCIAMS190554 B Pit",420,20)
  {
   Outlier("General",0.05);
  };
  R_Date("AA-7410 M midden",409,49)
  {
   Outlier("General",0.05);
  };
  R_Date("AA-6425 M midden",380,50)
  {
   Outlier("General",0.05);
  };
  R_Date("UCIAMS190552 B Hearth", 380, 15)
  {
   Outlier("General",0.05);
  };
  R_Date("UCIAMS190553 B Hearth", 370, 20)
  {
   Outlier("General",0.05);
  };
  R_Date("AA-7697 M midden",288,49)
  {
   Outlier("General",0.05);
  };
  Interval("Interval Elwood");
  Date("Date Elwood");
 };
 Boundary("End Elwood",Date(U(1150,1635)));
};
Sequence()
{
 Boundary ("Start Getman",Date(U(1150,1635)));
 Phase("Getman")
 {
  R_Date("M-783 Charcoal context not stated TPQ",560,150)
```

```
   {
    Outlier("Charcoal",1);
   };
   R_Date("UCIAMS190557 House 3 Hearth B",450,20)
   {
    Outlier("General",0.05);
   };
   R_Combine("Feature 28 Pit Event",8)
   {
    Outlier("General",0.05);
    R_Date("UCIAMS190558 House 3 B Pit",400,15)
    {
     Outlier("Simple",0.05);
    };
    R_Date("UCIAMS218481 House 3 M Pit",395,15)
    {
     Outlier("Simple",0.05);
    };
   };
   R_Date("UCIAMS218482 General Midden M",405,15)
   {
    Outlier("General",0.05);
   };
   R_Date("UCIAMS218480 House 5 Pit M",400,15)
   {
    Outlier("General",0.05);
   };
   R_Date("UCIAMS192976 House 1 Pit B",485,15)
   {
    Outlier("General",0.05);
   };
   R_Date("UCIAMS192975 Pit B",355,15)
   {
    Outlier("General",0.05);
   };
   R_Date("UCIAMS190555 Pit B",345,20)
   {
    Outlier("General",0.05);
   };
   R_Date("UCIAMS190556 Pit B",330,20)
   {
    Outlier("General",0.05);
   };
   Interval("Interval Getman");
   Date("Date Getman");
  };
  Boundary("End Getman",Date(U(1150,1635)));
 };
 Sequence()
 {
  Boundary("Start Smith-Pagerie",Date(U(1150,1635)));
  Phase("Smith-Pagerie")
  {
   R_Combine("Feature 54 Pit H1 - assume one event",8)
   {
    Outlier("General",0.05);
    R_Date("AA-7405 M",430,50)
```

```
    {
     Outlier("SSimple",0.05);
    };
   R_Date("AA-6419 M",405,50)
    {
     Outlier("SSimple",0.05);
    };
   R_Date("UCIAMS218490 M",325,30)
    {
     Outlier("SSimple",0.05);
    };
   };
  R_Date("ISGS-A0528 R H1",445,40)
   {
    Outlier("Charcoal",1);
   };
  R_Date("UCIAMS190566 B H1",375,15)
   {
    Outlier("General",0.05);
   };
  R_Date("UCIAMS190565 B H1",370,15)
   {
    Outlier("General",0.05);
   };
  R_Date("UCIAMS190563 B H1",360,20)
   {
    Outlier("General",0.05);
   };
  R_Date("UCIAMS218491 Material?? H1",265,15)
   {
    Outlier("General",0.05);
   };
  R_Date("UCIAMS218493 M Longhouse 5 Pit",480,15)
   {
    Outlier("General",0.05);
   };
  R_Date("UCIAMS218492 M Longhouse 2 Hearth",350,15)
   {
    Outlier("General",0.05);
   };
  R_Date("UCIAMS190564 B Longhouse 4 Hearth",345,20)
   {
    Outlier("General",0.05);
   };
  Interval("Interval Smith-Pagerie");
  Date("Date Smith-Pagerie");
 };
 Boundary("End Smith-Pagerie",Date(U(1150,1635)));
};
Sequence()
{
 Boundary("Start Otstungo",Date(U(1150,1635)));
 Phase("Otstungo House 1 Midden and Hearths")
 {
  R_Date("UCIAMS190551 Hearth B",355,20)
  {
   Outlier("General",0.05);
```

```
  };
  R_Date("UCIAMS190549 Hearth B",335,20)
  {
   Outlier("General",0.05);
  };
  R_Date("UCIAMS190550 Hearth B",310,15)
  {
   Outlier("General",0.05);
  };
  R_Date("UCIAMS190548 Hearth B",310,15)
  {
   Outlier("General",0.05);
  };
  R_Date("AA-7400 M",415,50)
  {
   Outlier("General",0.05);
  };
  R_Date("AA-7402 M",410,50)
  {
   Outlier("General",0.05);
  };
  R_Date("AA-6423 M",400,55)
  {
   Outlier("General",0.05);
  };
  R_Date("UCIAMS218483 M",400,15)
  {
   Outlier("General",0.05);
  };
  R_Date("AA-7398 M",380,55)
  {
   Outlier("General",0.05);
  };
  R_Date("UCIAMS218489 M",380,20)
  {
   Outlier("General",0.05);
  };
  R_Date("AA-7401 M",365,50)
  {
   Outlier("General",0.05);
  };
  R_Date("AA-7399 M",345,55)
  {
   Outlier("General",0.05);
  };
  R_Date("UCIAMS218487 M",340,15)
  {
   Outlier("General",0.05);
  };
  Interval("Interval Otstungo");
  Date("Date Otstungo");
 };
 Boundary("End Otstungo",Date(U(1150,1635)));
};
Sequence()
{
 Boundary("Start Klock",Date(U(1150,1635)));
```

```
Phase("Klock")
{
 R_Combine("Feature 84 Pit H1 - assume one event")
 {
  Outlier("General",0.05);
  R_Date("UCIAMS218474 M",365,15)
  {
   Outlier("SSimple",0.05);
  };
  R_Date("ISGS-A0326 M", 317, 38)
  {
   Outlier("SSimple",0.05);
  };
 };
 R_Date("ISGS-A0523 R Pit",480,40)
 {
  Outlier("Charcoal",1);
 };
 R_Date("UCIAMS218476 M Pit",365,15)
 {
  Outlier("General",0.05);
 };
 R_Date("UCIAMS190559 B Pit",360,15)
 {
  Outlier("General",0.05);
 };
 R_Date("UCIAMS190561 B Pit",335,15)
 {
  Outlier("General",0.05);
 };
 R_Date("UCIAMS218475 M Pit",335,20)
 {
  Outlier("General",0.05);
 };
 R_Date("UCIAMS218473 B Pit",325,15)
 {
  Outlier("General",0.05);
 };
 R_Date("UCIAMS190562 B Hearth H1",355,20)
 {
  Outlier("General",0.05);
 };
 R_Date("UCIAMS190560 M Hearth H1",325,15)
 {
  Outlier("General",0.05);
 };
 R_Date("AA-6418 M Hearth H7", 315, 60)
 {
  Outlier("General",0.05);
 };
 R_Date("AA-7404 M Hearth H7",520,75)
 {
  Outlier("General",0.05);
 };
 Interval("Interval Klock");
 Date("Date Klock");
};
```

```
 Boundary("End Klock",Date(U(1150,1635)));
};
Sequence()
{
 Boundary("Start Cayadutta Midden",Date(U(1150,1635)));
 Phase("Cayadutta Midden")
 {
  R_Date("AA-7689 M", 557, 58)
  {
   Outlier("General",0.05);
  };
  R_Date("AA-7690 M", 415, 56)
  {
   Outlier("General",0.05);
  };
  R_Date("AA-7407 M", 367, 52)
  {
   Outlier("General",0.05);
  };
  R_Date("AA-6421 M", 300, 50)
  {
   Outlier("General",0.05);
  };
  R_Date("UCIAMS-205965 M", 355, 20)
  {
   Outlier("General",0.05);
  };
  R_Date("UCIAMS-205966 M", 340, 15)
  {
   Outlier("General",0.05);
  };
  R_Date("UCIAMS-205967 M", 315, 15)
  {
   Outlier("General",0.05);
  };
  R_Date("UCIAMS-205968 M", 375, 15)
  {
   Outlier("General",0.05);
  };
  Interval("Interval Cayadutta Midden");
  Date("Date Cayadutta Midden");
 };
 Boundary("End Cayadutta Midden",Date(U(1150,1635)));
};
Sequence()
{
 Boundary("Start Garoga",Date(U(1150,1635)));
 Phase("Garoga")
 {
  R_Date("Y-1381 charred wood",620,100)
  {
   Outlier("Charcoal",1);
  };
  R_Combine("Feature 2 Pit H9",8)
  {
   Outlier("General",0.05);
   R_Date("AA-7695 M F2 Pit H9",431,39)
```

```
    {
     Outlier("SSimple",0.05);
    };
    R_Date("AA-7403 M F2 Pit H9",410,60)
    {
     Outlier("SSimple",0.05);
    };
    R_Date("UCIAMS218478 M F2 Pit H9",345,15)
    {
     Outlier("SSimple",0.05);
    };
    R_Date("UCIAMS190537 M F2 Pit H9",335,20)
    {
     Outlier("SSimple",0.05);
    };
    R_Date("AA-6417 M F2 Pit H9",300,50)
    {
     Outlier("SSimple",0.05);
    };
   };
   R_Date("AA-8370 M H9 Pit",585,40)
   {
    Outlier("General",0.05);
   };
   R_Date("ISGS-A0522 R H5 Pit",425,40)
   {
    Outlier("Charcoal",1);
   };
   R_Date("UCIAMS190540 M H4 Pit",345,20)
   {
    Outlier("General",0.05);
   };
   R_Date("UCIAMS190539 M H2 Pit",320,20)
   {
    Outlier("General",0.05);
   };
   R_Date("UCIAMS218479 M H12 Pit",315,15)
   {
    Outlier("General",0.05);
   };
   R_Combine("F184 Pit btw H1&Stockade",8)
   {
    Outlier("General",0.05);
    R_Date("UCIAMS190538 M F184 Pit",305,20)
    {
     Outlier("SSimple",0.05);
    };
    R_Date("UCIAMS218477 M F184 Pit",330,20)
    {
     Outlier("SSimple",0.05);
    };
   };
   Interval("Interval Garoga");
   Date("Date Garoga");
  };
  Boundary("End Garoga",Date(U(1150,1635)));
 };
```

```
   Sequence()
   {
    Boundary("Start Wormuth",Date(U(1150,1635)));
    Phase("Wormuth")
    {
     R_Date("AA-6416 M",385,50)
     {
      Outlier("General",0.05);
     };
     R_Date("UCIAMS192700 M Pit",360,15)
     {
      Outlier("General",0.05);
     };
     R_Date("AA-6065 M",340,25)
     {
      Outlier("General",0.05);
     };
     R_Date("DIC-1176 Lowest Level Midden Charcoal",560,50)
     {
      Outlier("Charcoal",1);
     };
     R_Date("DIC-1177 Higher Up Midden Charcoal",250,50)
     {
      Outlier("Charcoal",1);
     };
     R_Date("DIC-1178 Fill of Burial #2",410,55)
     {
      Outlier("Charcoal",1);
     };
     Interval("Interval Wormuth");
     Date("Date Wormuth");
    };
    Boundary("End Wormuth",Date(U(1150,1635)));
   };
  };
 };
```

Model 2 (version with uniform probability 0-120 years Interval query constraint applied to each site Phase). Outliers not included are indicated by the // notation. See text. Note: the order of Elwood and Getman are changed in this runfile versus the Model 1 runfile. This does not affect the analysis, but allows the site Phases to list in the apparent order subsequently identified in Table 4.

```
Options()
 {
  Resolution=1;
 };
 Plot()
 {
  Outlier_Model("General",T(5),U(0,4),"t");
  Outlier_Model("Charcoal",Exp(1,-10,0),U(0,3),"t");
  Outlier_Model("SSimple",N(0,2),0,"s");
  Phase ()
  {
```

```
Sequence()
{
 Boundary("Start Snell Pits",Date(U(1150,1635)));
 Phase("Snell Pits")
 {
  R_Date("M-28 charred wood",1670,300)
  {
   Outlier("Charcoal",1);
  };
  R_Date("M-178 charred wood",1170,200)
  {
   Outlier("Charcoal",1);
  };
  R_Date("M-492 charred wood",794,200)
  {
   Outlier("Charcoal",1);
  };
  R_Date("UCIAMS190544 B",710,15)
  {
   Outlier("General",0.05);
  };
  R_Date("UCIAMS190542 B",710,20)
  {
   Outlier("General",0.05);
  };
  R_Date("UCIAMS190543 B",705,20)
  {
   Outlier("General",0.05);
  };
  R_Date("ISGS-A0327 M",691,39)
  {
   Outlier("General",0.05);
  };
  R_Date("UCIAMS192977 B",685,15)
  {
   Outlier("General",0.05);
  };
  Interval("Interval Snell",U(0,120));
  Date("Date Snell Pits");
 };
 Boundary("End Snell Pits",Date(U(1150,1635)));
};
Sequence()
{
 Boundary("Start Pethick",Date(U(1150,1635)));
 Phase("Pethick hearths or small pits")
 {
  R_Date("Beta 199857 C",730,70)
  {
   Outlier("Charcoal",1);
  };
  R_Date("Beta 198540 C",670,90)
  {
   Outlier("Charcoal",1);
  };
  R_Date("Beta 211490 C",560,40)
  {
```

```
   Outlier("Charcoal",1);
  };
  R_Date("Beta 22779 C",560,40)
  {
   Outlier("Charcoal",1);
  };
  R_Date("UCIAMS218494 M",675,15)
  {
   Outlier("General",0.05);
  };
  R_Date("UCIAMS218495 M",670,15)
  {
   Outlier("General",0.05);
  };
  R_Date("UCIAMS218496 M",595,20)
  {
   Outlier("General",0.05);
  };
  Interval("Interval Pethick",U(0,120));
  Date("Date Pethick");
 };
 Boundary("End Pethick",Date(U(1150,1635)));
};
Sequence()
{
 Boundary("Start Second Woods",Date(U(1150,1635)));
 Phase("Second Woods")
 {
  R_Combine("Feature 2 Shallow Pit Deposit - Assume One Event",8)
  {
   Outlier("General",0.05);
   R_Date("UCIAMS190546 B Feature 2",405,15)
   {
    Outlier("Simple",0.05);
   };
   R_Date("UCIAMS190547 B Feature 2",375,15)
   {
    Outlier("Simple",0.05);
   };
  };
  R_Date("UCIAMS190536 M",420,15)
  {
   Outlier("General",0.05);
  };
  R_Date("UCIAMS190535 M",380,20)
  {
   Outlier("General",0.05);
  };
  Interval("Interval Second Woods",U(0,120));
  Date("Date Second Woods");
 };
 Boundary("End Second Woods",Date(U(1150,1635)));
};
Sequence()
{
 Boundary ("Start Getman",Date(U(1150,1635)));
 Phase("Getman")
```

```
  {
   R_Date("M-783 Charcoal context not stated TPQ",560,150)
   {
    Outlier("Charcoal",1);
   };
   R_Date("UCIAMS190557 House 3 Hearth B",450,20)
   {
    Outlier("General",0.05);
   };
   R_Combine("Feature 28 Pit Event",8)
   {
    Outlier("General",0.05);
    R_Date("UCIAMS190558 House 3 B Pit",400,15)
    {
     Outlier("Simple",0.05);
    };
    R_Date("UCIAMS218481 House 3 M Pit",395,15)
    {
     Outlier("Simple",0.05);
    };
   };
   R_Date("UCIAMS218482 General Midden M",405,15)
   {
    Outlier("General",0.05);
   };
   R_Date("UCIAMS218480 House 5 Pit M",400,15)
   {
    Outlier("General",0.05);
   };
   R_Date("UCIAMS192976 House 1 Pit B",485,15)
   {
    Outlier("General",0.05);
   };
   R_Date("UCIAMS192975 Pit B",355,15)
   {
    Outlier("General",0.05);
   };
   R_Date("UCIAMS190555 Pit B",345,20)
   {
    Outlier("General",0.05);
   };
   R_Date("UCIAMS190556 Pit B",330,20)
   {
    Outlier("General",0.05);
   };
   Interval("Interval Getman",U(0,120));
   Date("Date Getman");
  };
  Boundary("End Getman",Date(U(1150,1635)));
 };
 Sequence()
 {
  Boundary("Start Elwood",Date(U(1150,1635)));
  Phase ("Elwood")
  {
   R_Date("UCIAMS190554 B Pit",420,20)
   {
```

```
   Outlier("General",0.05);
  };
  R_Date("AA-7410 M midden",409,49)
  {
   Outlier("General",0.05);
  };
  R_Date("AA-6425 M midden",380,50)
  {
   Outlier("General",0.05);
  };
  R_Date("UCIAMS190552 B Hearth", 380, 15)
  {
   Outlier("General",0.05);
  };
  R_Date("UCIAMS190553 B Hearth", 370, 20)
  {
   Outlier("General",0.05);
  };
  R_Date("AA-7697 M midden",288,49)
  {
   Outlier("General",0.05);
  };
  Interval("Interval Elwood",U(0,120));
  Date("Date Elwood");
 };
 Boundary("End Elwood",Date(U(1150,1635)));
};
Sequence()
{
 Boundary("Start Smith-Pagerie",Date(U(1150,1635)));
 Phase("Smith-Pagerie")
 {
  R_Combine("Feature 54 Pit H1 - assume one event",8)
  {
   Outlier("General",0.05);
   R_Date("AA-7405 M",430,50)
   {
    Outlier("SSimple",0.05);
   };
   R_Date("AA-6419 M",405,50)
   {
    Outlier("SSimple",0.05);
   };
   R_Date("UCIAMS218490 M",325,30)
   {
    Outlier("SSimple",0.05);
   };
  };
  R_Date("ISGS-A0528 R H1",445,40)
  {
   Outlier("Charcoal",1);
  };
  R_Date("UCIAMS190566 B H1",375,15)
  {
   Outlier("General",0.05);
  };
  R_Date("UCIAMS190565 B H1",370,15)
```

```
   {
    Outlier("General",0.05);
   };
   R_Date("UCIAMS190563 B H1",360,20)
   {
    Outlier("General",0.05);
   };
   //R_Date("UCIAMS218491 Material?? H1",265,15)
   //{
   // Outlier("General",0.05);
   //};
   //R_Date("UCIAMS218493 M Longhouse 5 Pit",480,15)
   //{
   // Outlier("General",0.05);
   //};
   R_Date("UCIAMS218492 M Longhouse 2 Hearth",350,15)
   {
    Outlier("General",0.05);
   };
   R_Date("UCIAMS190564 B Longhouse 4 Hearth",345,20)
   {
    Outlier("General",0.05);
   };
   Interval("Interval Smith-Pagerie",U(0,120));
   Date("Date Smith-Pagerie");
  };
  Boundary("End Smith-Pagerie",Date(U(1150,1635)));
 };
 Sequence()
 {
  Boundary("Start Otstungo",Date(U(1150,1635)));
  Phase("Otstungo House 1 Midden and Hearths")
  {
   R_Date("UCIAMS190551 Hearth B",355,20)
   {
    Outlier("General",0.05);
   };
   R_Date("UCIAMS190549 Hearth B",335,20)
   {
    Outlier("General",0.05);
   };
   R_Date("UCIAMS190550 Hearth B",310,15)
   {
    Outlier("General",0.05);
   };
   R_Date("UCIAMS190548 Hearth B",310,15)
   {
    Outlier("General",0.05);
   };
   R_Date("AA-7400 M",415,50)
   {
    Outlier("General",0.05);
   };
   R_Date("AA-7402 M",410,50)
   {
    Outlier("General",0.05);
   };
```

```
  R_Date("AA-6423 M",400,55)
  {
   Outlier("General",0.05);
  };
  R_Date("UCIAMS218483 M",400,15)
  {
   Outlier("General",0.05);
  };
  R_Date("AA-7398 M",380,55)
  {
   Outlier("General",0.05);
  };
  R_Date("UCIAMS218489 M",380,20)
  {
   Outlier("General",0.05);
  };
  R_Date("AA-7401 M",365,50)
  {
   Outlier("General",0.05);
  };
  R_Date("AA-7399 M",345,55)
  {
   Outlier("General",0.05);
  };
  R_Date("UCIAMS218487 M",340,15)
  {
   Outlier("General",0.05);
  };
  Interval("Interval Otstungo",U(0,120));
  Date("Date Otstungo");
 };
 Boundary("End Otstungo",Date(U(1150,1635)));
};
Sequence()
{
 Boundary("Start Klock",Date(U(1150,1635)));
 Phase("Klock")
 {
  R_Combine("Feature 84 Pit H1 - assume one event")
  {
   Outlier("General",0.05);
   R_Date("UCIAMS218474 M",365,15)
   {
    Outlier("SSimple",0.05);
   };
   R_Date("ISGS-A0326 M", 317, 38)
   {
    Outlier("SSimple",0.05);
   };
  };
  R_Date("ISGS-A0523 R Pit",480,40)
  {
   Outlier("Charcoal",1);
  };
  R_Date("UCIAMS218476 M Pit",365,15)
  {
   Outlier("General",0.05);
```

```
  };
  R_Date("UCIAMS190559 B Pit",360,15)
  {
   Outlier("General",0.05);
  };
  R_Date("UCIAMS190561 B Pit",335,15)
  {
   Outlier("General",0.05);
  };
  R_Date("UCIAMS218475 M Pit",335,20)
  {
   Outlier("General",0.05);
  };
  R_Date("UCIAMS218473 B Pit",325,15)
  {
   Outlier("General",0.05);
  };
  R_Date("UCIAMS190562 B Hearth H1",355,20)
  {
   Outlier("General",0.05);
  };
  R_Date("UCIAMS190560 M Hearth H1",325,15)
  {
   Outlier("General",0.05);
  };
  R_Date("AA-6418 M Hearth H7", 315, 60)
  {
   Outlier("General",0.05);
  };
  //R_Date("AA-7404 M Hearth H7",520,75)
  //{
  // Outlier("General",0.05);
  //};
  Interval("Interval Klock",U(0,120));
  Date("Date Klock");
 };
 Boundary("End Klock",Date(U(1150,1635)));
};
Sequence()
{
 Boundary("Start Cayadutta Midden",Date(U(1150,1635)));
 Phase("Cayadutta Midden")
 {
  //R_Date("AA-7689 M", 557, 58)
  //{
  // Outlier("General",0.05);
  //};
  R_Date("AA-7690 M", 415, 56)
  {
   Outlier("General",0.05);
  };
  R_Date("AA-7407 M", 367, 52)
  {
   Outlier("General",0.05);
  };
  R_Date("AA-6421 M", 300, 50)
  {
```

```
   Outlier("General",0.05);
  };
  R_Date("UCIAMS-205965 M", 355, 20)
  {
   Outlier("General",0.05);
  };
  R_Date("UCIAMS-205966 M", 340, 15)
  {
   Outlier("General",0.05);
  };
  R_Date("UCIAMS-205967 M", 315, 15)
  {
   Outlier("General",0.05);
  };
  R_Date("UCIAMS-205968 M", 375, 15)
  {
   Outlier("General",0.05);
  };
  Interval("Interval Cayadutta Midden",U(0,120));
  Date("Date Cayadutta Midden");
 };
 Boundary("End Cayadutta Midden",Date(U(1150,1635)));
};
Sequence()
{
 Boundary("Start Garoga",Date(U(1150,1635)));
 Phase("Garoga")
 {
  R_Date("Y-1381 charred wood",620,100)
  {
   Outlier("Charcoal",1);
  };
  R_Combine("Feature 2 Pit H9",8)
  {
   Outlier("General",0.05);
   //R_Date("AA-7695 M F2 Pit H9",431,39)
   //{
   // Outlier("SSimple",0.05);
   //};
   R_Date("AA-7403 M F2 Pit H9",410,60)
   {
    Outlier("SSimple",0.05);
   };
   R_Date("UCIAMS218478 M F2 Pit H9",345,15)
   {
    Outlier("SSimple",0.05);
   };
   R_Date("UCIAMS190537 M F2 Pit H9",335,20)
   {
    Outlier("SSimple",0.05);
   };
   R_Date("AA-6417 M F2 Pit H9",300,50)
   {
    Outlier("SSimple",0.05);
   };
  };
  //R_Date("AA-8370 M H9 Pit",585,40)
```

```
  //{
  // Outlier("General",0.05);
  //};
  R_Date("ISGS-A0522 R H5 Pit",425,40)
  {
   Outlier("Charcoal",1);
  };
  R_Date("UCIAMS190540 M H4 Pit",345,20)
  {
   Outlier("General",0.05);
  };
  R_Date("UCIAMS190539 M H2 Pit",320,20)
  {
   Outlier("General",0.05);
  };
  R_Date("UCIAMS218479 M H12 Pit",315,15)
  {
   Outlier("General",0.05);
  };
  R_Combine("F184 Pit btw H1&Stockade",8)
  {
   Outlier("General",0.05);
   R_Date("UCIAMS190538 M F184 Pit",305,20)
   {
    Outlier("SSimple",0.05);
   };
   R_Date("UCIAMS218477 M F184 Pit",330,20)
   {
    Outlier("SSimple",0.05);
   };
  };
  Interval("Interval Garoga",U(0,120));
  Date("Date Garoga");
 };
 Boundary("End Garoga",Date(U(1150,1635)));
};
Sequence()
{
 Boundary("Start Wormuth",Date(U(1150,1635)));
 Phase("Wormuth")
 {
  R_Date("AA-6416 M",385,50)
  {
   Outlier("General",0.05);
  };
  R_Date("UCIAMS192700 M Pit",360,15)
  {
   Outlier("General",0.05);
  };
  R_Date("AA-6065 M",340,25)
  {
   Outlier("General",0.05);
  };
  R_Date("DIC-1176 Lowest Level Midden Charcoal",560,50)
  {
   Outlier("Charcoal",1);
  };
```

```
      R_Date("DIC-1177 Higher Up Midden Charcoal",250,50)
      {
       Outlier("Charcoal",1);
      };
      R_Date("DIC-1178 Fill of Burial #2",410,55)
      {
       Outlier("Charcoal",1);
      };
      Interval("Interval Wormuth",U(0,120));
      Date("Date Wormuth");
     };
     Boundary("End Wormuth",Date(U(1150,1635)));
    };
    Order ("Order");
   };
  };
```

Model 2 RE-RUN adding Palatine Bridge and Briggs Run (each with 1580 TPQ)

```
Options()
 {
  Resolution=1;
 };
 Plot()
 {
  Outlier_Model("General",T(5),U(0,4),"t");
  Outlier_Model("Charcoal",Exp(1,-10,0),U(0,3),"t");
  Outlier_Model("SSimple",N(0,2),0,"s");
  Phase ()
  {
   Sequence()
   {
    Boundary("Start Snell Pits",Date(U(1150,1635)));
    Phase("Snell Pits")
    {
     R_Date("M-28 charred wood",1670,300)
     {
      Outlier("Charcoal",1);
     };
     R_Date("M-178 charred wood",1170,200)
     {
      Outlier("Charcoal",1);
     };
     R_Date("M-492 charred wood",794,200)
     {
      Outlier("Charcoal",1);
     };
     R_Date("UCIAMS190544 B",710,15)
     {
      Outlier("General",0.05);
     };
     R_Date("UCIAMS190542 B",710,20)
     {
      Outlier("General",0.05);
     };
```

```
  R_Date("UCIAMS190543 B",705,20)
  {
   Outlier("General",0.05);
  };
  R_Date("ISGS-A0327 M",691,39)
  {
   Outlier("General",0.05);
  };
  R_Date("UCIAMS192977 B",685,15)
  {
   Outlier("General",0.05);
  };
  Interval("Interval Snell",U(0,100));
  Date("Date Snell Pits");
 };
 Boundary("End Snell Pits",Date(U(1150,1635)));
};
Sequence()
{
 Boundary("Start Pethick",Date(U(1150,1635)));
 Phase("Pethick hearths or small pits")
 {
  R_Date("Beta 199857 C",730,70)
  {
   Outlier("Charcoal",1);
  };
  R_Date("Beta 198540 C",670,90)
  {
   Outlier("Charcoal",1);
  };
  R_Date("Beta 211490 C",560,40)
  {
   Outlier("Charcoal",1);
  };
  R_Date("Beta 22779 C",560,40)
  {
   Outlier("Charcoal",1);
  };
  R_Date("UCIAMS218494 M",675,15)
  {
   Outlier("General",0.05);
  };
  R_Date("UCIAMS218495 M",670,15)
  {
   Outlier("General",0.05);
  };
  R_Date("UCIAMS218496 M",595,20)
  {
   Outlier("General",0.05);
  };
  Interval("Interval Pethick",U(0,100));
  Date("Date Pethick");
 };
 Boundary("End Pethick",Date(U(1150,1635)));
};
Sequence()
{
```

```
 Boundary("Start Second Woods",Date(U(1150,1635)));
 Phase("Second Woods")
 {
  R_Combine("Feature 2 Shallow Pit Deposit - Assume One Event",8)
  {
   Outlier("General",0.05);
   R_Date("UCIAMS190546 B Feature 2",405,15)
   {
    Outlier("Simple",0.05);
   };
   R_Date("UCIAMS190547 B Feature 2",375,15)
   {
    Outlier("Simple",0.05);
   };
  };
  R_Date("UCIAMS190536 M",420,15)
  {
   Outlier("General",0.05);
  };
  R_Date("UCIAMS190535 M",380,20)
  {
   Outlier("General",0.05);
  };
  Interval("Interval Second Woods",U(0,100));
  Date("Date Second Woods");
 };
 Boundary("End Second Woods",Date(U(1150,1635)));
};
Sequence()
{
 Boundary ("Start Getman",Date(U(1150,1635)));
 Phase("Getman")
 {
  R_Date("M-783 Charcoal context not stated TPQ",560,150)
  {
   Outlier("Charcoal",1);
  };
  R_Date("UCIAMS190557 House 3 Hearth B",450,20)
  {
   Outlier("General",0.05);
  };
  R_Combine("Feature 28 Pit Event",8)
  {
   Outlier("General",0.05);
   R_Date("UCIAMS190558 House 3 B Pit",400,15)
   {
    Outlier("Simple",0.05);
   };
   R_Date("UCIAMS218481 House 3 M Pit",395,15)
   {
    Outlier("Simple",0.05);
   };
  };
  R_Date("UCIAMS218482 General Midden M",405,15)
  {
   Outlier("General",0.05);
  };
```

```
    R_Date("UCIAMS218480 House 5 Pit M",400,15)
    {
     Outlier("General",0.05);
    };
    //R_Date("UCIAMS192976 House 1 Pit B",485,15)
    //{
    // Outlier("General",0.05);
    //};
    R_Date("UCIAMS192975 Pit B",355,15)
    {
     Outlier("General",0.05);
    };
    R_Date("UCIAMS190555 Pit B",345,20)
    {
     Outlier("General",0.05);
    };
    R_Date("UCIAMS190556 Pit B",330,20)
    {
     Outlier("General",0.05);
    };
    Interval("Interval Getman",U(0,100));
    Date("Date Getman");
   };
   Boundary("End Getman",Date(U(1150,1635)));
  };
  Sequence()
  {
   Boundary("Start Elwood",Date(U(1150,1635)));
   Phase ("Elwood")
   {
    R_Date("UCIAMS190554 B Pit",420,20)
    {
     Outlier("General",0.05);
    };
    R_Date("AA-7410 M midden",409,49)
    {
     Outlier("General",0.05);
    };
    R_Date("AA-6425 M midden",380,50)
    {
     Outlier("General",0.05);
    };
    R_Date("UCIAMS190552 B Hearth", 380, 15)
    {
     Outlier("General",0.05);
    };
    R_Date("UCIAMS190553 B Hearth", 370, 20)
    {
     Outlier("General",0.05);
    };
    R_Date("AA-7697 M midden",288,49)
    {
     Outlier("General",0.05);
    };
    Interval("Interval Elwood",U(0,100));
    Date("Date Elwood");
   };
```

```
 Boundary("End Elwood",Date(U(1150,1635)));
};
Sequence()
{
 Boundary("Start Smith-Pagerie",Date(U(1150,1635)));
 Phase("Smith-Pagerie")
 {
  R_Combine("Feature 54 Pit H1 - assume one event",8)
  {
   Outlier("General",0.05);
   R_Date("AA-7405 M",430,50)
   {
    Outlier("SSimple",0.05);
   };
   R_Date("AA-6419 M",405,50)
   {
    Outlier("SSimple",0.05);
   };
   R_Date("UCIAMS218490 M",325,30)
   {
    Outlier("SSimple",0.05);
   };
  };
  R_Date("ISGS-A0528 R H1",445,40)
  {
   Outlier("Charcoal",1);
  };
  R_Date("UCIAMS190566 B H1",375,15)
  {
   Outlier("General",0.05);
  };
  R_Date("UCIAMS190565 B H1",370,15)
  {
   Outlier("General",0.05);
  };
  R_Date("UCIAMS190563 B H1",360,20)
  {
   Outlier("General",0.05);
  };
  //R_Date("UCIAMS218491 Material?? H1",265,15)
  //{
  // Outlier("General",0.05);
  //};
  //R_Date("UCIAMS218493 M Longhouse 5 Pit",480,15)
  //{
  // Outlier("General",0.05);
  //};
  R_Date("UCIAMS218492 M Longhouse 2 Hearth",350,15)
  {
   Outlier("General",0.05);
  };
  R_Date("UCIAMS190564 B Longhouse 4 Hearth",345,20)
  {
   Outlier("General",0.05);
  };
  Interval("Interval Smith-Pagerie",U(0,100));
  Date("Date Smith-Pagerie");
```

```
 };
 Boundary("End Smith-Pagerie",Date(U(1150,1635)));
};
Sequence()
{
 Boundary("Start Otstungo",Date(U(1150,1635)));
 Phase("Otstungo House 1 Midden and Hearths")
 {
  R_Date("UCIAMS190551 Hearth B",355,20)
  {
   Outlier("General",0.05);
  };
  R_Date("UCIAMS190549 Hearth B",335,20)
  {
   Outlier("General",0.05);
  };
  R_Date("UCIAMS190550 Hearth B",310,15)
  {
   Outlier("General",0.05);
  };
  R_Date("UCIAMS190548 Hearth B",310,15)
  {
   Outlier("General",0.05);
  };
  R_Date("AA-7400 M",415,50)
  {
   Outlier("General",0.05);
  };
  R_Date("AA-7402 M",410,50)
  {
   Outlier("General",0.05);
  };
  R_Date("AA-6423 M",400,55)
  {
   Outlier("General",0.05);
  };
  R_Date("UCIAMS218483 M",400,15)
  {
   Outlier("General",0.05);
  };
  R_Date("AA-7398 M",380,55)
  {
   Outlier("General",0.05);
  };
  R_Date("UCIAMS218489 M",380,20)
  {
   Outlier("General",0.05);
  };
  R_Date("AA-7401 M",365,50)
  {
   Outlier("General",0.05);
  };
  R_Date("AA-7399 M",345,55)
  {
   Outlier("General",0.05);
  };
  R_Date("UCIAMS218487 M",340,15)
```

```
   {
    Outlier("General",0.05);
   };
   Interval("Interval Otstungo",U(0,100));
   Date("Date Otstungo");
  };
  Boundary("End Otstungo",Date(U(1150,1635)));
 };
 Sequence()
 {
  Boundary("Start Klock",Date(U(1150,1635)));
  Phase("Klock")
  {
   R_Combine("Feature 84 Pit H1 - assume one event")
   {
    Outlier("General",0.05);
    R_Date("UCIAMS218474 M",365,15)
    {
     Outlier("SSimple",0.05);
    };
    R_Date("ISGS-A0326 M", 317, 38)
    {
     Outlier("SSimple",0.05);
    };
   };
   R_Date("ISGS-A0523 R Pit",480,40)
   {
    Outlier("Charcoal",1);
   };
   R_Date("UCIAMS218476 M Pit",365,15)
   {
    Outlier("General",0.05);
   };
   R_Date("UCIAMS190559 B Pit",360,15)
   {
    Outlier("General",0.05);
   };
   R_Date("UCIAMS190561 B Pit",335,15)
   {
    Outlier("General",0.05);
   };
   R_Date("UCIAMS218475 M Pit",335,20)
   {
    Outlier("General",0.05);
   };
   R_Date("UCIAMS218473 B Pit",325,15)
   {
    Outlier("General",0.05);
   };
   R_Date("UCIAMS190562 B Hearth H1",355,20)
   {
    Outlier("General",0.05);
   };
   R_Date("UCIAMS190560 M Hearth H1",325,15)
   {
    Outlier("General",0.05);
   };
```

```
  R_Date("AA-6418 M Hearth H7", 315, 60)
  {
   Outlier("General",0.05);
  };
  //R_Date("AA-7404 M Hearth H7",520,75)
  //{
  // Outlier("General",0.05);
  //};
  Interval("Interval Klock",U(0,100));
  Date("Date Klock");
 };
 Boundary("End Klock",Date(U(1150,1635)));
};
Sequence()
{
 Boundary("Start Cayadutta Midden",Date(U(1150,1635)));
 Phase("Cayadutta Midden")
 {
  //R_Date("AA-7689 M", 557, 58)
  //{
  // Outlier("General",0.05);
  //};
  R_Date("AA-7690 M", 415, 56)
  {
   Outlier("General",0.05);
  };
  R_Date("AA-7407 M", 367, 52)
  {
   Outlier("General",0.05);
  };
  R_Date("AA-6421 M", 300, 50)
  {
   Outlier("General",0.05);
  };
  R_Date("UCIAMS-205965 M", 355, 20)
  {
   Outlier("General",0.05);
  };
  R_Date("UCIAMS-205966 M", 340, 15)
  {
   Outlier("General",0.05);
  };
  R_Date("UCIAMS-205967 M", 315, 15)
  {
   Outlier("General",0.05);
  };
  R_Date("UCIAMS-205968 M", 375, 15)
  {
   Outlier("General",0.05);
  };
  Interval("Interval Cayadutta Midden",U(0,100));
  Date("Date Cayadutta Midden");
 };
 Boundary("End Cayadutta Midden",Date(U(1150,1635)));
};
Sequence()
{
```

```
Boundary("Start Garoga",Date(U(1150,1635)));
Phase("Garoga")
{
 R_Date("Y-1381 charred wood",620,100)
 {
  Outlier("Charcoal",1);
 };
 R_Combine("Feature 2 Pit H9",8)
 {
  Outlier("General",0.05);
  //R_Date("AA-7695 M F2 Pit H9",431,39)
  //{
  // Outlier("SSimple",0.05);
  //};
  R_Date("AA-7403 M F2 Pit H9",410,60)
  {
   Outlier("SSimple",0.05);
  };
  R_Date("UCIAMS218478 M F2 Pit H9",345,15)
  {
   Outlier("SSimple",0.05);
  };
  R_Date("UCIAMS190537 M F2 Pit H9",335,20)
  {
   Outlier("SSimple",0.05);
  };
  R_Date("AA-6417 M F2 Pit H9",300,50)
  {
   Outlier("SSimple",0.05);
  };
 };
 //R_Date("AA-8370 M H9 Pit",585,40)
 //{
 // Outlier("General",0.05);
 //};
 R_Date("ISGS-A0522 R H5 Pit",425,40)
 {
  Outlier("Charcoal",1);
 };
 R_Date("UCIAMS190540 M H4 Pit",345,20)
 {
  Outlier("General",0.05);
 };
 R_Date("UCIAMS190539 M H2 Pit",320,20)
 {
  Outlier("General",0.05);
 };
 R_Date("UCIAMS218479 M H12 Pit",315,15)
 {
  Outlier("General",0.05);
 };
 R_Combine("F184 Pit btw H1&Stockade",8)
 {
  Outlier("General",0.05);
  R_Date("UCIAMS190538 M F184 Pit",305,20)
  {
   Outlier("SSimple",0.05);
```

```
   };
   R_Date("UCIAMS218477 M F184 Pit",330,20)
   {
    Outlier("SSimple",0.05);
   };
  };
  Interval("Interval Garoga",U(0,100));
  Date("Date Garoga");
 };
 Boundary("End Garoga",Date(U(1150,1635)));
};
Sequence()
{
 Boundary("Start Wormuth",Date(U(1150,1635)));
 Phase("Wormuth")
 {
  R_Date("AA-6416 M",385,50)
  {
   Outlier("General",0.05);
  };
  R_Date("UCIAMS192700 M Pit",360,15)
  {
   Outlier("General",0.05);
  };
  R_Date("AA-6065 M",340,25)
  {
   Outlier("General",0.05);
  };
  R_Date("DIC-1176 Lowest Level Midden Charcoal",560,50)
  {
   Outlier("Charcoal",1);
  };
  R_Date("DIC-1177 Higher Up Midden Charcoal",250,50)
  {
   Outlier("Charcoal",1);
  };
  R_Date("DIC-1178 Fill of Burial #2",410,55)
  {
   Outlier("Charcoal",1);
  };
  Interval("Interval Wormuth",U(0,100));
  Date("Date Wormuth");
 };
 Boundary("End Wormuth",Date(U(1150,1635)));
};
Sequence()
{
 Boundary("Start Briggs Run");
 After(C_Date("Historic BR",1580));
 Phase("Briggs Run Pits")
 {
  R_Date("ISGSA0328 M",401,38)
  {
   Outlier("General",0.05);
  };
  R_Date("AA-7693 M",315,40)
  {
```

```
      Outlier("General",0.05);
     };
     R_Date("AA-AA-7417 M",290,37)
     {
      Outlier("General",0.05);
     };
     Interval("Interval Briggs Run Pits",U(0,100));
     Date("Date Briggs Run Pits");
    };
    Boundary("End Briggs Run",Date(U(1150,1635)));
   };
   Sequence()
   {
    Boundary("Start Palatine Bridge");
    After(C_Date("Historic PB",1580));
    Phase("Palatine Bridge Pits")
    {
     R_Date("ISGSA1713 M",325,15)
     {
      Outlier("General",0.05);
     };
     R_Date("OS-86116 M",310,25)
     {
      Outlier("General",0.05);
     };
     Interval("Interval Palatine Bridge Pits",U(0,100));
     Date("Date Palatine Bridge Pits");
    };
    Boundary("End Palatine Bridge",Date(U(1150,1635)));
   };
   Order ("Order");
  };
 };
```

Note: "Historic BR" and "Historic PB" are used to label the TPQ of 1580 for each site. This is noted in the captions to Figs 3 and 4. In Figure 4 it is noted that each "= TPQ 1580" to highlight.

Model 3

```
Options()
 {
  Resolution=1;
 };
 Plot()
 {
  Outlier_Model("General",T(5),U(0,4),"t");
  Outlier_Model("Charcoal",Exp(1,-10,0),U(0,3),"t");
  Outlier_Model("SSimple",N(0,2),0,"s");
  Sequence()
  {
   Boundary("Start Snell Pits",Date(U(1150,1635)));
   Phase("Snell Pits")
```

```
{
 R_Date("M-28 charred wood",1670,300)
 {
  Outlier("Charcoal",1);
 };
 R_Date("M-178 charred wood",1170,200)
 {
  Outlier("Charcoal",1);
 };
 R_Date("M-492 charred wood",794,200)
 {
  Outlier("Charcoal",1);
 };
 R_Date("UCIAMS190544 B",710,15)
 {
  Outlier("General",0.05);
 };
 R_Date("UCIAMS190542 B",710,20)
 {
  Outlier("General",0.05);
 };
 R_Date("UCIAMS190543 B",705,20)
 {
  Outlier("General",0.05);
 };
 R_Date("ISGS-A0327 M",691,39)
 {
  Outlier("General",0.05);
 };
 R_Date("UCIAMS192977 B",685,15)
 {
  Outlier("General",0.05);
 };
 Interval("Interval Snell",U(0,100));
 Date("Date Snell Pits");
};
Boundary("End Snell Pits");
Interval("Snell to Pethick");
Boundary("Start Pethick");
Phase("Pethick hearths or small pits")
{
 R_Date("Beta 199857 C",730,70)
 {
  Outlier("Charcoal",1);
 };
 R_Date("Beta 198540 C",670,90)
 {
  Outlier("Charcoal",1);
 };
 R_Date("Beta 211490 C",560,40)
 {
  Outlier("Charcoal",1);
 };
 R_Date("Beta 22779 C",560,40)
 {
  Outlier("Charcoal",1);
 };
```

```
 R_Date("UCIAMS218494 M",675,15)
 {
  Outlier("General",0.05);
 };
 R_Date("UCIAMS218495 M",670,15)
 {
  Outlier("General",0.05);
 };
 R_Date("UCIAMS218496 M",595,20)
 {
  Outlier("General",0.05);
 };
 Interval("Interval Pethick",U(0,100));
 Date("Date Pethick");
};
Boundary("End Pethick");
Interval("Pethick to Second Woods");
Phase ("Start Period Overlapping Sites")
{
 Sequence()
 {
  Boundary("Start Second Woods");
  Phase("Second Woods")
  {
   R_Combine("Feature 2 Shallow Pit Deposit - Assume One Event",8)
   {
    Outlier("General",0.05);
    R_Date("UCIAMS190546 B Feature 2",405,15)
    {
     Outlier("Simple",0.05);
    };
    R_Date("UCIAMS190547 B Feature 2",375,15)
    {
     Outlier("Simple",0.05);
    };
   };
   R_Date("UCIAMS190536 M",420,15)
   {
    Outlier("General",0.05);
   };
   R_Date("UCIAMS190535 M",380,20)
   {
    Outlier("General",0.05);
   };
   Interval("Interval Second Woods",U(0,100));
   Date("Date Second Woods");
  };
  Boundary("End Second Woods",Date(U(1150,1635)));
 };
 Sequence()
 {
  Boundary ("Start Getman");
  Phase("Getman")
  {
   R_Date("M-783 Charcoal context not stated TPQ",560,150)
   {
    Outlier("Charcoal",1);
```

```
   };
   R_Date("UCIAMS190557 House 3 Hearth B",450,20)
   {
    Outlier("General",0.05);
   };
   R_Combine("Feature 28 Pit Event",8)
   {
    Outlier("General",0.05);
    R_Date("UCIAMS190558 House 3 B Pit",400,15)
    {
     Outlier("Simple",0.05);
    };
    R_Date("UCIAMS218481 House 3 M Pit",395,15)
    {
     Outlier("Simple",0.05);
    };
   };
   R_Date("UCIAMS218482 General Midden M",405,15)
   {
    Outlier("General",0.05);
   };
   R_Date("UCIAMS218480 House 5 Pit M",400,15)
   {
    Outlier("General",0.05);
   };
   //R_Date("UCIAMS192976 House 1 Pit B",485,15)
   //{
   // Outlier("General",0.05);
   //};
   R_Date("UCIAMS192975 Pit B",355,15)
   {
    Outlier("General",0.05);
   };
   R_Date("UCIAMS190555 Pit B",345,20)
   {
    Outlier("General",0.05);
   };
   R_Date("UCIAMS190556 Pit B",330,20)
   {
    Outlier("General",0.05);
   };
   Interval("Interval Getman",U(0,100));
   Date("Date Getman");
  };
  Boundary("End Getman",Date(U(1150,1635)));
 };
 Sequence()
 {
  Boundary("Start Elwood");
  Phase ("Elwood")
  {
   R_Date("UCIAMS190554 B Pit",420,20)
   {
    Outlier("General",0.05);
   };
   R_Date("AA-7410 M midden",409,49)
   {
```

```
   Outlier("General",0.05);
  };
  R_Date("AA-6425 M midden",380,50)
  {
   Outlier("General",0.05);
  };
  R_Date("UCIAMS190552 B Hearth", 380, 15)
  {
   Outlier("General",0.05);
  };
  R_Date("UCIAMS190553 B Hearth", 370, 20)
  {
   Outlier("General",0.05);
  };
  R_Date("AA-7697 M midden",288,49)
  {
   Outlier("General",0.05);
  };
  Interval("Interval Elwood",U(0,100));
  Date("Date Elwood");
 };
 Boundary("End Elwood",Date(U(1150,1635)));
};
Sequence()
{
 Boundary("Start Smith-Pagerie");
 Phase("Smith-Pagerie")
 {
  R_Combine("Feature 54 Pit H1 - assume one event",8)
  {
   Outlier("General",0.05);
   R_Date("AA-7405 M",430,50)
   {
    Outlier("SSimple",0.05);
   };
   R_Date("AA-6419 M",405,50)
   {
    Outlier("SSimple",0.05);
   };
   R_Date("UCIAMS218490 M",325,30)
   {
    Outlier("SSimple",0.05);
   };
  };
  R_Date("ISGS-A0528 R H1",445,40)
  {
   Outlier("Charcoal",1);
  };
  R_Date("UCIAMS190566 B H1",375,15)
  {
   Outlier("General",0.05);
  };
  R_Date("UCIAMS190565 B H1",370,15)
  {
   Outlier("General",0.05);
  };
  R_Date("UCIAMS190563 B H1",360,20)
```

```
   {
    Outlier("General",0.05);
   };
   //R_Date("UCIAMS218491 Material?? H1",265,15)
   //{
   // Outlier("General",0.05);
   //};
   //Outlier, too recent - assume intrusive
   //R_Date("UCIAMS218493 M Longhouse 5 Pit",480,15)
   //{
   // Outlier("General",0.05);
   //};
   //Outlier, too old
   R_Date("UCIAMS218492 M Longhouse 2 Hearth",350,15)
   {
    Outlier("General",0.05);
   };
   R_Date("UCIAMS190564 B Longhouse 4 Hearth",345,20)
   {
    Outlier("General",0.05);
   };
   Interval("Interval Smith-Pagerie",U(0,100));
   Date("Date Smith-Pagerie");
  };
  Boundary("End Smith-Pagerie",Date(U(1150,1635)));
 };
 Sequence()
 {
  Boundary("Start Otstungo");
  Phase("Otstungo House 1 Midden and Hearths")
  {
   R_Date("UCIAMS190551 Hearth B",355,20)
   {
    Outlier("General",0.05);
   };
   R_Date("UCIAMS190549 Hearth B",335,20)
   {
    Outlier("General",0.05);
   };
   R_Date("UCIAMS190550 Hearth B",310,15)
   {
    Outlier("General",0.05);
   };
   R_Date("UCIAMS190548 Hearth B",310,15)
   {
    Outlier("General",0.05);
   };
   R_Date("AA-7400 M",415,50)
   {
    Outlier("General",0.05);
   };
   R_Date("AA-7402 M",410,50)
   {
    Outlier("General",0.05);
   };
   R_Date("AA-6423 M",400,55)
   {
```

```
   Outlier("General",0.05);
  };
  R_Date("UCIAMS218483 M",400,15)
  {
   Outlier("General",0.05);
  };
  R_Date("AA-7398 M",380,55)
  {
   Outlier("General",0.05);
  };
  R_Date("UCIAMS218489 M",380,20)
  {
   Outlier("General",0.05);
  };
  R_Date("AA-7401 M",365,50)
  {
   Outlier("General",0.05);
  };
  R_Date("AA-7399 M",345,55)
  {
   Outlier("General",0.05);
  };
  R_Date("UCIAMS218487 M",340,15)
  {
   Outlier("General",0.05);
  };
  Interval("Interval Otstungo",U(0,100));
  Date("Date Otstungo");
 };
 Boundary("End Otstungo",Date(U(1150,1635)));
};
Sequence()
{
 Boundary("Start Klock");
 Phase("Klock")
 {
  R_Combine("Feature 84 Pit H1 - assume one event")
  {
   Outlier("General",0.05);
   R_Date("UCIAMS218474 M",365,15)
   {
    Outlier("SSimple",0.05);
   };
   R_Date("ISGS-A0326 M", 317, 38)
   {
    Outlier("SSimple",0.05);
   };
  };
  R_Date("ISGS-A0523 R Pit",480,40)
  {
   Outlier("Charcoal",1);
  };
  R_Date("UCIAMS218476 M Pit",365,15)
  {
   Outlier("General",0.05);
  };
  R_Date("UCIAMS190559 B Pit",360,15)
```

```
      {
       Outlier("General",0.05);
      };
      R_Date("UCIAMS190561 B Pit",335,15)
      {
       Outlier("General",0.05);
      };
      R_Date("UCIAMS218475 M Pit",335,20)
      {
       Outlier("General",0.05);
      };
      R_Date("UCIAMS218473 B Pit",325,15)
      {
       Outlier("General",0.05);
      };
      R_Date("UCIAMS190562 B Hearth H1",355,20)
      {
       Outlier("General",0.05);
      };
      R_Date("UCIAMS190560 M Hearth H1",325,15)
      {
       Outlier("General",0.05);
      };
      R_Date("AA-6418 M Hearth H7", 315, 60)
      {
       Outlier("General",0.05);
      };
      //R_Date("AA-7404 M Hearth H7",520,75)
      //{
      // Outlier("General",0.05);
      // };
      //A-6418 & 7404 both maize from same context but v different and fail
W&W test; exclude AA-7404 as too old - v poor agreement also
      Interval("Interval Klock",U(0,100));
      Date("Date Klock");
     };
     Boundary("End Klock",Date(U(1150,1635)));
    };
    Sequence()
    {
     Boundary("Start Cayadutta Midden");
     Phase("Cayadutta Midden")
     {
      //R_Date("AA-7689 M", 557, 58)
      //{
      // Outlier("General",0.05);
      //};
      //Outlier too old
      R_Date("AA-7690 M", 415, 56)
      {
       Outlier("General",0.05);
      };
      R_Date("AA-7407 M", 367, 52)
      {
       Outlier("General",0.05);
      };
      R_Date("AA-6421 M", 300, 50)
```

```
      {
       Outlier("General",0.05);
      };
      R_Date("UCIAMS-205965 M", 355, 20)
      {
       Outlier("General",0.05);
      };
      R_Date("UCIAMS-205966 M", 340, 15)
      {
       Outlier("General",0.05);
      };
      R_Date("UCIAMS-205967 M", 315, 15)
      {
       Outlier("General",0.05);
      };
      R_Date("UCIAMS-205968 M", 375, 15)
      {
       Outlier("General",0.05);
      };
      Interval("Interval Cayadutta Midden",U(0,100));
      Date("Date Cayadutta Midden");
     };
     Boundary("End Cayadutta Midden",Date(U(1150,1635)));
    };
    Sequence()
    {
     Boundary("Start Garoga");
     Phase("Garoga")
     {
      R_Date("Y-1381 charred wood",620,100)
      {
       Outlier("Charcoal",1);
      };
      R_Combine("Feature 2 Pit H9",8)
      {
       Outlier("General",0.05);
       //R_Date("AA-7695 M F2 Pit H9",431,39)
       //{
       // Outlier("SSimple",0.05);
       //};
       //Outlier too old. Note Snow 1995:162 on possibility earlier
occupation at site locus.
       R_Date("AA-7403 M F2 Pit H9",410,60)
       {
        Outlier("SSimple",0.05);
       };
       R_Date("UCIAMS218478 M F2 Pit H9",345,15)
       {
        Outlier("SSimple",0.05);
       };
       R_Date("UCIAMS190537 M F2 Pit H9",335,20)
       {
        Outlier("SSimple",0.05);
       };
       R_Date("AA-6417 M F2 Pit H9",300,50)
       {
        Outlier("SSimple",0.05);
```

```
        };
       };
       //R_Date("AA-8370 M H9 Pit",585,40)
       //{
       // Outlier("General",0.05);
       //};
       //Too old outlier. Note Snow 1995:162 on possibility earlier occupation
at site locus.
       R_Date("ISGS-A0522 R H5 Pit",425,40)
       {
        Outlier("Charcoal",1);
       };
       R_Date("UCIAMS190540 M H4 Pit",345,20)
       {
        Outlier("General",0.05);
       };
       R_Date("UCIAMS190539 M H2 Pit",320,20)
       {
        Outlier("General",0.05);
       };
       R_Date("UCIAMS218479 M H12 Pit",315,15)
       {
        Outlier("General",0.05);
       };
       R_Combine("F184 Pit btw H1&Stockade",8)
       {
        Outlier("General",0.05);
        R_Date("UCIAMS190538 M F184 Pit",305,20)
        {
         Outlier("SSimple",0.05);
        };
        R_Date("UCIAMS218477 M F184 Pit",330,20)
        {
         Outlier("SSimple",0.05);
        };
       };
       Interval("Interval Garoga",U(0,100));
       Date("Date Garoga");
      };
      Boundary("End Garoga",Date(U(1150,1635)));
     };
     Sequence()
     {
      Boundary("Start Wormuth");
      Phase("Wormuth")
      {
       R_Date("AA-6416 M",385,50)
       {
        Outlier("General",0.05);
       };
       R_Date("UCIAMS192700 M Pit",360,15)
       {
        Outlier("General",0.05);
       };
       R_Date("AA-6065 M",340,25)
       {
        Outlier("General",0.05);
```

```
  };
  R_Date("DIC-1176 Lowest Level Midden Charcoal",560,50)
  {
   Outlier("Charcoal",1);
  };
  R_Date("DIC-1177 Higher Up Midden Charcoal",250,50)
  {
   Outlier("Charcoal",1);
  };
  R_Date("DIC-1178 Fill of Burial #2",410,55)
  {
   Outlier("Charcoal",1);
  };
  Interval("Interval Wormuth",U(0,100));
  Date("Date Wormuth");
 };
 Boundary("End Wormuth",Date(U(1150,1635)));
};
Sequence()
{
 Boundary("Start Palatine Bridge");
 After(C_Date("Historic PB",1580));
 Phase("Palatine Bridge Pits")
 {
  R_Date("ISGSA1713 M",325,15)
  {
   Outlier("General",0.05);
  };
  R_Date("OS-86116 M",310,25)
  {
   Outlier("General",0.05);
  };
  Interval("Interval Palatine Bridge Pits",U(0,100));
  Date("Date Palatine Bridge Pits");
 };
 Boundary("End Palatine Bridge",Date(U(1150,1635)));
};
Sequence()
{
 Boundary("Start Briggs Run");
 After(C_Date("Historic BR",1580));
 Phase("Briggs Run Pits")
 {
  R_Date("ISGSA0328 M",401,38)
  {
   Outlier("General",0.05);
  };
  R_Date("AA-7693 M",315,40)
  {
   Outlier("General",0.05);
  };
  R_Date("AA-AA-7417 M",290,37)
  {
   Outlier("General",0.05);
  };
  Interval("Interval Briggs Run Pits",U(0,100));
  Date("Date Briggs Run Pits");
```

```
      };
      Boundary("End Briggs Run",Date(U(1150,1635)));
     };
    };
   };
   Sequence ("Overlapping but Ordered 11 Site Phase Group")
   {
    Date("=Date Second Woods");
    Date("=Date Getman");
    Date("=Date Elwood");
    Date("=Date Smith-Pagerie");
    Date("=Date Otstungo");
    Date("=Date Klock");
    Date("=Date Cayadutta Midden");
    Date("=Date Garoga");
    Date("=Date Wormuth");
    Date("=Date Palatine Bridge Pits");
    Date("=Date Briggs Run Pits");
   };
  };
```

Model 3A

```
Options()
 {
  Resolution=1;
 };
 Plot()
 {
  Outlier_Model("General",T(5),U(0,4),"t");
  Outlier_Model("Charcoal",Exp(1,-10,0),U(0,3),"t");
  Outlier_Model("SSimple",N(0,2),0,"s");
  Sequence()
  {
   Boundary("Start Snell Pits",Date(U(1150,1635)));
   Phase("Snell Pits")
   {
    R_Date("M-28 charred wood",1670,300)
    {
     Outlier("Charcoal",1);
    };
    R_Date("M-178 charred wood",1170,200)
    {
     Outlier("Charcoal",1);
    };
    R_Date("M-492 charred wood",794,200)
    {
     Outlier("Charcoal",1);
    };
    R_Date("UCIAMS190544 B",710,15)
    {
     Outlier("General",0.05);
    };
    R_Date("UCIAMS190542 B",710,20)
```

```
 {
  Outlier("General",0.05);
 };
 R_Date("UCIAMS190543 B",705,20)
 {
  Outlier("General",0.05);
 };
 R_Date("ISGS-A0327 M",691,39)
 {
  Outlier("General",0.05);
 };
 R_Date("UCIAMS192977 B",685,15)
 {
  Outlier("General",0.05);
 };
 Interval("Interval Snell",U(0,100));
 Date("Date Snell Pits");
};
Boundary("End Snell Pits");
Interval("Snell to Pethick");
Boundary("Start Pethick");
Phase("Pethick hearths or small pits")
{
 R_Date("Beta 199857 C",730,70)
 {
  Outlier("Charcoal",1);
 };
 R_Date("Beta 198540 C",670,90)
 {
  Outlier("Charcoal",1);
 };
 R_Date("Beta 211490 C",560,40)
 {
  Outlier("Charcoal",1);
 };
 R_Date("Beta 22779 C",560,40)
 {
  Outlier("Charcoal",1);
 };
 R_Date("UCIAMS218494 M",675,15)
 {
  Outlier("General",0.05);
 };
 R_Date("UCIAMS218495 M",670,15)
 {
  Outlier("General",0.05);
 };
 R_Date("UCIAMS218496 M",595,20)
 {
  Outlier("General",0.05);
 };
 Interval("Interval Pethick",U(0,100));
 Date("Date Pethick");
};
Boundary("End Pethick");
Interval("Pethick to Second Woods");
Phase ("Start Period Overlapping Sites")
```

```
{
 Sequence()
 {
  Boundary("Start Second Woods");
  Phase("Second Woods")
  {
   R_Combine("Feature 2 Shallow Pit Deposit - Assume One Event",8)
   {
    Outlier("General",0.05);
    R_Date("UCIAMS190546 B Feature 2",405,15)
    {
     Outlier("Simple",0.05);
    };
    R_Date("UCIAMS190547 B Feature 2",375,15)
    {
     Outlier("Simple",0.05);
    };
   };
   R_Date("UCIAMS190536 M",420,15)
   {
    Outlier("General",0.05);
   };
   R_Date("UCIAMS190535 M",380,20)
   {
    Outlier("General",0.05);
   };
   Interval("Interval Second Woods",U(0,100));
   Date("Date Second Woods");
  };
  Boundary("End Second Woods",Date(U(1150,1635)));
 };
 Sequence()
 {
  Boundary ("Start Getman");
  Phase("Getman")
  {
   R_Date("M-783 Charcoal context not stated TPQ",560,150)
   {
    Outlier("Charcoal",1);
   };
   R_Date("UCIAMS190557 House 3 Hearth B",450,20)
   {
    Outlier("General",0.05);
   };
   R_Combine("Feature 28 Pit Event",8)
   {
    Outlier("General",0.05);
    R_Date("UCIAMS190558 House 3 B Pit",400,15)
    {
     Outlier("Simple",0.05);
    };
    R_Date("UCIAMS218481 House 3 M Pit",395,15)
    {
     Outlier("Simple",0.05);
    };
   };
   R_Date("UCIAMS218482 General Midden M",405,15)
```

```
   {
    Outlier("General",0.05);
   };
   R_Date("UCIAMS218480 House 5 Pit M",400,15)
   {
    Outlier("General",0.05);
   };
   //R_Date("UCIAMS192976 House 1 Pit B",485,15)
   //{
   // Outlier("General",0.05);
   //};
   R_Date("UCIAMS192975 Pit B",355,15)
   {
    Outlier("General",0.05);
   };
   R_Date("UCIAMS190555 Pit B",345,20)
   {
    Outlier("General",0.05);
   };
   R_Date("UCIAMS190556 Pit B",330,20)
   {
    Outlier("General",0.05);
   };
   Interval("Interval Getman",U(0,100));
   Date("Date Getman");
  };
  Boundary("End Getman",Date(U(1150,1635)));
 };
 Sequence()
 {
  Boundary("Start Elwood");
  Phase ("Elwood")
  {
   R_Date("UCIAMS190554 B Pit",420,20)
   {
    Outlier("General",0.05);
   };
   R_Date("AA-7410 M midden",409,49)
   {
    Outlier("General",0.05);
   };
   R_Date("AA-6425 M midden",380,50)
   {
    Outlier("General",0.05);
   };
   R_Date("UCIAMS190552 B Hearth", 380, 15)
   {
    Outlier("General",0.05);
   };
   R_Date("UCIAMS190553 B Hearth", 370, 20)
   {
    Outlier("General",0.05);
   };
   R_Date("AA-7697 M midden",288,49)
   {
    Outlier("General",0.05);
   };
```

```
  Interval("Interval Elwood",U(0,100));
  Date("Date Elwood");
 };
 Boundary("End Elwood",Date(U(1150,1635)));
};
Sequence()
{
 Boundary("Start Smith-Pagerie");
 Phase("Smith-Pagerie")
 {
  R_Combine("Feature 54 Pit H1 - assume one event",8)
  {
   Outlier("General",0.05);
   R_Date("AA-7405 M",430,50)
   {
    Outlier("SSimple",0.05);
   };
   R_Date("AA-6419 M",405,50)
   {
    Outlier("SSimple",0.05);
   };
   R_Date("UCIAMS218490 M",325,30)
   {
    Outlier("SSimple",0.05);
   };
  };
  R_Date("ISGS-A0528 R H1",445,40)
  {
   Outlier("Charcoal",1);
  };
  R_Date("UCIAMS190566 B H1",375,15)
  {
   Outlier("General",0.05);
  };
  R_Date("UCIAMS190565 B H1",370,15)
  {
   Outlier("General",0.05);
  };
  R_Date("UCIAMS190563 B H1",360,20)
  {
   Outlier("General",0.05);
  };
  //R_Date("UCIAMS218491 Material?? H1",265,15)
  //{
  // Outlier("General",0.05);
  //};
  //Outlier, too recent - assume intrusive
  //R_Date("UCIAMS218493 M Longhouse 5 Pit",480,15)
  //{
  // Outlier("General",0.05);
  //};
  //Outlier, too old
  R_Date("UCIAMS218492 M Longhouse 2 Hearth",350,15)
  {
   Outlier("General",0.05);
  };
  R_Date("UCIAMS190564 B Longhouse 4 Hearth",345,20)
```

```
   {
    Outlier("General",0.05);
   };
   Interval("Interval Smith-Pagerie",U(0,100));
   Date("Date Smith-Pagerie");
  };
  Boundary("End Smith-Pagerie",Date(U(1150,1635)));
 };
 Sequence()
 {
  Boundary("Start Otstungo");
  Phase("Otstungo House 1 Midden and Hearths")
  {
   R_Date("UCIAMS190551 Hearth B",355,20)
   {
    Outlier("General",0.05);
   };
   R_Date("UCIAMS190549 Hearth B",335,20)
   {
    Outlier("General",0.05);
   };
   R_Date("UCIAMS190550 Hearth B",310,15)
   {
    Outlier("General",0.05);
   };
   R_Date("UCIAMS190548 Hearth B",310,15)
   {
    Outlier("General",0.05);
   };
   R_Date("AA-7400 M",415,50)
   {
    Outlier("General",0.05);
   };
   R_Date("AA-7402 M",410,50)
   {
    Outlier("General",0.05);
   };
   R_Date("AA-6423 M",400,55)
   {
    Outlier("General",0.05);
   };
   R_Date("UCIAMS218483 M",400,15)
   {
    Outlier("General",0.05);
   };
   R_Date("AA-7398 M",380,55)
   {
    Outlier("General",0.05);
   };
   R_Date("UCIAMS218489 M",380,20)
   {
    Outlier("General",0.05);
   };
   R_Date("AA-7401 M",365,50)
   {
    Outlier("General",0.05);
   };
```

```
  R_Date("AA-7399 M",345,55)
  {
   Outlier("General",0.05);
  };
  R_Date("UCIAMS218487 M",340,15)
  {
   Outlier("General",0.05);
  };
  Interval("Interval Otstungo",U(0,100));
  Date("Date Otstungo");
 };
 Boundary("End Otstungo",Date(U(1150,1635)));
};
Sequence()
{
 Boundary("Start Klock");
 Phase("Klock")
 {
  R_Combine("Feature 84 Pit H1 - assume one event")
  {
   Outlier("General",0.05);
   R_Date("UCIAMS218474 M",365,15)
   {
    Outlier("SSimple",0.05);
   };
   R_Date("ISGS-A0326 M", 317, 38)
   {
    Outlier("SSimple",0.05);
   };
  };
  R_Date("ISGS-A0523 R Pit",480,40)
  {
   Outlier("Charcoal",1);
  };
  R_Date("UCIAMS218476 M Pit",365,15)
  {
   Outlier("General",0.05);
  };
  R_Date("UCIAMS190559 B Pit",360,15)
  {
   Outlier("General",0.05);
  };
  R_Date("UCIAMS190561 B Pit",335,15)
  {
   Outlier("General",0.05);
  };
  R_Date("UCIAMS218475 M Pit",335,20)
  {
   Outlier("General",0.05);
  };
  R_Date("UCIAMS218473 B Pit",325,15)
  {
   Outlier("General",0.05);
  };
  R_Date("UCIAMS190562 B Hearth H1",355,20)
  {
   Outlier("General",0.05);
```

```
      };
      R_Date("UCIAMS190560 M Hearth H1",325,15)
      {
       Outlier("General",0.05);
      };
      R_Date("AA-6418 M Hearth H7", 315, 60)
      {
       Outlier("General",0.05);
      };
      //R_Date("AA-7404 M Hearth H7",520,75)
      //{
      // Outlier("General",0.05);
      // };
      //A-6418 & 7404 both maize from same context but v different and fail
W&W test; exclude AA-7404 as too old - v poor agreement also
      Interval("Interval Klock",U(0,100));
      Date("Date Klock");
     };
     Boundary("End Klock",Date(U(1150,1635)));
    };
    Sequence()
    {
     Boundary("Start Cayadutta Midden");
     Phase("Cayadutta Midden")
     {
      //R_Date("AA-7689 M", 557, 58)
      //{
      // Outlier("General",0.05);
      //};
      //Outlier too old
      R_Date("AA-7690 M", 415, 56)
      {
       Outlier("General",0.05);
      };
      R_Date("AA-7407 M", 367, 52)
      {
       Outlier("General",0.05);
      };
      R_Date("AA-6421 M", 300, 50)
      {
       Outlier("General",0.05);
      };
      R_Date("UCIAMS-205965 M", 355, 20)
      {
       Outlier("General",0.05);
      };
      R_Date("UCIAMS-205966 M", 340, 15)
      {
       Outlier("General",0.05);
      };
      R_Date("UCIAMS-205967 M", 315, 15)
      {
       Outlier("General",0.05);
      };
      R_Date("UCIAMS-205968 M", 375, 15)
      {
       Outlier("General",0.05);
```

```
      };
      Interval("Interval Cayadutta Midden",U(0,100));
      Date("Date Cayadutta Midden");
     };
     Boundary("End Cayadutta Midden",Date(U(1150,1635)));
    };
    Sequence()
    {
     Boundary("Start Garoga");
     Phase("Garoga")
     {
      R_Date("Y-1381 charred wood",620,100)
      {
       Outlier("Charcoal",1);
      };
      R_Combine("Feature 2 Pit H9",8)
      {
       Outlier("General",0.05);
       //R_Date("AA-7695 M F2 Pit H9",431,39)
       //{
       // Outlier("SSimple",0.05);
       //};
       //Outlier too old. Note Snow 1995:162 on possibility earlier
occupation at site locus.
       R_Date("AA-7403 M F2 Pit H9",410,60)
       {
        Outlier("SSimple",0.05);
       };
       R_Date("UCIAMS218478 M F2 Pit H9",345,15)
       {
        Outlier("SSimple",0.05);
       };
       R_Date("UCIAMS190537 M F2 Pit H9",335,20)
       {
        Outlier("SSimple",0.05);
       };
       R_Date("AA-6417 M F2 Pit H9",300,50)
       {
        Outlier("SSimple",0.05);
       };
      };
      //R_Date("AA-8370 M H9 Pit",585,40)
      //{
      // Outlier("General",0.05);
      //};
      //Too old outlier. Note Snow 1995:162 on possibility earlier occupation
at site locus.
      R_Date("ISGS-A0522 R H5 Pit",425,40)
      {
       Outlier("Charcoal",1);
      };
      R_Date("UCIAMS190540 M H4 Pit",345,20)
      {
       Outlier("General",0.05);
      };
      R_Date("UCIAMS190539 M H2 Pit",320,20)
      {
```

```
   Outlier("General",0.05);
  };
  R_Date("UCIAMS218479 M H12 Pit",315,15)
  {
   Outlier("General",0.05);
  };
  R_Combine("F184 Pit btw H1&Stockade",8)
  {
   Outlier("General",0.05);
   R_Date("UCIAMS190538 M F184 Pit",305,20)
   {
    Outlier("SSimple",0.05);
   };
   R_Date("UCIAMS218477 M F184 Pit",330,20)
   {
    Outlier("SSimple",0.05);
   };
  };
  Interval("Interval Garoga",U(0,100));
  Date("Date Garoga");
 };
 Boundary("End Garoga",Date(U(1150,1635)));
};
Sequence()
{
 Boundary("Start Wormuth");
 Phase("Wormuth")
 {
  R_Date("AA-6416 M",385,50)
  {
   Outlier("General",0.05);
  };
  R_Date("UCIAMS192700 M Pit",360,15)
  {
   Outlier("General",0.05);
  };
  R_Date("AA-6065 M",340,25)
  {
   Outlier("General",0.05);
  };
  R_Date("DIC-1176 Lowest Level Midden Charcoal",560,50)
  {
   Outlier("Charcoal",1);
  };
  R_Date("DIC-1177 Higher Up Midden Charcoal",250,50)
  {
   Outlier("Charcoal",1);
  };
  R_Date("DIC-1178 Fill of Burial #2",410,55)
  {
   Outlier("Charcoal",1);
  };
  Interval("Interval Wormuth",U(0,100));
  Date("Date Wormuth");
 };
 Boundary("End Wormuth",Date(U(1150,1635)));
};
```

```
   Sequence()
   {
    Boundary("Start Palatine Bridge");
    After(C_Date("Historic PB",1580));
    Phase("Palatine Bridge Pits")
    {
     R_Date("ISGSA1713 M",325,15)
     {
      Outlier("General",0.05);
     };
     R_Date("OS-86116 M",310,25)
     {
      Outlier("General",0.05);
     };
     Interval("Interval Palatine Bridge Pits",U(0,100));
     Date("Date Palatine Bridge Pits");
    };
    Boundary("End Palatine Bridge",Date(U(1150,1635)));
   };
   Sequence()
   {
    Boundary("Start Briggs Run");
    After(C_Date("Historic BR",1580));
    Phase("Briggs Run Pits")
    {
     R_Date("ISGSA0328 M",401,38)
     {
      Outlier("General",0.05);
     };
     R_Date("AA-7693 M",315,40)
     {
      Outlier("General",0.05);
     };
     R_Date("AA-AA-7417 M",290,37)
     {
      Outlier("General",0.05);
     };
     Interval("Interval Briggs Run Pits",U(0,100));
     Date("Date Briggs Run Pits");
    };
    Boundary("End Briggs Run",Date(U(1150,1635)));
   };
  };
 };
 Sequence ("Overlapping but Ordered 11 Site Phase Group")
 {
  Date("=Date Second Woods");
  Date("=Date Getman");
  Date("=Date Elwood");
  Date("=Date Smith-Pagerie");
  Date("=Date Otstungo");
  Date("=Date Klock");
  Phase()
  {
   Date("=Date Cayadutta Midden");
   Date("=Date Garoga");
  };
```

```
  Date("=Date Wormuth");
  Phase ()
  {
   Date("=Date Palatine Bridge Pits");
   Date("=Date Briggs Run Pits");
  };
 };
};
```
