# Supplementary material for: Radiocarbon, Bayesian chronological modeling and early European metal circulation in the sixteenth-century AD Mohawk River Valley, USA
Source: PLoS One. 2019 Dec 16;14(12):e0226334. doi: 10.1371/journal.pone.0226334 (PMC6913979; doi:10.1371/journal.pone.0226334)
Supplement: S4 File — Example of the run of Model 1 in Table 3 with the best Amodel (55.5) and Aoverall (53.1) values. Unmodelled results are the individual calibrated ranges for the samples (68.2% and 95.4% probability). Modelled results are after applying the model, results are 68.2% hpd and 95.4% hpd. A = OxCal Agreement index value (should be above 60 if data agree with model), C = OxCal Convergence value (should be ≥95). (DOCX) [file pone.0226334.s004.docx]

**S4 File. Table of results from Model 1 and Figures (part 1 and part 2) showing the results.** Example of the run of Model 1 in Table 3 with the best A_model_ (55.5) and A_overall_ (53.1) values. Unmodelled results are the individual calibrated ranges for the samples (68.2% and 95.4% probability). Modelled results are after applying the model, results are 68.2% hpd and 95.4% hpd. A = OxCal Agreement index value (should be above 60 if data agree with model), C = OxCal Convergence value (should be ≥95).

| **Name** | **Unmodelled (BC/AD)** | | | | | | **Modelled (BC/AD)** | | | | | |  | |
| --- | --- | --- | --- | --- | --- | --- | --- | --- | --- | --- | --- | --- | --- | --- |
|  | ***from*** | ***to*** | ***%*** | ***from*** | ***to*** | ***%*** | ***from*** | ***to*** | ***%*** | ***from*** | ***to*** | ***%*** | ***A*** | ***C*** |
| **Outlier_Model General** |  |  |  |  |  |  | -15 | 15 | 68.2 | -104 | 104 | 95.4 |  | 99.9 |
| **T(5)** | -1.135 | 1.135 | 68.2 | -2.65 | 2.65 | 95.4 |  |  |  |  |  |  |  | 99.8 |
| **U(0,4)** | 3.99E-17 | 4 | 68.2 | 3.99E-17 | 4 | 95.4 | 5.38E-17 | 1.436 | 68.2 | 5.38E-17 | 2.308 | 95.4 | 100 | 99.2 |
| **Outlier_Model Charcoal** |  |  |  |  |  |  | -73 | -1 | 68.2 | -203 | 1 | 95.4 |  | 99.9 |
| **Exp(1,-10,0)** | -1.24 | -0.05 | 68.2 | -3.18 | -0.05 | 95.4 |  |  |  |  |  |  |  | 100 |
| **U(0,3)** | 2.21E-17 | 3 | 68.2 | 2.21E-17 | 3 | 95.4 | 1.578 | 2.055 | 68.2 | 1.275 | 2.247 | 95.4 | 100 | 99.1 |
| **Outlier_Model SSimple** |  |  |  |  |  |  | -18 | 103 | 68.2 | -59 | 131 | 95.4 |  | 99.8 |
| **N(0,2)** | -2.04 | 2.04 | 68.2 | -4 | 4 | 95.4 |  |  |  |  |  |  |  | 100 |
| **Phase** |  |  |  |  |  |  |  |  |  |  |  |  |  |  |
| **Sequence** |  |  |  |  |  |  |  |  |  |  |  |  |  |  |
| **Boundary Start Snell Pits** | 1150 | 1635 | 68.2 | 1150 | 1635 | 95.4 | 1271 | 1285 | 68.2 | 1258 | 1290 | 95.4 | 100 | 99.9 |
| **U(1150,1635)** | 1150 | 1635 | 68.2 | 1150 | 1635 | 95.4 |  |  |  |  |  |  |  |  |
| **Phase Snell Pits** |  |  |  |  |  |  |  |  |  |  |  |  |  |  |
| **R_Date M-28 charred wood** | 21 | 657 | 68.2 | -377 | 963 | 95.4 | 1278 | 1287 | 68.2 | 1268 | 1296 | 95.4 | 11.2 | 99.9 |
| **R_Date M-178 charred wood** | 657 | 1028 | 68.2 | 434 | 1256 | 95.4 | 1278 | 1287 | 68.2 | 1268 | 1296 | 95.4 | 45.6 | 99.9 |
| **R_Date M-492 charred wood** | 1025 | 1390 | 68.2 | 769 | 1484 | 95.4 | 1278 | 1287 | 68.2 | 1269 | 1297 | 95.4 | 137.2 | 99.9 |
| **R_Date UCIAMS190544 B** | 1276 | 1286 | 68.2 | 1270 | 1292 | 95.4 | 1279 | 1286 | 68.2 | 1275 | 1290 | 95.4 | 117 | 99.9 |
| **R_Date UCIAMS190542 B** | 1274 | 1288 | 68.2 | 1264 | 1297 | 95.4 | 1279 | 1286 | 68.2 | 1274 | 1291 | 95.4 | 126.2 | 99.9 |
| **R_Date UCIAMS190543 B** | 1275 | 1289 | 68.2 | 1266 | 1378 | 95.4 | 1279 | 1286 | 68.2 | 1274 | 1291 | 95.4 | 131.6 | 99.9 |
| **R_Date ISGS-A0327 M** | 1272 | 1383 | 68.2 | 1258 | 1392 | 95.4 | 1279 | 1287 | 68.2 | 1273 | 1295 | 95.4 | 158.7 | 99.9 |
| **R_Date UCIAMS192977 B** | 1281 | 1295 | 68.2 | 1277 | 1381 | 95.4 | 1280 | 1287 | 68.2 | 1277 | 1292 | 95.4 | 118.7 | 99.9 |
| **Interval Interval Snell** |  |  |  |  |  |  | 0 | 14 | 68.2 | 0 | 38 | 95.4 |  | 99 |
| **Date Snell Pits** |  |  |  |  |  |  | 1278 | 1288 | 68.2 | 1269 | 1297 | 95.4 |  | 99.9 |
| **Boundary End Snell Pits** | 1150 | 1635 | 68.2 | 1150 | 1635 | 95.4 | 1280 | 1295 | 68.2 | 1276 | 1305 | 95.4 | 100 | 99.9 |
| **U(1150,1635)** | 1150 | 1635 | 68.2 | 1150 | 1635 | 95.4 |  |  |  |  |  |  |  |  |
| **Sequence** |  |  |  |  |  |  |  |  |  |  |  |  |  |  |
| **Boundary Start Pethick** | 1150 | 1635 | 68.2 | 1150 | 1635 | 95.4 | 1270 | 1378 | 68.2 | 1219 | 1382 | 95.4 | 100 | 98.5 |
| **U(1150,1635)** | 1150 | 1635 | 68.2 | 1150 | 1635 | 95.4 |  |  |  |  |  |  |  |  |
| **Phase Pethick hearths or small pits** |  |  |  |  |  |  |  |  |  |  |  |  |  |  |
| **R_Date Beta 199857 C** | 1219 | 1383 | 68.2 | 1161 | 1398 | 95.4 | 1333 | 1402 | 68.2 | 1270 | 1428 | 95.4 | 103 | 99.5 |
| **R_Date Beta 198540 C** | 1266 | 1396 | 68.2 | 1188 | 1432 | 95.4 | 1342 | 1402 | 68.2 | 1282 | 1436 | 95.4 | 111.3 | 99.6 |
| **R_Date Beta 211490 C** | 1318 | 1419 | 68.2 | 1301 | 1433 | 95.4 | 1343 | 1394 | 68.2 | 1315 | 1443 | 95.4 | 97.6 | 99.5 |
| **R_Date Beta 22779 C** | 1318 | 1419 | 68.2 | 1301 | 1433 | 95.4 | 1342 | 1394 | 68.2 | 1316 | 1443 | 95.4 | 97.6 | 99.5 |
| **R_Date UCIAMS218494 M** | 1283 | 1377 | 68.2 | 1279 | 1384 | 95.4 | 1290 | 1382 | 68.2 | 1282 | 1386 | 95.4 | 73.1 | 99.8 |
| **R_Date UCIAMS218495 M** | 1285 | 1379 | 68.2 | 1280 | 1385 | 95.4 | 1366 | 1383 | 68.2 | 1283 | 1386 | 95.4 | 85.8 | 99.8 |
| **R_Date UCIAMS218496 M** | 1314 | 1398 | 68.2 | 1302 | 1407 | 95.4 | 1339 | 1400 | 68.2 | 1312 | 1404 | 95.4 | 87.1 | 99.4 |
| **Interval Interval Pethick** |  |  |  |  |  |  | 0 | 109 | 68.2 | 0 | 265 | 95.4 |  | 97.9 |
| **Date Pethick** |  |  |  |  |  |  | 1337 | 1402 | 68.2 | 1263 | 1451 | 95.4 |  | 99.6 |
| **Boundary End Pethick** | 1150 | 1635 | 68.2 | 1150 | 1635 | 95.4 | 1369 | 1425 | 68.2 | 1335 | 1525 | 95.4 | 100 | 99.5 |
| **U(1150,1635)** | 1150 | 1635 | 68.2 | 1150 | 1635 | 95.4 |  |  |  |  |  |  |  |  |
| **Sequence** |  |  |  |  |  |  |  |  |  |  |  |  |  |  |
| **Boundary Start Second Woods** | 1150 | 1635 | 68.2 | 1150 | 1635 | 95.4 | 1419 | 1466 | 68.2 | 1151 | 1481 | 95.4 | 100 | 99.9 |
| **U(1150,1635)** | 1150 | 1635 | 68.2 | 1150 | 1635 | 95.4 |  |  |  |  |  |  |  |  |
| **Phase Second Woods** |  |  |  |  |  |  |  |  |  |  |  |  |  |  |
| **R_Combine Feature 2 Shallow Pit Deposit - Assume One Event** | 1452 | 1481 | 68.2 | 1447 | 1615 | 95.4 | 1452 | 1473 | 68.2 | 1447 | 1490 | 95.4 | 116.4 | 99.9 |
| **R_Date UCIAMS190546 B Feature 2** | 1448 | 1468 | 68.2 | 1443 | 1607 | 95.4 |  |  |  |  |  |  |  |  |
| **R_Date UCIAMS190547 B Feature 2** | 1458 | 1614 | 68.2 | 1451 | 1618 | 95.4 |  |  |  |  |  |  |  |  |
| **R_Date UCIAMS190536 M** | 1443 | 1459 | 68.2 | 1438 | 1475 | 95.4 | 1446 | 1463 | 68.2 | 1440 | 1477 | 95.4 | 91 | 99.9 |
| **R_Date UCIAMS190535 M** | 1453 | 1615 | 68.2 | 1447 | 1620 | 95.4 | 1452 | 1478 | 68.2 | 1447 | 1510 | 95.4 | 118 | 99.9 |
| **Date Second Woods** |  |  |  |  |  |  | 1440 | 1488 | 68.2 | 1362 | 1565 | 95.4 |  | 99.8 |
| **Interval Interval Second Woods** |  |  |  |  |  |  | 0 | 88 | 68.2 | 0 | 276 | 95.4 |  | 99 |
| **Boundary End Second Woods** | 1150 | 1635 | 68.2 | 1150 | 1635 | 95.4 | 1455 | 1513 | 68.2 | 1451 | 1630 | 95.4 | 100 | 99.9 |
| **U(1150,1635)** | 1150 | 1635 | 68.2 | 1150 | 1635 | 95.4 |  |  |  |  |  |  |  |  |
| **Sequence** |  |  |  |  |  |  |  |  |  |  |  |  |  |  |
| **Boundary Start Elwood** | 1150 | 1635 | 68.2 | 1150 | 1635 | 95.4 | 1433 | 1471 | 68.2 | 1378 | 1606 | 95.4 | 100 | 99.8 |
| **U(1150,1635)** | 1150 | 1635 | 68.2 | 1150 | 1635 | 95.4 |  |  |  |  |  |  |  |  |
| **Phase Elwood** |  |  |  |  |  |  |  |  |  |  |  |  |  |  |
| **R_Date UCIAMS190554 B Pit** | 1442 | 1464 | 68.2 | 1436 | 1606 | 95.4 | 1447 | 1473 | 68.2 | 1439 | 1491 | 95.4 | 79.3 | 99.8 |
| **R_Date AA-7410 M midden** | 1436 | 1617 | 68.2 | 1421 | 1634 | 95.3 | 1451 | 1488 | 68.2 | 1432 | 1611 | 95.4 | 130.3 | 99.8 |
| **R_Date AA-6425 M midden** | 1447 | 1621 | 68.2 | 1440 | 1636 | 95.4 | 1453 | 1491 | 68.2 | 1437 | 1613 | 95.4 | 121 | 99.8 |
| **R_Date UCIAMS190552 B Hearth** | 1455 | 1610 | 68.2 | 1449 | 1617 | 95.4 | 1458 | 1485 | 68.2 | 1450 | 1512 | 95.4 | 115.4 | 99.8 |
| **R_Date UCIAMS190553 B Hearth** | 1462 | 1616 | 68.2 | 1451 | 1630 | 95.4 | 1458 | 1490 | 68.2 | 1450 | 1607 | 95.4 | 112.5 | 99.8 |
| **R_Date AA-7697 M midden** | 1517 | 1660 | 68.2 | 1463 | ... | 95.4 | 1456 | 1502 | 68.2 | 1448 | 1568 | 95.4 | 55.8 | 99.7 |
| **Date Elwood** |  |  |  |  |  |  | 1450 | 1496 | 68.2 | 1419 | 1612 | 95.4 |  | 99.7 |
| **Interval Interval Elwood** |  |  |  |  |  |  | 0 | 80 | 68.2 | 0 | 191 | 95.4 |  | 98.7 |
| **Boundary End Elwood** | 1150 | 1635 | 68.2 | 1150 | 1635 | 95.4 | 1465 | 1528 | 68.2 | 1457 | 1630 | 95.4 | 100 | 99.8 |
| **U(1150,1635)** | 1150 | 1635 | 68.2 | 1150 | 1635 | 95.4 |  |  |  |  |  |  |  |  |
| **Sequence** |  |  |  |  |  |  |  |  |  |  |  |  |  |  |
| **Boundary Start Getman** | 1150 | 1635 | 68.2 | 1150 | 1635 | 95.4 | 1413 | 1439 | 68.2 | 1373 | 1450 | 95.4 | 100 | 99.5 |
| **U(1150,1635)** | 1150 | 1635 | 68.2 | 1150 | 1635 | 95.4 |  |  |  |  |  |  |  |  |
| **Phase Getman** |  |  |  |  |  |  |  |  |  |  |  |  |  |  |
| **R_Date M-783 Charcoal context not stated TPQ** | 1266 | 1609 | 68.2 | 1057 | 1663 | 95.4 | 1431 | 1490 | 68.2 | 1406 | 1534 | 95.4 | 124.9 | 99.1 |
| **R_Date UCIAMS190557 House 3 Hearth B** | 1435 | 1448 | 68.2 | 1424 | 1457 | 95.4 | 1436 | 1449 | 68.2 | 1426 | 1461 | 95.4 | 103 | 99.7 |
| **R_Combine Feature 28 Pit Event** | 1449 | 1473 | 68.2 | 1445 | 1609 | 95.4 | 1450 | 1472 | 68.2 | 1446 | 1489 | 95.4 | 105.9 | 99.8 |
| **R_Date UCIAMS190558 House 3 B Pit** | 1449 | 1472 | 68.2 | 1444 | 1609 | 95.4 |  |  |  |  |  |  |  |  |
| **R_Date UCIAMS218481 House 3 M Pit** | 1450 | 1477 | 68.2 | 1445 | 1614 | 95.4 |  |  |  |  |  |  |  |  |
| **R_Date UCIAMS218482 General Midden M** | 1448 | 1468 | 68.2 | 1443 | 1607 | 95.4 | 1448 | 1468 | 68.2 | 1444 | 1485 | 95.4 | 104.6 | 99.8 |
| **R_Date UCIAMS218480 House 5 Pit M** | 1449 | 1472 | 68.2 | 1444 | 1609 | 95.4 | 1449 | 1471 | 68.2 | 1445 | 1488 | 95.4 | 106.7 | 99.8 |
| **R_Date UCIAMS192976 House 1 Pit B** | 1424 | 1438 | 68.2 | 1417 | 1443 | 95.4 | 1428 | 1441 | 68.2 | 1418 | 1447 | 95.4 | 96.8 | 99.7 |
| **R_Date UCIAMS192975 Pit B** | 1478 | 1619 | 68.2 | 1466 | 1631 | 95.4 | 1470 | 1506 | 68.2 | 1455 | 1522 | 95.4 | 97.7 | 99.2 |
| **R_Date UCIAMS190555 Pit B** | 1489 | 1630 | 68.3 | 1469 | 1634 | 95.4 | 1470 | 1509 | 68.2 | 1450 | 1563 | 95.4 | 87.1 | 99 |
| **R_Date UCIAMS190556 Pit B** | 1500 | 1634 | 68.3 | 1487 | 1640 | 95.4 | 1472 | 1515 | 68.2 | 1453 | 1561 | 95.4 | 63.5 | 99 |
| **Interval Interval Getman** |  |  |  |  |  |  | 56 | 119 | 68.2 | 20 | 204 | 95.4 |  | 98.5 |
| **Date Getman** |  |  |  |  |  |  | 1433 | 1496 | 68.2 | 1406 | 1543 | 95.4 |  | 99.1 |
| **Boundary End Getman** | 1150 | 1635 | 68.2 | 1150 | 1635 | 95.4 | 1487 | 1535 | 68.2 | 1464 | 1601 | 95.4 | 100 | 98.7 |
| **U(1150,1635)** | 1150 | 1635 | 68.2 | 1150 | 1635 | 95.4 |  |  |  |  |  |  |  |  |
| **Sequence** |  |  |  |  |  |  |  |  |  |  |  |  |  |  |
| **Boundary Start Smith-Pagerie** | 1150 | 1635 | 68.2 | 1150 | 1635 | 95.4 | 1408 | 1440 | 68.2 | 1362 | 1450 | 95.4 | 100 | 99.9 |
| **U(1150,1635)** | 1150 | 1635 | 68.2 | 1150 | 1635 | 95.4 |  |  |  |  |  |  |  |  |
| **Phase Smith-Pagerie** |  |  |  |  |  |  |  |  |  |  |  |  |  |  |
| **R_Combine Feature 54 Pit H1 - assume one event** | 1463 | 1619 | 68.2 | 1451 | 1632 | 95.4 | 1464 | 1519 | 68.2 | 1447 | 1615 | 95.4 | 103 | 99.8 |
| **R_Date AA-7405 M** | 1423 | 1615 | 68.2 | 1410 | 1632 | 95.4 |  |  |  |  |  |  |  |  |
| **R_Date AA-6419 M** | 1438 | 1618 | 68.2 | 1425 | 1634 | 95.4 |  |  |  |  |  |  |  |  |
| **R_Date UCIAMS218490 M** | 1515 | 1636 | 68.2 | 1480 | 1644 | 95.4 |  |  |  |  |  |  |  |  |
| **R_Date ISGS-A0528 R H1** | 1423 | 1468 | 68.2 | 1408 | 1617 | 95.4 | 1445 | 1515 | 68.2 | 1428 | 1567 | 95.4 | 110.6 | 99.7 |
| **R_Date UCIAMS190566 B H1** | 1458 | 1614 | 68.2 | 1451 | 1618 | 95.4 | 1456 | 1494 | 68.2 | 1450 | 1611 | 95.4 | 102.7 | 99.9 |
| **R_Date UCIAMS190565 B H1** | 1464 | 1616 | 68.2 | 1452 | 1620 | 95.4 | 1464 | 1512 | 68.2 | 1451 | 1614 | 95.4 | 100.9 | 99.9 |
| **R_Date UCIAMS190563 B H1** | 1470 | 1619 | 68.2 | 1456 | 1631 | 95.5 | 1470 | 1518 | 68.2 | 1451 | 1616 | 95.3 | 103.6 | 99.9 |
| **R_Date UCIAMS218491 Material?? H1** | 1642 | 1654 | 68.2 | 1528 | 1789 | 95.3 | 1527 | 1542 | 68.2 | 1488 | 1570 | 95.4 | 7.8 | 99.8 |
| **R_Date UCIAMS218493 M Longhouse 5 Pit** | 1426 | 1440 | 68.2 | 1418 | 1444 | 95.4 | 1430 | 1442 | 68.2 | 1421 | 1447 | 95.4 | 100.1 | 99.9 |
| **R_Date UCIAMS218492 M Longhouse 2 Hearth** | 1485 | 1621 | 68.2 | 1470 | 1632 | 95.4 | 1480 | 1522 | 68.2 | 1462 | 1617 | 95.4 | 102.8 | 99.9 |
| **R_Date UCIAMS190564 B Longhouse 4 Hearth** | 1489 | 1630 | 68.3 | 1469 | 1634 | 95.4 | 1473 | 1527 | 68.2 | 1462 | 1602 | 95.4 | 97.5 | 99.9 |
| **Interval Interval Smith-Pagerie** |  |  |  |  |  |  | 97 | 203 | 68.2 | 75 | 246 | 95.4 |  | 99.6 |
| **Date Smith-Pagerie** |  |  |  |  |  |  | 1435 | 1533 | 68.2 | 1408 | 1601 | 95.4 |  | 99.9 |
| **Boundary End Smith-Pagerie** | 1150 | 1635 | 68.2 | 1150 | 1635 | 95.4 | 1529 | 1639 | 68.2 | 1526 | 1639 | 95.4 | 100 | 99.2 |
| **U(1150,1635)** | 1150 | 1635 | 68.2 | 1150 | 1635 | 95.4 |  |  |  |  |  |  |  |  |
| **Sequence** |  |  |  |  |  |  |  |  |  |  |  |  |  |  |
| **Boundary Start Otstungo** | 1150 | 1635 | 68.2 | 1150 | 1635 | 95.4 | 1431 | 1474 | 68.2 | 1402 | 1605 | 95.4 | 100 | 99.7 |
| **U(1150,1635)** | 1150 | 1635 | 68.2 | 1150 | 1635 | 95.4 |  |  |  |  |  |  |  |  |
| **Phase Otstungo House 1 Midden and Hearths** |  |  |  |  |  |  |  |  |  |  |  |  |  |  |
| **R_Date UCIAMS190551 Hearth B** | 1474 | 1620 | 68.2 | 1458 | 1633 | 95.4 | 1468 | 1603 | 68.2 | 1457 | 1620 | 95.4 | 104.2 | 99.7 |
| **R_Date UCIAMS190549 Hearth B** | 1496 | 1632 | 68.2 | 1484 | 1638 | 95.4 | 1485 | 1585 | 68.2 | 1472 | 1622 | 95.4 | 95.1 | 99.7 |
| **R_Date UCIAMS190550 Hearth B** | 1523 | 1641 | 68.3 | 1516 | 1644 | 95.4 | 1519 | 1569 | 68.2 | 1490 | 1624 | 95.4 | 96.7 | 99.7 |
| **R_Date UCIAMS190548 Hearth B** | 1523 | 1641 | 68.3 | 1516 | 1644 | 95.4 | 1519 | 1569 | 68.2 | 1490 | 1624 | 95.4 | 96.8 | 99.8 |
| **R_Date AA-7400 M** | 1433 | 1616 | 68.2 | 1417 | 1634 | 95.4 | 1449 | 1520 | 68.2 | 1438 | 1619 | 95.4 | 101.1 | 99.6 |
| **R_Date AA-7402 M** | 1435 | 1617 | 68.2 | 1421 | 1634 | 95.4 | 1449 | 1521 | 68.2 | 1440 | 1618 | 95.4 | 103.2 | 99.6 |
| **R_Date AA-6423 M** | 1438 | 1620 | 68.1 | 1427 | 1636 | 95.4 | 1450 | 1607 | 68.2 | 1444 | 1617 | 95.4 | 106.8 | 99.6 |
| **R_Date UCIAMS218483 M** | 1449 | 1472 | 68.2 | 1444 | 1609 | 95.4 | 1452 | 1484 | 68.2 | 1446 | 1614 | 95.4 | 81.8 | 99.4 |
| **R_Date AA-7398 M** | 1447 | 1625 | 68.1 | 1440 | 1638 | 95.4 | 1452 | 1608 | 68.2 | 1449 | 1614 | 95.4 | 107.4 | 99.6 |
| **R_Date UCIAMS218489 M** | 1453 | 1615 | 68.2 | 1447 | 1620 | 95.4 | 1458 | 1512 | 68.2 | 1449 | 1618 | 95.4 | 102.4 | 99.6 |
| **R_Date AA-7401 M** | 1455 | 1628 | 68.2 | 1447 | 1638 | 95.4 | 1455 | 1604 | 68.2 | 1452 | 1614 | 95.4 | 105.9 | 99.6 |
| **R_Date AA-7399 M** | 1476 | 1633 | 68.2 | 1449 | 1646 | 95.4 | 1463 | 1560 | 68.2 | 1454 | 1614 | 95.4 | 104.8 | 99.6 |
| **R_Date UCIAMS218487 M** | 1495 | 1630 | 68.2 | 1478 | 1634 | 95.4 | 1486 | 1596 | 68.2 | 1472 | 1623 | 95.4 | 96.6 | 99.7 |
| **Interval Interval Otstungo** |  |  |  |  |  |  | 74 | 175 | 68.2 | 20 | 203 | 95.4 |  | 99.1 |
| **Date Otstungo** |  |  |  |  |  |  | 1458 | 1553 | 68.2 | 1445 | 1617 | 95.4 |  | 99.6 |
| **Boundary End Otstungo** | 1150 | 1635 | 68.2 | 1150 | 1635 | 95.4 | 1528 | 1633 | 68.2 | 1522 | 1637 | 95.4 | 100 | 99.6 |
| **U(1150,1635)** | 1150 | 1635 | 68.2 | 1150 | 1635 | 95.4 |  |  |  |  |  |  |  |  |
| **Sequence** |  |  |  |  |  |  |  |  |  |  |  |  |  |  |
| **Boundary Start Klock** | 1150 | 1635 | 68.2 | 1150 | 1635 | 95.4 | 1452 | 1515 | 68.2 | 1411 | 1615 | 95.4 | 100 | 98.9 |
| **U(1150,1635)** | 1150 | 1635 | 68.2 | 1150 | 1635 | 95.4 |  |  |  |  |  |  |  |  |
| **Phase Klock** |  |  |  |  |  |  |  |  |  |  |  |  |  |  |
| **R_Combine Feature 84 Pit H1 - assume one event** | 1477 | 1618 | 68.2 | 1461 | 1630 | 95.5 | 1484 | 1604 | 68.2 | 1470 | 1620 | 95.4 | 108.3 | 99.5 |
| **R_Date UCIAMS218474 M** | 1470 | 1616 | 68.2 | 1454 | 1625 | 95.4 |  |  |  |  |  |  |  |  |
| **R_Date ISGS-A0326 M** | 1516 | 1642 | 68.2 | 1472 | 1649 | 95.4 |  |  |  |  |  |  |  |  |
| **R_Date ISGS-A0523 R Pit** | 1415 | 1446 | 68.2 | 1327 | 1474 | 95.4 | 1473 | 1601 | 68.2 | 1443 | 1623 | 95.4 | 98.6 | 99.3 |
| **R_Date UCIAMS218476 M Pit** | 1470 | 1616 | 68.2 | 1454 | 1625 | 95.4 | 1483 | 1605 | 68.2 | 1466 | 1619 | 95.4 | 100.9 | 99.5 |
| **R_Date UCIAMS190559 B Pit** | 1475 | 1617 | 68.2 | 1459 | 1630 | 95.5 | 1484 | 1604 | 68.2 | 1469 | 1620 | 95.4 | 108.8 | 99.5 |
| **R_Date UCIAMS190561 B Pit** | 1499 | 1632 | 68.2 | 1485 | 1635 | 95.4 | 1489 | 1600 | 68.2 | 1481 | 1627 | 95.4 | 95.1 | 99.5 |
| **R_Date UCIAMS218475 M Pit** | 1496 | 1632 | 68.2 | 1484 | 1638 | 95.4 | 1487 | 1600 | 68.2 | 1479 | 1626 | 95.4 | 100 | 99.5 |
| **R_Date UCIAMS218473 B Pit** | 1519 | 1634 | 68.2 | 1493 | 1640 | 95.4 | 1489 | 1597 | 68.2 | 1487 | 1625 | 95.4 | 77.3 | 99.4 |
| **R_Date UCIAMS190562 B Hearth H1** | 1474 | 1620 | 68.2 | 1458 | 1633 | 95.4 | 1484 | 1602 | 68.2 | 1469 | 1623 | 95.3 | 115.1 | 99.5 |
| **R_Date UCIAMS190560 M Hearth H1** | 1519 | 1634 | 68.2 | 1493 | 1640 | 95.4 | 1489 | 1597 | 68.2 | 1487 | 1625 | 95.4 | 77.2 | 99.4 |
| **R_Date AA-6418 M Hearth H7** | 1495 | 1644 | 68.1 | 1448 | 1795 | 95.4 | 1483 | 1602 | 68.2 | 1476 | 1622 | 95.4 | 106.3 | 99.5 |
| **R_Date AA-7404 M Hearth H7** | 1314 | 1447 | 68.2 | 1284 | 1616 | 95.4 | 1477 | 1610 | 68.2 | 1427 | 1624 | 95.4 | 21 | 99.4 |
| **Interval Interval Klock** |  |  |  |  |  |  | 0 | 68 | 68.2 | 0 | 187 | 95.4 |  | 98.6 |
| **Date Klock** |  |  |  |  |  |  | 1480 | 1604 | 68.2 | 1467 | 1624 | 95.4 |  | 99.5 |
| **Boundary End Klock** | 1150 | 1635 | 68.2 | 1150 | 1635 | 95.4 | 1500 | 1632 | 68.2 | 1493 | 1637 | 95.4 | 100 | 99.6 |
| **U(1150,1635)** | 1150 | 1635 | 68.2 | 1150 | 1635 | 95.4 |  |  |  |  |  |  |  |  |
| **Sequence** |  |  |  |  |  |  |  |  |  |  |  |  |  |  |
| **Boundary Start Cayadutta Midden** | 1150 | 1635 | 68.2 | 1150 | 1635 | 95.4 | 1353 | 1450 | 68.2 | 1249 | 1602 | 95.4 | 100 | 99.6 |
| **U(1150,1635)** | 1150 | 1635 | 68.2 | 1150 | 1635 | 95.4 |  |  |  |  |  |  |  |  |
| **Phase Cayadutta Midden** |  |  |  |  |  |  |  |  |  |  |  |  |  |  |
| **R_Date AA-7689 M** | 1314 | 1426 | 68.2 | 1296 | 1439 | 95.4 | 1389 | 1451 | 68.2 | 1323 | 1615 | 95.4 | 72.9 | 99.6 |
| **R_Date AA-7690 M** | 1431 | 1618 | 68.2 | 1416 | 1635 | 95.4 | 1435 | 1519 | 68.2 | 1420 | 1617 | 95.3 | 106.3 | 99.8 |
| **R_Date AA-7407 M** | 1454 | 1628 | 68.2 | 1446 | 1639 | 95.4 | 1448 | 1580 | 68.2 | 1444 | 1613 | 95.4 | 103.5 | 99.8 |
| **R_Date AA-6421 M** | 1513 | 1650 | 68.2 | 1462 | 1795 | 95.4 | 1487 | 1571 | 68.2 | 1457 | 1619 | 95.4 | 101.2 | 99.8 |
| **R_Date UCIAMS-205965 M** | 1474 | 1620 | 68.2 | 1458 | 1633 | 95.4 | 1467 | 1603 | 68.2 | 1455 | 1620 | 95.4 | 101.8 | 99.8 |
| **R_Date UCIAMS-205966 M** | 1495 | 1630 | 68.2 | 1478 | 1634 | 95.4 | 1487 | 1595 | 68.2 | 1472 | 1623 | 95.4 | 96.7 | 99.8 |
| **R_Date UCIAMS-205967 M** | 1522 | 1640 | 68.2 | 1499 | 1643 | 95.4 | 1519 | 1572 | 68.2 | 1490 | 1622 | 95.4 | 96.9 | 99.8 |
| **R_Date UCIAMS-205968 M** | 1458 | 1614 | 68.2 | 1451 | 1618 | 95.4 | 1457 | 1511 | 68.2 | 1451 | 1616 | 95.4 | 100.3 | 99.8 |
| **Interval Interval Cayadutta Midden** |  |  |  |  |  |  | 112 | 258 | 68.2 | 0 | 335 | 95.4 |  | 99.4 |
| **Date Cayadutta Midden** |  |  |  |  |  |  | 1428 | 1564 | 68.2 | 1372 | 1624 | 95.4 |  | 99.8 |
| **Boundary End Cayadutta Midden** | 1150 | 1635 | 68.2 | 1150 | 1635 | 95.4 | 1571 | 1637 | 68.2 | 1521 | 1637 | 95.4 | 100 | 99.8 |
| **U(1150,1635)** | 1150 | 1635 | 68.2 | 1150 | 1635 | 95.4 |  |  |  |  |  |  |  |  |
| **Sequence** |  |  |  |  |  |  |  |  |  |  |  |  |  |  |
| **Boundary Start Garoga** | 1150 | 1635 | 68.2 | 1150 | 1635 | 95.4 | 1311 | 1412 | 68.2 | 1147 | ... | 95.4 | 100 | 99.8 |
| **U(1150,1635)** | 1150 | 1635 | 68.2 | 1150 | 1635 | 95.4 |  |  |  |  |  |  |  |  |
| **Phase Garoga** |  |  |  |  |  |  |  |  |  |  |  |  |  |  |
| **R_Date Y-1381 charred wood** | 1287 | 1406 | 68.2 | 1193 | 1463 | 95.4 | 1370 | 1493 | 68.2 | 1315 | 1574 | 95.4 | 104.7 | 99.7 |
| **R_Combine Feature 2 Pit H9** | 1486 | 1622 | 68.3 | 1472 | 1632 | 95.4 | 1484 | 1600 | 68.2 | 1468 | 1623 | 95.4 | 97.3 | 99.8 |
| **R_Date AA-7695 M F2 Pit H9** | 1429 | 1483 | 68.2 | 1413 | 1620 | 95.3 |  |  |  |  |  |  |  |  |
| **R_Date AA-7403 M F2 Pit H9** | 1432 | 1619 | 68.2 | 1418 | 1636 | 95.4 |  |  |  |  |  |  |  |  |
| **R_Date UCIAMS218478 M F2 Pit H9** | 1491 | 1628 | 68.2 | 1472 | 1633 | 95.4 |  |  |  |  |  |  |  |  |
| **R_Date UCIAMS190537 M F2 Pit H9** | 1496 | 1632 | 68.2 | 1484 | 1638 | 95.4 |  |  |  |  |  |  |  |  |
| **R_Date AA-6417 M F2 Pit H9** | 1513 | 1650 | 68.2 | 1462 | 1795 | 95.4 |  |  |  |  |  |  |  |  |
| **R_Date AA-8370 M H9 Pit** | 1311 | 1408 | 68.2 | 1297 | 1418 | 95.4 | 1346 | 1427 | 68.2 | 1299 | 1443 | 95.4 | 86.9 | 99.7 |
| **R_Date ISGS-A0522 R H5 Pit** | 1431 | 1608 | 68.2 | 1417 | 1627 | 95.3 | 1452 | 1535 | 68.2 | 1437 | 1600 | 95.4 | 112.9 | 99.6 |
| **R_Date UCIAMS190540 M H4 Pit** | 1489 | 1630 | 68.3 | 1469 | 1634 | 95.4 | 1480 | 1595 | 68.2 | 1466 | 1620 | 95.4 | 98 | 99.8 |
| **R_Date UCIAMS190539 M H2 Pit** | 1521 | 1636 | 68.2 | 1491 | 1643 | 95.4 | 1515 | 1579 | 68.2 | 1486 | 1624 | 95.4 | 99.8 | 99.8 |
| **R_Date UCIAMS218479 M H12 Pit** | 1522 | 1640 | 68.2 | 1499 | 1643 | 95.4 | 1522 | 1570 | 68.2 | 1492 | 1597 | 95.4 | 102.7 | 99.9 |
| **R_Combine F184 Pit btw H1&Stockade** | 1522 | 1636 | 68.1 | 1494 | 1643 | 95.4 | 1519 | 1575 | 68.2 | 1489 | 1621 | 95.4 | 100.8 | 99.8 |
| **R_Date UCIAMS190538 M F184 Pit** | 1523 | 1643 | 68.2 | 1499 | 1647 | 95.4 |  |  |  |  |  |  |  |  |
| **R_Date UCIAMS218477 M F184 Pit** | 1500 | 1634 | 68.3 | 1487 | 1640 | 95.4 |  |  |  |  |  |  |  |  |
| **Interval Interval Garoga** |  |  |  |  |  |  | 178 | 306 | 68.2 | 110 | 485 | 95.4 |  | 99.7 |
| **Date Garoga** |  |  |  |  |  |  | 1402 | 1568 | 68.2 | 1319 | 1620 | 95.4 |  | 99.8 |
| **Boundary End Garoga** | 1150 | 1635 | 68.2 | 1150 | 1635 | 95.4 | 1583 | 1635 | 68.2 | 1545 | 1637 | 95.4 | 100 | 99.9 |
| **U(1150,1635)** | 1150 | 1635 | 68.2 | 1150 | 1635 | 95.4 |  |  |  |  |  |  |  |  |
| **Sequence** |  |  |  |  |  |  |  |  |  |  |  |  |  |  |
| **Boundary Start Wormuth** | 1150 | 1635 | 68.2 | 1150 | 1635 | 95.4 | 1407 | 1601 | 68.2 | 1321 | 1618 | 95.4 | 100 | 99.3 |
| **U(1150,1635)** | 1150 | 1635 | 68.2 | 1150 | 1635 | 95.4 |  |  |  |  |  |  |  |  |
| **Phase Wormuth** |  |  |  |  |  |  |  |  |  |  |  |  |  |  |
| **R_Date AA-6416 M** | 1445 | 1620 | 68.2 | 1438 | 1636 | 95.4 | 1466 | 1615 | 68.2 | 1448 | 1621 | 95.4 | 102.6 | 99.5 |
| **R_Date UCIAMS192700 M Pit** | 1475 | 1617 | 68.2 | 1459 | 1630 | 95.5 | 1479 | 1614 | 68.2 | 1463 | 1620 | 95.3 | 101.1 | 99.5 |
| **R_Date AA-6065 M** | 1491 | 1632 | 68.2 | 1473 | 1636 | 95.4 | 1485 | 1603 | 68.2 | 1473 | 1621 | 95.4 | 101.9 | 99.6 |
| **R_Date DIC-1176 Lowest Level Midden Charcoal** | 1315 | 1422 | 68.2 | 1298 | 1437 | 95.4 | 1443 | 1614 | 68.2 | 1401 | 1625 | 95.4 | 98.4 | 99.2 |
| **R_Date DIC-1177 Higher Up Midden Charcoal** | 1523 | ... | 68.3 | 1485 | ... | 95.4 | 1504 | 1607 | 68.2 | 1486 | 1622 | 95.4 | 61.3 | 99.5 |
| **R_Date DIC-1178 Fill of Burial #2** | 1434 | 1618 | 68.2 | 1419 | 1635 | 95.4 | 1474 | 1614 | 68.2 | 1458 | 1623 | 95.4 | 110 | 99.5 |
| **Interval Interval Wormuth** |  |  |  |  |  |  | 0 | 148 | 68.2 | 0 | 281 | 95.4 |  | 99.2 |
| **Date Wormuth** |  |  |  |  |  |  | 1478 | 1614 | 68.3 | 1418 | 1629 | 95.4 |  | 99.4 |
| **Boundary End Wormuth** | 1150 | 1635 | 68.2 | 1150 | 1635 | 95.4 | 1521 | 1637 | 68.2 | 1506 | 1637 | 95.4 | 100 | 99.6 |
| **U(1150,1635)** | 1150 | 1635 | 68.2 | 1150 | 1635 | 95.4 |  |  |  |  |  |  |  |  |


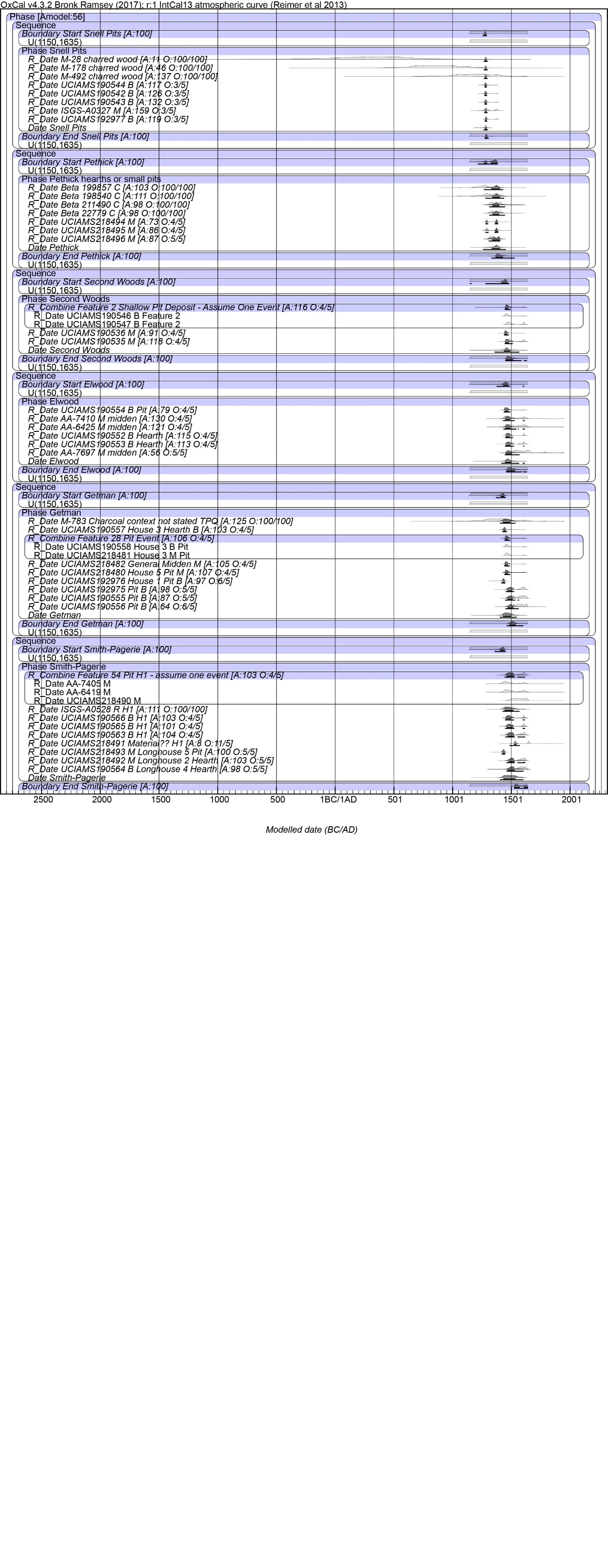


Model 1 Part 1


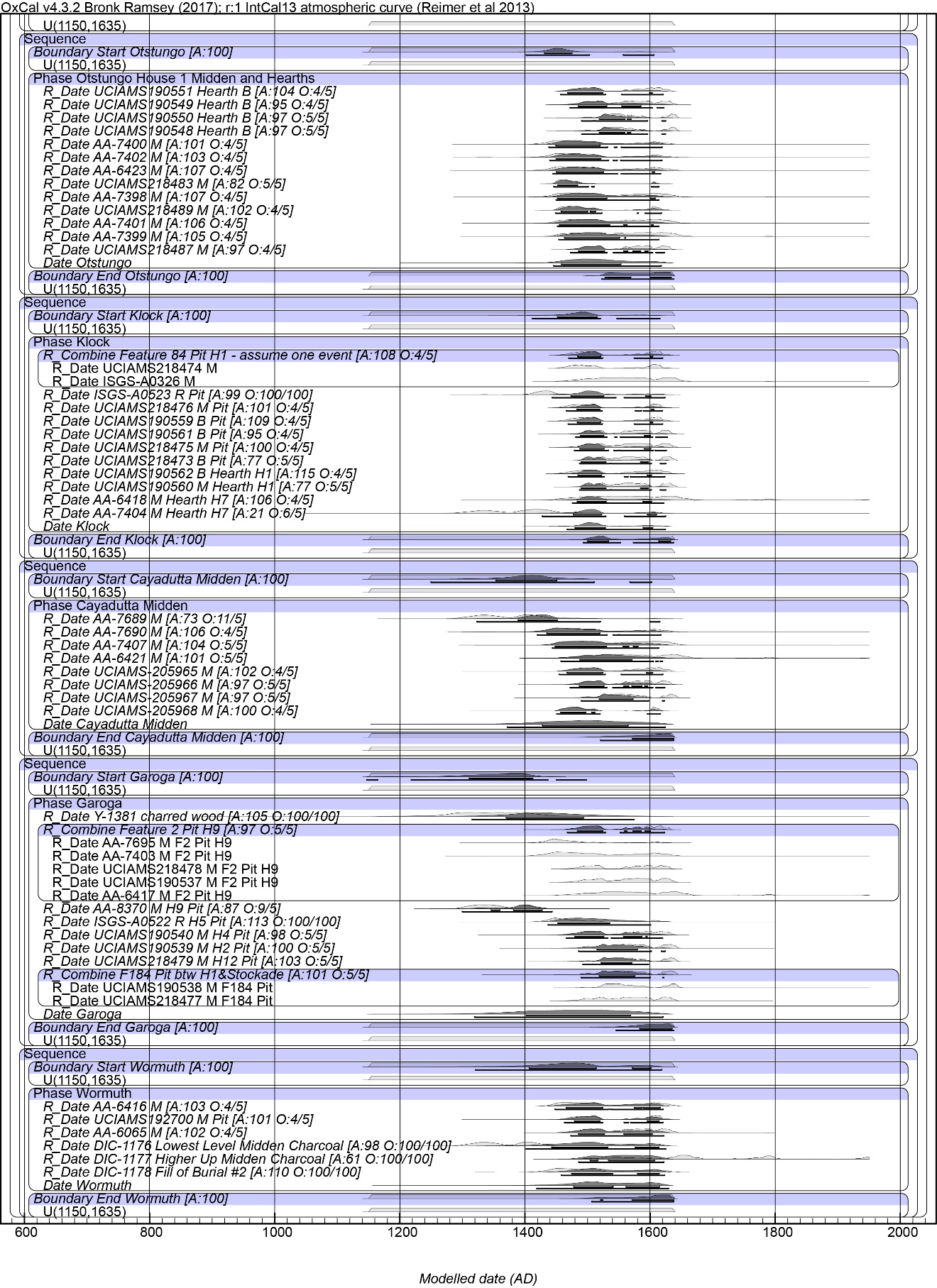


Model 1 Part 2.

Figures (Parts 1 and 2) show Model 1 (for details see Table above) and indicate the structure of the model. The light grey histograms are the non-modelled probability ranges. The black histograms are the modelled probability ranges. The lines under each black histogram indicate the 68.2% and 95.4% hpd ranges. A values are OxCal individual Agreement values. These should be over 60 where the modelled date agrees with the model. O values are from the Outlier models. The first value is the posterior value and the second is the prior value. For dates on charcoal samples the values are 100:100. For the dates on short-lived samples a value above 5 (5%) indicates a possible outlier. As discussed in the text (Methods) we considered only posterior values ≥10 (a 10% possibility of being an outlier) as outliers of concern in this modelling work.
