# Supplementary material for: Radiocarbon, Bayesian chronological modeling and early European metal circulation in the sixteenth-century AD Mohawk River Valley, USA
Source: PLoS One. 2019 Dec 16;14(12):e0226334. doi: 10.1371/journal.pone.0226334 (PMC6913979; doi:10.1371/journal.pone.0226334)
Supplement: S5 File — Example of a run of Model 2 with good Amodel (89.8) and Aoverall (83.8) values. Unmodelled results are the individual calibrated ranges for the samples (68.2% and 95.4% probability). Modelled results are after applying the model, results are 68.2% hpd and 95.4% hpd. A = OxCal Agreement index value (should be above 60 if data agree with model), C = OxCal Convergence value (should be ≥95). (DOCX) [file pone.0226334.s005.docx]

**S5 File. Table of results from Model 2 with 0-120 calendar years uniform probability constraint on an Interval query for each site Phase and Figures (part 1 and part 2) showing the results.** Example of a run of Model 2 with good A_model_ (89.8) and A_overall_ (83.8) values. Unmodelled results are the individual calibrated ranges for the samples (68.2% and 95.4% probability). Modelled results are after applying the model, results are 68.2% hpd and 95.4% hpd. A = OxCal Agreement index value (should be above 60 if data agree with model), C = OxCal Convergence value (should be ≥95).

| **Name** | **Unmodelled (BC/AD)** | | | | | | **Modelled (BC/AD)** | | | | | |  |  |
| --- | --- | --- | --- | --- | --- | --- | --- | --- | --- | --- | --- | --- | --- | --- |
|  | ***from*** | ***to*** | ***%*** | ***from*** | ***to*** | ***%*** | ***from*** | ***to*** | ***%*** | ***from*** | ***to*** | ***%*** | ***A*** | ***C*** |
| **Outlier_Model General** |  |  |  |  |  |  | -9 | 9 | 68.2 | -50 | 54 | 95.4 |  | 100 |
| **T(5)** | -1.135 | 1.135 | 68.2 | -2.65 | 2.65 | 95.4 |  |  |  |  |  |  |  | 99.9 |
| **U(0,4)** | 3.99E-17 | 4 | 68.2 | 3.99E-17 | 4 | 95.4 | 5.38E-17 | 1.168 | 68.2 | 5.38E-17 | 2.3 | 95.4 | 100 | 99.6 |
| **Outlier_Model Charcoal** |  |  |  |  |  |  | -107 | -3 | 68.2 | -277 | -2 | 95.4 |  | 99.9 |
| **Exp(1,-10,0)** | -1.24 | -0.05 | 68.2 | -3.18 | -0.05 | 95.4 |  |  |  |  |  |  |  | 100 |
| **U(0,3)** | 2.21E-17 | 3 | 68.2 | 2.21E-17 | 3 | 95.4 | 1.791 | 2.136 | 68.2 | 1.587 | 2.316 | 95.4 | 100 | 98 |
| **Outlier_Model SSimple** |  |  |  |  |  |  | -29 | 32 | 68.2 | -69 | 109 | 95.4 |  | 99.9 |
| **N(0,2)** | -2.04 | 2.04 | 68.2 | -4 | 4 | 95.4 |  |  |  |  |  |  |  | 100 |
| **Phase** |  |  |  |  |  |  |  |  |  |  |  |  |  |  |
| **Sequence** |  |  |  |  |  |  |  |  |  |  |  |  |  |  |
| **Boundary Start Snell Pits** | 1150 | 1635 | 68.2 | 1150 | 1635 | 95.4 | 1270 | 1285 | 68.2 | 1256 | 1290 | 95.4 | 100 | 99.7 |
| **U(1150,1635)** | 1150 | 1635 | 68.2 | 1150 | 1635 | 95.4 |  |  |  |  |  |  |  |  |
| **Phase Snell Pits** |  |  |  |  |  |  |  |  |  |  |  |  |  |  |
| **R_Date M-28 charred wood** | 21 | 657 | 68.2 | -377 | 963 | 95.4 | 1278 | 1288 | 68.2 | 1267 | 1298 | 95.4 | 21.4 | 99.8 |
| **R_Date M-178 charred wood** | 657 | 1028 | 68.2 | 434 | 1256 | 95.4 | 1278 | 1288 | 68.2 | 1267 | 1298 | 95.4 | 59 | 99.8 |
| **R_Date M-492 charred wood** | 1025 | 1390 | 68.2 | 769 | 1484 | 95.4 | 1278 | 1288 | 68.2 | 1267 | 1299 | 95.4 | 134.3 | 99.8 |
| **R_Date UCIAMS190544 B** | 1276 | 1286 | 68.2 | 1270 | 1292 | 95.4 | 1279 | 1286 | 68.2 | 1274 | 1290 | 95.4 | 115.4 | 99.9 |
| **R_Date UCIAMS190542 B** | 1274 | 1288 | 68.2 | 1264 | 1297 | 95.4 | 1278 | 1286 | 68.2 | 1273 | 1291 | 95.4 | 124.1 | 99.8 |
| **R_Date UCIAMS190543 B** | 1275 | 1289 | 68.2 | 1266 | 1378 | 95.4 | 1279 | 1287 | 68.2 | 1274 | 1292 | 95.4 | 129.5 | 99.9 |
| **R_Date ISGS-A0327 M** | 1272 | 1383 | 68.2 | 1258 | 1392 | 95.4 | 1279 | 1288 | 68.2 | 1272 | 1296 | 95.4 | 156.8 | 99.8 |
| **R_Date UCIAMS192977 B** | 1281 | 1295 | 68.2 | 1277 | 1381 | 95.4 | 1280 | 1288 | 68.2 | 1277 | 1293 | 95.4 | 119.1 | 99.7 |
| **Interval Interval Snell** | 0 | 120 | 68.2 | 0 | 120 | 95.4 | 0 | 16 | 68.2 | 0 | 43 | 95.4 | 100 | 99.1 |
| **U(0,120)** | 1.28E-15 | 120 | 68.2 | 1.28E-15 | 120 | 95.4 |  |  |  |  |  |  |  |  |
| **Date Snell Pits** |  |  |  |  |  |  | 1278 | 1288 | 68.2 | 1267 | 1299 | 95.4 |  | 99.8 |
| **Boundary End Snell Pits** | 1150 | 1635 | 68.2 | 1150 | 1635 | 95.4 | 1281 | 1295 | 68.2 | 1277 | 1308 | 95.4 | 100 | 99.5 |
| **U(1150,1635)** | 1150 | 1635 | 68.2 | 1150 | 1635 | 95.4 |  |  |  |  |  |  |  |  |
| **Sequence** |  |  |  |  |  |  |  |  |  |  |  |  |  |  |
| **Boundary Start Pethick** | 1150 | 1635 | 68.2 | 1150 | 1635 | 95.4 | 1338 | 1377 | 68.2 | 1273 | 1382 | 95.4 | 100 | 99.6 |
| **U(1150,1635)** | 1150 | 1635 | 68.2 | 1150 | 1635 | 95.4 |  |  |  |  |  |  |  |  |
| **Phase Pethick hearths or small pits** |  |  |  |  |  |  |  |  |  |  |  |  |  |  |
| **R_Date Beta 199857 C** | 1219 | 1383 | 68.2 | 1161 | 1398 | 95.4 | 1355 | 1395 | 68.2 | 1302 | 1419 | 95.4 | 104.1 | 99.9 |
| **R_Date Beta 198540 C** | 1266 | 1396 | 68.2 | 1188 | 1432 | 95.4 | 1356 | 1395 | 68.2 | 1310 | 1422 | 95.4 | 110.4 | 99.9 |
| **R_Date Beta 211490 C** | 1318 | 1419 | 68.2 | 1301 | 1433 | 95.4 | 1354 | 1391 | 68.2 | 1329 | 1421 | 95.4 | 97.8 | 99.8 |
| **R_Date Beta 22779 C** | 1318 | 1419 | 68.2 | 1301 | 1433 | 95.4 | 1354 | 1391 | 68.2 | 1329 | 1421 | 95.4 | 97.8 | 99.9 |
| **R_Date UCIAMS218494 M** | 1283 | 1377 | 68.2 | 1279 | 1384 | 95.4 | 1369 | 1381 | 68.2 | 1286 | 1386 | 95.4 | 62.6 | 99.9 |
| **R_Date UCIAMS218495 M** | 1285 | 1379 | 68.2 | 1280 | 1385 | 95.4 | 1369 | 1381 | 68.2 | 1288 | 1387 | 95.4 | 79.9 | 99.9 |
| **R_Date UCIAMS218496 M** | 1314 | 1398 | 68.2 | 1302 | 1407 | 95.4 | 1347 | 1398 | 68.2 | 1321 | 1405 | 95.4 | 80.5 | 99.7 |
| **Interval Interval Pethick** | 0 | 120 | 68.2 | 0 | 120 | 95.4 | 0 | 64 | 68.2 | 0 | 120 | 95.4 | 100 | 99.1 |
| **U(0,120)** | 1.28E-15 | 120 | 68.2 | 1.28E-15 | 120 | 95.4 |  |  |  |  |  |  |  |  |
| **Date Pethick** |  |  |  |  |  |  | 1354 | 1394 | 68.2 | 1304 | 1423 | 95.4 |  | 99.9 |
| **Boundary End Pethick** | 1150 | 1635 | 68.2 | 1150 | 1635 | 95.4 | 1373 | 1407 | 68.2 | 1363 | 1450 | 95.4 | 100 | 99.6 |
| **U(1150,1635)** | 1150 | 1635 | 68.2 | 1150 | 1635 | 95.4 |  |  |  |  |  |  |  |  |
| **Sequence** |  |  |  |  |  |  |  |  |  |  |  |  |  |  |
| **Boundary Start Second Woods** | 1150 | 1635 | 68.2 | 1150 | 1635 | 95.4 | 1434 | 1469 | 68.2 | 1402 | 1475 | 95.4 | 100 | 99.9 |
| **U(1150,1635)** | 1150 | 1635 | 68.2 | 1150 | 1635 | 95.4 |  |  |  |  |  |  |  |  |
| **Phase Second Woods** |  |  |  |  |  |  |  |  |  |  |  |  |  |  |
| **R_Combine Feature 2 Shallow Pit Deposit - Assume One Event** | 1452 | 1481 | 68.2 | 1447 | 1615 | 95.4 | 1452 | 1470 | 68.2 | 1448 | 1486 | 95.4 | 120.5 | 99.9 |
| **R_Date UCIAMS190546 B Feature 2** | 1448 | 1468 | 68.2 | 1443 | 1607 | 95.4 |  |  |  |  |  |  |  |  |
| **R_Date UCIAMS190547 B Feature 2** | 1458 | 1614 | 68.2 | 1451 | 1618 | 95.4 |  |  |  |  |  |  |  |  |
| **R_Date UCIAMS190536 M** | 1443 | 1459 | 68.2 | 1438 | 1475 | 95.4 | 1447 | 1465 | 68.2 | 1442 | 1478 | 95.4 | 86.3 | 99.9 |
| **R_Date UCIAMS190535 M** | 1453 | 1615 | 68.2 | 1447 | 1620 | 95.4 | 1452 | 1473 | 68.2 | 1447 | 1492 | 95.4 | 120.5 | 99.9 |
| **Date Second Woods** |  |  |  |  |  |  | 1448 | 1476 | 68.2 | 1424 | 1507 | 95.4 |  | 99.9 |
| **Interval Interval Second Woods** | 0 | 120 | 68.2 | 0 | 120 | 95.4 | 0 | 47 | 68.2 | 0 | 100 | 95.4 | 100 | 99.2 |
| **U(0,120)** | 1.28E-15 | 120 | 68.2 | 1.28E-15 | 120 | 95.4 |  |  |  |  |  |  |  |  |
| **Boundary End Second Woods** | 1150 | 1635 | 68.2 | 1150 | 1635 | 95.4 | 1456 | 1493 | 68.2 | 1450 | 1531 | 95.4 | 100 | 99.9 |
| **U(1150,1635)** | 1150 | 1635 | 68.2 | 1150 | 1635 | 95.4 |  |  |  |  |  |  |  |  |
| **Sequence** |  |  |  |  |  |  |  |  |  |  |  |  |  |  |
| **Boundary Start Elwood** | 1150 | 1635 | 68.2 | 1150 | 1635 | 95.4 | 1437 | 1470 | 68.2 | 1409 | 1491 | 95.4 | 100 | 99.9 |
| **U(1150,1635)** | 1150 | 1635 | 68.2 | 1150 | 1635 | 95.4 |  |  |  |  |  |  |  |  |
| **Phase Elwood** |  |  |  |  |  |  |  |  |  |  |  |  |  |  |
| **R_Date UCIAMS190554 B Pit** | 1442 | 1464 | 68.2 | 1436 | 1606 | 95.4 | 1448 | 1474 | 68.2 | 1441 | 1491 | 95.4 | 75.6 | 99.9 |
| **R_Date AA-7410 M midden** | 1436 | 1617 | 68.2 | 1421 | 1634 | 95.3 | 1453 | 1486 | 68.2 | 1438 | 1517 | 95.4 | 133.9 | 99.9 |
| **R_Date AA-6425 M midden** | 1447 | 1621 | 68.2 | 1440 | 1636 | 95.4 | 1455 | 1488 | 68.2 | 1442 | 1521 | 95.4 | 123.8 | 99.9 |
| **R_Date UCIAMS190552 B Hearth** | 1455 | 1610 | 68.2 | 1449 | 1617 | 95.4 | 1459 | 1484 | 68.2 | 1450 | 1509 | 95.4 | 117.2 | 99.9 |
| **R_Date UCIAMS190553 B Hearth** | 1462 | 1616 | 68.2 | 1451 | 1630 | 95.4 | 1459 | 1488 | 68.2 | 1451 | 1514 | 95.4 | 114 | 99.9 |
| **R_Date AA-7697 M midden** | 1517 | 1660 | 68.2 | 1463 | ... | 95.4 | 1458 | 1496 | 68.2 | 1449 | 1535 | 95.4 | 47.3 | 99.9 |
| **Date Elwood** |  |  |  |  |  |  | 1453 | 1491 | 68.2 | 1432 | 1607 | 95.4 |  | 99.9 |
| **Interval Interval Elwood** | 0 | 120 | 68.2 | 0 | 120 | 95.4 | 0 | 62 | 68.2 | 0 | 108 | 95.4 | 100 | 99.6 |
| **U(0,120)** | 1.28E-15 | 120 | 68.2 | 1.28E-15 | 120 | 95.4 |  |  |  |  |  |  |  |  |
| **Boundary End Elwood** | 1150 | 1635 | 68.2 | 1150 | 1635 | 95.4 | 1468 | 1517 | 68.2 | 1457 | 1556 | 95.4 | 100 | 99.8 |
| **U(1150,1635)** | 1150 | 1635 | 68.2 | 1150 | 1635 | 95.4 |  |  |  |  |  |  |  |  |
| **Sequence** |  |  |  |  |  |  |  |  |  |  |  |  |  |  |
| **Boundary Start Getman** | 1150 | 1635 | 68.2 | 1150 | 1635 | 95.4 | 1417 | 1437 | 68.2 | 1398 | 1444 | 95.4 | 100 | 99.9 |
| **U(1150,1635)** | 1150 | 1635 | 68.2 | 1150 | 1635 | 95.4 |  |  |  |  |  |  |  |  |
| **Phase Getman** |  |  |  |  |  |  |  |  |  |  |  |  |  |  |
| **R_Date M-783 Charcoal context not stated TPQ** | 1266 | 1609 | 68.2 | 1057 | 1663 | 95.4 | 1433 | 1491 | 68.2 | 1417 | 1522 | 95.4 | 127.4 | 99.7 |
| **R_Date UCIAMS190557 House 3 Hearth B** | 1435 | 1448 | 68.2 | 1424 | 1457 | 95.4 | 1436 | 1449 | 68.2 | 1426 | 1461 | 95.4 | 103.5 | 99.8 |
| **R_Combine Feature 28 Pit Event** | 1449 | 1473 | 68.2 | 1445 | 1609 | 95.4 | 1450 | 1472 | 68.2 | 1445 | 1489 | 95.4 | 104.5 | 99.6 |
| **R_Date UCIAMS190558 House 3 B Pit** | 1449 | 1472 | 68.2 | 1444 | 1609 | 95.4 |  |  |  |  |  |  |  |  |
| **R_Date UCIAMS218481 House 3 M Pit** | 1450 | 1477 | 68.2 | 1445 | 1614 | 95.4 |  |  |  |  |  |  |  |  |
| **R_Date UCIAMS218482 General Midden M** | 1448 | 1468 | 68.2 | 1443 | 1607 | 95.4 | 1448 | 1468 | 68.2 | 1443 | 1486 | 95.4 | 103.1 | 99.6 |
| **R_Date UCIAMS218480 House 5 Pit M** | 1449 | 1472 | 68.2 | 1444 | 1609 | 95.4 | 1449 | 1471 | 68.2 | 1445 | 1489 | 95.4 | 105.3 | 99.6 |
| **R_Date UCIAMS192976 House 1 Pit B** | 1424 | 1438 | 68.2 | 1417 | 1443 | 95.4 | 1429 | 1441 | 68.2 | 1419 | 1446 | 95.4 | 97.9 | 99.8 |
| **R_Date UCIAMS192975 Pit B** | 1478 | 1619 | 68.2 | 1466 | 1631 | 95.4 | 1472 | 1505 | 68.2 | 1460 | 1520 | 95.4 | 101 | 99.8 |
| **R_Date UCIAMS190555 Pit B** | 1489 | 1630 | 68.3 | 1469 | 1634 | 95.4 | 1473 | 1508 | 68.2 | 1458 | 1523 | 95.4 | 90.2 | 99.8 |
| **R_Date UCIAMS190556 Pit B** | 1500 | 1634 | 68.3 | 1487 | 1640 | 95.4 | 1475 | 1510 | 68.2 | 1462 | 1529 | 95.4 | 63.1 | 99.8 |
| **Interval Interval Getman** | 0 | 120 | 68.2 | 0 | 120 | 95.4 | 74 | 114 | 68.2 | 53 | 120 | 95.4 | 100 | 99.7 |
| **U(0,120)** | 1.28E-15 | 120 | 68.2 | 1.28E-15 | 120 | 95.4 |  |  |  |  |  |  |  |  |
| **Date Getman** |  |  |  |  |  |  | 1434 | 1494 | 68.2 | 1417 | 1524 | 95.4 |  | 99.7 |
| **Boundary End Getman** | 1150 | 1635 | 68.2 | 1150 | 1635 | 95.4 | 1496 | 1529 | 68.2 | 1479 | 1545 | 95.4 | 100 | 99.8 |
| **U(1150,1635)** | 1150 | 1635 | 68.2 | 1150 | 1635 | 95.4 |  |  |  |  |  |  |  |  |
| **Sequence** |  |  |  |  |  |  |  |  |  |  |  |  |  |  |
| **Boundary Start Smith-Pagerie** | 1150 | 1635 | 68.2 | 1150 | 1635 | 95.4 | 1454 | 1498 | 68.2 | 1433 | 1614 | 95.4 | 100 | 99.7 |
| **U(1150,1635)** | 1150 | 1635 | 68.2 | 1150 | 1635 | 95.4 |  |  |  |  |  |  |  |  |
| **Phase Smith-Pagerie** |  |  |  |  |  |  |  |  |  |  |  |  |  |  |
| **R_Combine Feature 54 Pit H1 - assume one event** | 1463 | 1619 | 68.2 | 1451 | 1632 | 95.4 | 1475 | 1512 | 68.2 | 1461 | 1618 | 95.4 | 121.5 | 99.8 |
| **R_Date AA-7405 M** | 1423 | 1615 | 68.2 | 1410 | 1632 | 95.4 |  |  |  |  |  |  |  |  |
| **R_Date AA-6419 M** | 1438 | 1618 | 68.2 | 1425 | 1634 | 95.4 |  |  |  |  |  |  |  |  |
| **R_Date UCIAMS218490 M** | 1515 | 1636 | 68.2 | 1480 | 1644 | 95.4 |  |  |  |  |  |  |  |  |
| **R_Date ISGS-A0528 R H1** | 1423 | 1468 | 68.2 | 1408 | 1617 | 95.4 | 1473 | 1513 | 68.2 | 1455 | 1621 | 95.4 | 108.4 | 99.7 |
| **R_Date UCIAMS190566 B H1** | 1458 | 1614 | 68.2 | 1451 | 1618 | 95.4 | 1473 | 1511 | 68.2 | 1462 | 1616 | 95.4 | 101.5 | 99.7 |
| **R_Date UCIAMS190565 B H1** | 1464 | 1616 | 68.2 | 1452 | 1620 | 95.4 | 1475 | 1511 | 68.2 | 1464 | 1617 | 95.4 | 109.3 | 99.8 |
| **R_Date UCIAMS190563 B H1** | 1470 | 1619 | 68.2 | 1456 | 1631 | 95.5 | 1476 | 1512 | 68.2 | 1464 | 1618 | 95.4 | 119.9 | 99.8 |
| **R_Date UCIAMS218492 M Longhouse 2 Hearth** | 1485 | 1621 | 68.2 | 1470 | 1632 | 95.4 | 1479 | 1513 | 68.2 | 1468 | 1618 | 95.4 | 108.5 | 99.8 |
| **R_Date UCIAMS190564 B Longhouse 4 Hearth** | 1489 | 1630 | 68.3 | 1469 | 1634 | 95.4 | 1478 | 1513 | 68.2 | 1467 | 1619 | 95.4 | 102.1 | 99.8 |
| **Interval Interval Smith-Pagerie** | 0 | 120 | 68.2 | 0 | 120 | 95.4 | 0 | 41 | 68.2 | 0 | 86 | 95.4 | 100 | 99.4 |
| **U(0,120)** | 1.28E-15 | 120 | 68.2 | 1.28E-15 | 120 | 95.4 |  |  |  |  |  |  |  |  |
| **Date Smith-Pagerie** |  |  |  |  |  |  | 1473 | 1514 | 68.2 | 1454 | 1621 | 95.4 |  | 99.8 |
| **Boundary End Smith-Pagerie** | 1150 | 1635 | 68.2 | 1150 | 1635 | 95.4 | 1486 | 1528 | 68.2 | 1475 | 1632 | 95.4 | 100 | 99.8 |
| **U(1150,1635)** | 1150 | 1635 | 68.2 | 1150 | 1635 | 95.4 |  |  |  |  |  |  |  |  |
| **Sequence** |  |  |  |  |  |  |  |  |  |  |  |  |  |  |
| **Boundary Start Otstungo** | 1150 | 1635 | 68.2 | 1150 | 1635 | 95.4 | 1440 | 1478 | 68.2 | 1425 | 1607 | 95.4 | 100 | 99.7 |
| **U(1150,1635)** | 1150 | 1635 | 68.2 | 1150 | 1635 | 95.4 |  |  |  |  |  |  |  |  |
| **Phase Otstungo House 1 Midden and Hearths** |  |  |  |  |  |  |  |  |  |  |  |  |  |  |
| **R_Date UCIAMS190551 Hearth B** | 1474 | 1620 | 68.2 | 1458 | 1633 | 95.4 | 1475 | 1522 | 68.2 | 1456 | 1622 | 95.4 | 107.9 | 99.5 |
| **R_Date UCIAMS190549 Hearth B** | 1496 | 1632 | 68.2 | 1484 | 1638 | 95.4 | 1479 | 1534 | 68.2 | 1470 | 1623 | 95.5 | 91.5 | 99.6 |
| **R_Date UCIAMS190550 Hearth B** | 1523 | 1641 | 68.3 | 1516 | 1644 | 95.4 | 1498 | 1554 | 68.2 | 1488 | 1627 | 95.4 | 91 | 99.6 |
| **R_Date UCIAMS190548 Hearth B** | 1523 | 1641 | 68.3 | 1516 | 1644 | 95.4 | 1498 | 1554 | 68.2 | 1489 | 1626 | 95.4 | 90.9 | 99.7 |
| **R_Date AA-7400 M** | 1433 | 1616 | 68.2 | 1417 | 1634 | 95.4 | 1458 | 1518 | 68.2 | 1443 | 1621 | 95.3 | 100.3 | 99.3 |
| **R_Date AA-7402 M** | 1435 | 1617 | 68.2 | 1421 | 1634 | 95.4 | 1458 | 1519 | 68.2 | 1444 | 1621 | 95.3 | 103.5 | 99.4 |
| **R_Date AA-6423 M** | 1438 | 1620 | 68.1 | 1427 | 1636 | 95.4 | 1459 | 1522 | 68.2 | 1446 | 1620 | 95.4 | 108.8 | 99.4 |
| **R_Date UCIAMS218483 M** | 1449 | 1472 | 68.2 | 1444 | 1609 | 95.4 | 1457 | 1608 | 68.2 | 1448 | 1614 | 95.4 | 70.6 | 98.5 |
| **R_Date AA-7398 M** | 1447 | 1625 | 68.1 | 1440 | 1638 | 95.4 | 1461 | 1526 | 68.2 | 1448 | 1621 | 95.4 | 110.1 | 99.4 |
| **R_Date UCIAMS218489 M** | 1453 | 1615 | 68.2 | 1447 | 1620 | 95.4 | 1462 | 1512 | 68.2 | 1451 | 1617 | 95.4 | 102.1 | 99.2 |
| **R_Date AA-7401 M** | 1455 | 1628 | 68.2 | 1447 | 1638 | 95.4 | 1462 | 1528 | 68.2 | 1450 | 1621 | 95.4 | 108.3 | 99.5 |
| **R_Date AA-7399 M** | 1476 | 1633 | 68.2 | 1449 | 1646 | 95.4 | 1466 | 1535 | 68.2 | 1452 | 1622 | 95.4 | 105.2 | 99.5 |
| **R_Date UCIAMS218487 M** | 1495 | 1630 | 68.2 | 1478 | 1634 | 95.4 | 1485 | 1527 | 68.2 | 1471 | 1624 | 95.4 | 95.1 | 99.6 |
| **Interval Interval Otstungo** | 0 | 120 | 68.2 | 0 | 120 | 95.4 | 70 | 120 | 68.2 | 23 | 120 | 95.4 | 100 | 99.6 |
| **U(0,120)** | 1.28E-15 | 120 | 68.2 | 1.28E-15 | 120 | 95.4 |  |  |  |  |  |  |  |  |
| **Date Otstungo** |  |  |  |  |  |  | 1464 | 1538 | 68.2 | 1448 | 1622 | 95.4 |  | 99.4 |
| **Boundary End Otstungo** | 1150 | 1635 | 68.2 | 1150 | 1635 | 95.4 | 1523 | 1572 | 68.2 | 1504 | 1637 | 95.4 | 100 | 99.2 |
| **U(1150,1635)** | 1150 | 1635 | 68.2 | 1150 | 1635 | 95.4 |  |  |  |  |  |  |  |  |
| **Sequence** |  |  |  |  |  |  |  |  |  |  |  |  |  |  |
| **Boundary Start Klock** | 1150 | 1635 | 68.2 | 1150 | 1635 | 95.4 | 1470 | 1587 | 68.2 | 1456 | 1616 | 95.4 | 100 | 98.6 |
| **U(1150,1635)** | 1150 | 1635 | 68.2 | 1150 | 1635 | 95.4 |  |  |  |  |  |  |  |  |
| **Phase Klock** |  |  |  |  |  |  |  |  |  |  |  |  |  |  |
| **R_Combine Feature 84 Pit H1 - assume one event** | 1477 | 1618 | 68.2 | 1461 | 1630 | 95.5 | 1489 | 1615 | 68.3 | 1477 | 1622 | 95.4 | 105.7 | 98.4 |
| **R_Date UCIAMS218474 M** | 1470 | 1616 | 68.2 | 1454 | 1625 | 95.4 |  |  |  |  |  |  |  |  |
| **R_Date ISGS-A0326 M** | 1516 | 1642 | 68.2 | 1472 | 1649 | 95.4 |  |  |  |  |  |  |  |  |
| **R_Date ISGS-A0523 R Pit** | 1415 | 1446 | 68.2 | 1327 | 1474 | 95.4 | 1486 | 1603 | 68.2 | 1475 | 1626 | 95.4 | 100.7 | 98.4 |
| **R_Date UCIAMS218476 M Pit** | 1470 | 1616 | 68.2 | 1454 | 1625 | 95.4 | 1490 | 1610 | 68.2 | 1476 | 1620 | 95.4 | 97 | 98.3 |
| **R_Date UCIAMS190559 B Pit** | 1475 | 1617 | 68.2 | 1459 | 1630 | 95.5 | 1489 | 1614 | 68.2 | 1477 | 1622 | 95.4 | 106.1 | 98.3 |
| **R_Date UCIAMS190561 B Pit** | 1499 | 1632 | 68.2 | 1485 | 1635 | 95.4 | 1491 | 1601 | 68.2 | 1485 | 1629 | 95.4 | 99.1 | 98.4 |
| **R_Date UCIAMS218475 M Pit** | 1496 | 1632 | 68.2 | 1484 | 1638 | 95.4 | 1490 | 1601 | 68.2 | 1483 | 1628 | 95.4 | 103.5 | 98.4 |
| **R_Date UCIAMS218473 B Pit** | 1519 | 1634 | 68.2 | 1493 | 1640 | 95.4 | 1492 | 1600 | 68.2 | 1488 | 1628 | 95.4 | 80.6 | 98.3 |
| **R_Date UCIAMS190562 B Hearth H1** | 1474 | 1620 | 68.2 | 1458 | 1633 | 95.4 | 1488 | 1616 | 68.1 | 1478 | 1626 | 95.4 | 114.4 | 98.5 |
| **R_Date UCIAMS190560 M Hearth H1** | 1519 | 1634 | 68.2 | 1493 | 1640 | 95.4 | 1492 | 1600 | 68.2 | 1488 | 1628 | 95.4 | 80.6 | 98.3 |
| **R_Date AA-6418 M Hearth H7** | 1495 | 1644 | 68.1 | 1448 | 1795 | 95.4 | 1488 | 1603 | 68.2 | 1481 | 1627 | 95.4 | 108.1 | 98.4 |
| **Interval Interval Klock** | 0 | 120 | 68.2 | 0 | 120 | 95.4 | 0 | 50 | 68.2 | 0 | 120 | 95.4 | 100 | 99.5 |
| **U(0,120)** | 1.28E-15 | 120 | 68.2 | 1.28E-15 | 120 | 95.4 |  |  |  |  |  |  |  |  |
| **Date Klock** |  |  |  |  |  |  | 1488 | 1617 | 68.2 | 1478 | 1627 | 95.4 |  | 98.3 |
| **Boundary End Klock** | 1150 | 1635 | 68.2 | 1150 | 1635 | 95.4 | 1502 | 1632 | 68.2 | 1494 | 1637 | 95.4 | 100 | 98 |
| **U(1150,1635)** | 1150 | 1635 | 68.2 | 1150 | 1635 | 95.4 |  |  |  |  |  |  |  |  |
| **Sequence** |  |  |  |  |  |  |  |  |  |  |  |  |  |  |
| **Boundary Start Cayadutta Midden** | 1150 | 1635 | 68.2 | 1150 | 1635 | 95.4 | 1455 | 1594 | 68.2 | 1440 | 1615 | 95.4 | 100 | 99.5 |
| **U(1150,1635)** | 1150 | 1635 | 68.2 | 1150 | 1635 | 95.4 |  |  |  |  |  |  |  |  |
| **Phase Cayadutta Midden** |  |  |  |  |  |  |  |  |  |  |  |  |  |  |
| **R_Date AA-7690 M** | 1431 | 1618 | 68.2 | 1416 | 1635 | 95.4 | 1480 | 1618 | 68.2 | 1460 | 1628 | 95.3 | 86.3 | 99.9 |
| **R_Date AA-7407 M** | 1454 | 1628 | 68.2 | 1446 | 1639 | 95.4 | 1485 | 1619 | 68.2 | 1470 | 1627 | 95.4 | 108 | 99.9 |
| **R_Date AA-6421 M** | 1513 | 1650 | 68.2 | 1462 | 1795 | 95.4 | 1489 | 1621 | 68.2 | 1482 | 1627 | 95.4 | 99.1 | 99.8 |
| **R_Date UCIAMS-205965 M** | 1474 | 1620 | 68.2 | 1458 | 1633 | 95.4 | 1486 | 1618 | 68.2 | 1470 | 1627 | 95.4 | 108.9 | 99.9 |
| **R_Date UCIAMS-205966 M** | 1495 | 1630 | 68.2 | 1478 | 1634 | 95.4 | 1490 | 1620 | 68.2 | 1479 | 1629 | 95.4 | 103.1 | 99.9 |
| **R_Date UCIAMS-205967 M** | 1522 | 1640 | 68.2 | 1499 | 1643 | 95.4 | 1494 | 1624 | 68.2 | 1489 | 1629 | 95.4 | 77 | 99.8 |
| **R_Date UCIAMS-205968 M** | 1458 | 1614 | 68.2 | 1451 | 1618 | 95.4 | 1481 | 1616 | 68.2 | 1462 | 1619 | 95.4 | 84 | 99.9 |
| **Interval Interval Cayadutta Midden** | 0 | 120 | 68.2 | 0 | 120 | 95.4 | 8 | 120 | 68.2 | 1 | 120 | 95.4 | 100 | 99.6 |
| **U(0,120)** | 1.28E-15 | 120 | 68.2 | 1.28E-15 | 120 | 95.4 |  |  |  |  |  |  |  |  |
| **Date Cayadutta Midden** |  |  |  |  |  |  | 1485 | 1620 | 68.2 | 1473 | 1627 | 95.4 |  | 99.9 |
| **Boundary End Cayadutta Midden** | 1150 | 1635 | 68.2 | 1150 | 1635 | 95.4 | 1511 | 1637 | 68.2 | 1502 | 1637 | 95.4 | 100 | 99.5 |
| **U(1150,1635)** | 1150 | 1635 | 68.2 | 1150 | 1635 | 95.4 |  |  |  |  |  |  |  |  |
| **Sequence** |  |  |  |  |  |  |  |  |  |  |  |  |  |  |
| **Boundary Start Garoga** | 1150 | 1635 | 68.2 | 1150 | 1635 | 95.4 | 1480 | 1563 | 68.2 | 1457 | 1586 | 95.4 | 100 | 99.8 |
| **U(1150,1635)** | 1150 | 1635 | 68.2 | 1150 | 1635 | 95.4 |  |  |  |  |  |  |  |  |
| **Phase Garoga** |  |  |  |  |  |  |  |  |  |  |  |  |  |  |
| **R_Date Y-1381 charred wood** | 1287 | 1406 | 68.2 | 1193 | 1463 | 95.4 | 1512 | 1581 | 68.2 | 1479 | 1627 | 95.4 | 93.8 | 99.9 |
| **R_Combine Feature 2 Pit H9** | 1493 | 1630 | 68.3 | 1476 | 1633 | 95.4 | 1506 | 1591 | 68.2 | 1488 | 1628 | 95.4 | 97.1 | 99.9 |
| **R_Date AA-7403 M F2 Pit H9** | 1432 | 1619 | 68.2 | 1418 | 1636 | 95.4 |  |  |  |  |  |  |  |  |
| **R_Date UCIAMS218478 M F2 Pit H9** | 1491 | 1628 | 68.2 | 1472 | 1633 | 95.4 |  |  |  |  |  |  |  |  |
| **R_Date UCIAMS190537 M F2 Pit H9** | 1496 | 1632 | 68.2 | 1484 | 1638 | 95.4 |  |  |  |  |  |  |  |  |
| **R_Date AA-6417 M F2 Pit H9** | 1513 | 1650 | 68.2 | 1462 | 1795 | 95.4 |  |  |  |  |  |  |  |  |
| **R_Date ISGS-A0522 R H5 Pit** | 1431 | 1608 | 68.2 | 1417 | 1627 | 95.3 | 1512 | 1580 | 68.2 | 1483 | 1627 | 95.4 | 103.9 | 99.8 |
| **R_Date UCIAMS190540 M H4 Pit** | 1489 | 1630 | 68.3 | 1469 | 1634 | 95.4 | 1504 | 1592 | 68.2 | 1487 | 1627 | 95.4 | 97.9 | 99.9 |
| **R_Date UCIAMS190539 M H2 Pit** | 1521 | 1636 | 68.2 | 1491 | 1643 | 95.4 | 1517 | 1580 | 68.2 | 1495 | 1624 | 95.4 | 106.9 | 99.9 |
| **R_Date UCIAMS218479 M H12 Pit** | 1522 | 1640 | 68.2 | 1499 | 1643 | 95.4 | 1521 | 1574 | 68.2 | 1498 | 1626 | 95.4 | 102.8 | 99.9 |
| **R_Combine F184 Pit btw H1&Stockade** | 1522 | 1636 | 68.1 | 1494 | 1643 | 95.4 | 1519 | 1576 | 68.2 | 1497 | 1623 | 95.4 | 105.3 | 99.9 |
| **R_Date UCIAMS190538 M F184 Pit** | 1523 | 1643 | 68.2 | 1499 | 1647 | 95.4 |  |  |  |  |  |  |  |  |
| **R_Date UCIAMS218477 M F184 Pit** | 1500 | 1634 | 68.3 | 1487 | 1640 | 95.4 |  |  |  |  |  |  |  |  |
| **Interval Interval Garoga** | 0 | 120 | 68.2 | 0 | 120 | 95.4 | 0 | 89 | 68.2 | 0 | 115 | 95.4 | 100 | 99.7 |
| **U(0,120)** | 1.28E-15 | 120 | 68.2 | 1.28E-15 | 120 | 95.4 |  |  |  |  |  |  |  |  |
| **Date Garoga** |  |  |  |  |  |  | 1515 | 1584 | 68.2 | 1486 | 1627 | 95.4 |  | 99.9 |
| **Boundary End Garoga** | 1150 | 1635 | 68.2 | 1150 | 1635 | 95.4 | 1526 | 1611 | 68.2 | 1523 | 1632 | 95.4 | 100 | 99.9 |
| **U(1150,1635)** | 1150 | 1635 | 68.2 | 1150 | 1635 | 95.4 |  |  |  |  |  |  |  |  |
| **Sequence** |  |  |  |  |  |  |  |  |  |  |  |  |  |  |
| **Boundary Start Wormuth** | 1150 | 1635 | 68.2 | 1150 | 1635 | 95.4 | 1463 | 1605 | 68.2 | 1445 | 1615 | 95.4 | 100 | 99.7 |
| **U(1150,1635)** | 1150 | 1635 | 68.2 | 1150 | 1635 | 95.4 |  |  |  |  |  |  |  |  |
| **Phase Wormuth** |  |  |  |  |  |  |  |  |  |  |  |  |  |  |
| **R_Date AA-6416 M** | 1445 | 1620 | 68.2 | 1438 | 1636 | 95.4 | 1488 | 1618 | 68.2 | 1468 | 1627 | 95.4 | 100.2 | 99.8 |
| **R_Date UCIAMS192700 M Pit** | 1475 | 1617 | 68.2 | 1459 | 1630 | 95.5 | 1489 | 1617 | 68.2 | 1472 | 1622 | 95.4 | 100.2 | 99.8 |
| **R_Date AA-6065 M** | 1491 | 1632 | 68.2 | 1473 | 1636 | 95.4 | 1492 | 1619 | 68.2 | 1478 | 1627 | 95.4 | 105.7 | 99.8 |
| **R_Date DIC-1176 Lowest Level Midden Charcoal** | 1315 | 1422 | 68.2 | 1298 | 1437 | 95.4 | 1486 | 1619 | 68.2 | 1469 | 1626 | 95.4 | 99.1 | 99.7 |
| **R_Date DIC-1177 Higher Up Midden Charcoal** | 1523 | ... | 68.3 | 1485 | ... | 95.4 | 1501 | 1619 | 68.2 | 1489 | 1625 | 95.4 | 60 | 99.8 |
| **R_Date DIC-1178 Fill of Burial #2** | 1434 | 1618 | 68.2 | 1419 | 1635 | 95.4 | 1490 | 1620 | 68.2 | 1478 | 1626 | 95.4 | 107.1 | 99.8 |
| **Interval Interval Wormuth** | 0 | 120 | 68.2 | 0 | 120 | 95.4 | 0 | 120 | 68.2 | 0 | 120 | 95.4 | 100 | 99.7 |
| **U(0,120)** | 1.28E-15 | 120 | 68.2 | 1.28E-15 | 120 | 95.4 |  |  |  |  |  |  |  |  |
| **Date Wormuth** |  |  |  |  |  |  | 1490 | 1620 | 68.2 | 1475 | 1627 | 95.4 |  | 99.8 |
| **Boundary End Wormuth** | 1150 | 1635 | 68.2 | 1150 | 1635 | 95.4 | 1512 | 1635 | 68.2 | 1505 | 1637 | 95.4 | 100 | 99.8 |
| **U(1150,1635)** | 1150 | 1635 | 68.2 | 1150 | 1635 | 95.4 |  |  |  |  |  |  |  |  |
| **Order Order** |  |  |  |  |  |  |  |  |  |  |  |  |  |  |


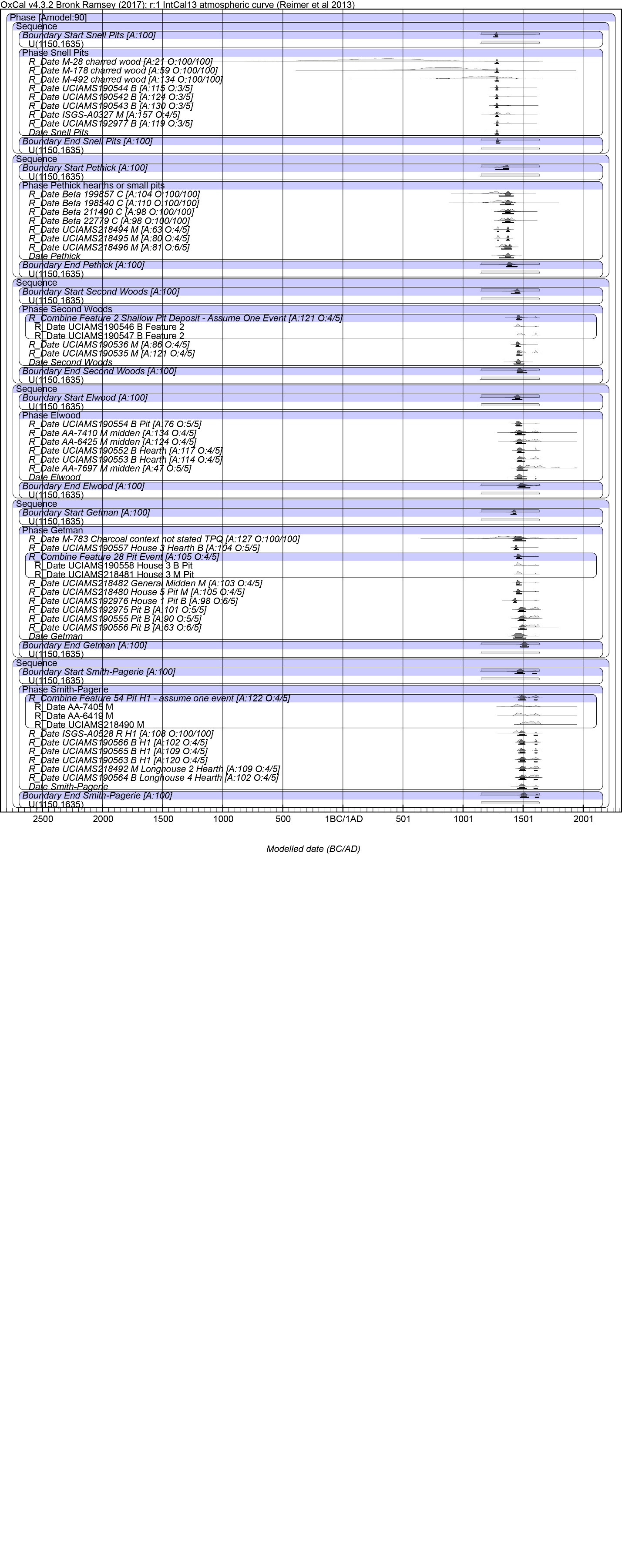


Model 2 Part 1


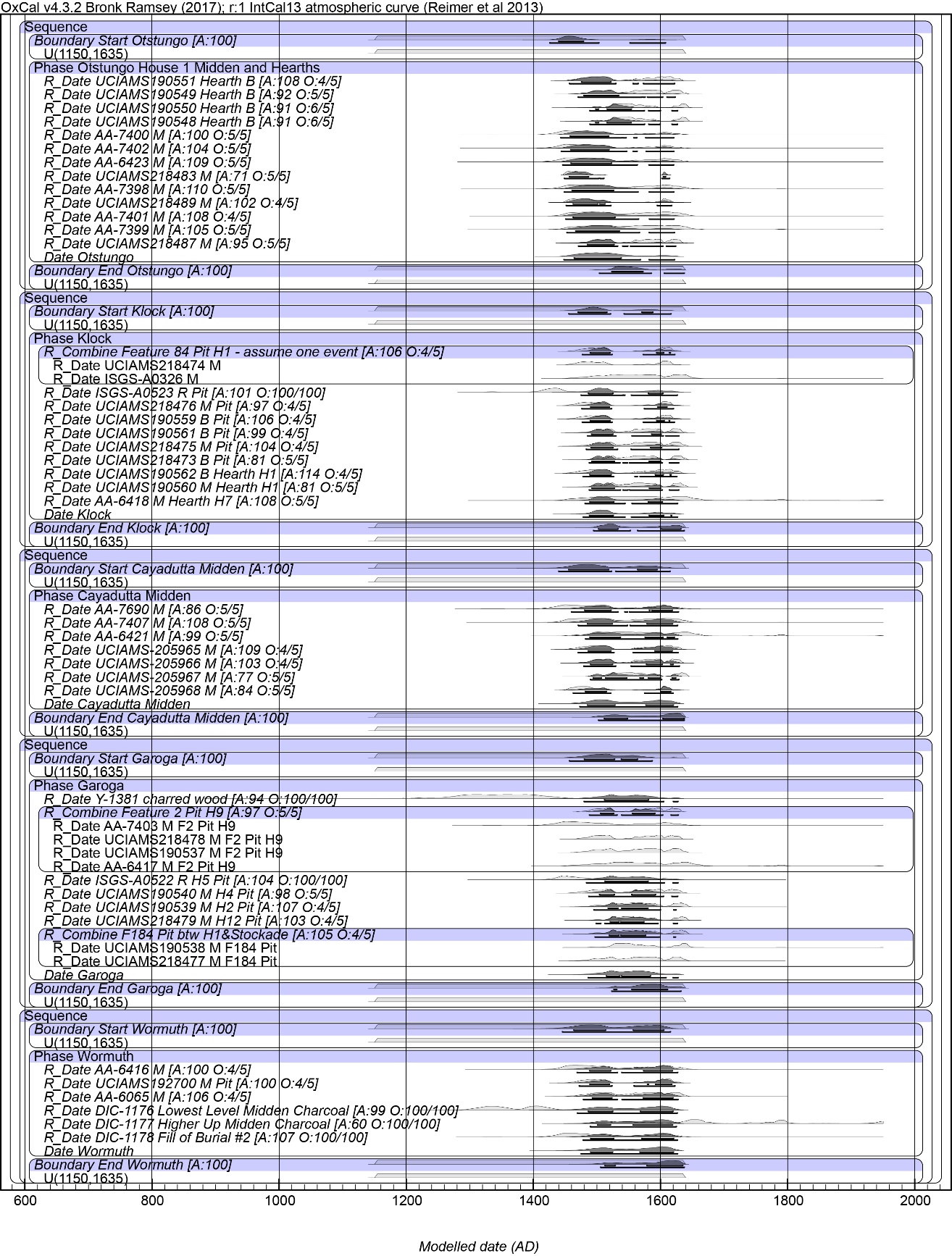


Model 2 Part 2

Figures (Parts 1 and 2) show Model 2 with the uniform probability 0-120 years constraint on an Interval query for each site Phase duration (for details see Table above). The figures indicate the structure of the model. The light grey histograms are the non-modelled probability ranges. The black histograms are the modelled probability ranges. The lines under each black histogram indicate the 68.2% and 95.4% hpd ranges.
