# Supplementary material for: Radiocarbon, Bayesian chronological modeling and early European metal circulation in the sixteenth-century AD Mohawk River Valley, USA
Source: PLoS One. 2019 Dec 16;14(12):e0226334. doi: 10.1371/journal.pone.0226334 (PMC6913979; doi:10.1371/journal.pone.0226334)
Supplement: S1 Appendix — (PDF) [file pone.0226334.s010.pdf]

S1 Appendix. Comparison test cases for the use of an Interval constraint on hypothetical known-age site Phase data.

In the main text paper we employ Interval constraints to avoid (much too) over-long possible site Phase Date estimates for data falling on the reversal/plateau in the radiocarbon calibration curve centered AD 1500–1600. This also introduced stability into the placement of model runs. Here, using some hypothetical data/cases, we briefly explore the effectiveness and limitations of such a strategy. This is not a comprehensive analysis. Rather, it is an exploratory discussion to highlight some relevant aspects for such analyses and to support its application, subject to caveats. As in the main text, the analyses employ OxCal [23] and IntCal13 [22] (curve resolution 5 years).

We consider a hypothetical set of 5 sites (A, B, C, D, E) which date within the periods 1500–1540, 1520–1560, 1540–1580, 1550–1590 and 1560–1600.

To begin, let us assume we have perfect radiocarbon measurements on annual growth samples from specific years from these sites under a range of scenarios. Thus, for a given calendar year, we assume a value that corresponds to the age for that year of the IntCal13 calibration dataset [22]. We consider all the radiocarbon dates to have measurement precision at about best current measurement level: ±15 ^{14}C years (whether very precise single measurements, or notional weighted averages of two or more original dates).

Model A considers two dates for each of the five sites for the years Site A 1510, 1520, Site B 1530, 1540, Site C 1550, 1560, Site D 1560, 1570 and Site E 1570, 1580. Model A1 applies an Interval constraint to each site Phase of 0–80 years and Model A2 applies a 0–120 years Interval constraint and Model A3 has no constraint. The Order probabilities for a Date estimate for each Site Phase are shown in Figure A. We observe that in all but one (marginal) case the Order probabilities are slightly improved, and correctly improved, or the same, comparing the Model A1 and Model A2 results versus the Model A3 results. Thus, of the 10 pairs of comparison, 8 are improved, one is effectively the same, and just one (Site B v. A) is made worse and incorrect (changing from 0.50 to 0.51 when a result <0.5 would be correct) in Model A1 and 5 of 10 values are correctly improved in Model A2. Thus, overall, consideration of an Interval constraint is useful and does little/no harm.

Model B considers three dates for each of the sites for the years Site A 1510, 1520, 1530, Site B 1530, 1540, 1550, Site C 1550, 1560, 1570, Site D 1560, 1570, 1580, and Site E 1570, 1580, 1590. Model B1 applies an Interval Constraint to each site Phase of 0–80 years and Model B2 applies a 0–120 years Interval constraint and Model B3 has no constraint. The Order probabilities for a Date estimate for each site Phase are shown in Figure A. We observe that whether with or without the Interval constraint one or two of the 10 pairs of comparisons yield the incorrect order (Sites B v. A and Site C v. A in two cases and Site B v. A in one case). Thus, here there is a challenge, but the use or non-use of an Interval constraint makes no substantive or helpful difference. Otherwise, in Model B1, 5 of the 10 pairs of comparisons yield clearer correct Order probabilities (versus Model B3), and in Model B2 8 of the 10 pairs yield clearer and correct Order probabilities (versus Model B3). In the other three, or one, cases respectively there is more or less no change compared to the no constraint Model B3. Again, overall, the

consideration of a very conservative and then a conservative Interval constraint is helpful and does no or little harm compared to a model with no Interval constraint.

Model A1: Order Test Interval Constraint 0-80 years

t_1	Probability $t_1 < t_2$: Date Site A Int	Date Site B Int	Date Site C Int	Date Site D Int	Date Site E Int
Date Site A Int	0.00	0.49	0.52	**0.55**	**0.56**
Date Site B Int	0.51	0.00	**0.59**	**0.67**	**0.67**
Date Site C Int	0.48	0.41	0.00	**0.59**	**0.60**
Date Site D Int	0.45	0.33	0.41	0.00	**0.52**
Date Site E Int	0.44	0.33	0.40	0.48	0.00

Model B1: Order Test Interval Constraint 0-80 years

t_1	Probability $t_1 < t_2$: Date Site A Int	Date Site B Int	Date Site C Int	Date Site D Int	Date Site E Int
Date Site A Int	0.00	0.40	0.47	0.52	0.52
Date Site B Int	0.60	0.00	**0.61**	**0.66**	**0.66**
Date Site C Int	0.53	0.39	0.00	**0.57**	**0.58**
Date Site D Int	0.48	0.34	0.43	0.00	0.51
Date Site E Int	0.48	0.34	0.42	0.49	0.00

Model C1: Order Test Interval Constraint 0-80 years

t_1	Probability $t_1 < t_2$: Date Site A Int	Date Site B Int	Date Site C Int	Date Site D Int	Date Site E Int
Date Site A Int	0.00	0.42	0.47	0.52	**0.58**
Date Site B Int	0.58	0.00	**0.56**	**0.64**	**0.70**
Date Site C Int	0.53	0.44	0.00	**0.58**	**0.65**
Date Site D Int	0.48	0.36	0.42	0.00	**0.58**
Date Site E Int	0.42	0.30	0.35	0.42	0.00

Model A2: Order Test Interval Constraint 0-120 years

t_1	Probability $t_1 < t_2$: Date Site A Int	Date Site B Int	Date Site C Int	Date Site D Int	Date Site E Int
Date Site A Int	0.00	0.50	0.52	0.54	0.54
Date Site B Int	0.50	0.00	**0.55**	**0.58**	**0.58**
Date Site C Int	0.48	0.45	0.00	0.53	**0.54**
Date Site D Int	0.46	0.42	0.47	0.00	**0.51**
Date Site E Int	0.46	0.42	0.46	0.49	0.00

Model B2: Order Test Interval Constraint 0-120 years

t_1	Probability $t_1 < t_2$: Date Site A Int	Date Site B Int	Date Site C Int	Date Site D Int	Date Site E Int
Date Site A Int	0.00	0.42	**0.51**	**0.58**	**0.59**
Date Site B Int	0.58	0.00	**0.65**	**0.73**	**0.73**
Date Site C Int	0.49	0.35	0.00	**0.61**	**0.62**
Date Site D Int	0.42	0.27	0.39	0.00	0.51
Date Site E Int	0.41	0.27	0.38	0.49	0.00

Model C2: Order Test Interval Constraint 0-120 years

t_1	Probability $t_1 < t_2$: Date Site A Int	Date Site B Int	Date Site C Int	Date Site D Int	Date Site E Int
Date Site A Int	0.00	0.45	0.48	0.53	**0.58**
Date Site B Int	0.55	0.00	**0.55**	**0.62**	**0.66**
Date Site C Int	0.52	0.45	0.00	**0.57**	**0.63**
Date Site D Int	0.47	0.38	0.43	0.00	**0.56**
Date Site E Int	0.42	0.34	0.37	0.44	0.00

Model A3: Order Test No Interval Constraint

t_1	Probability $t_1 < t_2$: Date Site A no Int	Date Site B no Int	Date Site C no Int	Date Site D no Int	Date Site E no Int
Date Site A no Int	0.00	0.50	0.52	0.54	0.54
Date Site B no Int	0.50	0.00	0.54	0.56	0.56
Date Site C no Int	0.48	0.46	0.00	0.53	0.53
Date Site D no Int	0.46	0.44	0.47	0.00	0.50
Date Site E no Int	0.46	0.44	0.47	0.50	0.00

Model B3: Order Test No Interval Constraint

t_1	Probability $t_1 < t_2$: Date Site A no Int	Date Site B no Int	Date Site C no Int	Date Site D no Int	Date Site E no Int
Date Site A no Int	0.00	0.46	0.50	0.52	0.53
Date Site B no Int	0.54	0.00	0.56	0.58	0.58
Date Site C no Int	0.50	0.44	0.00	0.53	0.53
Date Site D no Int	0.48	0.42	0.47	0.00	0.51
Date Site E no Int	0.47	0.42	0.47	0.49	0.00

Model C3: Order Test No Interval Constraint

t_1	Probability $t_1 < t_2$: Date Site A no Int	Date Site B no Int	Date Site C no Int	Date Site D no Int	Date Site E no Int
Date Site A no Int	0.00	0.47	0.50	0.54	0.57
Date Site B no Int	0.53	0.00	0.54	0.59	0.63
Date Site C no Int	0.50	0.46	0.00	0.56	0.60
Date Site D no Int	0.46	0.41	0.44	0.00	0.55
Date Site E no Int	0.43	0.37	0.40	0.45	0.00

Figure A. Order probabilities for Models A1 to A3, B1 to B3 and C1 to C3. Bold entries show better results for Models A1, B1 and C1 or A2, B2 or C2 versus Models A3, B3 and C3 respectively. The underlined result indicates a worse result for Models A1, A2, B1, B2, C1 or C2 versus, respectively, Models A3, B3 and C3. Data shown from typical model runs, each model run varies slightly.

Model C considers five dates for each of the sites for the years Site A 1500, 1510, 1520, 1530, 1540, Site B 1520, 1530, 1540, 1550, 1560, Site C 1540, 1550, 1560, 1570, 1580, Site D 1550, 1560, 1570, 1580, 1590, and Site E 1560, 1570, 1580, 1590, 1600. Model C1 applies an Interval Constraint to each site Phase of 0–80 years and Model C2 applies a 0–120 years Interval constraint and Model C3 has no constraint. The Order probabilities for a Date estimate for each site Phase are shown in Figure A. As for Model B, we observe that whether with or without the Interval constraint two of the 10 pairs of comparisons yield the incorrect order (Site B v. A and Site C v. A). For one other pair of comparisons (Site A v. Site D) the introduction of the Interval constraints slightly reduces the correct probability but do not change the indication that Site A is, correctly, older than Site D. In the other 7 of the 10 cases in both Model C1 and C2 the Interval constraint enhances the probability of the correct Order. Again, overall, use of the Interval constraints assists to clarify correctly the Order probabilities and does no actual harm. The OxCal runfiles for Models C1, C2 and C3 are listed below (to derive Models B and A reduce the dates in each site Phase to those described in the text above).

If we consider the findings from Models A to C, the introduction of the Interval constraint proves useful in the majority of cases and rarely (one marginal case only) creates a new incorrect Order outcome (versus enhancing an already existing incorrect Order outcome). In these models we observe a consistent problem that a site dating in the range ca. 1500–1540 does not necessarily resolve as older than sites dating in the range of 1520–1560 and 1540–1580. Here application of an Interval constraint can in fact exacerbate this issue. Care thus needs to be applied in cases where a possible early 16th century range is possible. We note this issue in the main text with regard to the Order of the sites of Cayadutta and Garoga (and hence consider an alternative).

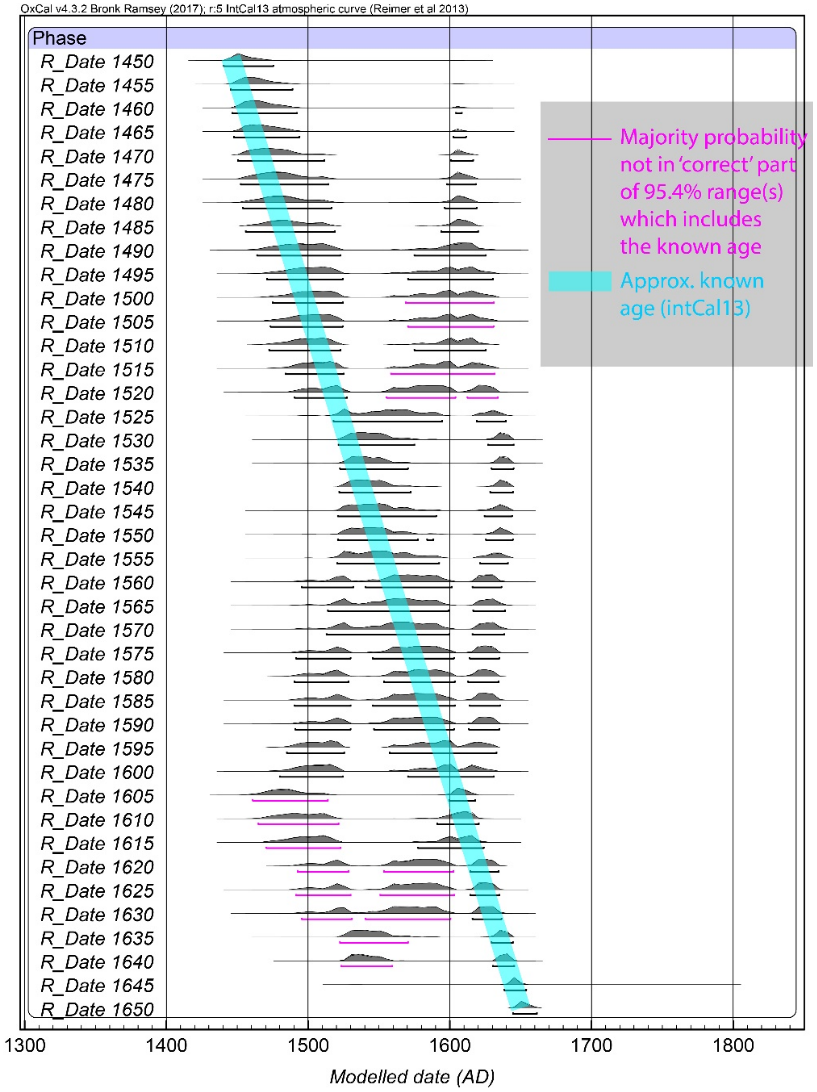

Figure B. Calibrated probability distributions for an OxCal Phase containing the IntCal13 calibration curve for the calendar years 1450 to 1650 with the 95.4% ranges for each 5 year interval indicated. The cyan line indicates the approximate IntCal13 curve trajectory. The magenta sub-ranges are those where the majority of the probability (within the most likely 95.4% range) is distinct from the range or sub-range including the known age (the IntCal calendar age). The late 15th through earlier 17th century ambiguity period caused by the reversal/plateau in the calibration curve is evident.

There are inherent limitations to an Order analysis given the history of past atmospheric radiocarbon levels – i.e. the radiocarbon calibration curve – which we should note. If we consider the IntCal13 radiocarbon curve and the values every 5 calendar years AD 1450 to 1650 [22], and calibrate these independently in a Phase [23], then we can identify some periods where the most likely sub-ranges at 95.4% probability do not include the correct known age: Figure B. We may note these as 1500, 1505, 1515, 1520 (consistent with the observation above about the early 16th century being a particularly difficult period for an Order analysis) and 1605 to 1640. If we conduct an Order analysis for this set of IntCal values 1450 to 1650 within a Phase treating each value as independent, then it is the regions 1500-1510, 1520-1525, 1560, 1590-1615 and 1635-1640 that stand out as including incorrect/ambiguous Order probabilities: see Figure C. We may assume that the periods indicated in Figures B and C will be the main challenges for analysis.

Probability $t_1 < t_2$

t_1 \ t_2	1450	1455	1460	1465	1470	1475	1480	1485	1490	1495	1500	1505	1510	1515	1520	1525	1530	1535	1540	1545	1550
**1450**	0.00	0.76	0.82	0.86	0.92	0.95	0.96	0.98	0.99	1.00	1.00	1.00	1.00	1.00	1.00	1.00	1.00	1.00	1.00	1.00	1.00
**1455**	0.24	0.00	0.59	0.66	0.78	0.86	0.89	0.92	0.96	0.98	0.98	0.98	0.98	0.98	0.99	0.99	0.99	0.99	0.99	0.99	0.99
**1460**	0.18	0.41	0.00	0.57	0.71	0.80	0.83	0.87	0.92	0.95	0.95	0.95	0.95	0.96	0.97	0.97	0.97	0.97	0.97	0.97	0.97
**1465**	0.14	0.34	0.43	0.00	0.65	0.74	0.78	0.83	0.89	0.92	0.93	0.93	0.92	0.94	0.94	0.95	0.95	0.95	0.95	0.95	0.95
**1470**	0.08	0.22	0.29	0.35	0.00	0.60	0.65	0.70	0.78	0.82	0.83	0.83	0.82	0.85	0.86	0.87	0.87	0.88	0.87	0.87	0.87
**1475**	0.05	0.14	0.20	0.26	0.40	0.00	0.55	0.60	0.69	0.74	0.75	0.75	0.74	0.77	0.79	0.80	0.81	0.82	0.81	0.81	0.81
**1480**	0.04	0.11	0.17	0.22	0.35	0.45	0.00	0.56	0.65	0.70	0.71	0.71	0.70	0.73	0.76	0.78	0.79	0.79	0.78	0.78	0.78
**1485**	0.02	0.08	0.13	0.17	0.30	0.40	0.44	0.00	0.60	0.65	0.67	0.67	0.65	0.69	0.73	0.74	0.75	0.76	0.75	0.74	0.75
**1490**	0.01	0.04	0.08	0.11	0.22	0.31	0.35	0.40	0.00	0.55	0.57	0.57	0.55	0.60	0.64	0.66	0.67	0.68	0.67	0.66	0.66
**1495**	0.00	0.02	0.05	0.08	0.18	0.26	0.30	0.35	0.45	0.00	0.52	0.51	0.49	0.55	0.60	0.61	0.62	0.63	0.62	0.61	0.61
**1500**	0.00	0.02	0.05	0.07	0.17	0.25	0.29	0.33	0.43	0.48	0.00	0.50	0.47	0.53	0.59	0.60	0.60	0.61	0.60	0.59	0.59
**1505**	0.00	0.02	0.05	0.07	0.17	0.25	0.29	0.33	0.43	0.49	0.50	0.00	0.47	0.54	0.59	0.60	0.61	0.62	0.61	0.60	0.60
**1510**	0.00	0.02	0.05	0.08	0.18	0.26	0.30	0.35	0.45	0.51	0.53	0.53	0.00	0.57	0.61	0.64	0.65	0.66	0.65	0.64	0.64
**1515**	0.00	0.02	0.04	0.06	0.15	0.23	0.27	0.31	0.40	0.45	0.47	0.46	0.44	0.00	0.55	0.56	0.56	0.57	0.56	0.55	0.55
**1520**	0.00	0.01	0.03	0.06	0.14	0.21	0.24	0.27	0.36	0.40	0.41	0.41	0.39	0.45	0.00	0.48	0.47	0.48	0.47	0.46	0.46
**1525**	0.00	0.01	0.03	0.05	0.13	0.20	0.22	0.26	0.34	0.39	0.40	0.40	0.36	0.44	0.52	0.00	0.43	0.43	0.43	0.44	0.43
**1530**	0.00	0.01	0.03	0.05	0.13	0.19	0.21	0.25	0.33	0.38	0.40	0.39	0.35	0.44	0.53	0.57	0.00	0.50	0.50	0.52	0.50
**1535**	0.00	0.01	0.03	0.05	0.12	0.18	0.21	0.24	0.32	0.37	0.39	0.38	0.34	0.43	0.52	0.57	0.50	0.00	0.50	0.52	0.51
**1540**	0.00	0.01	0.03	0.05	0.13	0.19	0.22	0.25	0.33	0.38	0.40	0.39	0.35	0.44	0.53	0.57	0.50	0.50	0.00	0.52	0.51
**1545**	0.00	0.01	0.03	0.05	0.13	0.19	0.22	0.26	0.34	0.39	0.41	0.40	0.36	0.45	0.54	0.56	0.48	0.48	0.48	0.00	0.48
**1550**	0.00	0.01	0.03	0.05	0.13	0.19	0.22	0.25	0.34	0.39	0.41	0.40	0.36	0.45	0.54	0.57	0.50	0.49	0.49	0.52	0.00
**1555**	0.00	0.01	0.03	0.05	0.13	0.20	0.22	0.26	0.34	0.39	0.41	0.40	0.37	0.45	0.53	0.53	0.46	0.46	0.45	0.47	0.46
**1560**	0.00	0.01	0.03	0.05	0.13	0.19	0.22	0.26	0.33	0.38	0.39	0.39	0.36	0.43	0.48	0.45	0.41	0.42	0.41	0.41	0.40
**1565**	0.00	0.01	0.03	0.05	0.13	0.20	0.22	0.26	0.33	0.38	0.40	0.39	0.36	0.43	0.50	0.47	0.42	0.42	0.41	0.42	0.41
**1570**	0.00	0.01	0.03	0.05	0.13	0.19	0.22	0.26	0.33	0.38	0.40	0.39	0.36	0.43	0.49	0.46	0.41	0.42	0.41	0.42	0.40
**1575**	0.00	0.01	0.03	0.05	0.13	0.20	0.23	0.26	0.34	0.38	0.40	0.39	0.37	0.43	0.48	0.45	0.43	0.44	0.42	0.42	0.42
**1580**	0.00	0.01	0.03	0.05	0.14	0.20	0.23	0.27	0.35	0.39	0.41	0.40	0.38	0.44	0.49	0.47	0.45	0.46	0.45	0.45	0.44
**1585**	0.00	0.01	0.03	0.05	0.13	0.20	0.23	0.26	0.34	0.39	0.40	0.39	0.37	0.43	0.49	0.46	0.43	0.44	0.43	0.42	0.42
**1590**	0.00	0.01	0.03	0.05	0.13	0.20	0.22	0.26	0.34	0.38	0.40	0.39	0.37	0.43	0.48	0.46	0.43	0.44	0.42	0.43	0.42
**1595**	0.00	0.02	0.04	0.06	0.15	0.22	0.26	0.30	0.39	0.44	0.45	0.45	0.42	0.49	0.54	0.54	0.54	0.55	0.54	0.53	0.53
**1600**	0.00	0.02	0.04	0.07	0.16	0.24	0.27	0.32	0.41	0.47	0.49	0.48	0.45	0.52	0.57	0.59	0.59	0.60	0.59	0.58	0.58
**1605**	0.02	0.08	0.13	0.18	0.31	0.42	0.47	0.53	0.63	0.68	0.69	0.69	0.68	0.72	0.74	0.75	0.77	0.77	0.77	0.76	0.76
**1610**	0.01	0.04	0.07	0.10	0.22	0.31	0.35	0.40	0.51	0.57	0.59	0.58	0.56	0.62	0.66	0.68	0.70	0.70	0.69	0.68	0.68
**1615**	0.00	0.02	0.05	0.08	0.18	0.27	0.31	0.36	0.46	0.52	0.54	0.53	0.51	0.57	0.62	0.65	0.66	0.67	0.66	0.65	0.65
**1620**	0.00	0.01	0.03	0.05	0.13	0.20	0.23	0.26	0.34	0.39	0.40	0.40	0.37	0.43	0.49	0.46	0.44	0.44	0.43	0.43	0.42
**1625**	0.00	0.01	0.03	0.05	0.13	0.20	0.22	0.26	0.34	0.38	0.39	0.39	0.36	0.43	0.48	0.45	0.42	0.43	0.42	0.41	0.41
**1630**	0.00	0.01	0.03	0.05	0.13	0.19	0.22	0.26	0.33	0.38	0.39	0.39	0.36	0.43	0.49	0.45	0.41	0.42	0.41	0.41	0.40
**1635**	0.00	0.01	0.03	0.05	0.13	0.19	0.22	0.25	0.33	0.38	0.40	0.39	0.35	0.44	0.53	0.58	0.50	0.50	0.50	0.52	0.51
**1640**	0.00	0.01	0.03	0.04	0.11	0.16	0.19	0.22	0.29	0.34	0.35	0.35	0.31	0.39	0.48	0.53	0.48	0.47	0.47	0.49	0.47
**1645**	0.00	0.00	0.00	0.00	0.00	0.00	0.00	0.00	0.01	0.01	0.01	0.01	0.01	0.01	0.01	0.01	0.01	0.01	0.01	0.01	0.01
**1650**	0.00	0.00	0.00	0.00	0.00	0.00	0.00	0.00	0.00	0.00	0.00	0.00	0.00	0.00	0.00	0.00	0.00	0.00	0.00	0.00	0.00

t_1 \ t_2	1555	1560	1565	1570	1575	1580	1585	1590	1595	1600	1605	1610	1615	1620	1625	1630	1635	1640	1645	1650
**1450**	1.00	1.00	1.00	1.00	1.00	1.00	1.00	1.00	1.00	1.00	0.98	0.99	1.00	1.00	1.00	1.00	1.00	1.00	1.00	1.00
**1455**	0.99	0.99	0.99	0.99	0.99	0.99	0.99	0.99	0.98	0.98	0.92	0.96	0.98	0.99	0.99	0.99	0.99	0.99	1.00	1.00
**1460**	0.97	0.97	0.97	0.97	0.97	0.97	0.97	0.97	0.96	0.96	0.87	0.93	0.95	0.97	0.97	0.97	0.97	0.97	1.00	1.00
**1465**	0.95	0.95	0.95	0.95	0.95	0.95	0.95	0.95	0.94	0.93	0.82	0.90	0.92	0.95	0.95	0.95	0.95	0.96	1.00	1.00
**1470**	0.87	0.87	0.87	0.87	0.87	0.86	0.87	0.87	0.85	0.84	0.69	0.78	0.82	0.87	0.87	0.87	0.87	0.89	1.00	1.00
**1475**	0.80	0.81	0.81	0.81	0.80	0.80	0.80	0.80	0.78	0.76	0.58	0.69	0.73	0.80	0.80	0.81	0.81	0.84	1.00	1.00
**1480**	0.78	0.78	0.78	0.78	0.77	0.77	0.77	0.78	0.74	0.73	0.53	0.65	0.69	0.77	0.78	0.78	0.78	0.81	1.00	1.00
**1485**	0.74	0.74	0.74	0.74	0.74	0.73	0.74	0.74	0.70	0.68	0.47	0.60	0.64	0.74	0.74	0.74	0.75	0.78	1.00	1.00
**1490**	0.66	0.67	0.67	0.67	0.66	0.65	0.66	0.66	0.61	0.59	0.37	0.49	0.54	0.66	0.67	0.67	0.67	0.71	0.99	1.00
**1495**	0.61	0.62	0.62	0.62	0.62	0.61	0.61	0.62	0.56	0.53	0.32	0.43	0.48	0.61	0.62	0.62	0.62	0.66	0.99	1.00
**1500**	0.59	0.61	0.60	0.61	0.60	0.59	0.60	0.60	0.55	0.51	0.31	0.41	0.46	0.60	0.61	0.61	0.60	0.65	0.99	1.00
**1505**	0.60	0.61	0.61	0.61	0.61	0.60	0.61	0.61	0.55	0.52	0.31	0.42	0.47	0.60	0.61	0.61	0.61	0.65	0.99	1.00
**1510**	0.63	0.64	0.64	0.64	0.63	0.62	0.63	0.63	0.58	0.55	0.32	0.44	0.49	0.63	0.64	0.64	0.65	0.69	0.99	1.00
**1515**	0.55	0.57	0.57	0.57	0.57	0.56	0.57	0.57	0.51	0.48	0.28	0.38	0.43	0.57	0.57	0.57	0.56	0.61	0.99	1.00
**1520**	0.47	0.52	0.50	0.51	0.52	0.51	0.51	0.52	0.46	0.43	0.26	0.34	0.38	0.51	0.52	0.51	0.47	0.52	0.99	1.00
**1525**	0.47	0.55	0.53	0.54	0.55	0.53	0.54	0.54	0.46	0.41	0.25	0.32	0.35	0.54	0.55	0.55	0.42	0.47	0.99	1.00
**1530**	0.54	0.59	0.58	0.59	0.57	0.55	0.57	0.57	0.46	0.41	0.23	0.30	0.34	0.56	0.58	0.59	0.50	0.52	0.99	1.00
**1535**	0.54	0.58	0.58	0.58	0.56	0.54	0.56	0.56	0.45	0.40	0.23	0.30	0.33	0.56	0.57	0.58	0.50	0.53	0.99	1.00
**1540**	0.55	0.59	0.59	0.59	0.58	0.55	0.57	0.58	0.46	0.41	0.23	0.31	0.34	0.57	0.58	0.59	0.50	0.53	0.99	1.00
**1545**	0.53	0.59	0.58	0.58	0.58	0.55	0.58	0.57	0.47	0.42	0.24	0.32	0.35	0.57	0.59	0.59	0.48	0.51	0.99	1.00
**1550**	0.54	0.60	0.59	0.60	0.58	0.56	0.58	0.58	0.47	0.42	0.24	0.32	0.35	0.58	0.59	0.60	0.49	0.53	0.99	1.00
**1555**	0.00	0.57	0.56	0.56	0.56	0.54	0.56	0.56	0.47	0.42	0.25	0.32	0.35	0.56	0.57	0.57	0.45	0.49	0.99	1.00
**1560**	0.43	0.00	0.48	0.49	0.50	0.49	0.50	0.50	0.44	0.40	0.24	0.32	0.35	0.50	0.51	0.50	0.41	0.47	0.99	1.00
**1565**	0.44	0.52	0.00	0.51	0.52	0.50	0.52	0.52	0.45	0.41	0.24	0.32	0.35	0.52	0.53	0.52	0.41	0.47	0.99	1.00
**1570**	0.44	0.51	0.49	0.00	0.51	0.50	0.51	0.51	0.44	0.40	0.24	0.31	0.35	0.51	0.52	0.51	0.41	0.47	0.99	1.00
**1575**	0.44	0.50	0.48	0.49	0.00	0.49	0.50	0.50	0.45	0.41	0.25	0.33	0.36	0.50	0.51	0.50	0.42	0.48	0.99	1.00
**1580**	0.46	0.51	0.50	0.50	0.51	0.00	0.51	0.51	0.46	0.42	0.25	0.34	0.37	0.51	0.52	0.51	0.45	0.51	0.99	1.00
**1585**	0.44	0.50	0.48	0.49	0.50	0.49	0.00	0.50	0.45	0.41	0.25	0.33	0.36	0.50	0.51	0.50	0.42	0.49	0.99	1.00
**1590**	0.44	0.50	0.48	0.49	0.50	0.49	0.50	0.00	0.44	0.41	0.24	0.32	0.36	0.50	0.51	0.50	0.42	0.49	0.99	1.00
**1595**	0.53	0.56	0.55	0.56	0.55	0.55	0.55	0.56	0.00	0.46	0.28	0.37	0.42	0.55	0.56	0.56	0.53	0.59	0.99	1.00
**1600**	0.58	0.60	0.59	0.60	0.59	0.58	0.59	0.59	0.54	0.00	0.29	0.40	0.45	0.59	0.60	0.60	0.59	0.64	0.99	1.00
**1605**	0.75	0.76	0.76	0.76	0.75	0.75	0.75	0.76	0.72	0.71	0.00	0.62	0.67	0.75	0.76	0.76	0.77	0.79	1.00	1.00
**1610**	0.68	0.68	0.68	0.69	0.67	0.66	0.67	0.68	0.63	0.60	0.38	0.00	0.55	0.67	0.68	0.68	0.69	0.73	0.99	1.00
**1615**	0.65	0.65	0.65	0.65	0.64	0.63	0.64	0.64	0.58	0.55	0.33	0.45	0.00	0.64	0.65	0.65	0.66	0.70	0.99	1.00
**1620**	0.44	0.50	0.48	0.49	0.50	0.49	0.50	0.50	0.45	0.41	0.25	0.33	0.36	0.00	0.51	0.50	0.43	0.49	0.99	1.00
**1625**	0.43	0.49	0.47	0.48	0.49	0.48	0.49	0.49	0.44	0.40	0.24	0.32	0.35	0.49	0.00	0.49	0.42	0.48	0.99	1.00
**1630**	0.43	0.50	0.48	0.49	0.50	0.49	0.50	0.50	0.44	0.40	0.24	0.32	0.35	0.50	0.51	0.00	0.41	0.47	0.99	1.00
**1635**	0.55	0.59	0.59	0.59	0.58	0.55	0.58	0.58	0.47	0.41	0.23	0.31	0.34	0.57	0.58	0.59	0.00	0.53	0.99	1.00
**1640**	0.51	0.53	0.53	0.53	0.52	0.49	0.51	0.51	0.41	0.36	0.21	0.27	0.30	0.51	0.52	0.53	0.47	0.00	0.98	1.00
**1645**	0.01	0.01	0.01	0.01	0.01	0.01	0.01	0.01	0.01	0.01	0.00	0.01	0.01	0.01	0.01	0.01	0.01	0.02	0.00	0.95
**1650**	0.00	0.00	0.00	0.00	0.00	0.00	0.00	0.00	0.00	0.00	0.00	0.00	0.00	0.00	0.00	0.00	0.00	0.00	0.05	0.00

Figure C. OxCal Order probabilities for the IntCal13 values 1450 to 1650 compared with each other if treated as independent data within a Phase. If the known order (calendar ordered data 1450, 1455, 1460 … 1650) were perfectly resolved, then all the red shaded cells would be above the grey diagonal line (top right) and all the green shaded cells would be below the line (bottom left). The main regions of discrepancy for the paired comparisons are indicated by the orange boxes (top right) and corresponding cyan boxes (bottom left) (relevant to years 1500-1510, 1520-1525, 1550, 1560, 1590-1615, 1635-1640).

We may note one important observation, however. The key ambiguous periods identified in Figures B and C vary in duration. Some are in fact quite short. For example: just 1560, or just 1635-1640, or just 1510, or just 1520-1525. Only 1595-1615 is a longer sustained period of challenge in the target interval for this paper. This suggests:

(i) if we have a site Phase that is not very short (e.g. under 20 years),

(ii) if we have a good set of dates that represent most of the site Phase's duration (from early, middle and later periods), and

(iii) if we have enough quality (accurate and precise) data to closely resolve this Phase in radiocarbon terms

that we may then be able to overcome the challenges or ambiguities of an Order analysis. (In contrast, if a site Phase is very short, or if the dates from a site Phase happen to derive from one particular context, like a pit, that represents a short period, and this site Phase or short interval happens to be about e.g. 1520-1525, then there will inevitably be a misleading Order result for this site Phase versus mid-16th century contexts and end 16th and earlier 17th century contexts.)

Model D1: Order Test Interval Constraint 0-80 years

t_1	Probability $t_1 < t_2$				
	Date Site A Int	Date Site B Int	Date Site C Int	Date Site D Int	Date Site E Int
Date Site A Int	0.00	**0.54**	**0.59**	**0.65**	**0.70**
Date Site B Int	0.46	0.00	**0.59**	**0.70**	**0.77**
Date Site C Int	0.41	0.41	0.00	**0.62**	**0.72**
Date Site D Int	0.35	0.30	0.38	0.00	**0.63**
Date Site E Int	0.30	0.23	0.28	0.37	0.00

Model D2: Order Test Interval Constraint 0-120 years

t_1	Probability $t_1 < t_2$				
	Date Site A Int	Date Site B Int	Date Site C Int	Date Site D Int	Date Site E Int
Date Site A Int	0.00	**0.53**	**0.57**	**0.63**	**0.68**
Date Site B Int	0.47	0.00	**0.57**	**0.67**	**0.73**
Date Site C Int	0.43	0.43	0.00	**0.60**	**0.69**
Date Site D Int	0.37	0.33	0.40	0.00	**0.60**
Date Site E Int	0.32	0.27	0.31	0.40	0.00

Model D3: Order Test no Interval constraint

t_1	Probability $t_1 < t_2$				
	Date Site A no Int	Date Site B no Int	Date Site C no Int	Date Site D no Int	Date Site E no Int
Date Site A no Int	0.00	0.52	0.56	0.61	0.65
Date Site B no Int	0.48	0.00	0.56	0.64	0.69
Date Site C no Int	0.44	0.44	0.00	0.58	0.65
Date Site D no Int	0.39	0.36	0.42	0.00	0.59
Date Site E no Int	0.35	0.31	0.35	0.41	0.00

Figure D. Order probabilities for Models D1 to D3 (a revision of Model C in Figure A with data now ±10 ^{14}C years). Bold entries show better results for Models D1 and D2 versus Models D3. Data shown from typical model runs, all model runs vary slightly.

To give an example, let us re-consider Model C in Figure A. There we assumed 5 radiocarbon dates from across each of a set of five 40-year site Phases. The 40-year Phases with 5 dates spread across these should partly bridge across the problem intervals. Let us further assume that we can achieve radiocarbon resolution that is another 33% better than in Model C in Figure A—for example by running additional date replicates on split samples to achieve coherent precise weighted average values—such that the dating error is now ±10 ^{14}C years. If so, we can in fact more or less achieve an entirely satisfactory Order analysis (with or without an Interval constraint) for the hypothetical case above re-running the new Model (now Model D): see Figure D. The no constraint Model D3 achieves the correct Order and the Interval constraint Models D1 and D2 find the same correct order but (progressively) a little more clearly. Again, the use of the Interval constraint is useful, but not determinative. Such near-perfect resolution is of course often

impractical in real-world terms—unless large sets of dates are run on carefully selected samples—but such a hypothetical model indicates a path forward. Another approach (or an additional approach) is to explore bringing other constraints to bear, for example TPQ information from dates on charcoal which may be able to help to resolve such cases.

Model E1 with 0 to 80 years Interval Constraint. Amodel=74, Aoverall=74

t_1	Probability $t_1 < t_2$: Date Site A Int	Date Site B Int	Date Site C Int	Date Site D Int	Date Site E Int	Site Phase Interval Durations (calendar years)	68.2%	95.4%	Site Phase Date Estimates: 68.2%	95.4%
Date Site A Int	0.00	**0.55**	**0.58**	**0.61**	**0.66**	Site A 1500-1540	44 to 80	18 to 80	1496-1576 (60.6%)	1490-1640
Date Site B Int	0.45	0.00	**0.55**	**0.62**	**0.71**	Site B 1520-1560	20 to 80	5 to 80	1520-1575	1502-1596 (88.6%)
Date Site C Int	0.42	0.45	0.00	**0.57**	**0.68**	Site C 1540-1580	7 to 80	0 to 80	1523-1579	1505-1600 (87.7%)
Date Site D Int	0.39	0.39	0.43	0.00	**0.62**	Site D 1550-1590	0 to 80	0 to 80	1539-1592 (61.7%)	1506-1605 (85.8%)
Date Site E Int	0.34	0.29	0.32	0.38	0.00	Site E 1560-1600	0 to 80	0 to 80	1557-1604 (56.4%)	1502-1635

Model E2 with No Interval Constraints. Amodel=80, Aoverall=81

t_1	Probability $t_1 < t_2$: Date Site A NO Int	Date Site B NO Int	Date Site C NO Int	Date Site D NO Int	Date Site E NO Int	Site Phase	68.2%	95.4%	68.2%	95.4%
Date Site A NO Int	0.00	0.52	0.54	0.57	0.61	Site A 1500-1540	37 to 142	4 to 228	1499-1601	1468-1651
Date Site B NO Int	0.48	0.00	0.53	0.58	0.63	Site B 1520-1560	4 to 91	0 to 173	1516-1582	1495-1643
Date Site C NO Int	0.46	0.47	0.00	0.55	0.61	Site C 1540-1580	0 to 87	0 to 173	1519-1586	1497-1642
Date Site D NO Int	0.43	0.42	0.45	0.00	0.57	Site D 1550-1590	0 to 89	0 to 175	1520-1593	1496-1642
Date Site E NO Int	0.39	0.37	0.39	0.43	0.00	Site E 1560-1600	0 to 89	0 to 183	1553-1608 (53.0%)	1490-1640

Figure E. Comparison of the Order probabilities, Site Phase Interval durations (calendar years) and site Phase Date estimates (calendar dates AD) for sites A to E in Model E with a 0 to 80 years Interval constraint applied to each site Phase versus with no Interval constraint. Bold entries show instances of clearer Order outcomes in Model E1 versus Model E2. The site Phase Date estimates show the modelled 68.2% and 95.4% probability ranges or the most likely sub-range within these. Data shown from typical model runs – each model run varies slightly.

The application of a plausible but conservative Interval constraint also has the benefit of potentially helping better to resolve dating in some ambiguous cases and achieving more successful model runs. We give an example. Let us take Model C above, but modify it by including a little realistic 'noise' in each site Phase dataset. We add one date that is (given the measurement precision of the data in the site Phases) 1SD (15 ^{14}C years) older than the oldest date in each set and one date that is 1SD more recent than the most recent date in each set. We then compare an analysis of the Model (now Model E) with a 0 to 80 years Interval constraint applied to each of the site Phases (Model E1) versus the analysis with no Interval constraint (Model E2): Figure E.

A comparison of the results from Models E1 versus Model E2 in Figure E illustrates the role of the Interval constraint. The Order analysis finds the same (and correct) order in both cases, but

the correct probabilities are clearer in Model E1 with the Interval constraint applied. We can further observe another benefit from the constraint applied to the site Phase Interval durations in Model E1. The results from Model E1 provide more satisfactory and appropriate Date estimates for each site Phase when we compare the results from Model E1 versus those from Model E2 and compare both versus the 'known' site Phase dates (Figure E).

With regard to the current paper, we observe especially the challenge of the early 16th century, and identify this as a caveat needing further clarification (e.g. Cayadutta and Garoga, especially). This is plausible through both additional high quality accurate and precise radiocarbon dates on securely associated short-lived samples, and from the dating of targeted charcoal samples which offer a specific TPQ, for example from a short radiocarbon wiggle-match (as employed at Warminster in [13, 14]). The early 17th century is not a focus of this paper, but this instance is likely resolved via the addition of historic information and constraints (see main text, Methods).

	**Probability $t_1 < t_2$**										
**t_1**	**Date Snell Pits**	**Date Pethick**	**Date Second Woods**	**Date Getman**	**Date Elwood**	**Date Smith-Pagerie**	**Date Otstungo**	**Date Klock**	**Date Cayadutta Midden**	**Date Garoga**	**Date Wormuth**
**Date Snell Pits**	0.00	**0.96**	**0.99**	1.00	1.00	1.00	1.00	1.00	1.00	1.00	1.00
**Date Pethick**	0.04	0.00	**0.95**	**0.97**	**0.97**	**0.99**	**0.99**	**0.99**	**0.99**	**0.99**	**0.98**
**Date Second Woods**	0.01	0.05	0.00	*0.56*	**0.68**	**0.88**	**0.86**	**0.95**	**0.92**	**0.94**	**0.89**
**Date Getman**	0.00	0.03	0.44	0.00	0.59	**0.77**	**0.80**	**0.89**	**0.87**	**0.92**	**0.86**
**Date Elwood**	0.00	0.03	0.32	0.41	0.00	**0.74**	**0.76**	**0.87**	**0.84**	**0.89**	**0.83**
**Date Smith-Pagerie**	0.00	0.01	0.12	0.23	0.26	0.00	*0.58*	**0.72**	**0.70**	**0.77**	**0.70**
**Date Otstungo**	0.00	0.01	0.14	0.20	0.24	0.42	0.00	**0.60**	**0.62**	**0.67**	**0.63**
**Date Klock**	0.00	0.01	0.05	0.11	0.13	0.28	0.40	0.00	**0.52**	**0.57**	**0.54**
**Date Cayadutta Midden**	0.00	0.01	0.08	0.13	0.16	0.30	0.38	0.48	0.00	0.53	**0.51**
**Date Garoga**	0.00	0.01	0.06	0.08	0.11	0.23	0.33	0.43	0.47	0.00	**0.50**
**Date Wormuth**	0.00	0.02	0.11	0.14	0.17	0.30	0.37	0.46	0.49	0.50	0.00

Figure F. Model 2 (main text) Order analysis (compare with Table 4) run with no Interval constraint. Bold red = higher value in Model 2 with 0–120 years Interval constraint (Table 4, S6 File). Italics = higher value in one or both of Model 2 with 0–100 years or 0–80 years Interval constraints (Table 4, S6 File). Underline value = case where possible ambiguity evident comparing other model runs with varying Interval constraints (Table 4, S6 File).

Overall, use of an Interval constraint can sometimes enhance clarity in an Order analysis of non-ordered independent site Phases, but, as the discussion above illustrates, care is also necessary. A range of different (conservative) Interval constraints should be considered, and differences in outcomes (likely ambiguous intervals) examined. Even if the Interval periods calculated are unrealistic (too long), a non-constrained model usually should indicate a similar Order analysis (which may be enhanced for a number of elements with a subsequent set of Interval constraint runs). In the main text we thus started with a no constraint model (Model 1), and then considered three different Interval constraint models for Model 2. It is important to note that Model 2 run

with no Interval constraints yields almost the same Order analysis: Figure F (compare to Table 4, S6 File).

If we compare the Figure F results with those for the models in Table 4 and S6 File, we can observe that 43 of the 55 pairs of comparisons (in bold in Figure F) exhibit a similar but more clear-cut probability in the Model 2 run with 0–120 years site Phase Interval constraint (Table 4, S6 File). Two other cases (italics in Figure F) exhibit a more clear-cut result in either or both the 0–100 years or 0–80 years Interval constraint versions of Model 2 (Table 4, S6 File). For example, the Interval constraint models all place Garoga more clearly as older than Wormuth, compared to near equivalence in Figure F (p=0.501 for Garoga as older in Figure 4). The potential more ambiguous or coeval placement of Cayadutta and Garoga (underlined value in Figure F) also comes out through a comparison of the different versions of Model 2 (especially the 0–80 years Interval constraint version: Table 4 and S6 File). Overall, we may observe that the Interval constraint was less than determinative, but did assist, including to achieve model runs that exhibit satisfactory OxCal diagnostic values and more plausible dating estimates.

OxCal runfiles

The OxCal runfiles for Models C1 to C3 are given as examples. Following the information in the text above Models A, B and D can be constructed from these. Models E1 and E2 are also listed below.

Model C1:

```
Plot()
 {
  Phase ("Test")
  {
   Sequence()
   {
    Boundary("Start Site A");
    Phase("Site A1 1500-1540")
    {
     R_Date("1500",349,15);
     R_Date("1510",353,15);
     R_Date("1520",337,15);
     R_Date("1530",306,15);
     R_Date("1540",307,15);
     Date("Date Site A Int");
     Interval(U(0,80));
    };
    Boundary("End Site A");
   };
   Sequence()
   {
    Boundary("Start Site B");
    Phase("Site B 1520-1560")
    {
     R_Date("1520b",337,15);
     R_Date("1530b",306,15);
     R_Date("1540b",307,15);
     R_Date("1550",310,15);
     R_Date("1560",328,15);
```

```
    Date("Date Site B Int");
    Interval(U(0,80));
   };
   Boundary("End Site B");
  };
  Sequence()
  {
   Boundary("Start Site C");
   Phase("Site C 1540-1580")
   {
    R_Date("1540c",307,15);
    R_Date("1550b",310,15);
    R_Date("1560b",328,15);
    R_Date("1570",325,15);
    R_Date("1580",335,15);
    Date("Date Site C Int");
    Interval(U(0,80));
   };
   Boundary("End Site C");
  };
  Sequence()
  {
   Boundary("Start Site D");
   Phase("Site D1 1550-1590")
   {
    R_Date("1550c",310,15);
    R_Date("1560c",328,15);
    R_Date("1570b",325,15);
    R_Date("1580b",335,15);
    R_Date("1590",332,15);
    Date("Date Site D Int");
    Interval(U(0,80));
   };
   Boundary("End Site D");
  };
  Sequence()
  {
   Boundary("Start Site E");
   Phase("Site E 1560-1600")
   {
    R_Date("1560d",328,15);
    R_Date("1570c",325,15);
    R_Date("1580c",335,15);
    R_Date("1590b",332,15);
    R_Date("1600",347,15);
    Date("Date Site E Int");
    Interval(U(0,80));
   };
   Boundary("End Site E");
  };
  Order("Test");
 };
};
```

Model C2:

```
Plot()
```

```
{
 Phase ("Test")
 {
  Sequence()
  {
   Boundary("Start Site A");
   Phase("Site A1 1500-1540")
   {
    R_Date("1500",349,15);
    R_Date("1510",353,15);
    R_Date("1520",337,15);
    R_Date("1530",306,15);
    R_Date("1540",307,15);
    Date("Date Site A Int");
    Interval(U(0,120));
   };
   Boundary("End Site A");
  };
  Sequence()
  {
   Boundary("Start Site B");
   Phase("Site B 1520-1560")
   {
    R_Date("1520b",337,15);
    R_Date("1530b",306,15);
    R_Date("1540b",307,15);
    R_Date("1550",310,15);
    R_Date("1560",328,15);
    Date("Date Site B Int");
    Interval(U(0,120));
   };
   Boundary("End Site B");
  };
  Sequence()
  {
   Boundary("Start Site C");
   Phase("Site C 1540-1580")
   {
    R_Date("1540c",307,15);
    R_Date("1550b",310,15);
    R_Date("1560b",328,15);
    R_Date("1570",325,15);
    R_Date("1580",335,15);
    Date("Date Site C Int");
    Interval(U(0,120));
   };
   Boundary("End Site C");
  };
  Sequence()
  {
   Boundary("Start Site D");
   Phase("Site D1 1550-1590")
   {
    R_Date("1550c",310,15);
    R_Date("1560c",328,15);
    R_Date("1570b",325,15);
    R_Date("1580b",335,15);
```

```
     R_Date("1590",332,15);
     Date("Date Site D Int");
     Interval(U(0,120));
    };
   Boundary("End Site D");
  };
  Sequence()
  {
   Boundary("Start Site E");
   Phase("Site E 1560-1600")
   {
    R_Date("1560d",328,15);
    R_Date("1570c",325,15);
    R_Date("1580c",335,15);
    R_Date("1590b",332,15);
    R_Date("1600",347,15);
    Date("Date Site E Int");
    Interval(U(0,120));
   };
   Boundary("End Site E");
  };
  Order("Test");
 };
};
```

Model C3:

```
Phase ("Test")
  {
   Sequence()
   {
    Boundary("Start Site A");
    Phase("Site A 1500-1540")
    {
     R_Date("1500",349,15);
     R_Date("1510",353,15);
     R_Date("1520",337,15);
     R_Date("1530",306,15);
     R_Date("1540",307,15);
     Date("Date Site A no Int");
    };
    Boundary("End Site A");
   };
   Sequence()
   {
    Boundary("Start Site B");
    Phase("Site B 1520-1560")
    {
     R_Date("1520b",337,15);
     R_Date("1530b",306,15);
     R_Date("1540b",307,15);
     R_Date("1550",310,15);
     R_Date("1560",328,15);
     Date("Date Site B no Int");
    };
    Boundary("End Site B");
   };
```

```
   Sequence()
   {
    Boundary("Start Site C");
    Phase("Site C 1540-1580")
    {
     R_Date("1540c",307,15);
     R_Date("1550b",310,15);
     R_Date("1560b",328,15);
     R_Date("1570",325,15);
     R_Date("1580",335,15);
     Date("Date Site C no Int");
    };
    Boundary("End Site C");
   };
   Sequence()
   {
    Boundary("Start Site D");
    Phase("Site D2 1550-1590")
    {
     R_Date("1550c",310,15);
     R_Date("1560c",328,15);
     R_Date("1570b",325,15);
     R_Date("1580b",335,15);
     R_Date("1590",332,15);
     Date("Date Site D no Int");
    };
    Boundary("End Site D");
   };
   Sequence()
   {
    Boundary("Start Site E");
    Phase("Site E 1560-1600")
    {
     R_Date("1560d",328,15);
     R_Date("1570c",325,15);
     R_Date("1580c",335,15);
     R_Date("1590b",332,15);
     R_Date("1600",347,15);
     Date("Date Site E no Int");
    };
    Boundary("End Site E");
   };
   Order("Test");
  };
 };
```

Model E1:

```
Plot()
 {
  Phase ("Test")
  {
   Sequence()
   {
    Boundary("Start Site A");
    Phase("Site A 1500-1540")
    {
```

```
  R_Date("Noise Early A 1SD",368,15);
  R_Date("1500",349,15);
  R_Date("1510",353,15);
  R_Date("1520",337,15);
  R_Date("1530",306,15);
  R_Date("1540",307,15);
  R_Date("Noise Late A 1SD",291,15);
  Date("Date Site A Int");
  Interval(U(0,80));
 };
 Boundary("End Site A");
};
Sequence()
{
 Boundary("Start Site B");
 Phase("Site B 1520-1560")
 {
  R_Date("Noise Early B 1SD",352,15);
  R_Date("1520b",337,15);
  R_Date("1530b",306,15);
  R_Date("1540b",307,15);
  R_Date("1550",310,15);
  R_Date("1560",328,15);
  R_Date("Noise Late B 1SD",291,15);
  Date("Date Site B Int");
  Interval(U(0,80));
 };
 Boundary("End Site B");
};
Sequence()
{
 Boundary("Start Site C");
 Phase("Site C 1540-1580")
 {
  R_Date("Noise Early C 1SD",350,15);
  R_Date("1540c",307,15);
  R_Date("1550b",310,15);
  R_Date("1560b",328,15);
  R_Date("1570",325,15);
  R_Date("1580",335,15);
  R_Date("Noise Late C 1SD",292,15);
  Date("Date Site C Int");
  Interval(U(0,80));
 };
 Boundary("End Site C");
};
Sequence()
{
 Boundary("Start Site D");
 Phase("Site D 1550-1590")
 {
  R_Date("Noise Early D 1SD",350,15);
  R_Date("1550c",310,15);
  R_Date("1560c",328,15);
  R_Date("1570b",325,15);
  R_Date("1580b",335,15);
  R_Date("1590",332,15);
```

```
    R_Date("Noise Late D 1SD",295,15);
    Date("Date Site D Int");
    Interval(U(0,80));
   };
   Boundary("End Site D");
  };
  Sequence()
  {
   Boundary("Start Site E");
   Phase("Site E 1560-1600")
   {
    R_Date("Noise Early E 1SD",362,15);
    R_Date("1560d",328,15);
    R_Date("1570c",325,15);
    R_Date("1580c",335,15);
    R_Date("1590b",332,15);
    R_Date("1600",347,15);
    R_Date("Noise Late E 1SD",310,15);
    Date("Date Site E Int");
    Interval(U(0,80));
   };
   Boundary("End Site E");
  };
  Order("Test");
 };
};
```

Model E2:

```
Plot()
 {
  Phase ("Test")
  {
   Sequence()
   {
    Boundary("Start Site A");
    Phase("Site A 1500-1540")
    {
     R_Date("Noise Early A 1SD",368,15);
     R_Date("1500",349,15);
     R_Date("1510",353,15);
     R_Date("1520",337,15);
     R_Date("1530",306,15);
     R_Date("1540",307,15);
     R_Date("Noise Late A 1SD",291,15);
     Date("Date Site A NO Int");
     Interval();
    };
    Boundary("End Site A");
   };
   Sequence()
   {
    Boundary("Start Site B");
    Phase("Site B 1520-1560")
    {
     R_Date("Noise Early B 1SD",352,15);
     R_Date("1520b",337,15);
```

```
  R_Date("1530b",306,15);
  R_Date("1540b",307,15);
  R_Date("1550",310,15);
  R_Date("1560",328,15);
  R_Date("Noise Late B 1SD",291,15);
  Date("Date Site B NO Int");
  Interval();
 };
 Boundary("End Site B");
};
Sequence()
{
 Boundary("Start Site C");
 Phase("Site C 1540-1580")
 {
  R_Date("Noise Early C 1SD",350,15);
  R_Date("1540c",307,15);
  R_Date("1550b",310,15);
  R_Date("1560b",328,15);
  R_Date("1570",325,15);
  R_Date("1580",335,15);
  R_Date("Noise Late C 1SD",292,15);
  Date("Date Site C NO Int");
  Interval();
 };
 Boundary("End Site C");
};
Sequence()
{
 Boundary("Start Site D");
 Phase("Site D 1550-1590")
 {
  R_Date("Noise Early D 1SD",350,15);
  R_Date("1550c",310,15);
  R_Date("1560c",328,15);
  R_Date("1570b",325,15);
  R_Date("1580b",335,15);
  R_Date("1590",332,15);
  R_Date("Noise Late D 1SD",295,15);
  Date("Date Site D NO Int");
  Interval();
 };
 Boundary("End Site D");
};
Sequence()
{
 Boundary("Start Site E");
 Phase("Site E 1560-1600")
 {
  R_Date("Noise Early E 1SD",362,15);
  R_Date("1560d",328,15);
  R_Date("1570c",325,15);
  R_Date("1580c",335,15);
  R_Date("1590b",332,15);
  R_Date("1600",347,15);
  R_Date("Noise Late E 1SD",310,15);
  Date("Date Site E NO Int");
```

```
    Interval();
   };
   Boundary("End Site E");
  };
  Order("Test");
 };
};
```
